# RAN-S100A10-EGFR axis facilitates papillary thyroid cancer metastasis by PI3K/AKT signaling

**DOI:** 10.1038/s41419-026-08649-6

**Published:** 2026-04-16

**Authors:** Wenbin Song, Zhaoyi Liu, Cangchang Shi, Guixin Wang, Long He, Zhaohui Chen, Yingxi Li, Yue Yu, Ruoyu Jiang, Xiaoning Wang, Ke Zhao, Ziyi Chen, Yao Tian, Feng Qi, Yizeng Wang, Xianghui He

**Affiliations:** 1 Department of General Surgery, Tianjin Medical University General Hospital, Tianjin Key Laboratory of Precise Vascular Reconstruction and Organ Function Repair, Tianjin General Surgery Institute, Tianjin, China; 2 the First Department of Breast Cancer, Key Laboratory of Cancer Prevention and Therapy, Tianjin’s Clinical Research Center for Cancer, National Clinical Research Center for Cancer, Key Laboratory of Breast Cancer Prevention and Therapy, Tianjin Medical University Cancer Institute and Hospital, Tianjin Medical University, Tianjin, China; 3 Health Science Center, Ningbo University, Ningbo, Zhejiang China; 4 Department of Thoracic Oncology, Tianjin Lung Cancer Center, Tianjin Cancer Institute & Hospital, Tianjin Medical University, Tianjin, China; 5 Department of Thoracic Surgery, The Affiliated LiHuiLi Hospital of Ningbo University, Ningbo, Zhejiang China

**Keywords:** Thyroid cancer, Oncogenesis, Thyroid diseases

## Abstract

Background: The incidence of thyroid cancer has been increasing in recent years, with papillary thyroid carcinoma (PTC) accounting for the majority of cases. Accumulating studies have demonstrated that S100A10 acts as an oncogene in the progression of various malignancies. However, the function and specific mechanisms of S100A10 in thyroid cancer remain poorly defined. Methods: Single-cell RNA sequencing data of PTC from public databases were analyzed to screen differentially expressed genes (DEGs), among which S100A10 emerged as a potential biomarker associated with PTC metastasis and prognosis. The expression of S100A10 in tissues and cell lines were validated by RT-qPCR and western blot. Protein-protein interactions were confirmed using mass spectrometry analysis and co-immunoprecipitation. The subcellular localization of the protein was determined by immunofluorescence. Stable PTC cell lines overexpressing S100A10 were constructed and followed by transwell assays, wound-healing assays, and western blot for EMT capability detection. Results: S100A10 was found to play essential roles in tumor metastasis and was associated with unfavorable prognosis in patients with PTC. S100A10 was expressed higher in both PTC tissues and cells. Furthermore, both in vitro and in vivo experiments confirmed that S100A10 activates the PI3K/AKT signaling and promotes EMT in PTC cells, enhancing the invasive capabilities of tumors. S100A10 could interact with both RAN and EGFR intracellularly, forming a RAN-S100A10-EGFR regulatory axis. Finally, several potential drugs targeting S100A10 were identified for further in-depth research. Conclusion: These findings clarify the role of S100A10 and RAN in PTC progression and highlight their potential as therapeutic targets, linking EMT with PI3K/AKT signaling.

## Introduction

According to the GLOBOCAN 2020 Cancer Incidence and Mortality database by the International Agency for Research on Cancer (IARC), part of the World Health Organization (WHO), thyroid cancer ranks ninth in global cancer incidence rates. The global incidence of thyroid cancer has continued to rise over the past four decades [1]. Among all malignant thyroid neoplasms, papillary thyroid carcinoma (PTC) is the most prevalent well-differentiated carcinoma, constituting approximately 80–85% of all thyroid cancers [2, 3]. While the majority of patients exhibit a favorable prognosis, a subset may encounter recurrence or distant metastasis during follow-up. Additionally, some may present with lateral cervical lymph node metastasis and distant metastasis at the time of initial diagnosis [4, 5]. This condition can lead to a reduced quality of life, a poorer prognosis, and increased treatment challenges [6]. Therefore, investigating the molecular mechanisms of lymph node metastasis in PTC is essential.

With the advancement of sequencing technology, scholars are now able to observe cellular-level changes in cancer tissue through single-cell RNA sequencing (scRNA-seq) technology, which offers novel strategies for cancer diagnosis and treatment [7]. Single-cell technology is a valuable method for exploring the occurrence and progression of tumors [8]. While techniques such as microarray sequencing reflect expression profile changes at the tissue level, single-cell analysis can examine the expression profile changes of individual cells. Consequently, compared to other technologies, single-cell methods can more effectively capture the continuous progression of disease development [9]. Indeed, it is highly conceivable that investigating the dynamic changes in the tumor microenvironment from primary PTC to metastatic PTC from a single-cell perspective can contribute to a deeper understanding of the biological processes involved in PTC metastasis.

S100A10 is a member of the S100 families, a group of small molecular weight calcium-binding proteins function in essential biological processes, including cell proliferation, apoptosis, migration, cytoskeleton remodeling, and extracellular matrix remodeling through interactions with target proteins [10, 11]. Many members of the S100 family, including S100A10 itself, are located in the epidermal differentiation complex (EDC) region on human chromosome 1q21. In tumors, abnormalities in the expression of this region and genomic alterations are closely associated with the epithelial-mesenchymal transition (EMT) [12, 13]. Current research indicates that S100A10 acts as an oncogene in the development of various malignant tumors. For example, in breast cancer, S100A10 regulated KDM6A-OCT4 axis to affect breast cancer stem cell properties [14]. Additionally, S100A10 promotes the initiation and progression of hepatocellular carcinoma (HCC) via transfer in extracellular vesicles and regulation of their protein cargos [15]. Furthermore, S100A10 promotes the proliferation, migration, and adhesion of pancreatic ductal adenocarcinoma cells through the JNK/LAMB3-LAMC2 axis [16]. However, its role and specific mechanisms in thyroid cancer remain unclear.

Herein, we performed an integrative analysis of tumor microenvironment heterogeneity in primary and metastatic PTC, identifying RAN-S100A10-complex enhances PTC invasiveness by stabilizing EGFR and activating the PI3K/AKT pathway, which reveal potential mechanisms of PTC metastasis and suggest therapeutic targets for clinical intervention.

## Results

### Identification of distinct cell types in PTC via single-cell RNA sequencing

**Fig. 1:**
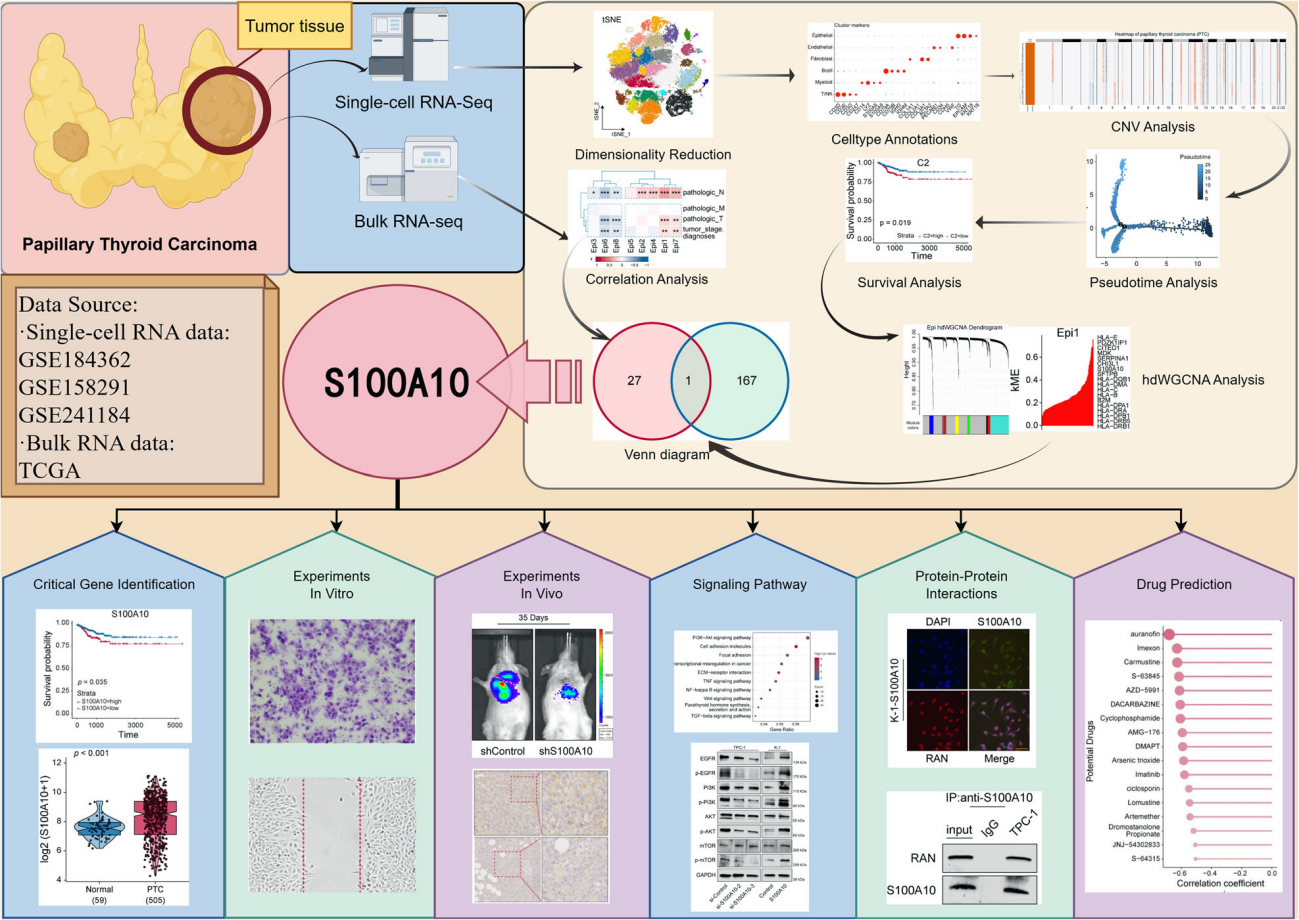
Schematic design of the study. GSE Gene Expression Omnibus series, TCGA The Cancer Genome Atlas.

**Fig. 2:**
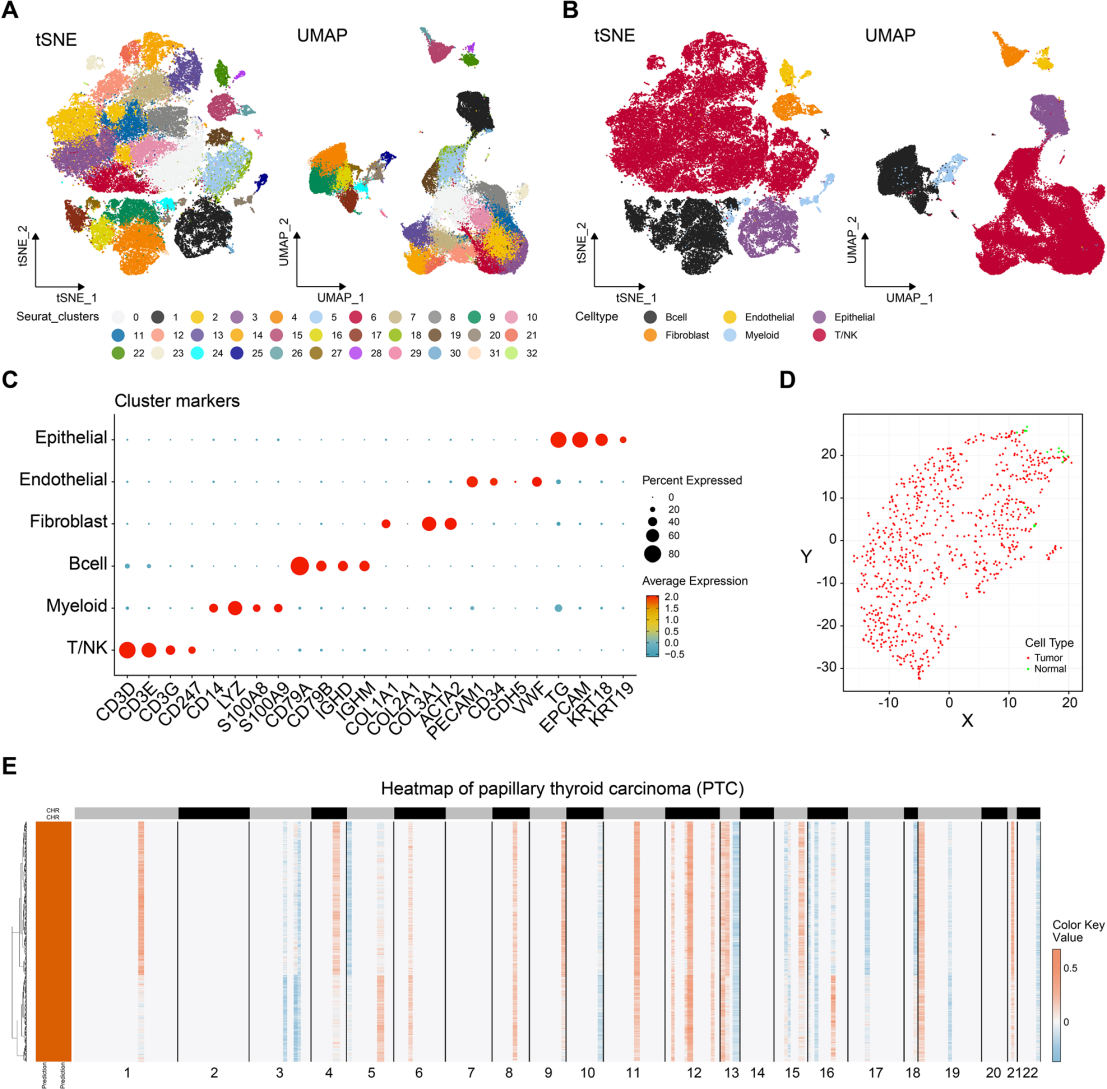
Different cell types in PTC were identified by single-cell sequencing. **A** tSNE and UMAP scatter plots displayed 33 different cell clusters in PTC samples. **B** tSNE and UMAP scatter plots displayed 6 different cell types in PTC samples. **C** Dot plots showed expression levels of marker genes used to note 6 different cell types. **D** Epithelial Cell Benignancy and Malignancy in Primary PTC Samples. **E** CNV in 794 PTC cells.

A total of 64,787 cells were subjected to quality control procedures utilizing the “Seurat” R package. Data were normalized using SCTransform, and the top 3000 highly variable genes were selected for dimensionality reduction (Fig. 1). High-quality cells were visualized using two dimensionality reduction methods (tSNE and UMAP), as shown in Fig. 2A, where different cell clusters were significantly distinguished in space. Subsequently, the cell clusters were annotated using markers from public databases and published studies [17]. The cells were further classified into six distinct cell types: T/NK cells, B cells, fibroblasts, endothelial cells, myeloma cells, and epithelial cells (Fig. 2B). The cell markers used for annotation are shown in Fig. 2C. To further investigate the epithelial cells, the “SCEVAN” R package was utilized to discern the benign and malignant nature of epithelial cells in primary PTC samples (Fig. 2D). A total of 16 normal epithelial cells and 794 tumor cells were identified. Through the analysis of copy-number variant (CNV) in 794 tumor cells (Fig. 2E), primary tumor cells exhibited varying degrees of amplification and deletion on the chromosomes. In summary, we successfully identified six principal cell types for further exploration.

### Cell trajectory and characteristics of various clusters of PTC cells

**Fig. 3:**
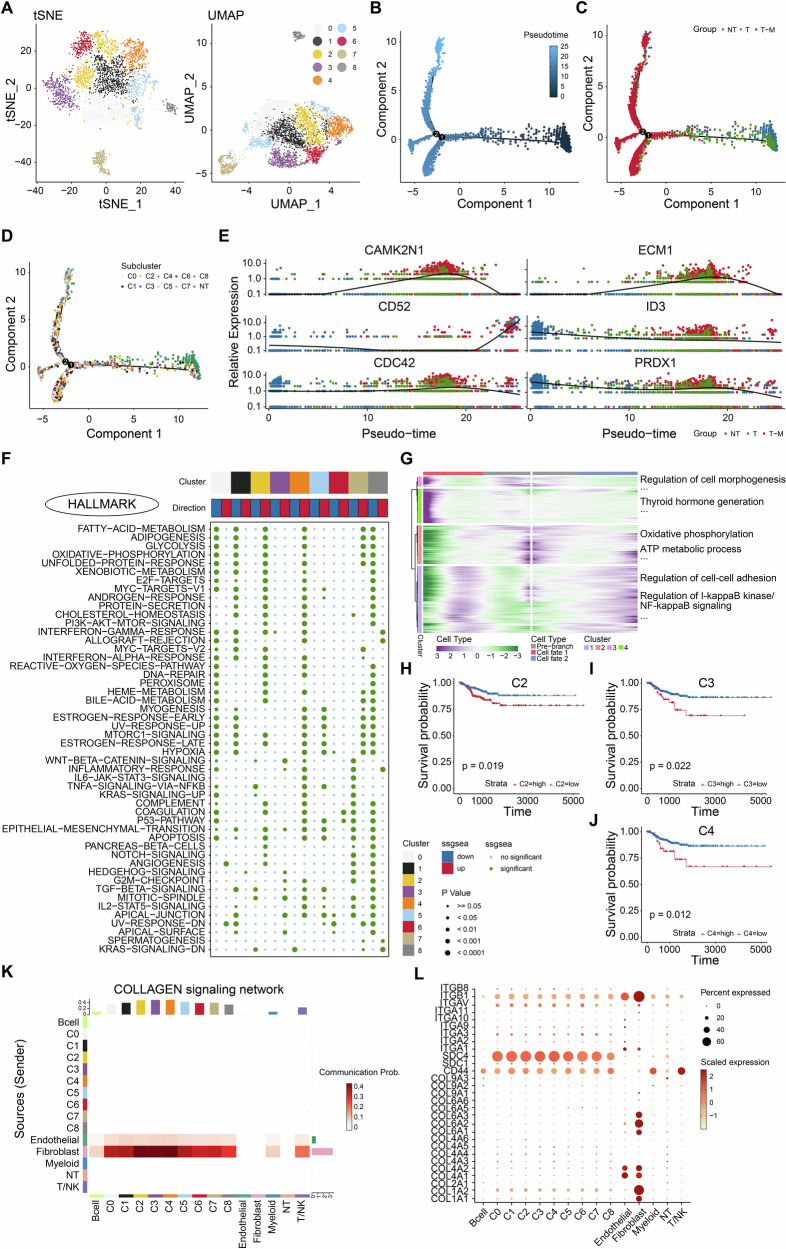
Cell trajectory and characteristics of various clusters of PTC tumor cells. **A** tSNE and UMAP scatter plots displayed 9 different clusters in PTC epithelial cells. The trajectory of primary PTC cells evolved into metastasis PTC cells was revealed by monocle analysis, visualized by pseudo-time (**B**), distribution of three cell states (**C**), and distribution of 9 clusters (**D**). **E** 6 most relevant genes were identified in the evolutionary process. **F** The hallmark pathway enrichment scores of different tumor subpopulation cells were illustrated by a heat map. **G** 2 distinct cell fates of PTC cells evolved into the lymph node metastatic samples. The PFI of PTC patients with different abundant levels of C2 (**H**), C3 (**I**), and C4 (**J**) cluster, depicted by KM curves. Expression Levels of Cellular Communication Analysis (**K**) and Collagen Pathway-Related Genes (**L**) in different clusters.

As epithelial cells can undergo malignant transformation and interact with other cells to promote tumor microenvironment remodeling [18, 19], we further integrated primary PTC cells with epithelial cells from metastatic lymph node samples for multidimensional reduction and clustering into 9 cell clusters (Fig. 3A). Lymph node epithelial cells are considered metastatic tumor cells. Given the temporal heterogeneity of gene expression during metastasis, tracing the evolutionary trajectory from primary to metastatic PTC cells may reveal key biological processes. In this study, pseudo-time analysis performed on malignant cells revealed four branches and three states in the cell trajectory (Fig. 3B–D), starting with normal epithelial cells as the point of origin and progressing through primary tumor cells, ultimately culminating in the development of metastatic tumor cells.

To explore the heterogeneity of malignant cells, we evaluated each cluster utilizing Hallmark gene sets and identified six genes most relevant to the evolutionary process: *CAMK2N1*, *CD52*, *CDC42*, *ECM1*, *ID3*, and *PRDX1* (Fig. 3E). Accompanied by evolutionary trajectories, the expression of *CAMK2N1*, a crucial negative regulator of Extracellular-signal-regulated kinases (ERK), was significantly decreased. Given that our study included metastatic samples, we focused on signaling pathways associated with tumor metastasis. Clusters C2, C3, C4, and C5 were positively correlated with the EMT pathway (Fig. 3F). C2 was also associated with PI3K/AKT/mTOR signaling and NOTCH signaling pathway, which were confirmed to be associated with tumor metastasis [20, 21]. C3 showed a remarkable correlation with the Wnt/β-catenin and TGF-β signaling pathways. C4 exhibited similar characteristics to C2, with notable positive correlations in metastasis-associated signaling pathways, including the PI3K/AKT/mTOR and Wnt/β-catenin signaling pathways. Additionally, C5, apart from the positive correlation of EMT, also displayed an increase in other metastasis-related signaling pathways, such as the TGF-β pathway. These findings indicated that C2, C3, C4, and C5 may play an essential role in the metastasis of PTC.

Analysis of the tumor evolution process revealed that cells differentiated in two directions as they transitioned from normal epithelial to primary tumor cells and metastatic cells (Fig. 3G). Prior to differentiation, cells were predominantly involved in oxidative phosphorylation and ATP metabolism, indicating an enhancement of cellular metabolism. Following differentiation, genes within tumor metastasis signaling pathways were upregulated, including those regulating cell adhesion molecules and NF-κB signaling, suggesting that the cells had begun to acquire the potential for metastasis. To find out clusters with worse prognosis for PTC, we used CIBERSOFTX to infer the abundance of PTC patients in various clusters in the METABRIC dataset and combined the progression-free interval (PFI) information to perform survival analysis on different clusters. Interestingly, the results showed that only clusters 2, 3, and 4 were significantly associated with poor prognosis in PTC patients (Fig. 3H–J).

To further validate the role of the clusters, we conducted a cellular communication analysis. Clusters C2, C3, and C4 exhibited the highest probability of interaction with stromal cells, including endothelial and fibroblast cells, which are crucial for the formation of the tumor microenvironment in metastasis (Fig. 3K). Accordingly, C2, C3, and C4 may play significant roles in the metastatic process of PTC. Figure 3L illustrates the expression levels of genes related to the COLLAGEN pathway. The extracellular matrix degradation-associated gene *SDC4* was upregulated in C0, C1, C2, C3, and C4 compared to others. Based on these findings, it can be inferred that subpopulations C2, C3, and C4 are involved in the early lymph node metastasis of PTC and are correlated with poor patient prognosis. These findings reveal a high degree of heterogeneity among different clusters of PTC and offer new resources and insights for clarifying the mechanisms of PTC metastasis.

### Identification of gene co-expression modules among PTC cells

**Fig. 4:**
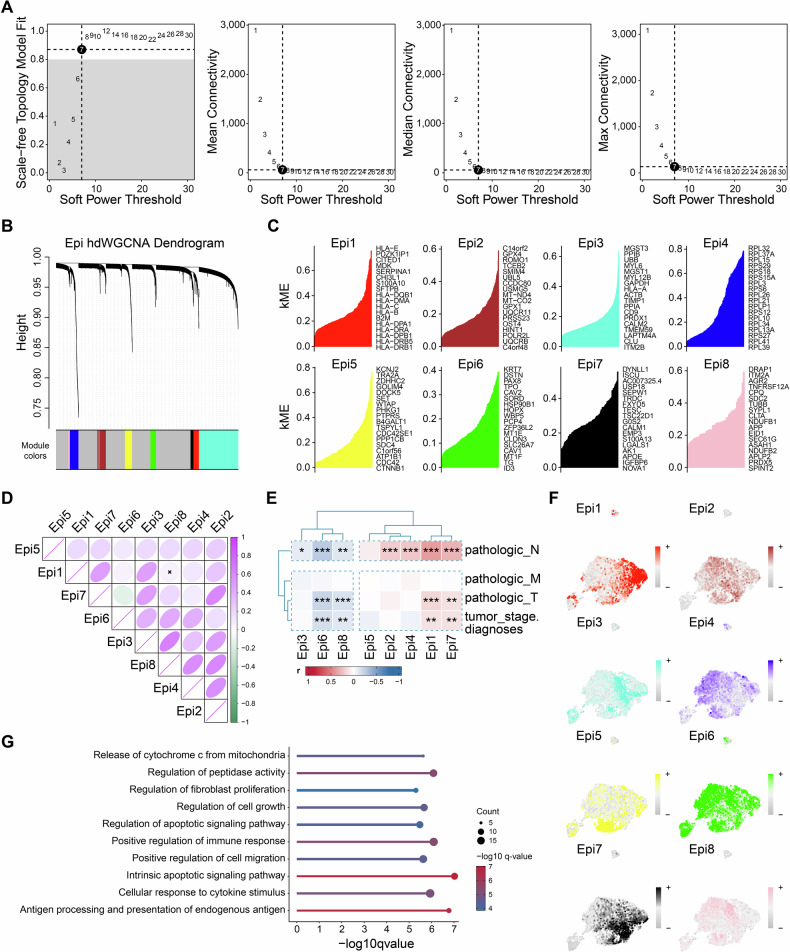
Identification of gene co-expression modules and Correlation Analysis among PTC cells. **A** Weighed gene co-expression network analysis was constructed among PTC cells. **B** The hdWGCNA dendrogram of 8 modules. **C** The eigengenes of each module, ranked by eigengene-based connectivity. **D** The correlation among 7 modules illustrated by heat map. **E** Correlation analysis of clinical features among different modules in TCGA-THCA samples. **F** UMAP scatter plots displayed the expression of modules 1–8 among all PTC cells. **G** GO Analysis of Epi1. *p < 0.05; **p < 0.01; ***p < 0.001.

To further investigate the co-expressed gene relationships that play crucial roles in clusters C2, C3, and C4, given their close correlation with poor prognosis in PTC, scale-free networks were constructed for these clusters with a soft threshold set at 7 (Fig. 4A, B). This analysis identified eight modules (Additional Table 1; Fig. 4C). As shown in Fig. 4D, F, Epi 1 and Epi 7 exhibited a certain degree of correlation, while other modules constituted another relevant group. Further analysis using ssGSEA to infer the correlation between module scores in TCGA-THCA samples and clinical characteristics revealed that Epi1 (168 genes) was significantly positively correlated with N stage and showed significant positive correlations with T stage and tumor grading (Fig. 4E). This suggests that the genes within Module 1 may be involved in lymph node metastasis of the tumor. GO analysis of the genes within Epi1 indicated a positive correlation with the regulation of cell migration (Fig. 4G). In summary, these findings imply that genes of Epi1 are part of a co-expressed network within clusters C2, C3, and C4, which is associated with lymph node metastasis in PTC.

### S100A10 is identified as a metastasis-related gene in PTC

**Fig. 5:**
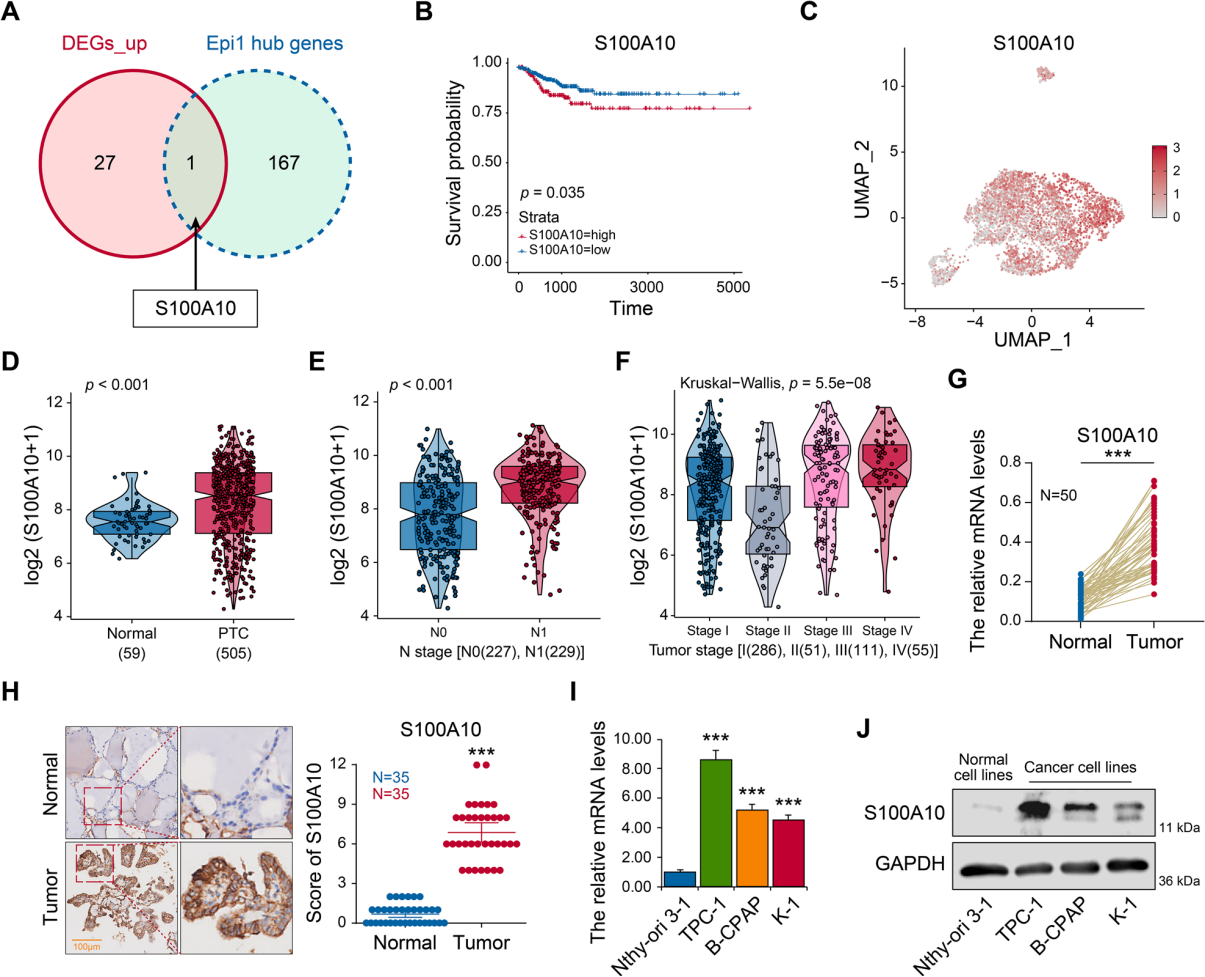
S100A10 was associated with metastasis and upregulated in PTC. **A** The intersection of genes in Epi1 and upregulated genes in metastatic PTC cells showed by Venn diagram. **B** The survival analysis of PTC patients with high- and low- S100A10 expression levels depicted by Kaplan-Meier curves. **C** tSNE scatter plots displayed the expression of S100A10 in PTC cells. **D** The expression of S100A10 in tumor and normal tissues in TCGA. **E**, **F** The relationship between tumor stage and S100A10 expression levels in PTC within the TCGA. **G** The mRNA expression levels of S100A10 in PTC tissue and corresponding normal thyroid tissue detected by RT-qPCR. **H** The protein expression level of S100A10 was detected by IHC and the scores of IHC staining. **I** The mRNA expression levels of S100A10 in normal thyroid epithelial cells and PTC cell lines detected by RT-qPCR. **J** Western blot showing the expression of S100A10 in normal thyroid epithelial cells and PTC cell lines. ***p < 0.001.

To investigate the critical genes that drive tumor metastasis, we performed a differential analysis of expression profiles between metastatic and primary tumor cells. This analysis led to the identification of 28 genes that are upregulated in metastatic tumor cells. (Additional Table 2). Considering that Epi1 is most closely associated with N stage, indicative of lymph node metastasis, we intersected the genes within Epi1 with the set of upregulated genes in metastatic tumor cells. This analysis ultimately identified the S100A10 gene (Fig. 5A). Kaplan-Meier (K-M) survival analysis revealed that the DFS values for the group with high expression levels of S100A10 were notably lower than those with low expression levels of S100A10 (Fig. 5B). Consistent with Fig. 3A, S100A10 was primarily expressed in clusters 0, 2, 3, and 4 (Fig. 5C). We further observed the expression and distribution of S100A10 in different cell types using two datasets containing PTC samples. Malignant epithelial cells were identified via infercnv-based CNV analysis (Supplementary Fig. S1A, B). Cell origin distribution analysis UMAP visualization showed clear separation of cells from PTC (cyan) and metastatic lymph nodes (red), indicating distinct cell populations between the two groups (Supplementary Fig. S1C). Differential expression of S100A10 in malignant cells S100A10 expression was visualized by UMAP, violin plot, and dot plot. Results showed that S100A10 expression was significantly higher in malignant cells from metastatic lesions than in those from primary lesions (Supplementary Fig. S1D–F).

To elucidate the expression of S100A10 in PTC, we utilized the expression profile data from TCGA. We selected 505 samples of PTC tissue and 59 samples of normal thyroid tissue. The expression of S100A10 in tumor tissue were significantly higher than those in normal thyroid tissue (Fig. 5D) and were correlated with the N stage of PTC (Fig. 5E). Furthermore, S100A10 expression varied across different stages of tumor development, with higher levels observed in Stages III and IV (Fig. 5F).

Following our initial analysis, we examined the expression of S100A10 in PTC patients. IHC was used to assess the protein expression levels of S100A10 in 35 pairs of matched tissues. Compared to normal tissues, the expression of S100A10 in PTC tissues was significantly upregulated (Fig. 5H). Concurrently, total RNA was extracted from fresh samples of 50 PTC tissues and their corresponding non-neoplastic thyroid tissues. The mRNA expression levels of S100A10 were then measured using RT-qPCR. The experimental results demonstrated that S100A10 mRNA was highly expressed in the cancerous tissues of PTC patients (Fig. 5G). Finally, RT-qPCR (Fig. 5I) and western blot (Fig. 5J) were conducted to detect S100A10 mRNA and protein levels in thyroid cell lines, including normal thyroid epithelial cells Nthy-ori3-1 and PTC cell lines TPC-1, B-CPAP, and K-1. These experiments indicated that S100A10 was increased in all PTC cell lines compared to Nthy-ori3-1 cells. Taken together, these results revealed that high expression of S100A10 was associated with PTC metastasis and worse prognosis.

### S100A10 promotes EMT by activating PI3K/AKT Pathway

**Fig. 6:**
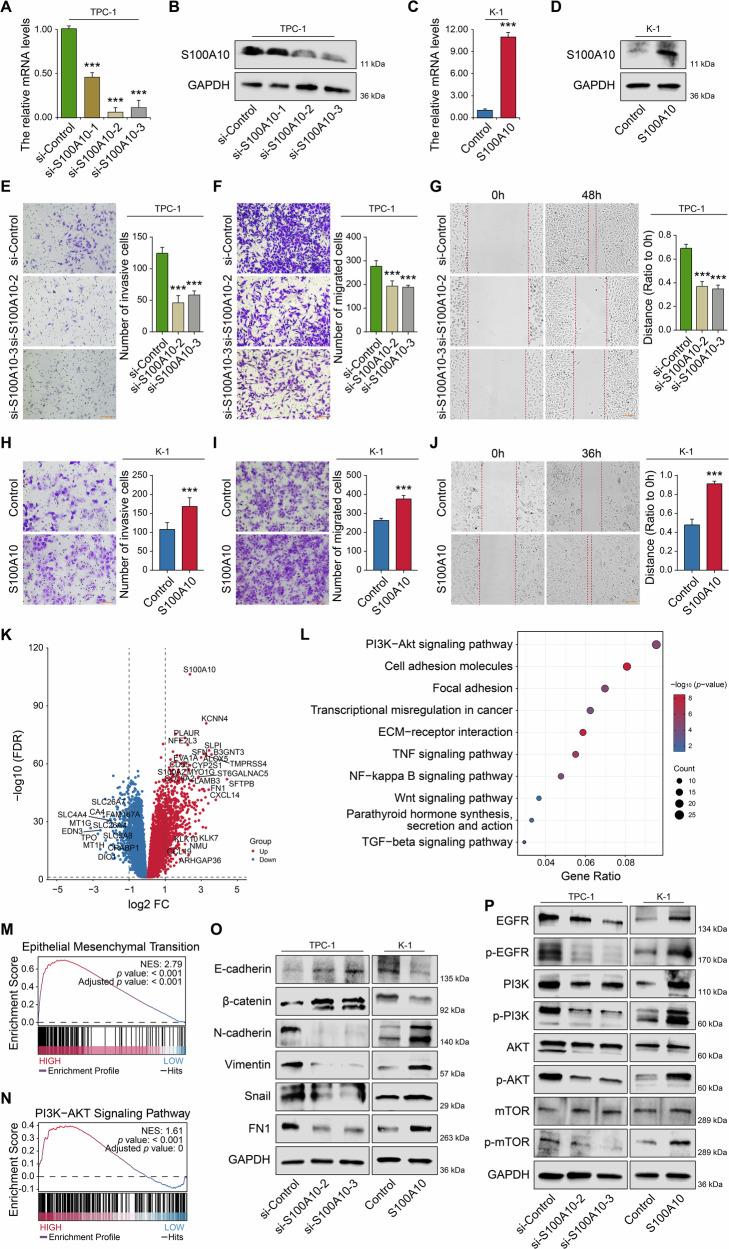
S100A10 was required for PTC cell invasion and migration and activated the PI3K/AKT pathway. RT-qPCR (**A**) and western blot (**B**) analysis of S100A10 expression levels in TPC-1-si-S100A10 cells compared with si-Control cells, respectively. Construction of K-1 cells S100A10- overexpressed was validated by RT-qPCR (**C**) and western blot (**D**). **E**, **H** Cell invasion in cells in (**B**) and (**D**) was detected by Matrigel-coated transwell analysis. Cell migration in cells as in (**B**) and (**D**) were detected by Matrigel non-coated transwell (**F**, **I**) and wound-healing (**G**, **J**) analysis. **K** Differential genes between the high- and low-PTC patients in the TCGA dataset. Enrichment analysis showing the PI3K/AKT signaling (**L**, **N**) and EMT signaling pathways (**M**) in a TCGA cohort. **O** The protein expression levels of EMT-related markers in TPC-1-si-S100A10 cells and K-1-S100A10 cells were detected by western blot, respectively, including E-cadherin, β-catenin, N-cadherin, Vimentin, Snail, and FN1. **P** Western blot shows the expression levels of proteins associated with the PI3K/AKT signaling pathway in TPC-1-si-S100A10 cells and K-1-S100A10 cells, respectively. *** *p* < 0.001.

To investigate the role of S100A10 in PTC progression, we used three specific siRNAs targeting S100A10. Two of these siRNAs effectively reduced the mRNA and protein expression of S100A10 in the PTC cell line TPC-1 (Fig. 6A, B), while S100A10 was stably overexpressed in K1 cells (Fig. 6C, D). We also constructed S100A10 stable knockdown (S100A10 short hairpin RNA [shS100A10]) cells and S100A10 stable overexpressed cells in B-CPAP cell lines (Supplementary Fig. S2A).

Using Matrigel-coated transwell assays, we found that depletion of S100A10 significantly suppressed cell invasion (Fig. 6E, Supplementary Fig. S2C), whereas overexpression of S100A10 significantly enhanced this ability (Fig. 6H, Supplementary Fig. S2B). Depletion of S100A10 also reduced cell migration in the Matrigel non-coated transwell (Fig. 6F) and wound-healing assays (Fig. 6G, Supplementary Fig. S2E). Conversely, overexpression of S100A10 enhanced these abilities (Fig. 6I, J, Supplementary Fig. S2D).

To explore the molecular mechanisms of S100A10 in PTC metastasis and invasion processes, we compared the differentially expressed genes between the high-expression and low-expression groups of S100A10 in TCGA database (Additional Table 3) (Fig. 6K) and conducted a KEGG pathway analysis for S100A10 (Fig. 6L). The results revealed that S100A10 exhibited the highest degree of association with the PI3K/AKT signaling, which is related to cancer proliferation and metastasis. Subsequently, we conducted GSEA for S100A10 in relation to both the EMT (Fig. 6M) and the PI3K/AKT signaling pathways (Fig. 6N). The results demonstrated a positive correlation between S100A10 and these two signaling pathways.

To investigate the impact of S100A10 on the expression levels of key proteins involved in the EMT in PTC cells, western blot analysis revealed that overexpression of S100A10 significantly downregulated the expression of E-cadherin and β-catenin, whereas upregulated the expression of N-cadherin, Vimentin, Snail, and FN-1. Conversely, the results of S100A10 depletion were opposite to these observations (Fig. 6O, Supplementary Fig. S2F). These findings indicated that S100A10 promotes EMT process in PTC cells and enhances their invasive and migratory capabilities. Similarly, we assessed the impact of S100A10 on the expression levels of key proteins in the PI3K/AKT/mTOR signaling pathway in PTC cells by western blot. Our results demonstrated that overexpression of S100A10 significantly upregulated the expression of EGFR, p-EGFR, p-PI3K, and p-AKT, while depletion of S100A10 yielded the opposite effect (Fig. 6P, Supplementary Fig. S2G). The experimental results confirmed that S100A10 could activate the PI3K/AKT/mTOR pathway.

To ascertain whether the activation of the PI3K/AKT pathway is crucial for the S100A10-mediated facilitation of EMT, we treated cell lines overexpressing S100A10 with LY294002 (10 μM) and observed alterations in cellular biological behaviors and EMT-related protein expression. The results indicated that the S100A10-mediated enhancement of cell invasion (Supplementary Fig. S3A, B) and migration (Supplementary Fig. S3C) was negated by the addition of LY294002. Western blot analysis revealed that compared to S100A10-overexpressed cells, cells treated with LY294002 exhibited increased expression of E-cadherin and β-catenin, while the expression of N-cadherin, Snail, and other proteins was suppressed (Supplementary Fig. S3D), indicating a significant inhibition of the EMT process. In addition, subcutaneous tumorigenesis experiments were performed in SCID mice using the B-CPAP cell line, with treatment administered via the PI3K agonist 740Y-P (Supplementary Fig. S3E). The results show that S100A10 knockdown significantly suppresses tumor growth, and this effect is partially reversed upon PI3K activation, thereby supporting the conclusion that S100A10 functions, at least in part, through the PI3K/AKT signaling axis. Based on these findings, we considered that S100A10 could regulate EMT by activating the PI3K/AKT pathway in PTC cells.

### S100A10 promotes PTC tumor growth and metastasis in vivo

**Fig. 7:**
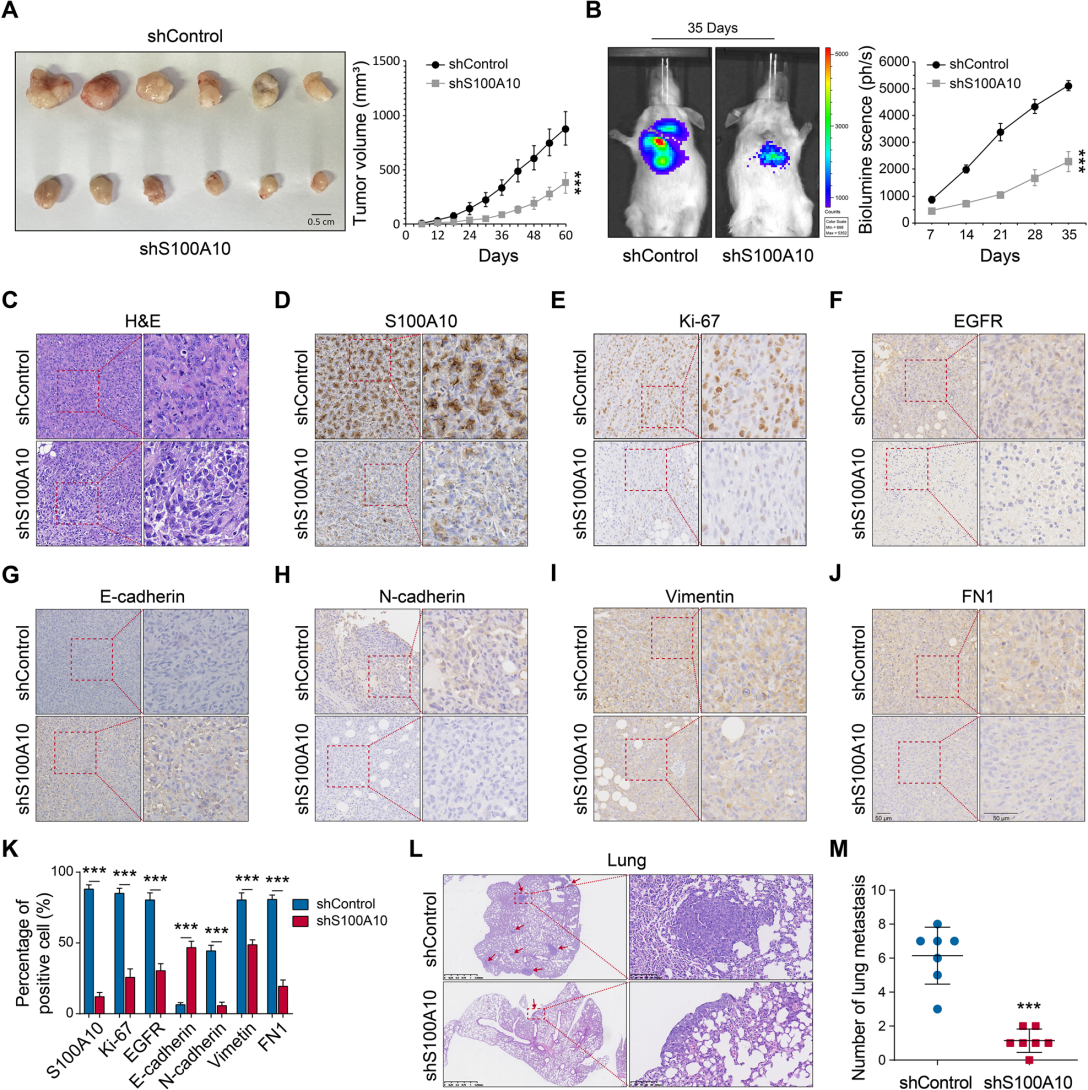
In vivo experiments confirmed that S100A10 was associated with poor prognosis in PTC. **A** Tumor growth curves of the subcutaneous tumor made of shS100A10 PTC cells and shControl cells in nude mice. The tumors were dissected and photographed at harvest time. **B** Tail vein metastasis in mice injected with PTC cells with shS100A10 cells, as well as shControl cells, determined via live imaging and quantification plot. **C**–**K** H&E staining and S100A10, Ki-67, EGFR, E-cadherin, N-cadherin, Vimentin, and FN1 protein expression levels in xenograft. **L**–**M** H&E staining and the number of pulmonary nodules in the lung metastasis model. *** *p* < 0.001.

To investigate the role of S100A10 in the development of PTC in vivo, we selected the higher efficient siRNA to establish a stable S100A10 knockdown cell line in B-CPAP cell lines. These cells were injected in subcutaneous or intravenously into SCID mice. The results showed that S100A10 promoted tumor growth (Fig. 7A) and metastasis (Fig. 7B). The live imaging data from all six mice utilized is in supplementary materials (Supplementary Fig. S2H).

Furthermore, the results of IHC staining showed that the expression levels of S100A10, Ki-67, EGFR, N-cadherin, Vimentin, and FN1 were significantly downregulated in mice with shS100A10 cells, while E-cadherin expression was upregulated (Fig. 7C–K). H&E staining also showed that the mice in the control group exhibited obvious lung metastasis, which was almost invisible in the shS100A10 group (Fig. 7L, M). These results suggest that S100A10 is involved in the tumor growth and metastasis processes of PTC and plays a positive role both in vitro and in vivo and is correlated with a worse PTC prognosis.

### S100A10 is positively correlated with the upstream regulator RAN

**Fig. 8:**
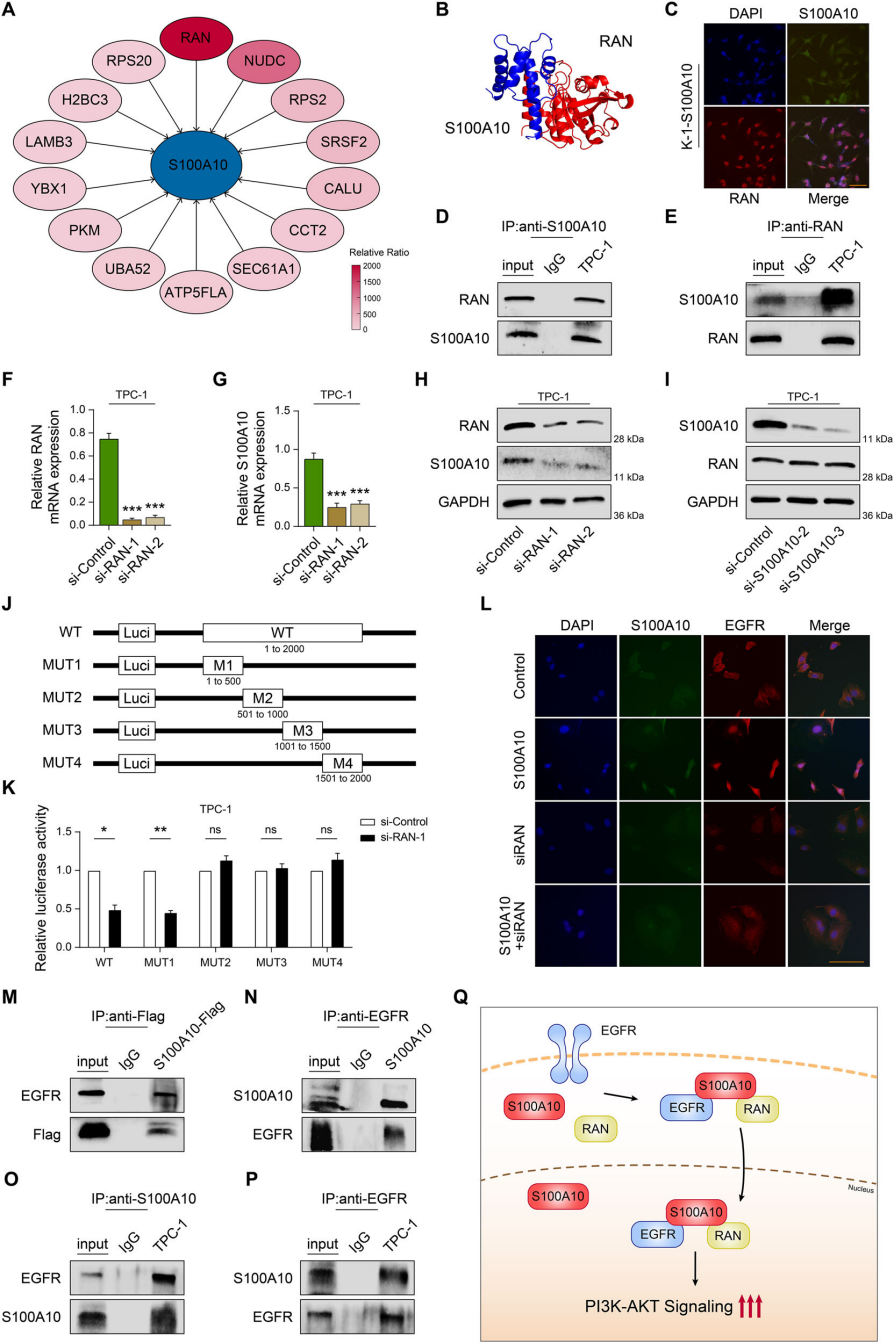
RAN facilitates the nuclear translocation of EGFR through the recruitment of S100A10. **A** The top 14 interacting genes identified by mass spectrometry. **B** The predictive protein binding model of S100A10 and RAN. **C** The intracellular colocalization of S100A10 and RAN visualized by IF in K-1-S100A10 cells. **D**, **E** The interaction of S100A10 and RAN was verified by co-IP assay. **F**, **G** RT-qPCR analysis of S100A10 mRNA expression after RAN knockdown. **H**, **I** Western blot demonstrated that RAN influences the expression of S100A10. **J** S100A10 promoter region truncated mutant plasmid schematic diagram. **K** The luciferase activity of different reporters that transfected into TPC-1 cell lines. **L** IF shows the relative position of the target proteins in the cell. Co-IP assay shows the interaction of S100A10 and EGFR, including exogenous (**M**, **N**) and endogenous (**O**, **P**). **Q** A model of the role of S100A10 in PTC. ***p < 0.001.

To further elucidate the mechanism of S100A10 function, immunoprecipitation followed by mass spectrometry was performed to identify its interacting proteins. This analysis revealed a total of 263 S100A10 binding proteins, with RAN emerging as the top candidate due to its established role in EMT (Fig. 8A). Next, the protein-protein interaction between S100A10 and RAN was analyzed using GRAMM-X (Protein-Protein Docking Web Server v.1.2.0), PDBePISA (https://www.ebi.ac.uk/), and PyMOL2 software (v.2.5). The predicted binding model of S100A10 and RAN is presented as a cartoon structure in Fig. 8B.

The co-localization of S100A10 and RAN in PTC cells was detected using immunofluorescence (Fig. 8C). Additionally, the interaction between S100A10 and RAN was confirmed by co-immunoprecipitation (Fig. 8D, E). These findings showed that S100A10 could interact with RAN and colocalize within the PTC cells.

To investigate the interaction between S100A10 and RAN, we utilized siRNA targeted against RAN to reduce its expression in the TPC-1 cells (Fig. 8F, Supplementary Fig. S4A, B). The results of RT-qPCR and Western blot indicated that the depletion of RAN led to a decrease in the mRNA and protein expression levels of S100A10 in TPC-1 cells (Fig. 8G, H). However, the downregulation of S100A10 expression did not affect the expression levels of RAN (Fig. 8I). Notably, neither RAN nor S100A10 has been previously reported to function as a transcription factor in the existing literature. To further investigate whether RAN influences S100A10 at the transcriptional level, a series of Truncated mutation plasmids were generated by truncating the S100A10 promoter region into four sequential fragments: 1 to 500, 501 to 1000, 1001 to 1500, and 1501 to 2000 bp relative to the transcription start site (Fig. 8J). Dual-luciferase reporter assays demonstrated that RAN knockdown significantly at tenuated luciferase activity driven by the 1 to 500 bp promoter fragment, whereas no significant changes were observed in the other Truncated mutation plasmid (Fig. 8K). In conclusion, these results demonstrate that RAN binds to S100A10 within cells and, at least in part by modulating the transcriptional activity of the S100A10 promoter region, regulates S100A10 expression.

### RAN-S100A10 complex promotes PTC progression by increasing EGFR nuclear localization

As we previously confirmed the regulatory role of S100A10 in the PI3K signaling pathway and observed a close regulatory association between RAN and S100A10, we further investigated the correlations of both RAN and S100A10 with the PI3K pathway and its key upstream regulator, EGFR. Co-immunoprecipitation assays demonstrated that S100A10 bound to EGFR, both exogenously (Fig. 8M, N) and endogenously (Fig. 8O, P).

Furthermore, we investigated the impact of S100A10 and RAN on EGFR using immunofluorescence in PTC cells. The results indicate that in cells with overexpression of S100A10, the nuclear localization of EGFR is increased. Conversely, upon depletion of RAN, the nuclear localization of EGFR is decreased (Fig. 8L).

To conduct a more in-depth study on the role of RAN in the development of PTC, we transferred the S100A10 plasmid into the TPC-1 cell line with RAN depletion for rescue experiments. Transwell (Supplementary Fig. S4C) and wound-healing (Supplementary Fig. S4D) assays demonstrated that the migration and invasion ability of PTC decreased significantly after depletion of RAN, whereas S100A10 overexpression completely abrogated these inhibitory effects. Western blot analysis revealed that RAN acted as a suppressor of EMT (Supplementary Fig. S4E) and inhibited PI3K-AKT signaling (Supplementary Fig. S4F), which were substantially reversed by S100A10 overexpression. Collectively, these data establish that RAN promotes EMT progression and PI3K-AKT activation in PTC cells primarily through S100A10-dependent mechanisms.

Based on the experimental results, we considered that these three proteins interact within the cell to exert their effects, activating the PI3K/AKT/mTOR signaling pathway, promoting EMT in tumors, and ultimately facilitating the metastasis of PTC (Fig. 8Q).

To identify potential therapeutic agents targeting S100A10, we conducted a correlation analysis of S100A10 expression with the IC_50_ values of 767 potential drugs using the CellMiner database (https://discover.nci.nih.gov/cellminer/home.do). As shown in Supplementary Fig. S7, seventeen candidate drugs displayed a significant negative correlation with S100A10 expression (Pearson correlation coefficients < −0.5). This suggests that these drugs may have the potential for targeting S100A10 in therapeutic applications. Overall, these findings provide strong theoretical support for further preclinical research targeting S100A10.

## Discussion

It is widely acknowledged that papillary thyroid carcinoma is prone to lymph node metastasis, which is an independent risk factor for poor prognosis in PTC patients. Statistics have shown that PTC patients with lymph node metastasis have a worse prognosis. PTC is a highly heterogeneous disease. Investigating the lineage evolution from primary to metastatic PTC cells can help identify key tumor subpopulations and genes that promote PTC metastasis. Although several studies have reported the effects of S100A10 on the invasion and metastasis of PTC—for instance, Martin Nipp et al. identified elevated S100A10 expression in PTC through proteomic analysis [22], and Yasuhiro Ito et al. demonstrated its role in promoting invasive characteristics of thyroid intermediate atypical carcinomas via immunohistochemical investigations [23]—none of these studies employed single-cell sequencing or other advanced technologies for systematic screening, nor did they provide a comprehensive analysis of the underlying carcinogenic mechanisms of S100A10.To our knowledge, this study is the first to elucidate the molecular mechanisms by which S100A10 promotes PTC metastasis.

Herein, we identified nine tumor subpopulations with distinct characteristics in PTC, among which C2, C3, and C4 demonstrated similar characteristics in EMT pathway. Subpopulations C2 and C4 were associated with cell metastasis signaling pathways such as PI3K/AKT/mTOR signaling pathway. Subpopulation C3 was significantly correlated with the Wnt/β-catenin signaling pathway, a known biomarker and target for cancer therapy [24]. Furthermore, our analysis indicates that C2, C3, and C4 are associated with a poor prognosis. These results suggest that these three subpopulations may significantly contribute to the progression of thyroid cancer. Previous research has shown that collagen can affect the tumor microenvironment, leading to the proliferation, migration, and metastasis of cancer cells, thereby facilitating cancer development [25]. Interestingly, cellular communication analysis has revealed that within the collagen pathway, C2, C3, and C4 have a stronger association with fibroblasts than other subpopulations. Activated fibroblasts in cancer can directly influence various aspects of cancer cell biology, such as migration, proliferation, metabolism, and drug resistance, and are considered tumor promoters [26]. Additionally, we found that Epi1 and Epi7 within C2, C3, and C4 are closely associated with the tumor N stage. These findings indicate that the C2, C3, and C4 subpopulations we have identified are strongly correlated with the promotion of tumor progression and increased migration of PTC cells. Moreover, we have determined that S100A10 is the core gene within these subpopulations.

Upregulated S100A10 has been associated with poor prognosis in various types of cancer. Overexpression of S100A10 has been linked to unfavorable outcomes in cancers such as gallbladder cancer, ovarian cancer, and pancreatic ductal adenocarcinoma [27–29]. In lung squamous cell carcinoma, S100A10 has been found to be positively immunostained on the membrane [30]. Similarly, in colorectal cancer, increased expression of S100A10 is correlated with poor prognosis [31, 32]. However, research on S100A10 in papillary thyroid carcinoma is less extensive. In this experiment, we conducted experiments in cell lines with overexpression of S100A10 and in those with S100A10 depletion in PTC. Functional assays validated that S100A10 promotes the migratory and invasive behavior of PTC cells. Animal experiments also confirmed this phenomenon. Concurrently, we examined the expression of EMT-related proteins E-cadherin and β-catenin, as well as the levels of N-cadherin, Vimentin, Snail, and FN-1 at the molecular level. The results indicated that high expression of S100A10 could facilitate EMT in PTC cells, while low expression inhibited this process.

Recent studies have demonstrated that S100A10 promotes the proliferation of gastric cancer, hepatocellular carcinoma and osteosarcoma cells through the mTOR signaling pathway and inhibits apoptosis by enhancing aerobic glycolysis [33]. Additionally, studies have found that S100A10 promoted pancreatic cancer cell proliferation, migration, adhesion, and in vivo tumor growth by regulating JNK pathway [16]. In our study, we have identified a novel regulatory mechanism for S100A10, which exerts its effects on PTC cell lines through the PI3K/AKT pathway. Our results demonstrated that overexpression of S100A10 significantly upregulated the expression of EGFR, p-PI3K, and p-AKT, while S100A10 depletion yielded contrasting outcomes.

RAN is a Ras-related GTPase involved in nucleocytoplasmic transport, cell cycle regulation, and cellular transformation [34]. The essential roles of RAN were reported in some tumors [35, 36]. In this experiment, both mass spectrometry and co-immunoprecipitation confirmed the binding of RAN to S100A10 within PTC cells. Furthermore, based on western blot and qPCR results, it is considered that RAN acts as an upstream regulator of S100A10 and can regulate its expression. Our luciferase reporter assay demonstrated that RAN influences the transcriptional activity of the S100A10 1 to 500 bp promoter fragment. This finding aligns with the research report by R. Pawliczak et al. on the effect of transcription factor AP-1 on S100A10 [37]. Furthermore, existing studies have indicated that RAN indirectly affects the nuclear import of transcription factors such as AP-1 by regulating the function of the nuclear pore complex [38]. Our study is the first to link the regulatory function of RAN to the transcriptional activity of S100A10 and provides preliminary evidence for the involvement of a “RAN-AP-1” pathway. Interestingly, immunofluorescence results revealed that RAN can influence the proportion of EGFR in the cell nucleus, and depletion of RAN led to a reduction in the nuclear translocation of EGFR.On this basis, the rescue experiment confirms that RAN promotes EMT and activates the PI3K/AKT pathway via S100A10. This represents a promising avenue for future research.

This study has some limitations that should be acknowledged. Notably, the three subpopulations we identified in PTC require further validation. In future studies, we will collect clinical samples and utilize multiplex immunofluorescence to confirm the presence of these subpopulations and investigate whether S100A10 can serve as a biomarker for PTC metastasis. Furthermore, future research in our group will investigate whether RAN and S100A10 enter the cell nucleus as a complex and elucidate the mechanism of EGFR nuclear entry. Beyond these specific questions, the detailed hierarchical regulatory mechanisms between RAN and S100A10 remain to be elucidated, representing a promising direction for future research.

## Conclusions

In summary, we identified S100A10 as an upregulated protein in metastatic PTC through integrative analysis. Functional assays demonstrated that S100A10 promotes PTC metastasis via the PI3K/AKT pathway, with RAN driving progression through S100A10-dependent mechanisms. These findings uncover a potential molecular mechanism underlying PTC progression and suggest S100A10 as a promising therapeutic target for metastatic PTC.

## Supplementary information

Supplementary Figures and Methods

Checklist

Additional table

## Data Availability

Datasets related to this article are from public database (GSE184362, GSE158291, GSE241184) and TCGA database (https://portal.gdc.cancer.gov/). All data generated or analyzed during this study are included in this article/Additional files.
