# Supplementary material for: RAN-S100A10-EGFR axis facilitates papillary thyroid cancer metastasis by PI3K/AKT signaling
Source: Cell Death Dis. 2026 Apr 16;17(1):510. doi: 10.1038/s41419-026-08649-6 (PMC13201611; doi:10.1038/s41419-026-08649-6)
Supplement: Supplementary file 1 — Supplementary Figures and Methods [file 41419_2026_8649_MOESM1_ESM.docx]

**Table S1. Oligonucleotides of siRNAs**

| siS100A10-1 | GCCTCACCATTGCATGCAA |
| --- | --- |
| siS100A10-2 | GGATAAAGGCTACTTAACA |
| siS100A10-3 | CCATGATGTTTACATTTCA |
| siRAN-1 | GCAACAAAGTGGATATTAA |
| siRAN-2 | CTACGACATTTCTGCCAAA |
| siRAN-3 | GTGCCATCATAATGTTTGA |

**Table S2. Oligonucleotides used for RT-qPCR**

| **Name** | **Sequence (5’ to 3’)** |
| --- | --- |
| S100A10 up  S100A10 low | GGCTACTTAACAAAGGAGGACC  GAGGCCCGCAATTAGGGAAA |
| RAN up  RAN low | GGTGGTACTGGAAAAACGACC  CCCAAGGTGGCTACATACTTCT |
| GAPDH up  GAPDH low | CAAGGTCATCCATGACAACTTTG  GTCCACCACCCTGTTGCTGTAG |

**Table S3. Antibodies used for study**

| **Name** | **Source** | **Catalog** |
| --- | --- | --- |
| E-Cadherin (24E10) Rabbit mAb | Cell Signaling Technology | 3195 |
| N-Cadherin (D4R1H) XP® Rabbit mAb | Cell Signaling Technology | 13116 |
| S100A10 Polyclonal antibody | Proteintech | 11250-1-AP |
| Beta Catenin Polyclonal antibody | Proteintech | 17565-1-AP |
| Vimentin (D21H3) XP® Rabbit mAb | Cell Signaling Technology | 5741 |
| Snail (C15D3) Rabbit mAb | Cell Signaling Technology | 3879 |
| Fibronectin Polyclonal antibody | Proteintech | 15613-1-AP |
| EGFR-Specific Polyclonal antibody | Proteintech | 18986-1-AP |
| PI3 Kinase p110 alpha Rabbit mAb | Abclona | A22730 |
| Phospho-PI3 Kinase p85 (Tyr458)/p55 (Tyr199) (E3U1H) Rabbit mAb | Cell Signaling Technology | 17366 |
| AKT1 Rabbit mAb | Abclona | A17909 |
| Phospho-Akt (Ser473) (D9E) XP® Rabbit mAb | Cell Signaling Technology | 4060 |
| mTOR Monoclonal antibody | Proteintech | 66888-1-Ig |
| Phospho-mTOR (Ser2448) Monoclonal antibody | Proteintech | 67778-1-Ig |
| Ran Antibody (A-7) | Santa Cruz | sc-271376 |
| Phospho-EGFR (Tyr1069) Polyclonal antibody | Proteintech | 30277-1-AP |
| GAPDH Monoclonal antibody | Proteintech | 60004-1-Ig |

**Table S4. Truncated mutation plasmids of S100A10 promoter region**

| **Truncated mutation** | **Sequence** |
| --- | --- |
| MUT1 | GGTACCTTTATAATCAGTATGATCACAATTATGCAAATATTTGTTACATATGCATAAAAAGACTAGAAATATGAAAATGTTTATCTTTGGCTCATGTATTTATAAATCATTTTTTATACATTGATATATTTTCCAAGCCTTCTAAGAATGGAATCTTTGTACAGATTAAAAAGTTACAATAAGTGTTATTGTTGTTTTTAAATTTCTACCCAATGTTGCTTCCTGCAGAAATTATTCTGTGACCACTCCAAGAGGACTTGGAAGCCCCTCCTCAGTGTTCTCCTAGTATCCAATAGTATCCTCTACCATTCACTAAGCACACTGGATTTTAATTGTTTGCTTATCTAACGCCCAATACTTGGAACTCTTCAGAGAAAGAGACTGATTCTCTAATCCCTGAATTCCCAGGCCTACCACATACACTAAAGCACTCCTAAGGTCTGTGGACCAGATGAAGAAATCAGATGAGTAGGGGTACGCAAACCCAGGCATTCCCATTCTAAACCGCTAGC |
| MUT2 | GGTACCCAGTCGCATTGCAATCCCTGCAACAAAAGGGATCCGAGATTTCCTCCACTGGTGACTGTGACCTGGGGACTGGGAAGTACTGTATACAGGAAGATCTTGAAGAGCTGTCCCCTTGAATAGTCAGCACCAGGGTTGCACGGAAAGTAATAGCTGAAATCCAAGTTGGGTTTTCCTGGCAACAGCCAATATTAGTCAGAGGTGGCACTTGGAGGATTCCCCAGGTCTGCTCATTCCTTTAGGATCATTCATTCCTCCCAAGGCCTCCCTTGGGAACAAAGGAAAACCCCAGAGTTTGGGATTGGGCTCTTTTCCACCAACAGCTGCTAGTAGTTTGTATTTAACCCTGAGTCACAATTAAAATAAAAGAGGGCGGGCGGGGGAGGGGCGAGGAGTTGGTAAGCATCCCCTAGGAAACACTTAGGTTTTCTCTAAATTTATTCCAGAAAATTCTTCTAAAGGACTTTTCAAGGACCACAGCATACTGCCTTGGAAACTTAGTTGCTAGC |
| MUT3 | GGTACCTTTAACCTTTTTCTTTTCTTTTTTTCTTTTTTCTTTTTTTTTTTTTTTTTTGAGACAGAGTCTCACTCTGTCACCCAGGCTGGAGTGCAGTGGTGCGATCTCGGCTCACTGCAACCTCTGTCTCCCAGGTTCAAGCGATTCTCATGCCTCAGTCTCCTGGGATTACAGGCGCGCATCACCACTCCCGGCTAATTTTTGTATCAGTAGAGACAAGGTTTCACCATGTTGGCTAAGCTGGTGTTGAACTCCTGACCTGAGGTGATCCGCCCGCCTCGGCCTCCCAAAGTGTTGGGATTACAGGCGTGAGCCACCGCGCCCGGCCAGTTTTTAACACTATTAGCCACACTGAAACTGAACTATTGATCAAGTGACGCCACACAAAGGGGTAAATCCCCTGTTCAACAAAGGGTTTGTGACGCCCCTGGGTGCTGACAAGCCAAACCGCACCCTCCCTGCGGCACCTCGCGGGCCGGTGGGGCGGGAAGCCCGGCTTCTGGGGAGCTAGC |
| MUT4 | GGTACCGGTGCCGCCCCTCCACTGGCGCAGGCCGCCGAGACCCCCAGACGGACCTCCTAGGGCTAATCTGATAGTGCCTCTGAGGTCGATAGGACTCCACGTGCCACTCCCTGCAGGGTCATCCAGCAAGTAATTCCTAGACCCGTAGGTGGCCGCAGAGCCGGTTACCTCTGGTTCTGCGCCAGCGTGCCCCACCCGCAGGACGGCCGGGTTCTTTGATTTGTACACTTTCTAAAACCAAACCCGAGAGGAAGGGCAGGCTCAGGGTGGGGATGCCCTGAAATATTCGAGAGCAGGACCGTTTCTACTGAAGAGAAGTTTACAAGAACGCTCTGTCTGGGGCGGGCGAGGCCTCTGCGAGGCGGGTCCGGGAGCGAGGGCAGGGCGTGGGCCGCGCGCCCGGGGTCGGGGGAGTCGGGGGCAGGAAGAGGGGGAGGAGACAGGGCTGGGGGAGCGCCCTGCCGAGCGCCCGCCAGGCTCCTCCCGCTCCCGCGCCGCCTCCCTCTGCTAGC |


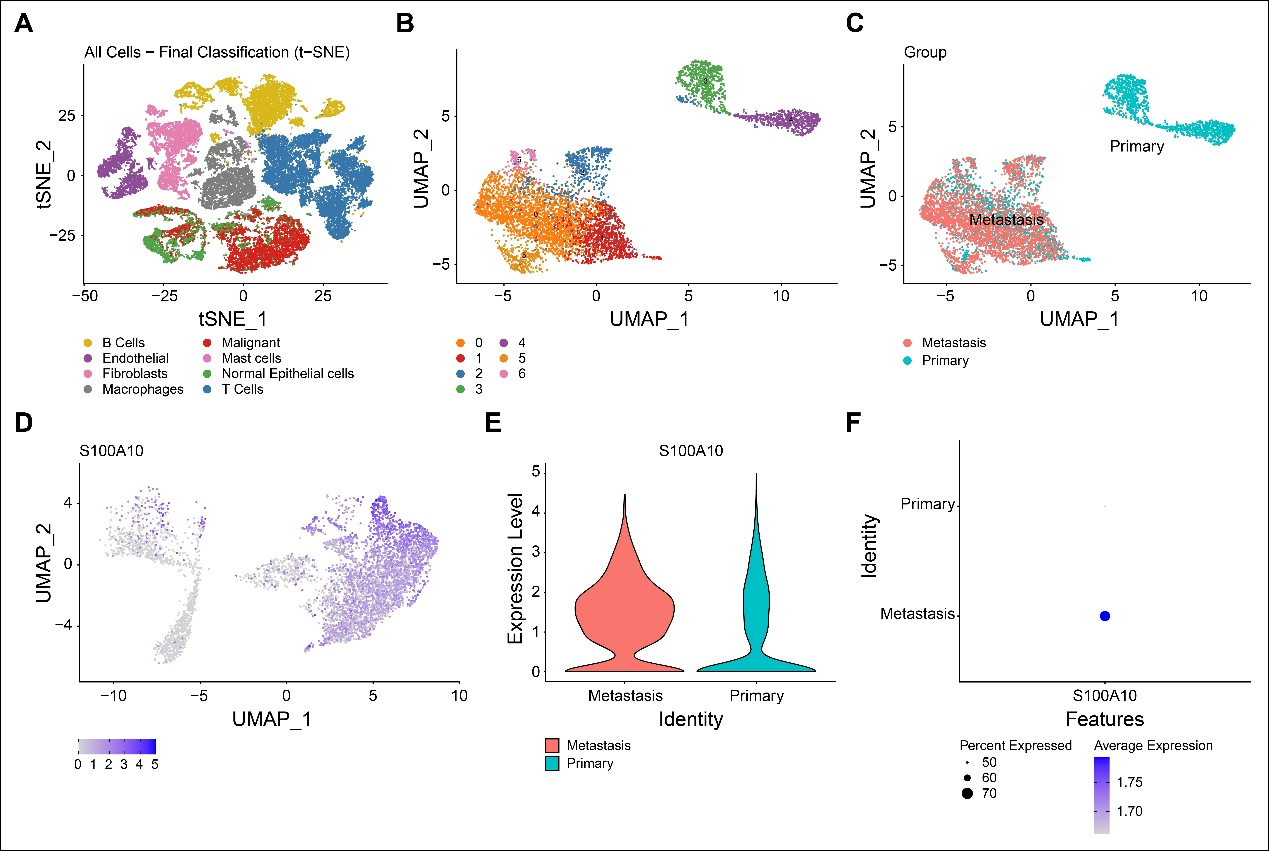


**Figure S1 Single-cell transcriptomic profiling of primary thyroid tumors and metastatic lymph nodes**

**(A)** t-SNE plot of all cells after Seurat preprocessing, batch correction, Louvain clustering, and infercnv-based CNV analysis. **(B)** UMAP plot generated from the top 30 principal components; colors correspond to cluster IDs assigned by the Louvain algorithm. **(C)** UMAP plot distinguishing cell origins. **(D)** UMAP plot showing S100A10 expression levels; darker colors indicate higher gene expression. **(E)** Violin plot of S100A10 expression distribution in malignant cells from primary and metastatic lesions. **(F)** Dot plot of S100A10 expression features; black dot size is percentage of expressing cells, blue dot color intensity is average expression level.


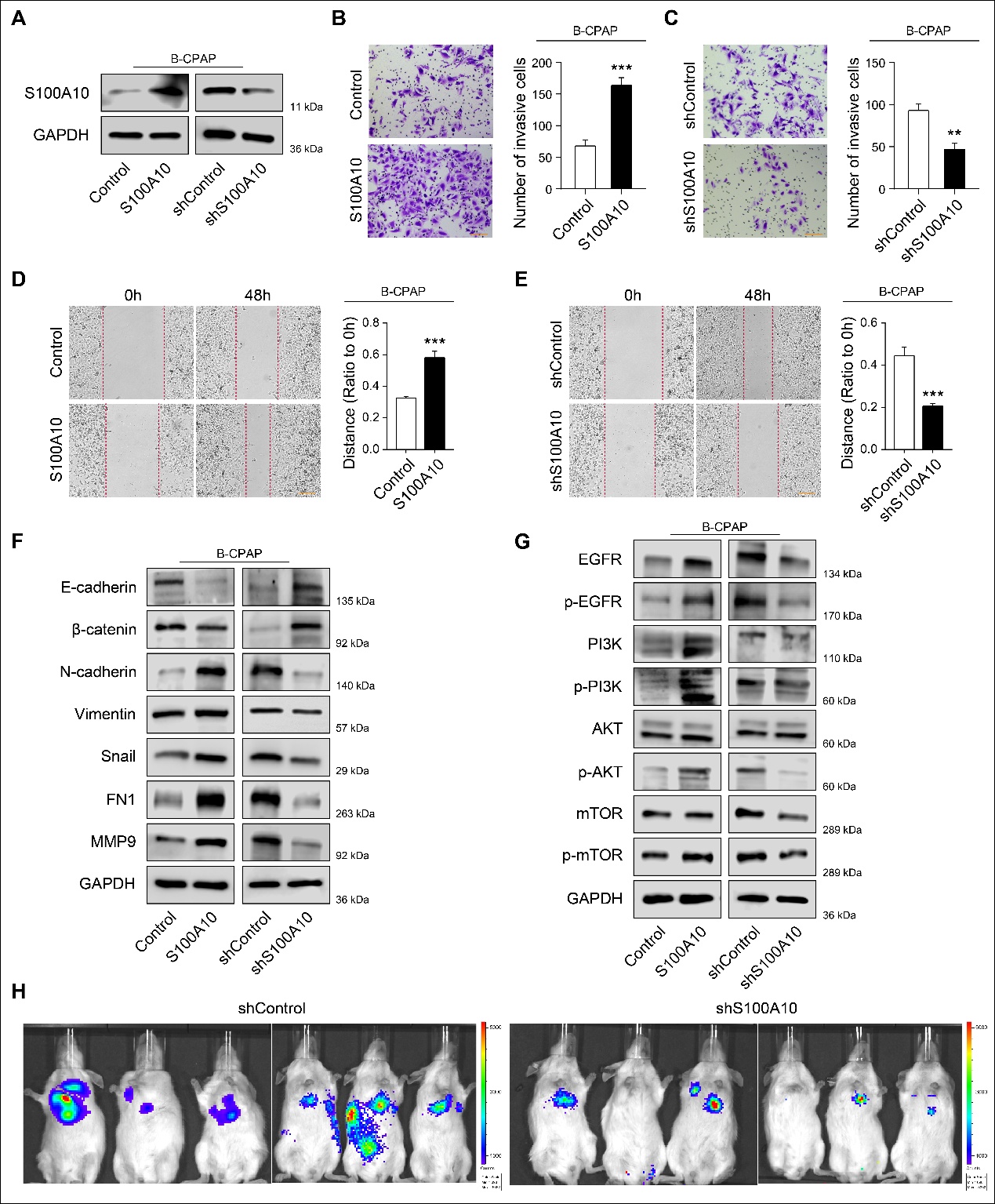


**Figure S2 S100A10 regulates EMT and PI3K/AKT signaling pathway in B-CPAP cells.**

**(A)** S100A10 overexpression cell line and shS100A10 cell line were constructed in BCPAP cell line. **(B-E)** Transwell and wound-healing assays in B-CPAP cells following S100A10 overexpression or shS100A10, respectively. **(F)** Western blot analysis of EMT-related proteins in control, S100A10-OE and shS100A10 cells. **(G)** Western blot analysis of PI3K/AKT Pathway in control, S100A10-OE and shS100A10 cells. **(H)** The live imaging data of all six mice. ***p* < 0.01; ****p* < 0.001


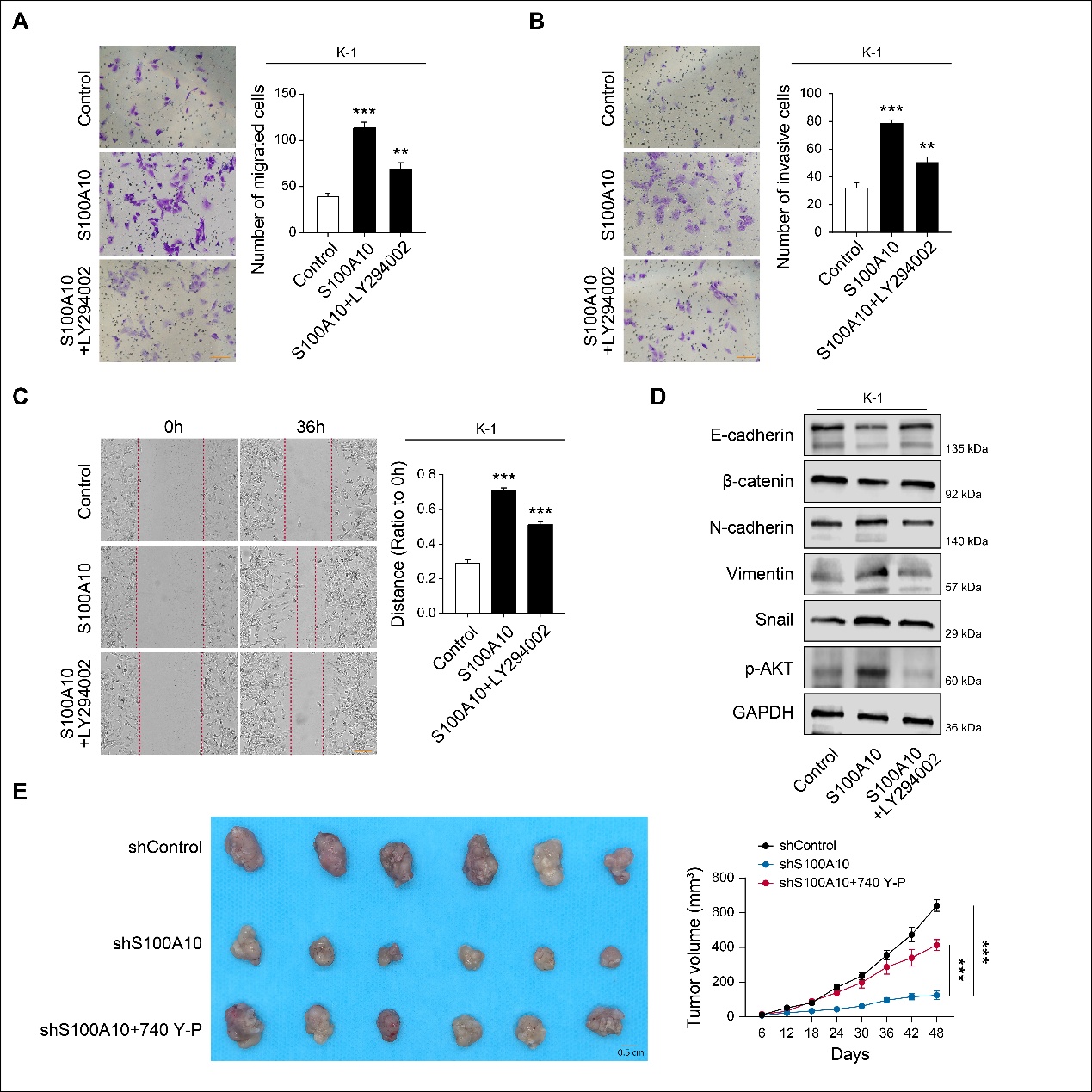


**Figure S3 S100A10 promotes EMT in PTC cells via PI3K/AKT signaling pathway.**

Representative data from transwell migration **(A)** and matrigel invasion assays **(B)** performed in the indicated cells treated with or without LY294002. **(C)** Representative data from wound-healing migration assays performed in the indicated cells treated with or without LY294002. **(D)** Relative expression levels of E-cadherin, β-catenin, N-cadherin, Vimentin, Snail, p-AKT and GAPDH in the indicated cells treated with or without LY294002. **(E)** Tumor growth curves of SCID mice treated with 740Y-P and subcutaneously injected with B-CPAP cells. The tumors were dissected and photographed at harvest time. **p* < 0.05; ***p* < 0.01; ****p* < 0.001


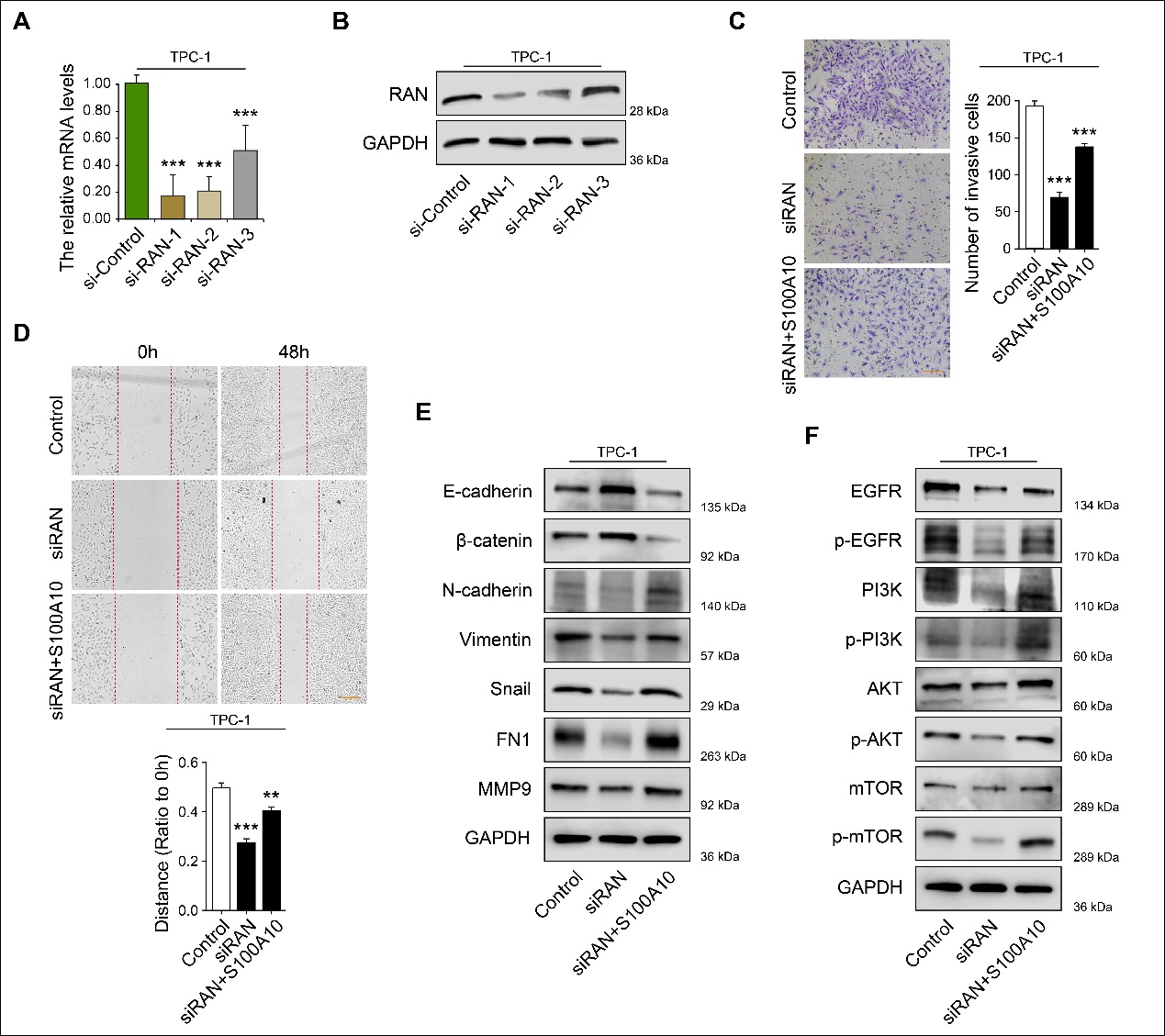


**Figure S4 S100A10 rescues the inhibition of EMT and the PI3K**/**AKT pathway caused by RAN deficiency in PTC cells.**

**(A-B)** RT‑qPCR and western blot showing the efficiency of S100A10-depleted TPC-1cells with siRNA. **(C-D)** Transwell and wound-healing assays in TPC-1 cells with control, siRAN and siRAN+S100A10 treated. **(E-F)** WB analysis of EMT-related proteins and PI3K/AKT Pathway expression in TPC-1 cells with control, siRAN and siRAN+S100A10 treated. ***p* < 0.01; ****p* < 0.001


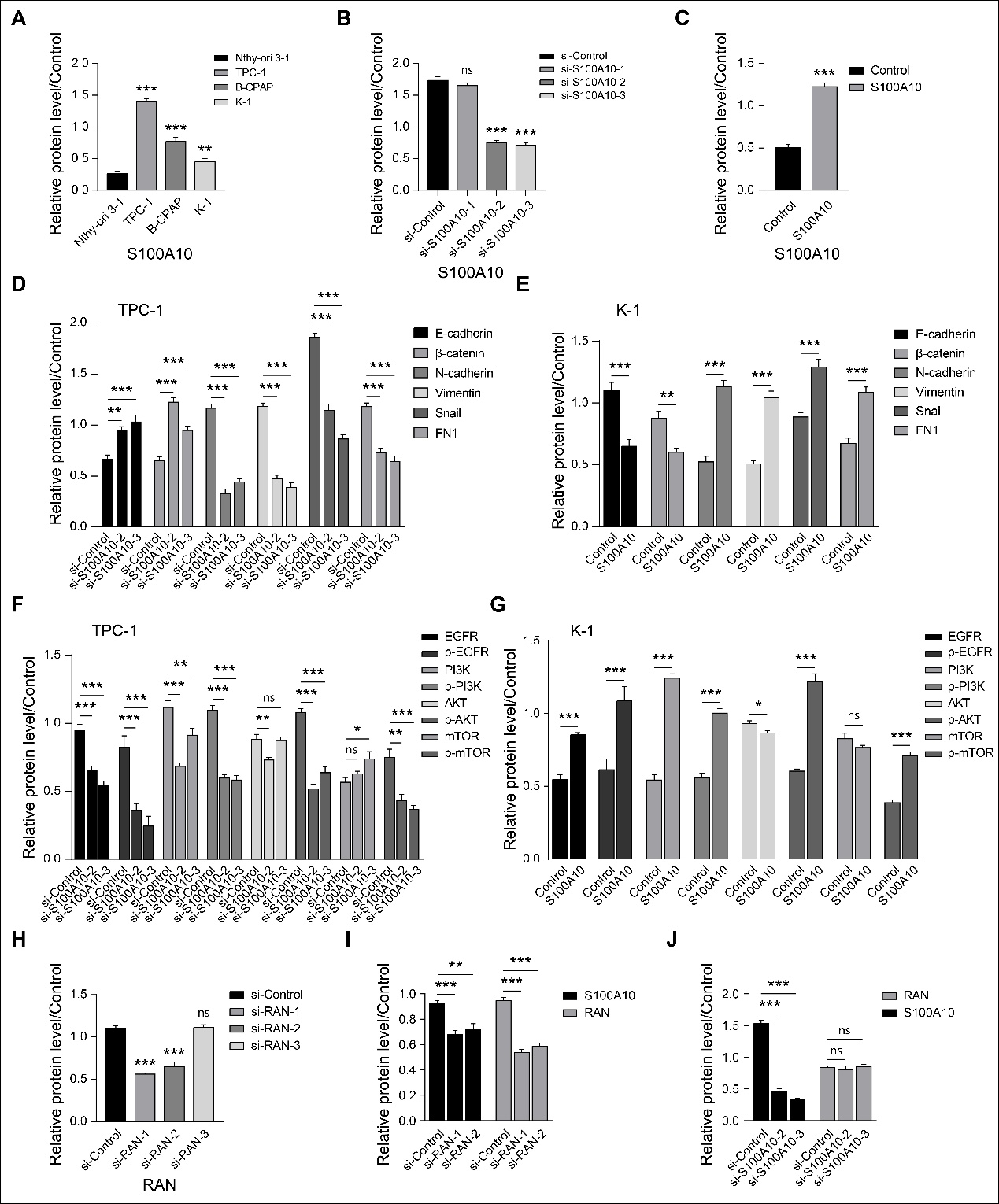


**Figure S5 The statistical analysis of immunoblots.**

The scale values of each single stripe were measured by Image J software and normalized by GAPDH, including **(A)** Figure 5J, **(B)** Figure 6B, **(C)** Figure 6D, **(D-E)** Figure 6O, **(F-G)** Figure 6P, **(H)** Figure S4B, **(I)** Figure 8H, **(J)** Figure 8I. **p* < 0.05; ***p* < 0.01; ****p* < 0.001


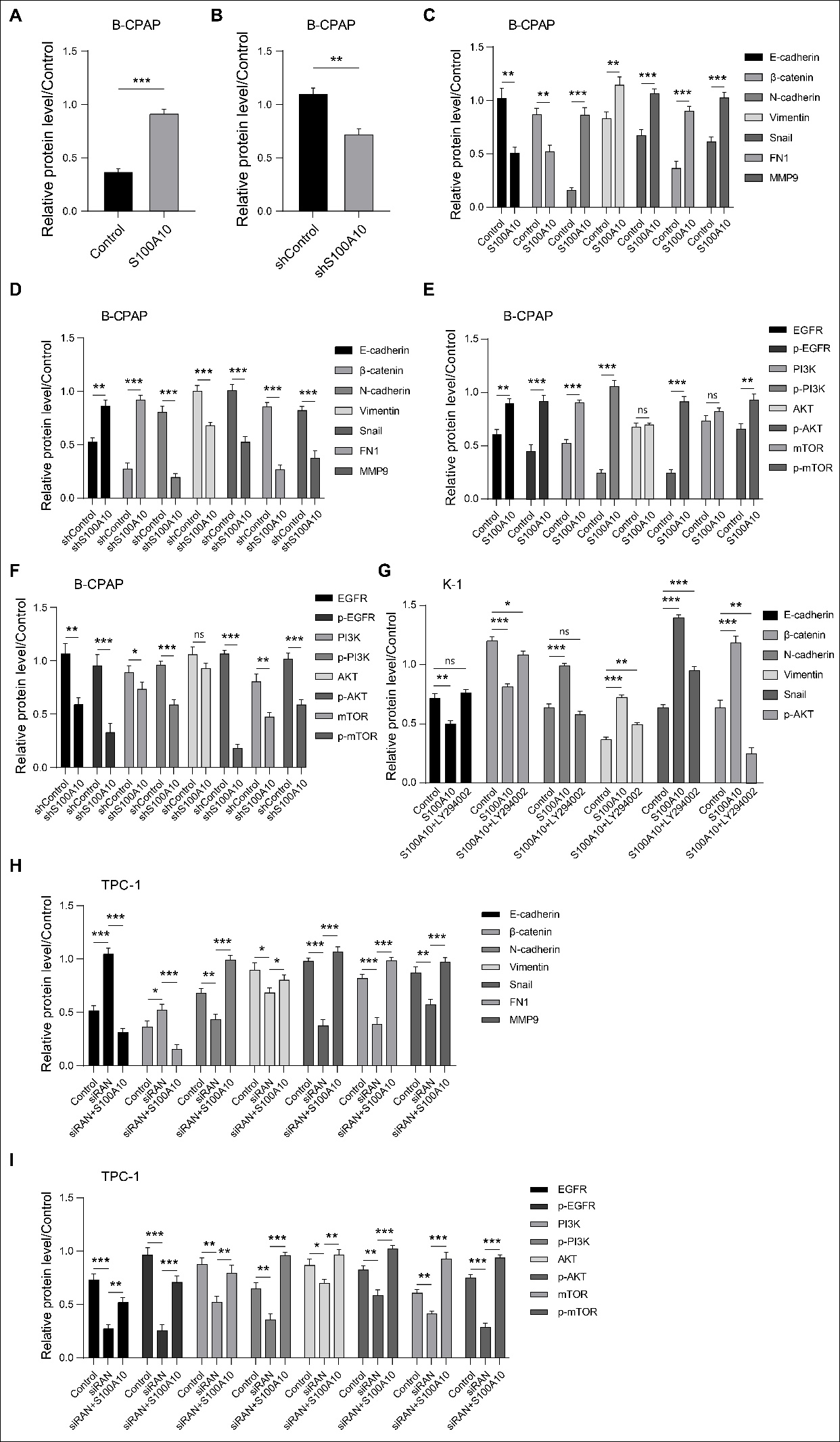


**Figure S6 The statistical analysis of immunoblots.**

The scale values of each single stripe were measured by Image J software and normalized by GAPDH, including **(A-B)** Figure S2A, **(C-D)** Figure S2F, **(E-F)** Figure S2G, **(G)** Figure S3D, **(H)** Figure S4E, **(I)** Figure S4F. **p* < 0.05; ***p* < 0.01; ****p* < 0.001


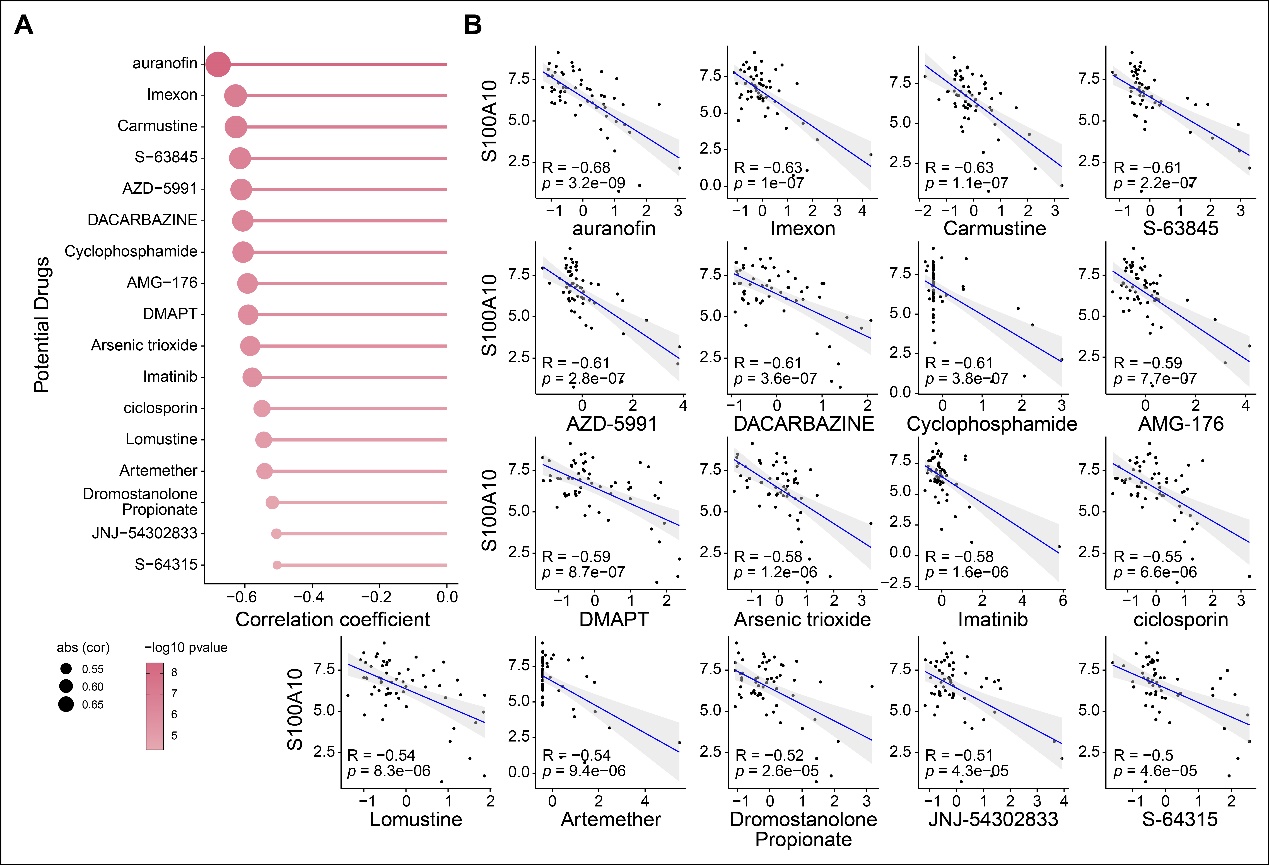


**Figure S7 Analysis of Potential Drugs Targeting S100A10.**

**(A)** Bar diagram showing the candidate 17 potential drugs for high S100A10 PTC patients. **(B)** IC50 values of 17 potential drugs.

**Materials and methods**

**1. Data Collection**

Single-cell RNA sequencing data (sc RNA-seq) was downloaded from public databases. The expression matrix and clinical information of single-cell RNA sequencing gene expression data (GSE184362, GSE158291, GSE241184) were downloaded from the Gene Expression Omnibus (GEO) database. Additionally, the TCGA-THCA dataset, including transcriptome files and clinical details, was downloaded using the "TCGAbiolinks" R package[1]. After excluding samples with incomplete prognostic information and non-papillary thyroid cancer samples, a total of 505 PTC samples were collected for subsequent analysis. The expression data format was converted to Transcripts Per Million (TPM) for further analysis.

**2. Data Preprocessing**

Single-cell data were preprocessed utilizing the Seurat R package (version 4.3.0) to ensure the acquisition of high-quality cells. The following quality control criteria were applied to exclude low-quality cells and potential doublets: 1. 500 < nCount_RNA < 5000; 2. The proportion of mitochondrial genes in the cells was less than 10%. Subsequently, the DoubletFinder R package[2] was utilized to remove potential doublets. The Harmony R package was then performed to correct for batch effects present in the data. The single-cell matrix was normalized using the SCTransform function, followed by dimensionality reduction with the top 3,000 highly variable genes to distinguish different cell clusters. Public databases, such as the CellMarker website[3], and markers from published studies were used to annotate the various cell clusters. Ultimately, six major cell types were identified for further analysis.

**3. Single-cell copy number analysis**

Single-cell expression profiles of three primary PTC cases were extracted, and the "SCEVAN" R package[4] was utilized to infer malignant cells within the primary PTC samples. Following the segregation of benign and malignant cells from the primary PTC, the malignant epithelial cells were integrated with epithelial cells from metastatic PTC and subjected to dimensionality reduction and clustering as previously described. This process ultimately identified nine distinct tumor cell subpopulations.

**4. Pseudotime and enrichment analyses**

The "Monocle2" R package (version 2.26.0)[5] was used to analyze all epithelial cells. The dimensionality reduction method was set to "DDRtree", with a maximum number of dimensions set to 2. The Beam analysis within the "Monocle" R package was utilized to explore the dynamic changes in genes between branching points. Genes from different clusters were subjected to GO annotation using the "clusterProfiler" R package. Additionally, the "UCell" and "irGSEA" R packages were employed to analyze the activity of 50 hallmark pathways in tumor subpopulation cells.

**5. Cell-cell communication analysis**

To evaluate the proportions of different cell types within bulk RNA sequencing data, we utilized the CIBERSORTx web tool. We constructed a single-cell expression matrix by extracting 50 tumor cell subpopulations of each type. This matrix, along with bulk RNA data of PTC from the TCGA-THCA dataset, was uploaded to the CIBERSORTx website (<https://cibersortx.stanford.edu/>). This process enabled us to determine the proportions of each tumor subpopulation in every sample within the TCGA-THCA dataset. Subsequently, we analyzed disease-free survival (DFS) by integrating survival information with the estimated subpopulation proportions.

The "CellChat" R package[6] was utilized to analyze potential receptor-ligand interactions between cell subpopulations. The CellChatDB human database was employed to predict potential cellular communication interactions based on known receptor-ligand pairs.

**6. Weighted Gene Co-expression Network Analysis**

The "hdWGCNA" R package was employed for co-expression gene modules analyzing within tumor subpopulation cells. A soft threshold of 7 was selected based on the variability in expression profiles. The single-cell expression information of all tumor cells was categorized into 8 co-expression modules, where genes within each module exhibited similar expression patterns. Subsequently, the single sample gene set enrichment analysis (ssGSEA) algorithm was applied to calculate the scores for each of the 8 modules in each sample and to perform a correlation analysis with clinical information, including T, N and M stage. The Spearman method was used to determine the correlation coefficients.

**7. Thyroid Cancer Specimens**

The thyroid cancer tissues and their corresponding peritumoral tissues from 50 patients with PTC were collected from Tianjin Medical University General Hospital. All sample diagnoses were confirmed histologically. This study was approved by the Ethical Committee of Tianjin Medical University General Hospital and adhered to the ethical guidelines of the Helsinki Declaration. PTC tissues and their corresponding peritumoral tissues were rapidly frozen with liquid nitrogen and placed at -80°C.

**8. Cell Cultures and Transfection**

The PTC cell lines TPC-1, B-CPAP, K-1, and Nthy-ori-3-1 were all derived from the General Surgery Laboratory of Tianjin Medical University General Hospital. TPC-1 and B-CPAP were cultured in 1640 (Gibco, USA), K-1 was cultured in DMEM (Gibco, USA), and Nthy-ori-3-1 was cultured in F-12K (Gibco, USA). All media included 1% penicillin/streptomycin (Solarbio, China) and 10% fetal bovine serum (NEWZERUM, Australia). The cells were maintained at 37°C in a humidified cell incubator with 5% CO₂. S100A10 lentiviral-based expression constructs were prepared using standard molecular cloning techniques with pCDH-CMV-S100A10-Flag-Puro vectors. The siRNAs targeting S100A10 and RAN constructs were procured from RiboBio (Guangzhou, China), and their oligonucleotide sequences are listed in Additional Table S1. Lentiviral particles expressing shRNA against S100A10 were commercially packaged by RiboBio, with the shRNA construct designed based on the siS100A10-2 sequence. The operation of cell transfection was performed as previous described[7, 8].Cells were also treated with 10 μM LY294002 (MCE, USA).

**9. Reverse Transcription-Quantitative PCR (RT‒qPCR) Analysis**

Total RNA was extracted using Trizol according to the manufacturer's instructions (Invitrogen, USA) and was reverse transcribed using a reverse transcription kit (Vazyme, China). For RT-qPCR, 2 μL of cDNA was mixed with primers and SYBR Green PCR Master Mix (Vazyme, China). The S100A10 and GAPDH primers were purchased from Synbio Technologies (Suzhou, China). All primers involved are listed in Additional Table S2.

**10. Western Blot and Antibodies**

Cells were lysed using RIPA buffer with phosphorylase inhibitor and protease inhibitor mixture (MCE, USA). Lysates (20 μL) were subjected to SDS-PAGE for protein separation and then transferred to a PVDF membrane. Primary antibodies were diluted 1:1000 and incubated on the membrane. Detailed information about the antibodies is provided in Additional Table S3. The target proteins were immunoblotted with corresponding antibodies, and an ECL reagent (Vazyme, China) was used for visualization. The band intensity of each protein was quantified using ImageJ software and normalized to GAPDH.

**11. Immunohistochemistry (IHC) and Cell Invasion Assays**

The formalin-fixed, paraffin-embedded tissues were deparaffinized and rehydrated, and boiled in sodium citrate buffer for antigen retrieval. The tissues were treated with primary antibodies at a 1:100 dilution overnight at 4°C, followed by HRP-conjugated secondary antibody treatment and incubated by 3,3′-diaminobenzidine. The tissues were observed and captured using a microscope. Each section was independently assessed and scored by two experienced pathologists. The standard score for the extent of staining was conducted as previous described[9]. Additional Table S3 provides information on the antibodies used.

Transwell assays (with or without Matrigel) and wound healing assays were performed to assess cell invasion and migration. All experiments were performed as previously described[8, 10].

**12. Immunofluorescence (IF)**

Cells were seeded onto glass coverslips at a density of 3×10⁴ cells per well, followed by washing, fixation, and permeabilization. The coverslips were treated with primary antibodies overnight at 4°C. Subsequently, the FITC/TRITC-conjugated secondary antibodies were added onto the coverslips for 1 hour at room temperature. After 4′,6-diamidino-2-phenylindole (DAPI) staining, the coverslips were observed and analyzed using a fluorescence microscope (Zeiss).

**13. Xenograft modeling**

Stable shS100A10 B-CPAP cells and control cells, mixed with 5% Matrigel (BD Biosciences, USA), were injected into the inguinal region of 5-week-old severe combined immune-deficient (SCID) mice at the total number of 5×10⁶ cells per entity, and raised for 6 weeks. The tumor growth was recorded weekly. After euthanasia, the volume and weight of the tumors were measured. To assess metastatic ability, cells were injected intravenously. The Xenogen IVIS 200 Imaging System (Caliper Life Sciences, USA) was utilized to determine the fluorescence intensity at 7, 14, 21, 28, and 35 days. The tumors and lungs obtained from mice were sectioned for hematoxylin-eosin (HE) staining and IHC analysis. Some of mice were treated with 740 YP 10 mg/kg. Each single group contained six mice, and the experiments were approved by the Ethics Committee of Tianjin Medical University General Hospital.

**14. Luciferase reporter assay**

The S100A10 promoter fragments spanning1 to 500, 501 to 1000, 1001 to 1500, and 1501 to 2000 bp relative to the transcription start site were cloned into the pGL3-basic vector (Promega) upstream of the Firefly luciferase reporter gene. The inserted sequences were listed in Supplementary Table S4. For dual-luciferase reporter assays, TPC-1 cells were seeded in 24-well plates and co-transfected with 0.5μg of the respective promoter construct and 25ng renilla luciferase reporter vector using Lipofectamine 3000 (Thermo Fisher Scientific, USA). After 48-hour incubation, cell lysates were analyzed using Dual-Glo Luciferase system (Promega), with Firefly luciferase activity normalized to Renilla luciferase activity to account for transfection efficiency. All experiments were performed in triplicate.

**15. Statistical analysis**

The data were showed as the mean ± standard deviation (SD) based on at least three repeated individual experiments. All R language analyses in this paper were conducted using R version 4.2.2. Quantitative results were analyzed with a two-tailed Student's *t*-test. Prism 8 (GraphPad Software, CA) was applied for data visualization. A *p*-value of less than 0.05 was considered statistically significant.

**Reference**

1. Colaprico A, Silva TC, Olsen C, Garofano L, Cava C, Garolini D, Sabedot TS, Malta TM, Pagnotta SM, Castiglioni I *et al*: **TCGAbiolinks: an R/Bioconductor package for integrative analysis of TCGA data**. *Nucleic Acids Res* 2016, **44**(8):e71.

2. McGinnis CS, Murrow LM, Gartner ZJ: **DoubletFinder: Doublet Detection in Single-Cell RNA Sequencing Data Using Artificial Nearest Neighbors**. *Cell Syst* 2019, **8**(4):329-337.e324.

3. Hu C, Li T, Xu Y, Zhang X, Li F, Bai J, Chen J, Jiang W, Yang K, Ou Q *et al*: **CellMarker 2.0: an updated database of manually curated cell markers in human/mouse and web tools based on scRNA-seq data**. *Nucleic Acids Res* 2023, **51**(D1):D870-d876.

4. De Falco A, Caruso F, Su XD, Iavarone A, Ceccarelli M: **A variational algorithm to detect the clonal copy number substructure of tumors from scRNA-seq data**. *Nat Commun* 2023, **14**(1):1074.

5. Qiu X, Mao Q, Tang Y, Wang L, Chawla R, Pliner HA, Trapnell C: **Reversed graph embedding resolves complex single-cell trajectories**. *Nat Methods* 2017, **14**(10):979-982.

6. Jin S, Guerrero-Juarez CF, Zhang L, Chang I, Ramos R, Kuan CH, Myung P, Plikus MV, Nie Q: **Inference and analysis of cell-cell communication using CellChat**. *Nat Commun* 2021, **12**(1):1088.

7. Tian Y, Yu Y, Hou LK, Chi JR, Mao JF, Xia L, Wang X, Wang P, Cao XC: **Serum deprivation response inhibits breast cancer progression by blocking transforming growth factor-β signaling**. *Cancer Sci* 2016, **107**(3):274-280.

8. Chen ZH, Tian Y, Zhou GL, Yue HR, Zhou XJ, Ma HY, Ge J, Wang X, Cao XC, Yu Y: **CMTM7 inhibits breast cancer progression by regulating Wnt/β-catenin signaling**. *Breast Cancer Res* 2023, **25**(1):22.

9. Zhu K, Wang B, Li Y, Yu Y, Chen Z, Yue H, Meng Q, Tian D, Liu X, Shen W *et al*: **CAVIN2/SDPR Functioned as a Tumor Suppressor in Lung Adenocarcinoma from Systematic Analysis of Caveolae-Related Genes and Experimental Validation**. *J Cancer* 2023, **14**(11):2001-2014.

10. Li Y, Wu J, Tian Y, Zhu Q, Ge Y, Yu H, Huang J, Li H, Zhang J, Zhang L *et al*: **MED1 Downregulation Contributes to TGFβ-Induced Metastasis by Inhibiting SMAD2 Ubiquitination Degradation in Cutaneous Melanoma**. *J Invest Dermatol* 2022, **142**(8):2228-2237.e2224.
