# Supplementary material for: RAN-S100A10-EGFR axis facilitates papillary thyroid cancer metastasis by PI3K/AKT signaling
Source: Cell Death Dis. 2026 Apr 16;17(1):510. doi: 10.1038/s41419-026-08649-6 (PMC13201611; doi:10.1038/s41419-026-08649-6)
Supplement: Supplementary file 3 — Additional table [file 41419_2026_8649_MOESM3_ESM.docx]

**Additional table1**

| gene_name | module | color | kME_grey | kME_Epi1 | kME_Epi2 | kME_Epi3 | kME_Epi4 | kME_Epi5 | kME_Epi6 | kME_Epi7 | kME_Epi8 |
| --- | --- | --- | --- | --- | --- | --- | --- | --- | --- | --- | --- |
| MXRA8 | Epi1 | red | 0.127984335 | 0.211924822 | 0.058828675 | 0.129366138 | 0.049041242 | 0.027709016 | 0.029612908 | 0.103598292 | 0.039778912 |
| FBXO2 | Epi1 | red | 0.113870331 | 0.217711664 | 0.071430324 | 0.08932229 | -0.016431768 | 0.047788357 | -0.027980151 | 0.182265037 | -0.042461966 |
| TMEM50A | Epi1 | red | 0.18283233 | 0.206461405 | 0.14855829 | 0.195821098 | 0.065010589 | 0.084711013 | 0.04839126 | 0.149049697 | 0.123699202 |
| SH3BGRL3 | Epi1 | red | 0.292193566 | 0.299045285 | 0.258479121 | 0.169046681 | 0.121779463 | 0.134388498 | 0.011434525 | 0.285386574 | 0.07525893 |
| IFI6 | Epi1 | red | 0.195728919 | 0.376939077 | 0.105266755 | 0.177253467 | 0.026099763 | 0.126621197 | 0.071011333 | 0.119703444 | 0.077145714 |
| MARCKSL1 | Epi1 | red | 0.266482081 | 0.217634988 | 0.225602676 | 0.129660784 | 0.168242858 | 0.092525506 | 0.009138267 | 0.197017874 | 0.076025329 |
| TRAPPC3 | Epi1 | red | 0.16922791 | 0.174148092 | 0.124475205 | 0.108785266 | 0.104426829 | 0.071660287 | 0.03852199 | 0.131799625 | 0.063395877 |
| PDZK1IP1 | Epi1 | red | 0.228435971 | 0.427740187 | 0.131878414 | 0.161700658 | 0.071341308 | 0.068210914 | -0.008199851 | 0.227396816 | 0.008352188 |
| GADD45A | Epi1 | red | 0.119455569 | 0.221110897 | 0.000635747 | 0.066898332 | 0.010872688 | 0.031255709 | 0.065879036 | 0.057085533 | -0.045731397 |
| GBP1 | Epi1 | red | 0.083031947 | 0.181794986 | -0.01625471 | 0.025574915 | -0.017085722 | 0.01569154 | 0.025422994 | 0.012429778 | -0.049353356 |
| S100A10 | Epi1 | red | 0.299201842 | 0.533357566 | 0.213265479 | 0.282937906 | 0.103705859 | 0.087558273 | -0.01879843 | 0.258069948 | 0.099048366 |
| S100A6 | Epi1 | red | 0.318479747 | 0.394751583 | 0.325336817 | 0.287407999 | 0.254016315 | 0.094755836 | -0.026314685 | 0.2686038 | 0.224007977 |
| S100A16 | Epi1 | red | 0.150459404 | 0.187690454 | 0.100736005 | 0.108935515 | 0.072885789 | 0.047171434 | -0.001178066 | 0.105057297 | 0.044376956 |
| S100A14 | Epi1 | red | 0.163997601 | 0.269562147 | 0.053711924 | 0.06764431 | 0.16271549 | 0.042697983 | 0.024260894 | 0.065620237 | -0.011615188 |
| GLMP | Epi1 | red | 0.044863979 | 0.093218201 | 0.001068775 | 0.084587391 | -0.007525209 | 0.03846061 | 0.026099437 | 0.040457372 | 0.008378052 |
| CRABP2 | Epi1 | red | 0.133744836 | 0.305132098 | 0.048476107 | 0.134309715 | -0.023788333 | 0.040593126 | -0.059732522 | 0.189137178 | -0.05202921 |
| IFI16 | Epi1 | red | 0.10823748 | 0.166287198 | 0.054937076 | 0.107227796 | 0.023070789 | 0.024954742 | 0.010909858 | 0.083774865 | 0.030052278 |
| DUSP23 | Epi1 | red | 0.180298258 | 0.251358417 | 0.122331801 | 0.130776823 | 0.098470867 | 0.052452274 | 0.022301612 | 0.118021558 | 0.06404103 |
| TOR3A | Epi1 | red | 0.09191248 | 0.122816063 | 0.055392771 | 0.084776648 | 0.035405074 | 0.027872216 | 0.024269401 | 0.063368557 | 0.026170722 |
| XPR1 | Epi1 | red | 0.101689436 | 0.149343215 | 0.064648949 | 0.035899229 | 0.031066563 | 0.048225334 | 0.006206538 | 0.084516308 | -0.003477428 |
| CHI3L1 | Epi1 | red | 0.149021811 | 0.520636948 | 0.046746039 | 0.238151644 | -0.125544215 | 0.036208852 | -0.10543976 | 0.300600682 | -0.069821603 |
| KLHDC8A | Epi1 | red | 0.071172949 | 0.169299272 | 0.042178229 | 0.041120923 | -0.013241791 | 0.014603224 | -0.047836122 | 0.130423581 | -0.047786251 |
| CD46 | Epi1 | red | 0.126100102 | 0.193345678 | 0.048900624 | 0.142367382 | 0.013607003 | 0.033342201 | 0.07136351 | 0.07185541 | 0.059993913 |
| RHOU | Epi1 | red | 0.139890898 | 0.200090282 | 0.120884514 | 0.082499589 | 0.024051319 | 0.059054533 | -0.042079255 | 0.175078033 | -0.00499927 |
| FAM49A | Epi1 | red | 0.098731709 | 0.143989335 | 0.062061628 | 0.051822184 | -0.013040606 | 0.034014945 | -0.040967307 | 0.138059758 | -0.025772478 |
| RHOB | Epi1 | red | 0.187966935 | 0.290482967 | 0.073024069 | 0.033435721 | 0.056934865 | 0.064718359 | 0.015387156 | 0.111390667 | -0.049082518 |
| CDC42EP3 | Epi1 | red | 0.127971101 | 0.175429267 | 0.067860182 | 0.079945142 | 0.053668887 | 0.040549263 | 0.019183289 | 0.113902497 | 0.003128546 |
| CYP1B1 | Epi1 | red | 0.185875068 | 0.341917663 | 0.115750743 | 0.103875119 | 0.005954895 | 0.055844929 | -0.040828849 | 0.209915375 | -0.018775397 |
| RTN4 | Epi1 | red | 0.168035335 | 0.179567796 | 0.054252885 | 0.151656561 | 0.037848488 | 0.058339523 | 0.080543366 | 0.092774557 | 0.029267146 |
| REL | Epi1 | red | 0.133788869 | 0.193506527 | 0.091928908 | 0.045598685 | 0.040938143 | 0.042669973 | -0.024157421 | 0.125701185 | -0.01746396 |
| VAMP8 | Epi1 | red | 0.263191533 | 0.315749328 | 0.190930482 | 0.187121939 | 0.149362212 | 0.128662638 | 0.037280109 | 0.200092907 | 0.076967304 |
| SFTPB | Epi1 | red | 0.15950952 | 0.534902273 | -0.004316236 | 0.112060296 | -0.025422701 | 0.062562866 | -0.037122556 | 0.135554618 | -0.120380508 |
| FAM178B | Epi1 | red | 0.051876931 | 0.228621961 | 0.029777648 | 0.051926466 | -0.06867096 | 0.00403546 | -0.117408388 | 0.206374337 | -0.102508253 |
| GALNT3 | Epi1 | red | 0.053309238 | 0.139853378 | -0.042022943 | 0.056017453 | 0.018515277 | -0.005581179 | 0.06414559 | -0.008120317 | -0.018512271 |
| ITGA6 | Epi1 | red | 0.100378952 | 0.153609968 | 0.089169285 | 0.050893126 | 0.013743236 | 0.031363018 | -0.058816655 | 0.115518939 | 0.007731803 |
| SLC40A1 | Epi1 | red | 0.137360823 | 0.184383826 | 0.120008409 | 0.112597298 | 0.039860708 | 0.054096273 | -0.030342851 | 0.165062264 | 0.062004654 |
| STAT1 | Epi1 | red | 0.176741511 | 0.262826447 | 0.092516184 | 0.063190847 | 0.069965751 | 0.052647465 | 0.055221339 | 0.073858516 | 0.031617631 |
| CLK1 | Epi1 | red | 0.1351212 | 0.150606438 | 0.078672609 | 0.151032265 | 0.022548778 | 0.128336567 | 0.043832187 | 0.11270363 | 0.046520567 |
| NRP2 | Epi1 | red | 0.098044588 | 0.152682846 | 0.039984701 | 0.048487569 | 0.033499306 | 0.046571485 | -0.002624747 | 0.07997597 | -0.019096619 |
| OTOS | Epi1 | red | 0.067506335 | 0.106038013 | -0.018515151 | 0.128003424 | -0.091753769 | 0.022955848 | 0.113553169 | 0.066531606 | -0.023165214 |
| PPP1R7 | Epi1 | red | 0.172182042 | 0.253766867 | 0.132778158 | 0.15936766 | 0.038427718 | 0.062558187 | -0.018918049 | 0.16400308 | 0.044125586 |
| AC018816.3 | Epi1 | red | 0.111267057 | 0.179900899 | 0.101685327 | 0.070181324 | 0.044041096 | 0.0448156 | -0.032085976 | 0.119066126 | 0.01116504 |
| CMTM7 | Epi1 | red | 0.097608806 | 0.131616868 | 0.060543929 | 0.071979529 | 0.056103333 | 0.040275832 | -0.000380268 | 0.077434473 | 0.030538777 |
| CMTM6 | Epi1 | red | 0.228591685 | 0.207309033 | 0.212374989 | 0.190370205 | 0.101296942 | 0.077026995 | 0.032694099 | 0.195384575 | 0.143846413 |
| HHATL | Epi1 | red | 0.079631761 | 0.259520914 | 0.022092587 | 0.136234445 | -0.060710685 | 0.03509092 | -0.025087429 | 0.150669476 | -0.032764961 |
| CISH | Epi1 | red | 0.100539956 | 0.199969852 | 0.058303836 | 0.113434772 | -0.027062921 | 0.041251016 | -0.012353755 | 0.145549463 | -0.006880351 |
| MAPKAPK3 | Epi1 | red | 0.127062937 | 0.210231832 | 0.095105443 | 0.03696855 | 0.059130591 | 0.040959061 | -0.039929541 | 0.119239934 | -0.010018504 |
| DCBLD2 | Epi1 | red | 0.122697085 | 0.156274844 | 0.104363674 | 0.041246168 | 0.064263719 | 0.088613117 | -0.019267834 | 0.095363119 | 0.023055147 |
| COL8A1 | Epi1 | red | 0.132958873 | 0.209353534 | 0.088477842 | 0.124489154 | 0.030406941 | 0.042720241 | 0.024482679 | 0.114821557 | 0.059360717 |
| TM4SF1 | Epi1 | red | 0.184670665 | 0.242582741 | 0.130008736 | 0.20499526 | 0.081863203 | 0.045475102 | 0.042149346 | 0.123694672 | 0.115809763 |
| CXCL8 | Epi1 | red | 0.107414631 | 0.223152935 | 0.048189494 | 0.036710061 | 0.035337916 | 0.051921771 | 0.010676744 | 0.046669088 | -0.022105528 |
| CXCL2 | Epi1 | red | 0.118243998 | 0.297093432 | 0.029718446 | 0.027864154 | 0.044235662 | 0.049290195 | 0.009640525 | 0.026034588 | -0.051272223 |
| EMB | Epi1 | red | 0.123550629 | 0.169098691 | 0.091862 | 0.047326349 | 0.041006802 | 0.023566968 | -0.005587409 | 0.111911244 | 0.014804121 |
| MAST4 | Epi1 | red | 0.101742323 | 0.194580304 | 0.025356433 | 0.047176677 | -0.020152907 | 0.027762444 | -0.019084831 | 0.111395808 | -0.064733376 |
| FCHO2 | Epi1 | red | 0.079459898 | 0.14294409 | 0.055198177 | 0.047161417 | 0.007764617 | 0.044936957 | -0.026526965 | 0.102014702 | -0.007597853 |
| SSBP2 | Epi1 | red | 0.125936213 | 0.238424746 | 0.057630297 | 0.12433815 | -0.01139367 | 0.059208789 | -0.072026171 | 0.16758316 | -0.010398946 |
| PDLIM4 | Epi1 | red | 0.189706178 | 0.270372038 | 0.168785907 | 0.203474429 | 0.044612268 | 0.063970517 | -0.02375532 | 0.223081385 | 0.097359283 |
| TMEM173 | Epi1 | red | 0.132742014 | 0.205926847 | 0.118960587 | 0.124143752 | 0.031605235 | 0.062390242 | -0.060810363 | 0.192113721 | 0.050360679 |
| CYSTM1 | Epi1 | red | 0.230387127 | 0.36104063 | 0.184910908 | 0.227094437 | 0.027069759 | 0.055810941 | -0.012855063 | 0.254233019 | 0.059655891 |
| HBEGF | Epi1 | red | 0.121311299 | 0.231063774 | 0.028236765 | -0.008055639 | 0.024943071 | 0.028948368 | 0.008391894 | 0.029278133 | -0.039065122 |
| CD74 | Epi1 | red | 0.215414107 | 0.753635677 | -0.00066395 | 0.237755477 | -0.064897435 | 0.061145787 | -0.037062433 | 0.20442704 | -0.107781964 |
| GPX3 | Epi1 | red | 0.153467564 | 0.248224742 | -0.093500579 | 0.218646367 | 0.0100244 | 0.063460448 | 0.280628403 | -0.049990975 | 0.011263456 |
| DUSP1 | Epi1 | red | 0.207249289 | 0.259015256 | 0.00584165 | 0.214408799 | -0.006937974 | 0.06770809 | 0.17931426 | 0.078359105 | 0.013732133 |
| PDLIM7 | Epi1 | red | 0.117557878 | 0.262109595 | 0.099984089 | 0.123144607 | -0.027380095 | 0.052661285 | -0.102857751 | 0.234978926 | -0.026423277 |
| HLA-E | Epi1 | red | 0.177713043 | 0.426722476 | 0.024784058 | 0.174354611 | 0.004313442 | 0.06953923 | 0.051790655 | 0.108117952 | -0.018598208 |
| IER3 | Epi1 | red | 0.130873803 | 0.298504128 | 0.101177748 | 0.114232772 | 0.028738042 | 0.059995487 | -0.050836457 | 0.164635926 | 0.004514321 |
| HLA-C | Epi1 | red | 0.221758453 | 0.579665124 | 0.060049821 | 0.397469098 | -0.028926976 | 0.059292239 | 0.091225811 | 0.188306099 | 0.085750535 |
| HLA-B | Epi1 | red | 0.228205023 | 0.599207127 | 0.056981402 | 0.348234171 | -0.051214673 | 0.069255554 | 0.026992813 | 0.239861752 | 0.023466489 |
| CLIC1 | Epi1 | red | 0.284316365 | 0.273176214 | 0.289369764 | 0.281512783 | 0.13897276 | 0.098623923 | 0.021379224 | 0.288092043 | 0.191681356 |
| HLA-DRA | Epi1 | red | 0.17184562 | 0.681942226 | -0.016582023 | 0.250277753 | -0.071561496 | 0.044972399 | -0.024555943 | 0.180275969 | -0.092762585 |
| HLA-DRB5 | Epi1 | red | 0.209670936 | 0.686611089 | 0.01594239 | 0.205445536 | -0.058131135 | 0.060366753 | -0.034667079 | 0.22121744 | -0.117162148 |
| HLA-DRB1 | Epi1 | red | 0.183337651 | 0.694573496 | 0.003694862 | 0.245903736 | -0.067331002 | 0.047053048 | -0.036412338 | 0.204939163 | -0.091412415 |
| HLA-DQB1 | Epi1 | red | 0.159189986 | 0.562395436 | 0.004941197 | 0.177458877 | -0.044915184 | 0.044510997 | -0.017847885 | 0.163601293 | -0.092052965 |
| PSMB8 | Epi1 | red | 0.133644235 | 0.222118466 | 0.060135004 | 0.123663648 | 0.030886857 | 0.021361161 | 0.034991934 | 0.119235181 | 0.022833026 |
| PSMB9 | Epi1 | red | 0.098522318 | 0.26540138 | 0.007491511 | 0.11852745 | -0.01463834 | 0.014995053 | 0.033674999 | 0.085686633 | -0.026339079 |
| HLA-DMA | Epi1 | red | 0.175294831 | 0.563403631 | 0.007996778 | 0.187951204 | -0.055586736 | 0.049156625 | -0.009128008 | 0.180793005 | -0.093579181 |
| HLA-DPA1 | Epi1 | red | 0.172469798 | 0.669362713 | -0.005461155 | 0.214017624 | -0.068627721 | 0.043422973 | -0.048317914 | 0.184453328 | -0.097533687 |
| HLA-DPB1 | Epi1 | red | 0.207980941 | 0.686275691 | 0.024994006 | 0.186551628 | -0.027174137 | 0.071596426 | -0.038575305 | 0.199994072 | -0.11437765 |
| CDKN1A | Epi1 | red | 0.173394178 | 0.394885934 | 0.079206328 | 0.061118823 | 0.045958098 | 0.045437128 | -0.04681458 | 0.136439911 | -0.069383396 |
| NT5E | Epi1 | red | 0.140080583 | 0.221597356 | 0.121517481 | 0.117176622 | 0.065979018 | 0.046342553 | -0.036688838 | 0.164844929 | 0.053787207 |
| NCOA7 | Epi1 | red | 0.115568908 | 0.209732803 | 0.012932516 | 0.040463465 | 0.052420884 | 0.026871186 | 0.030204336 | 0.023260249 | -0.017989002 |
| SOD2 | Epi1 | red | 0.095315937 | 0.127631314 | 0.040002859 | 0.086526715 | 0.031232234 | 0.030413972 | 0.05533623 | 0.04928995 | 0.010021226 |
| FZD1 | Epi1 | red | 0.101973776 | 0.148807184 | 0.079496491 | 0.049690095 | 0.02820253 | 0.05649325 | -0.018930762 | 0.12651847 | -0.021458951 |
| AKAP9 | Epi1 | red | 0.169230145 | 0.197135731 | 0.142763824 | 0.143073443 | 0.033822605 | 0.067275778 | 0.003414897 | 0.176808926 | 0.074044057 |
| CALD1 | Epi1 | red | 0.174714885 | 0.266053274 | 0.019378283 | 0.120195488 | 0.076920553 | 0.084875657 | 0.129075689 | 0.047638077 | 0.004733069 |
| CD99 | Epi1 | red | 0.154106937 | 0.173976898 | 0.08787895 | 0.155088404 | 0.082359887 | 0.041497216 | 0.056819143 | 0.092419015 | 0.083239163 |
| PCSK1N | Epi1 | red | 0.175822535 | 0.39101044 | 0.063526109 | 0.100624046 | 0.083913653 | 0.042678544 | -0.005867538 | 0.122788285 | -0.059277919 |
| PLP2 | Epi1 | red | 0.129353788 | 0.231192892 | 0.130412512 | 0.08398636 | 0.087591012 | 0.059310394 | -0.070730643 | 0.160289964 | 0.030285183 |
| CITED1 | Epi1 | red | 0.151037848 | 0.44381313 | 0.054061462 | 0.11203815 | -0.070575606 | 0.055641359 | -0.112977429 | 0.237277883 | -0.125816392 |
| RNF128 | Epi1 | red | 0.104335676 | 0.155681132 | 0.077668012 | 0.08043865 | 0.037528732 | 0.046004182 | 0.001450631 | 0.10880688 | 0.01127546 |
| TSC22D3 | Epi1 | red | 0.109630374 | 0.157622609 | -0.04689172 | 0.158639507 | -0.0645568 | 0.046106376 | 0.12631418 | 0.072099164 | -0.042475258 |
| LAGE3 | Epi1 | red | 0.117868726 | 0.13890253 | 0.085293742 | 0.083777622 | 0.05619652 | 0.039226954 | -0.005035554 | 0.137465921 | 0.02766347 |
| CTSB | Epi1 | red | 0.234716331 | 0.259096478 | 0.046772835 | 0.322873505 | 0.054748852 | 0.073950677 | 0.225216121 | 0.092753937 | 0.137526581 |
| PEBP4 | Epi1 | red | 0.101076557 | 0.257513716 | -0.000281733 | 0.139268447 | -0.085433153 | 0.022863137 | 0.014996718 | 0.160196772 | -0.06280746 |
| C8orf4 | Epi1 | red | 0.182976225 | 0.382947204 | 0.082254428 | 0.103883759 | 0.010327483 | 0.07441027 | -0.04175103 | 0.165618605 | -0.020386531 |
| SGK3 | Epi1 | red | 0.096846711 | 0.191068245 | 0.05904321 | 0.056232439 | -0.040561105 | 0.036581394 | -0.024604458 | 0.171018615 | -0.049900933 |
| FABP5 | Epi1 | red | 0.084998416 | 0.245478267 | 0.026717374 | 0.099919046 | -0.017989944 | -0.005603664 | -0.038319865 | 0.166914371 | -0.058723961 |
| CLIC3 | Epi1 | red | 0.033117166 | 0.018175852 | -0.140724703 | 0.176408628 | -0.074236138 | -0.006352814 | 0.261659586 | -0.061215121 | -0.013921218 |
| DPP7 | Epi1 | red | 0.10798503 | 0.155710013 | 0.013706021 | 0.180384563 | -0.009783517 | 0.033864343 | 0.078515032 | 0.080065229 | 0.048752575 |
| PHLDA2 | Epi1 | red | 0.193353222 | 0.345861357 | 0.204286411 | 0.134160366 | 0.092295374 | 0.064052409 | -0.100501995 | 0.219825707 | 0.046731511 |
| TEAD1 | Epi1 | red | 0.112916523 | 0.123316073 | 0.053738218 | 0.045527545 | 0.017845436 | 0.048452061 | 0.016148883 | 0.082051631 | -0.006953604 |
| SERGEF | Epi1 | red | 0.156095582 | 0.214762486 | 0.092112599 | 0.102307679 | 0.127287075 | 0.048840285 | 0.002183298 | 0.118356697 | 0.040171977 |
| LDHA | Epi1 | red | 0.223566395 | 0.353268797 | 0.113370657 | 0.231557668 | 0.074902891 | 0.0821114 | 0.081252525 | 0.150470956 | 0.073900431 |
| HTATIP2 | Epi1 | red | 0.135827976 | 0.18245646 | 0.073375946 | 0.11612079 | 0.012456463 | 0.031654748 | 0.03228647 | 0.129402471 | 0.013943542 |
| CD44 | Epi1 | red | 0.131998216 | 0.167026941 | 0.079398993 | 0.110928052 | 0.048837175 | 0.047598284 | -0.002429284 | 0.159307957 | 0.004267997 |
| CHST1 | Epi1 | red | 0.118309019 | 0.339780901 | 0.050675589 | 0.065426277 | -0.05798638 | 0.048012477 | -0.079486799 | 0.199738014 | -0.109026367 |
| MDK | Epi1 | red | 0.22448351 | 0.445934094 | 0.048879094 | 0.141510631 | 0.049841777 | 0.062778094 | 0.063435277 | 0.111558143 | -0.030899803 |
| UBE2L6 | Epi1 | red | 0.077555504 | 0.142175606 | 0.021126424 | 0.095659645 | 0.025046021 | 0.019576141 | 0.055408911 | 0.032662988 | 0.036621352 |
| RARRES3 | Epi1 | red | 0.140391007 | 0.264444582 | 0.093699808 | 0.194694068 | 0.00726621 | 0.043550549 | 0.034487478 | 0.152177758 | 0.05350514 |
| CTSC | Epi1 | red | 0.165009952 | 0.42085634 | 0.10238803 | 0.300462732 | 0.021563424 | 0.023706002 | -0.005422804 | 0.173755943 | 0.118229806 |
| SMCO4 | Epi1 | red | 0.152471874 | 0.301919484 | 0.098358914 | 0.093008709 | 0.009403405 | 0.052076046 | -0.049473884 | 0.207850071 | -0.043978002 |
| MPZL2 | Epi1 | red | 0.18912756 | 0.294319716 | 0.142107608 | 0.238866408 | 0.056243804 | 0.081374614 | -0.004431458 | 0.194833211 | 0.110542655 |
| SORL1 | Epi1 | red | 0.093194397 | 0.16230641 | 0.096223162 | 0.052429772 | -0.046128451 | 0.023014193 | -0.080531649 | 0.177425994 | -0.029463031 |
| NRP1 | Epi1 | red | 0.104219222 | 0.22512236 | 0.048273319 | 0.050818168 | 0.001170997 | 0.034984963 | -0.035798171 | 0.105689664 | -0.032638541 |
| SLC16A9 | Epi1 | red | 0.135793145 | 0.224812719 | 0.155029103 | 0.076023894 | -0.028610997 | 0.042202839 | -0.10533126 | 0.26381679 | -0.048589502 |
| SFTPA1 | Epi1 | red | 0.09290652 | 0.256808032 | 0.010046898 | 0.031911848 | -0.009287774 | 0.020071555 | 0.00732129 | 0.025234679 | -0.044140925 |
| FAM213A | Epi1 | red | 0.140822592 | 0.202233018 | 0.069359737 | 0.126498137 | 0.017302562 | 0.084248393 | 0.032784305 | 0.136681019 | -0.003011797 |
| SNCG | Epi1 | red | 0.100648555 | 0.225392419 | 0.062110397 | 0.037017878 | 0.014977144 | 0.079577644 | -0.040923935 | 0.136723875 | -0.070698047 |
| UBTD1 | Epi1 | red | 0.073336686 | 0.135494319 | 0.017935053 | 0.03327074 | 0.025022356 | 0.05474613 | 0.004542836 | 0.038132177 | -0.019003585 |
| SCNN1A | Epi1 | red | 0.083417334 | 0.188615929 | 0.010077985 | 0.030257382 | 0.028551518 | 0.035474456 | 0.011492374 | 0.047461199 | -0.033145643 |
| PLEKHA5 | Epi1 | red | 0.102690069 | 0.168241598 | 0.042844406 | 0.055328961 | 0.047383973 | 0.021223492 | 0.022097732 | 0.048032276 | 0.010050892 |
| NR4A1 | Epi1 | red | 0.1216226 | 0.218231835 | -0.028303907 | 0.055138115 | -0.025713755 | 0.043000993 | 0.09842189 | 0.01661326 | -0.066246907 |
| KRT8 | Epi1 | red | 0.145949889 | 0.189511493 | 0.025159976 | 0.237303924 | 0.01179754 | 0.032404253 | 0.195891407 | 0.070190819 | 0.084001471 |
| CNPY2 | Epi1 | red | 0.208026636 | 0.193293399 | 0.176865515 | 0.173045518 | 0.120236595 | 0.072186588 | 0.046626308 | 0.179462564 | 0.112866224 |
| BTG1 | Epi1 | red | 0.160289804 | 0.180966894 | 0.043682179 | 0.101146734 | 0.05681337 | 0.078043242 | 0.092965206 | 0.078333599 | -0.012385249 |
| SOCS2 | Epi1 | red | 0.133794535 | 0.20308735 | 0.099938844 | 0.069401552 | 0.031322168 | 0.063760893 | -0.003664332 | 0.1232598 | 0.011117283 |
| CORO1C | Epi1 | red | 0.077012731 | 0.141407093 | 0.020996317 | 0.037830683 | 0.012588736 | 0.041548239 | 0.006313334 | 0.048693775 | -0.023062585 |
| TMEM233 | Epi1 | red | 0.109835291 | 0.185541936 | 0.061677923 | 0.048814629 | 0.008880184 | 0.074177436 | -0.036227575 | 0.135933568 | -0.0345343 |
| CLDN10 | Epi1 | red | 0.101174275 | 0.361996124 | -0.031543316 | 0.0940015 | -0.008834704 | 0.021995688 | -0.054173928 | 0.108908923 | -0.111071678 |
| GAS6 | Epi1 | red | 0.079536486 | 0.171730551 | -0.02534649 | 0.098385645 | -0.023118293 | 0.004467523 | 0.077566415 | 0.030278679 | -0.028186864 |
| IFI27 | Epi1 | red | 0.130841582 | 0.303555423 | 0.048966673 | 0.081703297 | 0.01232067 | 0.03238627 | 0.040309606 | 0.05805754 | -0.010692406 |
| SERPINA1 | Epi1 | red | 0.253204504 | 0.494820339 | 0.170193968 | 0.298761208 | 0.010944997 | 0.076642973 | -0.048916965 | 0.355268134 | 0.036781034 |
| CRIP2 | Epi1 | red | 0.135257712 | 0.218201339 | 0.073291399 | 0.125994634 | 0.002382374 | 0.035696228 | -0.002445631 | 0.194725765 | -0.020140956 |
| GCHFR | Epi1 | red | 0.116510849 | 0.168286924 | 0.078074289 | 0.086285124 | 0.045737488 | 0.032477092 | -0.009620036 | 0.12459854 | 0.016329878 |
| B2M | Epi1 | red | 0.342001862 | 0.638348324 | 0.157944461 | 0.357715663 | 0.072617273 | 0.093664605 | 0.132840415 | 0.199805909 | 0.116424652 |
| RPS27L | Epi1 | red | 0.316347917 | 0.382889115 | 0.305016735 | 0.202056231 | 0.190534793 | 0.059881171 | -0.036500517 | 0.269263193 | 0.116519385 |
| SEMA4B | Epi1 | red | 0.103005282 | 0.159850568 | 0.057758808 | 0.049283228 | 0.020896421 | 0.04115472 | 0.006983745 | 0.089357149 | -0.002827639 |
| MCTP2 | Epi1 | red | 0.082451679 | 0.136459005 | 0.031074499 | 0.037461896 | 0.0093047 | 0.014515429 | 0.001802875 | 0.065620775 | -0.015896837 |
| CDIP1 | Epi1 | red | 0.045290555 | 0.081107793 | 0.025153311 | 0.06948196 | 0.003762823 | 0.009958609 | 0.03224504 | 0.063272191 | 0.010886267 |
| NUPR1 | Epi1 | red | 0.095305118 | 0.230121999 | -0.07637098 | 0.160215188 | -0.138419811 | 0.033417275 | 0.116886301 | 0.128142056 | -0.122995034 |
| CKLF | Epi1 | red | 0.175319471 | 0.294532856 | 0.15342983 | 0.172583912 | 0.058435923 | 0.040174665 | -0.050346113 | 0.215765609 | 0.058516715 |
| MAP1LC3B | Epi1 | red | 0.161587421 | 0.19629159 | 0.129046729 | 0.150388389 | 0.029182098 | 0.060271979 | 0.011198727 | 0.157819941 | 0.054326979 |
| SLC22A31 | Epi1 | red | 0.142311037 | 0.264608563 | 0.093386415 | 0.138630252 | 0.056842352 | 0.033807915 | 0.020005273 | 0.078391518 | 0.067603917 |
| TXNDC17 | Epi1 | red | 0.236326414 | 0.335035008 | 0.17593119 | 0.21778578 | 0.103592259 | 0.044117669 | 0.035468974 | 0.210476308 | 0.082129338 |
| TRAPPC1 | Epi1 | red | 0.198634295 | 0.181271684 | 0.169792743 | 0.225181674 | 0.104709005 | 0.076553041 | 0.06764586 | 0.157761977 | 0.142151331 |
| SREBF1 | Epi1 | red | 0.147868843 | 0.354503617 | 0.054500764 | 0.087556736 | 0.009575804 | 0.03441273 | -0.015617265 | 0.110039647 | -0.046189319 |
| KRT19 | Epi1 | red | 0.160197357 | 0.27806708 | 0.057856774 | 0.17512624 | 0.100937105 | 0.066188371 | 0.059055047 | 0.066508282 | 0.063696716 |
| ITGA3 | Epi1 | red | 0.109966748 | 0.19528727 | 0.06012203 | 0.05007052 | 0.028561511 | 0.046282026 | 0.00332499 | 0.087481757 | -0.009528015 |
| VMP1 | Epi1 | red | 0.138797917 | 0.309922184 | 0.032363179 | 0.074779197 | -0.011218672 | 0.089352334 | -0.014403718 | 0.11327481 | -0.058207141 |
| MRC2 | Epi1 | red | 0.078181765 | 0.113028444 | 0.063563161 | 0.052770616 | 0.0047064 | 0.051474055 | -0.002458453 | 0.115041376 | 0.003553782 |
| FAM20A | Epi1 | red | 0.124856946 | 0.245102715 | 0.06374559 | 0.055239032 | 0.034968728 | 0.031532383 | -0.020378428 | 0.073925637 | 0.007140509 |
| H3F3B | Epi1 | red | 0.312679931 | 0.283989361 | 0.175729608 | 0.198056491 | 0.16718399 | 0.092964453 | 0.122242531 | 0.145801435 | 0.091407076 |
| PMAIP1 | Epi1 | red | 0.144933023 | 0.35889851 | -0.010367642 | 0.026022666 | 0.04855487 | 0.044778314 | 0.019441181 | 0.042414726 | -0.102895632 |
| BCL2L1 | Epi1 | red | 0.132619763 | 0.202625029 | 0.105934065 | 0.040086975 | 0.071537754 | 0.025868866 | -0.022270721 | 0.110925839 | -0.002911674 |
| SLPI | Epi1 | red | 0.156048271 | 0.320077507 | 0.094736916 | 0.222284172 | 0.014177636 | 0.06196072 | -0.020949516 | 0.197209967 | 0.059730101 |
| TGFBR3L | Epi1 | red | 0.085559108 | 0.138713516 | 0.074064066 | 0.059581395 | -0.011732808 | 0.065569278 | -0.020429892 | 0.146961809 | -0.009544329 |
| ANGPTL4 | Epi1 | red | 0.113046886 | 0.35680637 | 0.03043325 | 0.129628627 | -0.025746709 | 0.043206588 | -0.026700933 | 0.120772334 | -0.02929762 |
| BST2 | Epi1 | red | 0.132956989 | 0.362190019 | 0.04888192 | 0.096285127 | -0.009810157 | 0.041615038 | 0.014700945 | 0.117701365 | -0.037653171 |
| ZFP36 | Epi1 | red | 0.202203626 | 0.276502449 | 0.110760368 | 0.147618309 | 0.033579857 | 0.065551214 | 0.003522991 | 0.191175844 | 0.021128409 |
| ETHE1 | Epi1 | red | 0.205311654 | 0.33550247 | 0.140466104 | 0.19805146 | 0.092946381 | 0.044822228 | 0.025657139 | 0.159854602 | 0.081383836 |
| KCNN4 | Epi1 | red | 0.132202943 | 0.271783889 | 0.115981658 | 0.131013416 | 0.00378254 | 0.063437496 | -0.064319029 | 0.18057879 | 0.024254031 |
| BAX | Epi1 | red | 0.154885419 | 0.215712771 | 0.145413274 | 0.117325846 | 0.112373848 | 0.034228726 | -0.024091395 | 0.133484799 | 0.057971754 |
| CHCHD10 | Epi1 | red | 0.105018827 | 0.111521633 | -0.00786137 | 0.160950485 | -0.032148106 | 0.020699609 | 0.168584737 | 0.065968183 | -0.013453919 |
| CABIN1 | Epi1 | red | 0.071878798 | 0.119264138 | 0.055235212 | 0.0466276 | 0.006680494 | 0.039647077 | -0.024131357 | 0.083127349 | 0.006105732 |
| GGT5 | Epi1 | red | 0.033996155 | 0.083239102 | -0.021605967 | 0.127035958 | -0.043282307 | -0.01472965 | 0.026969473 | 0.07004714 | 0.015411659 |
| TYMP | Epi1 | red | 0.154927759 | 0.218048997 | 0.133769677 | 0.12702031 | 0.086553048 | 0.026258229 | -0.002556461 | 0.139524175 | 0.056791759 |
| BTG3 | Epi1 | red | 0.122424869 | 0.238858697 | 0.028397909 | 0.142139912 | 0.021844826 | 0.045122068 | 0.027665254 | 0.061205963 | 0.017508045 |
| KCNJ15 | Epi1 | red | 0.139402005 | 0.198204178 | 0.089589774 | 0.133562315 | -0.00796675 | 0.072459601 | -0.003871297 | 0.151742915 | 0.011005487 |
| GNB1 | Epi2 | brown | 0.055618589 | 0.038532112 | 0.06782089 | 0.02587643 | 0.008607391 | 0.009045879 | 0.003238334 | 0.06246146 | 0.023566942 |
| CAMTA1 | Epi2 | brown | 0.184570107 | 0.023696779 | 0.26215726 | 0.088374291 | 0.192191712 | 0.06121812 | 0.040283849 | 0.125578845 | 0.181552435 |
| EFHD2 | Epi2 | brown | 0.133640986 | 0.05526173 | 0.170811423 | 0.067351649 | 0.093496377 | 0.043657749 | -0.032320448 | 0.150638318 | 0.097077877 |
| UBXN10-AS1 | Epi2 | brown | 0.061512373 | 0.032287593 | 0.125304434 | 0.021548503 | 0.015425926 | 0.024865604 | -0.052019952 | 0.142999533 | 0.003357089 |
| CAMK2N1 | Epi2 | brown | 0.247303917 | 0.184674521 | 0.340381461 | 0.042347456 | 0.103742491 | 0.103450572 | -0.137817095 | 0.354135455 | 0.036455975 |
| RCAN3 | Epi2 | brown | 0.049987208 | 0.01206756 | 0.057511556 | 0.020780903 | 0.036849962 | 0.01713309 | 0.003398121 | 0.03359488 | 0.020325973 |
| EVA1B | Epi2 | brown | 0.065417354 | 0.045964614 | 0.111335705 | 0.030115292 | 0.026805513 | 0.018408761 | -0.014883529 | 0.110701716 | 0.021952795 |
| LSM10 | Epi2 | brown | 0.089380271 | 0.072524117 | 0.132423758 | 0.040826116 | 0.042708241 | 0.05266457 | -0.032287702 | 0.124288435 | 0.039098886 |
| C1orf122 | Epi2 | brown | 0.168130718 | 0.093939 | 0.196281289 | 0.10561263 | 0.09561465 | 0.056079571 | 0.006922356 | 0.17438997 | 0.08612236 |
| RNF11 | Epi2 | brown | 0.150196326 | 0.066920278 | 0.157935639 | 0.07207557 | 0.099558739 | 0.050391272 | 0.041257025 | 0.102236894 | 0.076780823 |
| RP11-386I14.4 | Epi2 | brown | 0.09328636 | 0.037238293 | 0.123169751 | -0.004824057 | 0.079666466 | 0.02896218 | -0.025874988 | 0.067518898 | 0.026254577 |
| VAV3 | Epi2 | brown | 0.01606067 | -0.011083734 | 0.062627737 | 0.002217302 | 0.020180995 | -0.018538793 | -0.036348594 | 0.05304995 | 0.009401434 |
| RHOC | Epi2 | brown | 0.200173086 | 0.156846352 | 0.232877769 | 0.161164787 | 0.182291618 | 0.086117825 | 0.011626059 | 0.155025701 | 0.155245194 |
| PDE4DIP | Epi2 | brown | 0.067851785 | 0.054145113 | 0.087573356 | 0.020762517 | 0.025100318 | 0.01600998 | -0.018862027 | 0.073777425 | 0.005233239 |
| CH17-189H20.1 | Epi2 | brown | 0.117813145 | 0.066708119 | 0.13216191 | 0.064158445 | 0.080027012 | 0.048124914 | 0.01257997 | 0.125227683 | 0.067122295 |
| RPRD2 | Epi2 | brown | 0.072203559 | 0.023230376 | 0.100406811 | 0.039661294 | 0.049324852 | 0.041109837 | -0.023019845 | 0.073322201 | 0.066265512 |
| S100A1 | Epi2 | brown | 0.128486205 | -0.01699933 | 0.196349622 | 0.174789647 | 0.012017904 | 0.013288117 | 0.03435664 | 0.232178108 | 0.129302831 |
| PTPN14 | Epi2 | brown | 0.035850535 | -0.001970235 | 0.057176816 | -0.003477532 | 0.027971129 | 0.054682948 | -0.007524268 | 0.040994626 | 0.005398565 |
| ENAH | Epi2 | brown | 0.105820594 | 0.055320639 | 0.123956186 | 0.00805679 | 0.038367822 | 0.043792758 | -0.008663273 | 0.110108369 | 0.025997853 |
| SRP9 | Epi2 | brown | 0.258303742 | 0.096199402 | 0.288286025 | 0.208481641 | 0.176360749 | 0.07588585 | 0.067551783 | 0.226600629 | 0.196577697 |
| GUK1 | Epi2 | brown | 0.272778735 | 0.153850444 | 0.325446322 | 0.121139232 | 0.229296479 | 0.077941234 | 0.030164877 | 0.206452207 | 0.154622289 |
| RAB4A | Epi2 | brown | 0.131305497 | 0.066425013 | 0.153244295 | 0.059075169 | 0.132540962 | 0.058762369 | 0.029604945 | 0.091197143 | 0.079729049 |
| COX20 | Epi2 | brown | 0.174064227 | 0.042872392 | 0.243999334 | 0.073520946 | 0.150602949 | 0.042423181 | 0.028266837 | 0.133657906 | 0.124183366 |
| HPCAL1 | Epi2 | brown | 0.151294618 | 0.069027802 | 0.18384977 | 0.073284481 | 0.101393211 | 0.050331772 | 0.009475144 | 0.159768573 | 0.077092173 |
| ODC1 | Epi2 | brown | 0.145579165 | 0.009506036 | 0.274072269 | 0.026263796 | 0.11478098 | 0.04053077 | -0.066696107 | 0.185216836 | 0.121675646 |
| NBAS | Epi2 | brown | 0.081611315 | 0.052055679 | 0.119810244 | 0.045255585 | 0.04310221 | 0.037541962 | -0.037023546 | 0.075218029 | 0.059210169 |
| OST4 | Epi2 | brown | 0.355794563 | 0.116186559 | 0.448444499 | 0.219480278 | 0.34970271 | 0.121110342 | 0.102594469 | 0.246840626 | 0.31876993 |
| LBH | Epi2 | brown | 0.071697608 | -0.027356935 | 0.10745842 | 0.040179251 | 0.042412014 | 0.013855915 | 0.023188019 | 0.067405371 | 0.076142383 |
| LCLAT1 | Epi2 | brown | 0.047927583 | 0.03750059 | 0.071081853 | 0.008433028 | 0.02546081 | 0.023121612 | -0.020015814 | 0.057363063 | 0.008230971 |
| LTBP1 | Epi2 | brown | 0.073060738 | 0.007380294 | 0.098658089 | -0.003200449 | 0.064758668 | 0.019397257 | -0.005179609 | 0.008512594 | 0.050996454 |
| PCBP1 | Epi2 | brown | 0.195002715 | 0.086020354 | 0.210670196 | 0.051491704 | 0.128422019 | 0.064453482 | 0.032823332 | 0.12385702 | 0.097236762 |
| SNRPG | Epi2 | brown | 0.291942025 | 0.161662409 | 0.360781947 | 0.22053819 | 0.2082178 | 0.090862802 | 0.056027085 | 0.233647496 | 0.229985168 |
| TGFA | Epi2 | brown | 0.119991573 | 0.063330033 | 0.18169249 | 0.031347817 | 0.08255684 | 0.024643375 | -0.047112371 | 0.127689584 | 0.060847617 |
| DGUOK-AS1 | Epi2 | brown | 0.052219103 | -0.00949844 | 0.124471409 | 0.001398953 | 0.044813318 | 0.02637046 | -0.043982149 | 0.077846106 | 0.030153861 |
| HK2 | Epi2 | brown | 0.071875885 | 0.006696207 | 0.150748998 | 0.002705671 | 0.038426725 | 0.043799895 | -0.015289463 | 0.079009093 | 0.05963036 |
| POLE4 | Epi2 | brown | 0.142273738 | 0.052602279 | 0.216887063 | 0.090200944 | 0.10111054 | 0.04660916 | -0.042220069 | 0.158769513 | 0.123569734 |
| EIF5B | Epi2 | brown | 0.205920969 | 0.089174144 | 0.222569679 | 0.112446054 | 0.119264267 | 0.051601845 | 0.019274628 | 0.152093901 | 0.110335236 |
| LONRF2 | Epi2 | brown | 0.048182212 | -0.030256804 | 0.104166999 | 0.004900746 | 0.063688903 | 0.033537321 | 0.011103143 | 0.023718641 | 0.062171106 |
| MAP4K4 | Epi2 | brown | 0.082589932 | 0.058810152 | 0.082215976 | 0.025283879 | 0.069301399 | 0.02633516 | -0.010070603 | 0.044357593 | 0.037282209 |
| FMNL2 | Epi2 | brown | 0.113628414 | 0.068916767 | 0.152825603 | 0.039655723 | 0.028826084 | 0.038351377 | -0.052628714 | 0.162744862 | 0.042220929 |
| NCKAP1 | Epi2 | brown | 0.116968251 | 0.059280504 | 0.122354091 | 0.043758083 | 0.06524906 | 0.054232661 | 0.005125834 | 0.091852841 | 0.054944616 |
| SPATS2L | Epi2 | brown | 0.221182988 | 0.116400096 | 0.243182023 | 0.107872859 | 0.190387339 | 0.053248108 | 0.070280816 | 0.097698382 | 0.180514648 |
| EPHA4 | Epi2 | brown | 0.1129842 | 0.060088354 | 0.148601464 | 0.074319763 | 0.071071673 | 0.037639784 | 0.003465711 | 0.094357288 | 0.093803805 |
| CAB39 | Epi2 | brown | 0.102385138 | 0.038701682 | 0.172985175 | 0.038692361 | 0.059714825 | 0.019193311 | -0.032893897 | 0.122924866 | 0.050309545 |
| TADA3 | Epi2 | brown | 0.107090827 | 0.034381387 | 0.138970624 | 0.046429345 | 0.088861805 | 0.041764673 | 0.00714128 | 0.106681874 | 0.068351042 |
| BRK1 | Epi2 | brown | 0.232311197 | 0.072645421 | 0.2621266 | 0.177554411 | 0.233912455 | 0.09629183 | 0.094757267 | 0.153383692 | 0.208269735 |
| OXNAD1 | Epi2 | brown | 0.048289352 | -0.00800979 | 0.114837705 | 0.02455798 | 0.025788021 | 0.014858706 | -0.013869129 | 0.113103048 | 0.045018311 |
| KAT2B | Epi2 | brown | 0.084922914 | 0.027187917 | 0.133512552 | 0.042450721 | 0.059478892 | 0.007581045 | -0.029532073 | 0.116951548 | 0.059249877 |
| GPX1 | Epi2 | brown | 0.344189609 | 0.210889741 | 0.432178846 | 0.260922371 | 0.258001125 | 0.093717734 | -0.017780894 | 0.365344134 | 0.24818286 |
| SEMA3F | Epi2 | brown | 0.051410458 | -0.005289845 | 0.090468234 | 0.01292517 | 0.019623464 | 0.027650039 | -0.002360556 | 0.072595916 | 0.025173144 |
| SMIM4 | Epi2 | brown | 0.234195034 | 0.016147154 | 0.386801623 | 0.172335276 | 0.218255864 | 0.080024065 | 0.015322434 | 0.227230058 | 0.274121048 |
| PROS1 | Epi2 | brown | 0.134924016 | 0.083374235 | 0.191122158 | 0.108755323 | 0.075565477 | 0.043832946 | -0.030151633 | 0.136303809 | 0.119141782 |
| PVRL3 | Epi2 | brown | 0.078987182 | 0.006572935 | 0.094068826 | 0.043802387 | 0.048093345 | 0.022855089 | 0.002807196 | 0.065369662 | 0.054539048 |
| CCDC80 | Epi2 | brown | 0.265107418 | 0.024120981 | 0.390682058 | 0.105956987 | 0.201098706 | 0.094582348 | -0.024719341 | 0.251814327 | 0.233114949 |
| USF3 | Epi2 | brown | 0.040707795 | 0.002278219 | 0.066348368 | 0.015322093 | 0.039987351 | 0.031097079 | -0.003865523 | 0.043987667 | 0.021055203 |
| ATP6V1A | Epi2 | brown | 0.096465114 | 0.01892994 | 0.129118652 | 0.060953235 | 0.070494329 | 0.035337715 | 0.015056571 | 0.076433823 | 0.085896872 |
| COX17 | Epi2 | brown | 0.204139025 | 0.133261088 | 0.260764198 | 0.104268155 | 0.132058933 | 0.069628352 | 0.007477646 | 0.16757186 | 0.116827685 |
| ITGB5 | Epi2 | brown | 0.078572314 | -0.019007994 | 0.160875403 | 0.028408656 | 0.092818621 | 0.007295907 | -0.010819441 | 0.067636271 | 0.119327383 |
| TMEM108 | Epi2 | brown | 0.090599228 | 0.013200645 | 0.148569311 | 0.053145166 | 0.088226317 | 0.008699959 | 0.000347896 | 0.068940448 | 0.102772102 |
| PXYLP1 | Epi2 | brown | 0.042716568 | 0.010914402 | 0.082261132 | 0.003998289 | 0.02026857 | 0.021040128 | -0.035486772 | 0.074627795 | 0.012222665 |
| SERP1 | Epi2 | brown | 0.197894175 | 0.098215872 | 0.240284355 | 0.098138758 | 0.181101793 | 0.037436515 | 0.039414413 | 0.143939557 | 0.126649019 |
| ZMAT3 | Epi2 | brown | 0.142511683 | 0.06567411 | 0.198068632 | 0.038968132 | 0.123162271 | 0.067106415 | -0.01577991 | 0.11192364 | 0.085667942 |
| CLDN1 | Epi2 | brown | 0.167842702 | 0.087218806 | 0.23998273 | 0.101184116 | 0.103868961 | 0.055982253 | -0.02855606 | 0.147872627 | 0.155505246 |
| IL1RAP | Epi2 | brown | 0.106078073 | 0.030431435 | 0.144695129 | 0.020016469 | 0.071947978 | 0.04610136 | -0.022457105 | 0.08109894 | 0.083155071 |
| HES1 | Epi2 | brown | 0.123032336 | 0.022123943 | 0.166948638 | 0.08464933 | 0.078904054 | 0.040982854 | 0.012465146 | 0.126736389 | 0.10123155 |
| RP11-513G11.4 | Epi2 | brown | 0.068142655 | 0.014285323 | 0.123906651 | 0.013776422 | 0.063645994 | 0.026537307 | -0.009109355 | 0.058294126 | 0.068843943 |
| ACAP2 | Epi2 | brown | 0.086180625 | 0.043514722 | 0.099972535 | 0.018037275 | 0.060351647 | 0.062109194 | 0.008409318 | 0.085968134 | 0.031625932 |
| ATP5I | Epi2 | brown | 0.275760128 | 0.088109468 | 0.357150642 | 0.228365865 | 0.260633844 | 0.076225545 | 0.089915057 | 0.168591542 | 0.298687893 |
| C4orf48 | Epi2 | brown | 0.351129272 | 0.172807834 | 0.524413083 | 0.203528667 | 0.239210801 | 0.107294422 | -0.069930794 | 0.383164892 | 0.260790085 |
| MXD4 | Epi2 | brown | 0.063768057 | -0.024912935 | 0.1293676 | 0.005547163 | 0.061446112 | 0.012118435 | 0.000373821 | 0.072110425 | 0.070584824 |
| DEFB131 | Epi2 | brown | 0.033279392 | -0.013414945 | 0.092615732 | 0.021642178 | -0.005124208 | 0.027381132 | -0.037549711 | 0.102294261 | 0.007317335 |
| PCDH7 | Epi2 | brown | 0.097619976 | 0.053345051 | 0.128683016 | 0.032294287 | 0.027455373 | 0.02586133 | -0.046124181 | 0.123340271 | 0.021683026 |
| UBE2K | Epi2 | brown | 0.091562826 | 0.046890335 | 0.099607708 | 0.039458461 | 0.073101753 | 0.04363951 | 0.041836709 | 0.040291171 | 0.059861748 |
| MTHFD2L | Epi2 | brown | 0.105440478 | 0.06227991 | 0.13738241 | 0.062674835 | 0.039912005 | 0.038551102 | -0.005989591 | 0.138844406 | 0.043117481 |
| PTPN13 | Epi2 | brown | 0.063446969 | -0.021110701 | 0.107715824 | 0.013830715 | 0.064993651 | 0.050286461 | -0.003920419 | 0.055469096 | 0.053636931 |
| PYURF | Epi2 | brown | 0.228860812 | 0.061621362 | 0.237067506 | 0.162253608 | 0.187951792 | 0.050879708 | 0.090569245 | 0.148652382 | 0.184482293 |
| C4orf3 | Epi2 | brown | 0.249666301 | 0.154575724 | 0.25597661 | 0.120264937 | 0.196111542 | 0.093000977 | 0.072541511 | 0.148065317 | 0.131688327 |
| SPRY1 | Epi2 | brown | 0.103177924 | 0.029570904 | 0.155014126 | 0.101636772 | 0.074845972 | 0.055774483 | -0.039314827 | 0.135047276 | 0.108147886 |
| TMEM154 | Epi2 | brown | 0.025412727 | 0.028210672 | 0.085188409 | 0.022732594 | -0.007612686 | 0.01486154 | -0.049226191 | 0.083913859 | 0.01564403 |
| CFAP97 | Epi2 | brown | 0.136759434 | 0.039511386 | 0.169176756 | 0.063715659 | 0.099363585 | 0.028403763 | 0.003500555 | 0.089968936 | 0.09488403 |
| DAP | Epi2 | brown | 0.127096497 | 0.031875002 | 0.135289495 | 0.055471268 | 0.092860576 | 0.026770509 | 0.024707972 | 0.090804649 | 0.090759915 |
| PTGER4 | Epi2 | brown | 0.048133004 | 0.02175919 | 0.084795166 | 0.031572118 | 0.025446266 | 0.022706771 | -0.027789415 | 0.083809202 | 0.040273538 |
| IL6ST | Epi2 | brown | 0.165187694 | 0.05487696 | 0.212865853 | 0.050757125 | 0.111812143 | 0.065541581 | 0.010371287 | 0.134588014 | 0.097483584 |
| MAP3K1 | Epi2 | brown | 0.110632462 | 0.070341217 | 0.12569076 | 0.032902341 | 0.077600284 | 0.052343268 | 0.015139261 | 0.078004376 | 0.036023771 |
| RHOBTB3 | Epi2 | brown | 0.10106258 | -0.01693718 | 0.115945102 | 0.033449123 | 0.068189744 | 0.035618991 | 0.045685149 | 0.080547654 | 0.074074737 |
| TRIM36 | Epi2 | brown | 0.087348165 | -0.088334502 | 0.17005609 | 0.037341471 | 0.08729944 | 0.021724336 | 0.046674476 | 0.073885462 | 0.138703854 |
| HINT1 | Epi2 | brown | 0.364300477 | 0.087405501 | 0.459992901 | 0.287876969 | 0.402891125 | 0.106393589 | 0.088609682 | 0.264711434 | 0.357280865 |
| UQCRQ | Epi2 | brown | 0.30380087 | 0.190540415 | 0.346524299 | 0.252995255 | 0.213269497 | 0.066259154 | 0.071002947 | 0.231726975 | 0.241933193 |
| ADRB2 | Epi2 | brown | 0.059265773 | 0.048234864 | 0.073493817 | 0.014592522 | 0.018784572 | 0.02958245 | -0.011589634 | 0.035283012 | 0.040552475 |
| SLIT3 | Epi2 | brown | 0.045966772 | 0.005581363 | 0.095195654 | 0.015843907 | 0.006070653 | 0.033127262 | -0.026662737 | 0.092743285 | 0.028023224 |
| KCNMB1 | Epi2 | brown | 0.011264326 | -0.026445623 | 0.076775733 | 0.010103254 | -0.011921506 | 0.018239619 | -0.032694766 | 0.079026968 | 0.02197398 |
| PRR7 | Epi2 | brown | 0.047007564 | 0.050897489 | 0.048277468 | 0.016018637 | 0.019214434 | 0.018391445 | -0.028540268 | 0.058997325 | 0.007804667 |
| DDAH2 | Epi2 | brown | 0.161172985 | 0.037371767 | 0.196315715 | 0.096910882 | 0.140977577 | 0.042350981 | 0.043152645 | 0.094960622 | 0.137062922 |
| MAPK14 | Epi2 | brown | 0.07211116 | 0.040073909 | 0.094752019 | 0.038001719 | 0.040052875 | 0.036034871 | -0.004567728 | 0.070535082 | 0.045428101 |
| SLC17A5 | Epi2 | brown | 0.126858628 | 0.04138065 | 0.145942048 | 0.103106932 | 0.072199537 | 0.049077174 | 0.054981138 | 0.087665106 | 0.126796702 |
| TMEM30A | Epi2 | brown | 0.131867693 | 0.087379661 | 0.161108608 | 0.078165141 | 0.092162802 | 0.042038695 | -0.013216197 | 0.120486023 | 0.08405989 |
| POPDC3 | Epi2 | brown | 0.047715815 | -0.059817487 | 0.157127072 | -0.001567933 | 0.048557893 | 0.017084361 | -0.038478535 | 0.087872676 | 0.082506557 |
| GTF3C6 | Epi2 | brown | 0.175582285 | 0.074953089 | 0.19742716 | 0.112712384 | 0.144386282 | 0.06298204 | 0.029587821 | 0.107468593 | 0.130092617 |
| TPD52L1 | Epi2 | brown | 0.187519306 | 0.072081571 | 0.291654127 | 0.121149584 | 0.125239332 | 0.066311182 | -0.071478864 | 0.260314841 | 0.143432881 |
| ARHGAP18 | Epi2 | brown | 0.127774367 | 0.036053741 | 0.194893669 | 0.076404837 | 0.102899927 | 0.077345264 | -0.012240367 | 0.134215574 | 0.109093147 |
| RAC1 | Epi2 | brown | 0.301234729 | 0.156896177 | 0.302286806 | 0.229712851 | 0.205560124 | 0.119652783 | 0.085293606 | 0.183718079 | 0.212447395 |
| ETV1 | Epi2 | brown | 0.10646445 | -0.012410501 | 0.164614895 | 0.045361667 | 0.116443541 | 0.016489049 | -0.001666498 | 0.078687286 | 0.121795564 |
| TAX1BP1 | Epi2 | brown | 0.157325031 | 0.037592043 | 0.165386824 | 0.135788336 | 0.109331177 | 0.05436119 | 0.061478576 | 0.099116949 | 0.135459997 |
| CREB5 | Epi2 | brown | 0.094241678 | 0.070751982 | 0.102534009 | 0.019171902 | 0.066942718 | 0.030258966 | -0.023403225 | 0.053508296 | 0.034979831 |
| PRR15 | Epi2 | brown | 0.173140576 | -0.002255178 | 0.315037311 | 0.060462378 | 0.161853474 | 0.047971543 | -0.051217179 | 0.177862173 | 0.166848059 |
| DPY19L1 | Epi2 | brown | 0.094741611 | 0.048677162 | 0.136167624 | 0.033176074 | 0.071004548 | 0.043686706 | 0.016945753 | 0.062045853 | 0.057852647 |
| UPP1 | Epi2 | brown | 0.13943849 | 0.029313864 | 0.201192306 | 0.064483042 | 0.122758042 | 0.056740316 | 0.014855728 | 0.107692715 | 0.115385243 |
| TMEM248 | Epi2 | brown | 0.099554238 | 0.045842678 | 0.10396157 | 0.046324617 | 0.079822825 | 0.045833066 | 0.040517197 | 0.061876935 | 0.057395258 |
| HIP1 | Epi2 | brown | 0.1075521 | 0.076706689 | 0.159379829 | 0.035524237 | 0.048916262 | 0.038571281 | -0.034943126 | 0.096882944 | 0.052101592 |
| TMEM60 | Epi2 | brown | 0.084783184 | 0.019271133 | 0.11191036 | 0.053094505 | 0.076699047 | 0.03459972 | -0.018090474 | 0.076480431 | 0.071300708 |
| TMEM243 | Epi2 | brown | 0.242033169 | 0.061202267 | 0.342176862 | 0.104916431 | 0.192058926 | 0.058587396 | 0.009053313 | 0.18197914 | 0.192617883 |
| ARPC1B | Epi2 | brown | 0.174570537 | 0.119975228 | 0.195683116 | 0.128923102 | 0.118002308 | 0.048247912 | 0.047277993 | 0.117372206 | 0.134172903 |
| LAMTOR4 | Epi2 | brown | 0.185786009 | 0.017397686 | 0.300649528 | 0.180584035 | 0.172504946 | 0.037039034 | 0.056902302 | 0.183374496 | 0.23672906 |
| ZNHIT1 | Epi2 | brown | 0.189523069 | 0.073912326 | 0.238236635 | 0.137777528 | 0.142681781 | 0.047916273 | 0.050976282 | 0.151359019 | 0.144482226 |
| UBE2H | Epi2 | brown | 0.11745824 | 0.025763351 | 0.166884488 | 0.049074065 | 0.06717125 | 0.061827656 | -0.006264204 | 0.119726982 | 0.056205002 |
| MTPN | Epi2 | brown | 0.152078007 | 0.026231946 | 0.175803701 | 0.081932201 | 0.134689433 | 0.055300539 | 0.054966897 | 0.077267222 | 0.122434828 |
| AC090498.1 | Epi2 | brown | 0.092889681 | 0.024329821 | 0.161376601 | 0.006494834 | 0.164077623 | 0.033940588 | 0.004143666 | 0.06926749 | 0.081393657 |
| HIPK2 | Epi2 | brown | 0.084544571 | 0.010190539 | 0.138537138 | 0.022861951 | 0.0532625 | 0.039845862 | -0.009485243 | 0.068172659 | 0.070343676 |
| AC093673.5 | Epi2 | brown | 0.076061855 | 0.04372329 | 0.092230632 | 0.051971655 | 0.05025391 | 0.034454072 | 0.001564216 | 0.068176038 | 0.066159342 |
| RHEB | Epi2 | brown | 0.227424545 | 0.086555766 | 0.245046381 | 0.169546642 | 0.149757012 | 0.067946924 | 0.079950279 | 0.167317055 | 0.156708996 |
| PHEX | Epi2 | brown | 0.118862963 | -0.051888223 | 0.204320648 | 0.052158525 | 0.101884572 | 0.065731933 | -0.003500269 | 0.110890365 | 0.147137731 |
| DMD | Epi2 | brown | 0.095888547 | -0.041293223 | 0.198327055 | 0.052947949 | 0.092105821 | 0.03424937 | -0.025413321 | 0.109518488 | 0.123585352 |
| MAOA | Epi2 | brown | 0.132937961 | -0.038656781 | 0.276137883 | 0.116041615 | 0.085925564 | 0.04275494 | -0.029974905 | 0.224177587 | 0.165794923 |
| MIR222HG | Epi2 | brown | 0.045265336 | -0.030052877 | 0.125645095 | 0.038536711 | 0.073644842 | 0.008811571 | -0.004406004 | 0.068647692 | 0.091236048 |
| MSN | Epi2 | brown | 0.118497549 | 0.064538416 | 0.137748793 | 0.047242314 | 0.069747798 | 0.011792993 | -0.013448316 | 0.110196087 | 0.052428538 |
| AR | Epi2 | brown | 0.043956522 | 0.015943442 | 0.09178912 | 0.034254141 | -0.011792083 | 0.011474901 | -0.045142445 | 0.122553489 | 0.001377484 |
| ARMCX3 | Epi2 | brown | 0.131814065 | 0.047984445 | 0.194119137 | 0.09768999 | 0.099810628 | 0.042308092 | -0.008768065 | 0.13938205 | 0.118764901 |
| PAK3 | Epi2 | brown | 0.029589702 | -0.019471967 | 0.071197403 | -0.005044688 | 0.029879363 | -0.011299869 | -0.019125228 | 0.029526429 | 0.039787364 |
| ZCCHC12 | Epi2 | brown | 0.134711777 | -0.054574364 | 0.288557134 | 0.220077585 | 0.096164781 | 0.040836863 | -0.058246672 | 0.269357394 | 0.255634739 |
| TMEM255A | Epi2 | brown | 0.062600159 | 0.027184169 | 0.141224141 | 0.046136273 | 0.043761375 | 0.006458365 | -0.046754759 | 0.089708298 | 0.095966556 |
| IGSF1 | Epi2 | brown | 0.103744017 | -0.055816121 | 0.238543637 | 0.153871625 | 0.115187362 | 0.009822083 | 0.012815847 | 0.122740171 | 0.258023282 |
| SLC7A2 | Epi2 | brown | 0.09456283 | -0.027457376 | 0.211789475 | 0.042038668 | 0.092555824 | 0.03002628 | -0.030619217 | 0.131919675 | 0.134614335 |
| MTUS1 | Epi2 | brown | 0.165439171 | 0.094878065 | 0.198746498 | 0.073566734 | 0.105717479 | 0.053922982 | -0.026969446 | 0.17317029 | 0.072566638 |
| PAG1 | Epi2 | brown | 0.111030523 | 0.05333537 | 0.143035783 | 0.03792351 | 0.096088382 | 0.034726987 | -0.010032643 | 0.095268484 | 0.07333472 |
| UQCRB | Epi2 | brown | 0.300231234 | 0.031071914 | 0.46811753 | 0.217574198 | 0.341317717 | 0.049407524 | 0.048057729 | 0.252015578 | 0.342228663 |
| YWHAZ | Epi2 | brown | 0.211617522 | 0.08524714 | 0.247671101 | 0.086199738 | 0.180534456 | 0.099707421 | 0.057169811 | 0.111992769 | 0.158360383 |
| DERL1 | Epi2 | brown | 0.106481892 | -0.00842562 | 0.13805796 | 0.082707554 | 0.084438917 | 0.05991657 | 0.023293711 | 0.058959871 | 0.114453695 |
| RP11-195F19.5 | Epi2 | brown | 0.07557745 | 0.024632916 | 0.131508397 | 0.068117486 | 0.056813431 | 0.041329522 | -0.003410132 | 0.105004252 | 0.050309438 |
| RGP1 | Epi2 | brown | 0.03069377 | -0.012626845 | 0.051116253 | -0.005572826 | 0.036038443 | 0.031147066 | -0.016845649 | 0.048623091 | 0.010326137 |
| RFK | Epi2 | brown | 0.127496286 | 0.073077549 | 0.134069595 | 0.082813454 | 0.075770689 | 0.046891735 | 0.007404709 | 0.111548742 | 0.085902625 |
| NTRK2 | Epi2 | brown | 0.250773305 | 0.120073266 | 0.310555072 | 0.128694664 | 0.147024254 | 0.090630187 | 0.007062946 | 0.203419758 | 0.181237898 |
| C9orf3 | Epi2 | brown | 0.071088763 | -0.012971423 | 0.148894683 | 0.045307068 | 0.08950162 | 0.036674793 | -0.025325723 | 0.075251347 | 0.095808189 |
| RAD23B | Epi2 | brown | 0.122406201 | -0.000559509 | 0.168569731 | 0.028930504 | 0.134404806 | 0.027115653 | 0.02352883 | 0.051399118 | 0.127531671 |
| PPP2R4 | Epi2 | brown | 0.0839655 | 0.036284325 | 0.091979783 | 0.039263983 | 0.061565376 | 0.025841195 | 0.016867235 | 0.057179224 | 0.048122049 |
| POLR2L | Epi2 | brown | 0.350656202 | 0.112081415 | 0.461830162 | 0.301044254 | 0.297023357 | 0.100233813 | 0.120345643 | 0.281961754 | 0.351685852 |
| SVIP | Epi2 | brown | 0.17063663 | 0.049877157 | 0.20371257 | 0.068699541 | 0.129187447 | 0.045846143 | 0.022099786 | 0.138167324 | 0.107515248 |
| LRP4 | Epi2 | brown | 0.109802256 | 0.074947008 | 0.160283613 | 0.05019491 | 0.071608364 | 0.05363301 | -0.0445876 | 0.115822559 | 0.063825315 |
| CCDC85B | Epi2 | brown | 0.181659608 | 0.065166688 | 0.228500022 | 0.071081922 | 0.185624293 | 0.058603901 | 0.031994893 | 0.095892619 | 0.131957402 |
| PRSS23 | Epi2 | brown | 0.331691881 | 0.240051953 | 0.44588666 | 0.30520448 | 0.245016525 | 0.074398039 | -0.037841723 | 0.271093914 | 0.346172041 |
| SESN3 | Epi2 | brown | 0.142482105 | 0.092313546 | 0.182513973 | 0.057322347 | 0.067181643 | 0.061363451 | -0.007411824 | 0.147207389 | 0.051148034 |
| YAP1 | Epi2 | brown | 0.106463311 | 0.040281391 | 0.120128666 | 0.038393463 | 0.069471484 | 0.04184882 | 0.019769811 | 0.058170227 | 0.06349863 |
| H2AFX | Epi2 | brown | 0.107528035 | 0.05233369 | 0.172468668 | 0.07743657 | 0.041375919 | 0.03415039 | -0.02269775 | 0.138083135 | 0.075137773 |
| RBM17 | Epi2 | brown | 0.121103681 | 0.066921521 | 0.129586001 | 0.071787895 | 0.036902399 | 0.026812332 | -0.007444737 | 0.164664558 | 0.03170707 |
| OTUD1 | Epi2 | brown | 0.053915826 | -0.005533186 | 0.096744755 | -0.001309498 | 0.046386708 | 0.001872089 | 0.007801867 | 0.054517732 | 0.026142955 |
| LINC00844 | Epi2 | brown | 0.035923263 | -0.067116873 | 0.141074803 | 0.023881493 | 0.00586402 | 0.02422448 | -0.060139909 | 0.135117814 | 0.065456299 |
| USMG5 | Epi2 | brown | 0.311088476 | 0.131038886 | 0.393732537 | 0.254530106 | 0.276922765 | 0.098299602 | 0.091377729 | 0.22636964 | 0.302715492 |
| CCND2 | Epi2 | brown | 0.206118399 | 0.056471628 | 0.278299059 | 0.080501892 | 0.162771348 | 0.103857633 | 0.037885995 | 0.155840986 | 0.161780283 |
| CD27 | Epi2 | brown | 0.027649978 | -0.054969827 | 0.083842463 | 0.039068545 | 0.024428972 | -0.001704438 | -0.012953455 | 0.067089215 | 0.080723979 |
| CHD4 | Epi2 | brown | 0.110927538 | 0.065374348 | 0.099443958 | 0.035807164 | 0.062452506 | 0.045342051 | 0.018189609 | 0.066313805 | 0.054540312 |
| LPAR5 | Epi2 | brown | 0.106936876 | -0.001802361 | 0.168786389 | 0.074184715 | 0.094375231 | 0.053224774 | 0.015446391 | 0.083827636 | 0.133457856 |
| ARHGDIB | Epi2 | brown | 0.167134018 | 0.008258911 | 0.262174278 | 0.193638636 | 0.141979061 | 0.054160888 | 0.013861232 | 0.169858591 | 0.24162435 |
| EPS8 | Epi2 | brown | 0.110746022 | 0.051653357 | 0.128394253 | 0.031780231 | 0.075011698 | 0.057437402 | -0.020567074 | 0.08381887 | 0.058194429 |
| LMO3 | Epi2 | brown | 0.193324671 | 0.087452523 | 0.22427256 | 0.087009438 | 0.161241484 | 0.08734001 | 0.012205884 | 0.120243376 | 0.131806727 |
| LRRK2 | Epi2 | brown | 0.188429664 | 0.08226595 | 0.245982009 | 0.042833275 | 0.146560162 | 0.060966151 | -0.038341765 | 0.155134754 | 0.110103471 |
| PRICKLE1 | Epi2 | brown | 0.105765235 | 0.046197539 | 0.13255149 | 0.007372672 | 0.078711701 | 0.051498536 | -0.024450381 | 0.066665821 | 0.058830738 |
| SLC38A1 | Epi2 | brown | 0.100011287 | 0.058506721 | 0.116401683 | 0.033239104 | 0.035846078 | 0.015904388 | -0.009682724 | 0.10366073 | 0.030403068 |
| RPS26 | Epi2 | brown | 0.355580629 | 0.193817791 | 0.332210364 | 0.255170166 | 0.268209387 | 0.108824604 | 0.163575944 | 0.186578082 | 0.246845207 |
| TMEM5 | Epi2 | brown | 0.068376823 | 0.016670777 | 0.08916285 | 0.037926592 | 0.038169884 | 0.033802975 | 0.002235268 | 0.075636024 | 0.034984156 |
| XPOT | Epi2 | brown | 0.054692148 | 0.030160507 | 0.064200716 | 0.024816378 | 0.020986762 | 0.028650041 | 0.007786785 | 0.06190769 | 0.029062192 |
| CHPT1 | Epi2 | brown | 0.102554691 | 0.029690467 | 0.153895868 | 0.067385924 | 0.045979146 | 0.032080717 | -0.027032138 | 0.135837472 | 0.089882461 |
| ZDHHC20 | Epi2 | brown | 0.076619536 | 0.035948068 | 0.102544847 | 0.026641324 | 0.044019186 | 0.019327736 | -0.004041451 | 0.030876021 | 0.061033184 |
| POMP | Epi2 | brown | 0.250556725 | 0.126626724 | 0.330003993 | 0.275631666 | 0.15639249 | 0.040765973 | 0.064557998 | 0.228082925 | 0.239985674 |
| SERP2 | Epi2 | brown | 0.066978759 | 0.043043326 | 0.077531443 | 0.034062546 | 0.018573105 | 0.00744356 | -0.026549104 | 0.090853509 | 0.018732616 |
| KCTD12 | Epi2 | brown | 0.231688606 | 0.219814153 | 0.325158788 | 0.091609534 | 0.095956761 | 0.064096233 | -0.07721104 | 0.247715987 | 0.115539885 |
| SPRY2 | Epi2 | brown | 0.080160445 | 0.017598102 | 0.127585279 | -0.002231511 | 0.037817084 | 0.045685525 | -0.022071125 | 0.109304409 | 0.030795117 |
| NEDD8 | Epi2 | brown | 0.23068598 | 0.135688376 | 0.254277584 | 0.158169119 | 0.187462087 | 0.053648555 | 0.075034322 | 0.166004561 | 0.156294009 |
| PAX9 | Epi2 | brown | 0.111855797 | 0.023051663 | 0.188919599 | 0.048631758 | 0.125797523 | 0.019293912 | -0.027659941 | 0.054469179 | 0.131089555 |
| SAV1 | Epi2 | brown | 0.106755804 | 0.02843553 | 0.105587194 | 0.037101403 | 0.066355681 | 0.035183885 | 0.013660993 | 0.085496172 | 0.03816881 |
| C14orf2 | Epi2 | brown | 0.323356472 | 0.122161555 | 0.36454733 | 0.271108641 | 0.26680632 | 0.104135999 | 0.153945279 | 0.21037896 | 0.278258823 |
| NOP10 | Epi2 | brown | 0.219557711 | 0.104889894 | 0.282017792 | 0.166910442 | 0.175499857 | 0.074296515 | 0.037869497 | 0.191682712 | 0.156769876 |
| SPRED1 | Epi2 | brown | 0.163379181 | 0.104320569 | 0.176644079 | 0.068289758 | 0.127151659 | 0.059072176 | 0.039137297 | 0.108093148 | 0.099904314 |
| ZNF106 | Epi2 | brown | 0.108433688 | 0.048364951 | 0.111630498 | 0.043738598 | 0.060997568 | 0.035535812 | 0.018865466 | 0.076600904 | 0.036108543 |
| DUOXA1 | Epi2 | brown | 0.086268424 | 0.004127211 | 0.12338723 | 0.088891054 | 0.063677781 | 0.037755522 | 0.0384016 | 0.060303851 | 0.104021477 |
| RAB27A | Epi2 | brown | 0.157166155 | 0.05752092 | 0.187795804 | 0.072285209 | 0.128548539 | 0.066563812 | 0.021599198 | 0.096849331 | 0.112746263 |
| POLR2M | Epi2 | brown | 0.070658534 | 0.030379036 | 0.091003088 | 0.018641871 | 0.060982568 | 0.016917829 | 0.019862654 | 0.031504807 | 0.042161096 |
| TPM1 | Epi2 | brown | 0.122017155 | 0.044459701 | 0.167047667 | 0.09714055 | 0.114010779 | 0.061279758 | -0.000376924 | 0.09562554 | 0.122091657 |
| CA12 | Epi2 | brown | 0.085193945 | 0.028028883 | 0.172170774 | 0.025896008 | 0.072650014 | 0.013685459 | -0.036928374 | 0.080330565 | 0.092455457 |
| HERC1 | Epi2 | brown | 0.085584641 | -0.011623452 | 0.140435749 | 0.064532288 | 0.069737864 | 0.001048768 | 0.025514421 | 0.084014804 | 0.099788531 |
| NMB | Epi2 | brown | 0.15334392 | 0.05082104 | 0.305169974 | 0.08074221 | 0.160733067 | 0.068813815 | -0.071911535 | 0.151014937 | 0.190754919 |
| STUB1 | Epi2 | brown | 0.120176498 | 0.061969572 | 0.130858455 | 0.059672929 | 0.089284252 | 0.071633084 | 0.030615854 | 0.090986985 | 0.044132094 |
| TSR3 | Epi2 | brown | 0.031046267 | 0.029295409 | 0.042240318 | -0.016015524 | 0.028558602 | -0.022810507 | -0.010088564 | 0.026392222 | -0.019405658 |
| TCEB2 | Epi2 | brown | 0.30965217 | 0.124960494 | 0.375654407 | 0.269276864 | 0.27777646 | 0.069073404 | 0.106464353 | 0.233089053 | 0.287876673 |
| SMIM22 | Epi2 | brown | 0.071976138 | 0.000258394 | 0.103517833 | 0.03835656 | 0.102949566 | 0.032931341 | 0.013363799 | 0.01964433 | 0.106363941 |
| METTL9 | Epi2 | brown | 0.200008486 | 0.06680868 | 0.235672929 | 0.089935596 | 0.197384343 | 0.030639721 | 0.03757439 | 0.096475223 | 0.159100806 |
| TMEM219 | Epi2 | brown | 0.203560138 | 0.091748006 | 0.282175053 | 0.106549371 | 0.168918432 | 0.093032793 | -0.003327352 | 0.216707352 | 0.12732989 |
| CBLN1 | Epi2 | brown | 0.056510767 | -0.010793065 | 0.129121341 | 0.029660766 | 0.093148155 | 0.034520073 | -0.017264087 | 0.029393498 | 0.092045529 |
| CRNDE | Epi2 | brown | 0.097633376 | -0.012376907 | 0.180236243 | 0.017553373 | 0.100916627 | 0.040685038 | -0.007810254 | 0.097214228 | 0.083031478 |
| TAOK1 | Epi2 | brown | 0.113041101 | 0.08265988 | 0.111555108 | 0.065317032 | 0.041323458 | 0.040656382 | -0.002214529 | 0.080988731 | 0.061236495 |
| AP2B1 | Epi2 | brown | 0.150617789 | 0.089565216 | 0.163581399 | 0.084224107 | 0.067156231 | 0.027686772 | 0.012357651 | 0.119412804 | 0.072196095 |
| RARA-AS1 | Epi2 | brown | 0.065647311 | 0.030404859 | 0.102828958 | 0.037523799 | 0.062609115 | -0.001228727 | -0.01364099 | 0.058243834 | 0.055679476 |
| ETV4 | Epi2 | brown | 0.043751458 | -0.003723612 | 0.090198717 | 0.012491611 | 0.058861702 | 0.006143224 | -0.013870608 | 0.057274475 | 0.030888587 |
| MMD | Epi2 | brown | 0.079321964 | -0.038863436 | 0.114875708 | 0.034530913 | 0.068228267 | 0.021342994 | 0.023071821 | 0.043328279 | 0.086561589 |
| KCNJ16 | Epi2 | brown | 0.127356822 | -0.030630624 | 0.186217581 | 0.099872475 | 0.084556338 | 0.107871122 | 0.029514244 | 0.105771655 | 0.140449652 |
| TMC6 | Epi2 | brown | 0.089009929 | 0.06199212 | 0.106030346 | 0.018119727 | 0.036866337 | -0.000902788 | -0.015384012 | 0.092082792 | 0.037143556 |
| C17orf89 | Epi2 | brown | 0.186694628 | 0.136845375 | 0.215803587 | 0.075550986 | 0.139293375 | 0.064181133 | -0.002189309 | 0.160972653 | 0.078994844 |
| EMILIN2 | Epi2 | brown | 0.073421762 | 0.070483528 | 0.115338825 | 0.03598088 | 0.024856412 | 0.019273784 | -0.04117929 | 0.122789957 | 0.017959569 |
| GAREM1 | Epi2 | brown | 0.063839894 | 0.021068463 | 0.094749623 | 0.020410174 | 0.0102939 | -0.003451369 | -0.014461303 | 0.099957254 | 0.012431063 |
| RP11-128M1.1 | Epi2 | brown | 0.042647092 | 0.004574469 | 0.089335178 | 0.03035584 | 0.03381897 | -0.012322311 | -0.024865122 | 0.068429982 | 0.046812787 |
| PLCB4 | Epi2 | brown | 0.067970103 | -0.069911426 | 0.145801176 | 0.024027182 | 0.053880159 | 0.008069591 | 0.005020699 | 0.100874618 | 0.08565312 |
| ROMO1 | Epi2 | brown | 0.299467523 | 0.148469595 | 0.373043313 | 0.243058125 | 0.221478354 | 0.080870596 | 0.070129557 | 0.239338231 | 0.248239706 |
| LINC00657 | Epi2 | brown | 0.146078182 | 0.057195699 | 0.160933904 | 0.049670376 | 0.11991795 | 0.087784059 | 0.024378298 | 0.098448693 | 0.09060414 |
| RP4-569M23.2 | Epi2 | brown | 0.071160646 | 0.005219464 | 0.14100908 | 0.030746894 | 0.091475368 | 0.009613773 | -0.009638385 | 0.055398233 | 0.087129317 |
| GNAS | Epi2 | brown | 0.190542522 | -0.055165132 | 0.298639165 | 0.144796564 | 0.159955467 | 0.056940531 | 0.02490519 | 0.23674942 | 0.17080686 |
| ATP5E | Epi2 | brown | 0.393186461 | 0.127905998 | 0.588618551 | 0.239893914 | 0.334564677 | 0.09241144 | -0.009964146 | 0.418024276 | 0.323741092 |
| PRELID3B | Epi2 | brown | 0.070973644 | 0.045068693 | 0.099932356 | 0.031289205 | 0.027174523 | 0.029748332 | -0.019492024 | 0.094404121 | 0.018829938 |
| GPX4 | Epi2 | brown | 0.297985314 | 0.09620389 | 0.370669704 | 0.225374598 | 0.245231545 | 0.102353597 | 0.053859905 | 0.279630753 | 0.241385328 |
| UQCR11 | Epi2 | brown | 0.334066443 | 0.112925882 | 0.437234642 | 0.220968557 | 0.284204169 | 0.104029783 | 0.075143084 | 0.261654555 | 0.285167155 |
| AES | Epi2 | brown | 0.130639884 | 0.029496417 | 0.195541392 | 0.06113962 | 0.10208716 | 0.050692583 | 0.001624138 | 0.148090298 | 0.069940939 |
| UBL5 | Epi2 | brown | 0.322156486 | 0.150774384 | 0.387020369 | 0.297881556 | 0.248313846 | 0.079981185 | 0.123605947 | 0.275777545 | 0.287044951 |
| C19orf53 | Epi2 | brown | 0.249143286 | 0.139039693 | 0.289017795 | 0.132168743 | 0.224113431 | 0.078012895 | 0.046613448 | 0.18271996 | 0.125174953 |
| SSBP4 | Epi2 | brown | 0.137962804 | 0.07081364 | 0.172088849 | 0.044916595 | 0.092137977 | 0.073510555 | -0.008803482 | 0.133686343 | 0.060875414 |
| BORCS8 | Epi2 | brown | 0.066130901 | 0.026079793 | 0.07666065 | 0.042418454 | 0.030244657 | 0.018732548 | 0.030833042 | 0.052045168 | 0.0508751 |
| ZNF208 | Epi2 | brown | 0.073331544 | 0.000755004 | 0.113360795 | 0.022469918 | 0.083048016 | 0.036635942 | -0.006713846 | 0.068788273 | 0.067882268 |
| LINC00662 | Epi2 | brown | 0.045144887 | 0.013435863 | 0.055404141 | 0.032160959 | 0.029214045 | -0.010034804 | -0.004677234 | 0.040147049 | 0.045030037 |
| RRAS | Epi2 | brown | 0.104852803 | 0.082900797 | 0.133697765 | 0.071542009 | 0.071054271 | 0.038074011 | -0.011538574 | 0.127248952 | 0.079609925 |
| DGCR6L | Epi2 | brown | 0.131108003 | 0.017757111 | 0.175108662 | 0.085870545 | 0.078020546 | 0.033336596 | 0.012643917 | 0.171833196 | 0.095591221 |
| MAPK1 | Epi2 | brown | 0.113098002 | 0.050023185 | 0.148105774 | 0.05308523 | 0.071457988 | 0.036770442 | 0.011957915 | 0.059120519 | 0.088692283 |
| LRP5L | Epi2 | brown | 0.055665746 | -0.009405839 | 0.08269623 | 0.01592027 | 0.045521949 | 0.009356336 | 0.01500033 | 0.053265289 | 0.048728841 |
| INPP5J | Epi2 | brown | 0.057683499 | -0.015615624 | 0.085776764 | 0.061895501 | 0.03153176 | 0.016938927 | 0.000371729 | 0.072582124 | 0.065586544 |
| RPS19BP1 | Epi2 | brown | 0.198167972 | 0.101202669 | 0.242617418 | 0.109403749 | 0.152532846 | 0.080731848 | 0.03129752 | 0.176914981 | 0.136251367 |
| PRR34-AS1 | Epi2 | brown | 0.106514313 | 0.06554416 | 0.166823833 | 0.074040292 | 0.069055482 | 0.034001045 | -0.008397833 | 0.137656645 | 0.097663943 |
| MT-ND1 | Epi2 | brown | 0.201071985 | 0.050578671 | 0.205377344 | 0.077544298 | 0.090208459 | 0.073541584 | 0.064530759 | 0.167895195 | 0.067150368 |
| MT-ND2 | Epi2 | brown | 0.200859743 | 0.049189843 | 0.241921646 | 0.109640422 | 0.081685305 | 0.067967771 | 0.016792905 | 0.215158183 | 0.087823652 |
| MT-CO2 | Epi2 | brown | 0.355562417 | 0.155474989 | 0.412820024 | 0.137228872 | 0.217916194 | 0.10832445 | 0.045039621 | 0.288942596 | 0.172632903 |
| MT-ATP6 | Epi2 | brown | 0.270443776 | 0.076171203 | 0.297589316 | 0.12218769 | 0.142988728 | 0.058312208 | 0.068274065 | 0.201487263 | 0.137262957 |
| MT-ND3 | Epi2 | brown | 0.219287203 | 0.035202725 | 0.294755835 | 0.155171612 | 0.049496258 | 0.064938599 | 0.007830445 | 0.27831108 | 0.11625624 |
| MT-ND4L | Epi2 | brown | 0.111326858 | -0.020082416 | 0.196327746 | 0.0545103 | 0.066511212 | 0.055314348 | -0.037687143 | 0.166694208 | 0.077249723 |
| MT-ND4 | Epi2 | brown | 0.298270575 | 0.021427181 | 0.409849019 | 0.159589402 | 0.134757718 | 0.10329262 | 0.01227509 | 0.326741396 | 0.181650198 |
| MT-ND5 | Epi2 | brown | 0.149473847 | -0.018032335 | 0.246358125 | 0.071730284 | 0.112969412 | 0.068390686 | 6.74E-05 | 0.145898616 | 0.143220772 |
| MT-CYB | Epi2 | brown | 0.218810733 | 0.040407738 | 0.276629781 | 0.115712813 | 0.103553694 | 0.075143929 | 0.034167928 | 0.201877649 | 0.123815347 |
| RER1 | Epi3 | turquoise | 0.130881826 | 0.116962547 | 0.107913591 | 0.153279507 | 0.058800312 | 0.026625525 | 0.064513846 | 0.090547952 | 0.116630233 |
| LRRC47 | Epi3 | turquoise | 0.067024461 | 0.051604335 | 0.024983067 | 0.073942713 | 0.034743052 | 0.010907316 | 0.050416609 | 0.004739441 | 0.051284523 |
| PARK7 | Epi3 | turquoise | 0.221858573 | 0.100487946 | 0.215133239 | 0.309558466 | 0.144694804 | 0.058404265 | 0.116885993 | 0.163261051 | 0.250839924 |
| ENO1 | Epi3 | turquoise | 0.149552788 | 0.153530402 | 0.093578302 | 0.380165526 | 0.016697113 | 0.013871953 | 0.12220372 | 0.147449607 | 0.212567492 |
| SDHB | Epi3 | turquoise | 0.062012382 | 0.092645377 | 0.019147842 | 0.131026971 | 0.016664333 | 0.02575978 | 0.056284413 | 0.050930741 | 0.046338388 |
| AKR7A2 | Epi3 | turquoise | 0.053177368 | -0.001450143 | 0.018585676 | 0.130319783 | 0.036350563 | 0.007502463 | 0.07233402 | 0.020291935 | 0.092689519 |
| CAPZB | Epi3 | turquoise | 0.140613615 | 0.122828915 | 0.106536345 | 0.266038419 | 0.058058356 | 0.040980343 | 0.070457058 | 0.10924772 | 0.159516767 |
| DDOST | Epi3 | turquoise | 0.115017943 | 0.10990885 | 0.039192088 | 0.208509302 | 0.04722209 | 0.008060868 | 0.120296514 | 0.036781022 | 0.120997604 |
| GALE | Epi3 | turquoise | 0.077101669 | 0.056050419 | 0.098100504 | 0.12378774 | 0.033508299 | 0.044490608 | -0.016293346 | 0.115261313 | 0.07735731 |
| HMGCL | Epi3 | turquoise | 0.073129392 | 0.045873304 | 0.036568592 | 0.14950364 | 0.021413194 | 0.046563604 | 0.082083316 | 0.023092374 | 0.08128262 |
| FUCA1 | Epi3 | turquoise | 0.116734521 | 0.223297592 | 0.058034067 | 0.274452232 | -0.009302099 | 0.017833909 | 0.02805031 | 0.137911751 | 0.089701684 |
| SYF2 | Epi3 | turquoise | 0.1721783 | 0.065757988 | 0.139544952 | 0.197781969 | 0.145294975 | 0.050924982 | 0.093263138 | 0.097267033 | 0.171608527 |
| NUDC | Epi3 | turquoise | 0.127681612 | 0.129546932 | 0.08129281 | 0.189869987 | 0.04101209 | 0.05527312 | 0.06020364 | 0.119786318 | 0.093785068 |
| STX12 | Epi3 | turquoise | 0.119463746 | 0.030223219 | 0.112813148 | 0.169769205 | 0.063202221 | 0.042395025 | 0.099906313 | 0.061601632 | 0.136500733 |
| RPA2 | Epi3 | turquoise | 0.091407175 | 0.021497197 | 0.092142355 | 0.162996194 | 0.042784761 | 0.015779321 | 0.058058474 | 0.082519693 | 0.12715638 |
| DNAJC8 | Epi3 | turquoise | 0.13103081 | 0.088801848 | 0.100214222 | 0.185753103 | 0.04544752 | 0.048306574 | 0.069632389 | 0.122838649 | 0.111664003 |
| ATPIF1 | Epi3 | turquoise | 0.248833308 | 0.09112885 | 0.273426296 | 0.256511531 | 0.219113068 | 0.082108841 | 0.09608196 | 0.165386321 | 0.28371053 |
| SNRNP40 | Epi3 | turquoise | 0.068318708 | 0.079582239 | 0.033635724 | 0.154152493 | 0.030930989 | 0.012766237 | 0.043584899 | 0.039735709 | 0.083260972 |
| PEF1 | Epi3 | turquoise | 0.077147908 | 0.054530639 | 0.056701148 | 0.14884149 | 0.026267542 | 0.045022085 | 0.097796963 | 0.042438184 | 0.076196198 |
| EIF3I | Epi3 | turquoise | 0.108582543 | 0.072009652 | 0.089230086 | 0.210455038 | 0.063136369 | 0.009339605 | 0.064239567 | 0.086260771 | 0.129723104 |
| HDAC1 | Epi3 | turquoise | 0.063195258 | 0.067710601 | 0.058985721 | 0.136614291 | 0.006235044 | 0.015619936 | 0.029260886 | 0.084448255 | 0.076176203 |
| RBBP4 | Epi3 | turquoise | 0.109706969 | 0.089392817 | 0.084506994 | 0.141065792 | 0.041282073 | 0.084005148 | 0.032146555 | 0.09099979 | 0.086402958 |
| AK2 | Epi3 | turquoise | 0.120875889 | 0.070461882 | 0.137000827 | 0.168869645 | 0.052018541 | 0.021796233 | 0.035814328 | 0.123070056 | 0.12014686 |
| PSMB2 | Epi3 | turquoise | 0.117923281 | 0.087978006 | 0.113928674 | 0.213784693 | 0.043747994 | 0.033510565 | 0.085328852 | 0.122082837 | 0.147167543 |
| ADPRHL2 | Epi3 | turquoise | 0.086864015 | 0.092051589 | 0.04394456 | 0.158416828 | 0.026680089 | 0.015400109 | 0.066117476 | 0.050363462 | 0.09899167 |
| UTP11L | Epi3 | turquoise | 0.066945739 | 0.05637367 | 0.051092443 | 0.09085212 | 0.037854732 | 0.039312753 | 0.047107789 | 0.044638508 | 0.052794948 |
| NDUFS5 | Epi3 | turquoise | 0.278721832 | 0.101151829 | 0.303744781 | 0.275574806 | 0.292459331 | 0.0679844 | 0.129193375 | 0.140686601 | 0.317414084 |
| PPIE | Epi3 | turquoise | 0.068371587 | 0.003498349 | 0.061437521 | 0.15353854 | 0.051004545 | 0.039118662 | 0.077592777 | 0.033276078 | 0.12054391 |
| MFSD2A | Epi3 | turquoise | 0.075562929 | 0.165508081 | 0.057883744 | 0.149985229 | -0.11396242 | 0.045847198 | -0.084199119 | 0.241523287 | -0.028545552 |
| CAP1 | Epi3 | turquoise | 0.152831904 | 0.130669257 | 0.144025335 | 0.146041123 | 0.089591696 | 0.051356517 | 0.034223921 | 0.093026294 | 0.119979905 |
| PPT1 | Epi3 | turquoise | 0.062638878 | 0.06815044 | 0.009104162 | 0.13642838 | 0.018253949 | -0.015102054 | 0.063283478 | 0.003289644 | 0.073860042 |
| PPIH | Epi3 | turquoise | 0.052237477 | 0.031875875 | 0.0233353 | 0.099961445 | 0.021855076 | 0.017762302 | 0.054340046 | 0.013833365 | 0.077393646 |
| ELOVL1 | Epi3 | turquoise | 0.055652209 | 0.045335995 | 0.019736582 | 0.085775389 | 0.036793146 | 0.006927776 | 0.090494789 | 0.010097362 | 0.039009963 |
| MED8 | Epi3 | turquoise | 0.049920968 | 0.033979379 | 0.020552035 | 0.08562697 | 0.042579043 | 0.000822551 | 0.08334418 | -0.000800446 | 0.046912489 |
| HYI | Epi3 | turquoise | 0.084095821 | 0.087450788 | 0.07641766 | 0.139157685 | 0.057556296 | 0.053489341 | -0.010774193 | 0.111094104 | 0.079526269 |
| ATP6V0B | Epi3 | turquoise | 0.168850674 | 0.179723057 | 0.123716401 | 0.242340399 | 0.036446249 | 0.008243302 | 0.044366336 | 0.173788202 | 0.094419215 |
| DMAP1 | Epi3 | turquoise | 0.054804637 | 0.035309939 | 0.036419577 | 0.104182016 | 0.03466521 | -0.013414092 | 0.084939882 | 0.023442656 | 0.069189473 |
| ERI3 | Epi3 | turquoise | 0.090261576 | 0.058972064 | 0.072293925 | 0.119616271 | 0.027515135 | 0.034996178 | 0.075935254 | 0.056540131 | 0.081891363 |
| UROD | Epi3 | turquoise | 0.078198511 | 0.056679379 | 0.03826365 | 0.157960438 | 0.002392778 | 0.014750067 | 0.039857239 | 0.063025205 | 0.079801084 |
| PRDX1 | Epi3 | turquoise | 0.198438907 | 0.13529253 | 0.107805738 | 0.532466834 | 0.02744868 | 0.028011556 | 0.285207385 | 0.108494239 | 0.3263504 |
| AKR1A1 | Epi3 | turquoise | 0.105355564 | 0.03854472 | 0.084717278 | 0.139359933 | 0.110094352 | 0.046003407 | 0.120452095 | 0.023973737 | 0.136536642 |
| NASP | Epi3 | turquoise | 0.034432622 | 0.028645816 | -0.01101115 | 0.121980889 | -0.026909385 | -0.016040021 | 0.080754714 | 0.013403142 | 0.041088104 |
| TMEM69 | Epi3 | turquoise | 0.085688102 | 0.037566883 | 0.09177662 | 0.096596931 | 0.055809178 | 0.035712471 | 0.024856413 | 0.070761495 | 0.072030672 |
| LRRC41 | Epi3 | turquoise | 0.054714388 | 0.084971278 | 0.036810033 | 0.121634312 | 0.02145298 | 0.028515182 | 0.076412444 | 0.022082495 | 0.066666942 |
| BTF3L4 | Epi3 | turquoise | 0.147219024 | 0.11041481 | 0.111649917 | 0.198882525 | 0.086407361 | 0.074804279 | 0.104813105 | 0.102553546 | 0.126543133 |
| ECHDC2 | Epi3 | turquoise | 0.092105878 | 0.046773905 | 0.049060372 | 0.144655495 | 0.028660014 | 0.026791918 | 0.101685281 | 0.03488364 | 0.079964109 |
| SCP2 | Epi3 | turquoise | 0.158102447 | 0.041649298 | 0.162035591 | 0.250788249 | 0.109109842 | 0.041774592 | 0.115148888 | 0.122818106 | 0.192833898 |
| C1orf123 | Epi3 | turquoise | 0.073963257 | 0.018619579 | 0.072927582 | 0.116161369 | 0.040114415 | 0.049587629 | 0.047091112 | 0.060327776 | 0.070801993 |
| MAGOH | Epi3 | turquoise | 0.076409841 | 0.047674887 | 0.045657867 | 0.149170184 | 0.039801022 | -0.00327551 | 0.07600202 | 0.030998149 | 0.082949189 |
| TMEM59 | Epi3 | turquoise | 0.233789511 | 0.207827931 | 0.177620062 | 0.555797465 | 0.027024618 | 0.063395249 | 0.136307671 | 0.252106891 | 0.338867371 |
| OMA1 | Epi3 | turquoise | 0.03324154 | 0.041316581 | 0.022989641 | 0.119093314 | -0.004673308 | -0.00798572 | 0.049178978 | 0.029889358 | 0.059927865 |
| TM2D1 | Epi3 | turquoise | 0.094832936 | 0.055941193 | 0.085963027 | 0.162020216 | 0.031832964 | 0.018666787 | 0.042552251 | 0.115789998 | 0.102242845 |
| ITGB3BP | Epi3 | turquoise | 0.104240167 | 0.047631812 | 0.090604171 | 0.123488746 | 0.05295537 | 0.040109021 | 0.038234831 | 0.097244707 | 0.077867379 |
| LEPROT | Epi3 | turquoise | 0.124107177 | 0.057383996 | 0.093967391 | 0.14565112 | 0.083311717 | 0.049979173 | 0.086531869 | 0.048585999 | 0.140735239 |
| SRSF11 | Epi3 | turquoise | 0.182133151 | 0.099564437 | 0.116215223 | 0.155608237 | 0.093430873 | 0.05847624 | 0.08512629 | 0.089618027 | 0.10729587 |
| PIGK | Epi3 | turquoise | 0.049334709 | 0.068029843 | 0.015655706 | 0.127059954 | 0.002678054 | 0.003940823 | 0.051473791 | 0.039103478 | 0.066573278 |
| RPF1 | Epi3 | turquoise | 0.056878944 | 0.045439665 | 0.027183712 | 0.106916078 | 0.02414314 | 0.027526311 | 0.054621267 | 0.022830656 | 0.073978702 |
| CTBS | Epi3 | turquoise | 0.0474983 | 0.019847812 | 0.026143168 | 0.079121512 | 0.022866372 | -0.007485887 | 0.018544284 | 0.036415203 | 0.049304198 |
| 15-Sep | Epi3 | turquoise | 0.161643519 | 0.113693647 | 0.126877841 | 0.270164072 | 0.044252094 | 0.034034087 | 0.051013831 | 0.1720496 | 0.164516283 |
| GTF2B | Epi3 | turquoise | 0.065858231 | 0.080212555 | 0.010374042 | 0.18882601 | -0.013337728 | 0.006929939 | 0.080846513 | 0.044609109 | 0.094364855 |
| DNTTIP2 | Epi3 | turquoise | 0.053176989 | 0.070237085 | 0.032070335 | 0.082003163 | 0.007534014 | 0.017498104 | 0.025314996 | 0.067068007 | 0.040224913 |
| ALG14 | Epi3 | turquoise | 0.056169887 | 0.05452235 | 0.05602641 | 0.09756676 | 0.02320127 | 0.024363152 | 0.024842102 | 0.069951038 | 0.049331296 |
| STXBP3 | Epi3 | turquoise | 0.077765435 | 0.064704556 | 0.059316607 | 0.083759908 | 0.028186857 | 0.038132399 | 0.036702599 | 0.041135191 | 0.046969984 |
| SARS | Epi3 | turquoise | 0.088531293 | 0.071224148 | 0.063472205 | 0.159466753 | 0.023544901 | 0.047601868 | 0.037549994 | 0.108807943 | 0.092781086 |
| PSMA5 | Epi3 | turquoise | 0.090945703 | 0.097149166 | 0.062023259 | 0.162274899 | 0.013373009 | 0.006343805 | 0.050290062 | 0.069050192 | 0.088587223 |
| GSTM3 | Epi3 | turquoise | 0.042094623 | 0.008659771 | -0.013898819 | 0.134547014 | -0.006045991 | 0.01503917 | 0.136397069 | -0.013661924 | 0.062451671 |
| LAMTOR5 | Epi3 | turquoise | 0.201464016 | 0.105669245 | 0.200506468 | 0.246207876 | 0.107886352 | 0.038719491 | 0.080934497 | 0.162544884 | 0.19036785 |
| CEPT1 | Epi3 | turquoise | 0.040105478 | 0.016040277 | 0.040362647 | 0.089170251 | 0.018584243 | 0.007538607 | 0.036903311 | 0.038913647 | 0.064352664 |
| ATP5F1 | Epi3 | turquoise | 0.132754186 | 0.072303928 | 0.104708663 | 0.209241919 | 0.070508178 | 0.040225847 | 0.100917228 | 0.092267271 | 0.135446921 |
| BCAS2 | Epi3 | turquoise | 0.077593463 | 0.051536946 | 0.024764306 | 0.129838721 | 0.023703686 | 0.004200411 | 0.081988973 | 0.032055837 | 0.080069928 |
| SIKE1 | Epi3 | turquoise | 0.045704866 | 0.030656562 | 0.037665987 | 0.076298346 | 0.020587898 | 0.021451432 | 0.050779224 | 0.041319374 | 0.051368028 |
| ATP1A1 | Epi3 | turquoise | 0.123886822 | 0.134296627 | 0.008112429 | 0.193693208 | -0.011151614 | 0.05034261 | 0.187061269 | 0.004815295 | 0.051520482 |
| CD58 | Epi3 | turquoise | 0.079085964 | 0.140216236 | 0.05834463 | 0.155616461 | -0.026131634 | 0.018729148 | -0.006785494 | 0.130540533 | 0.047350728 |
| RBM8A | Epi3 | turquoise | 0.128863128 | 0.100673994 | 0.09477719 | 0.154751752 | 0.071311956 | 0.045255645 | 0.070246593 | 0.070466327 | 0.105214911 |
| SF3B4 | Epi3 | turquoise | 0.02606035 | 0.018335831 | -0.002800085 | 0.131038378 | -0.008348998 | -0.022086953 | 0.074769131 | 0.003368815 | 0.063331494 |
| ECM1 | Epi3 | turquoise | 0.13610355 | 0.123557857 | 0.202436631 | 0.383933126 | 0.035088266 | 0.007535114 | 0.009642596 | 0.205704812 | 0.2911511 |
| CTSS | Epi3 | turquoise | 0.108766583 | 0.173545394 | 0.081071622 | 0.159791659 | 0.015801645 | 0.001532622 | 0.019486686 | 0.092875456 | 0.079082987 |
| PSMD4 | Epi3 | turquoise | 0.088350755 | 0.083317154 | 0.066972186 | 0.152138313 | 0.034852864 | 0.000705937 | 0.055964202 | 0.076087694 | 0.07396137 |
| SELENBP1 | Epi3 | turquoise | 0.053618095 | 0.102577172 | -0.028187233 | 0.269592258 | -0.080073995 | -0.001204703 | 0.11386614 | 0.071218053 | 0.07613205 |
| S100A11 | Epi3 | turquoise | 0.289739176 | 0.311755338 | 0.271924721 | 0.390525475 | 0.18178838 | 0.099040264 | 0.082068057 | 0.232079902 | 0.280563621 |
| ILF2 | Epi3 | turquoise | 0.086530368 | 0.044507025 | 0.053984872 | 0.195331637 | 0.047404066 | 0.005429345 | 0.10760633 | 0.037299311 | 0.13249643 |
| PMVK | Epi3 | turquoise | 0.110568695 | 0.069207493 | 0.102444372 | 0.101276643 | 0.064339673 | 0.071994594 | -0.000716256 | 0.114885645 | 0.064078848 |
| PBXIP1 | Epi3 | turquoise | 0.057929955 | 0.084771623 | 0.014152444 | 0.089625277 | -0.002803809 | 0.003365694 | 0.046487112 | 0.026170832 | 0.035036541 |
| EFNA4 | Epi3 | turquoise | 0.032351093 | 0.035389636 | 0.02733091 | 0.056556377 | 0.008503524 | 0.020760319 | 0.018192652 | 0.031361921 | 0.039815855 |
| SLC50A1 | Epi3 | turquoise | 0.093650755 | 0.054935002 | 0.084719835 | 0.114756996 | 0.065453744 | 0.033238972 | 0.051753554 | 0.063621231 | 0.102434477 |
| DPM3 | Epi3 | turquoise | 0.128329796 | 0.074109649 | 0.108714588 | 0.219874891 | 0.060597476 | 0.017465424 | 0.07299737 | 0.105266453 | 0.138516573 |
| KRTCAP2 | Epi3 | turquoise | 0.150878244 | 0.156468169 | 0.141854542 | 0.293993075 | 0.079251401 | 0.032004188 | 0.082020855 | 0.11882366 | 0.18871422 |
| MTX1 | Epi3 | turquoise | 0.059334504 | 0.057095276 | 0.037617144 | 0.146816901 | -0.00267369 | 0.043030473 | 0.059690533 | 0.047280566 | 0.086640194 |
| SCAMP3 | Epi3 | turquoise | 0.090710786 | 0.076499916 | 0.053338965 | 0.099714356 | 0.038687134 | 0.031377036 | 0.027627199 | 0.055340986 | 0.055891689 |
| FDPS | Epi3 | turquoise | 0.06727525 | 0.066061594 | 0.008905704 | 0.158124723 | 0.027748094 | 0.034859524 | 0.10555848 | 0.023492181 | 0.086651827 |
| LMNA | Epi3 | turquoise | 0.192976083 | 0.218050477 | 0.117391326 | 0.224892388 | 0.051572108 | 0.061048556 | 0.089797474 | 0.141545346 | 0.109663334 |
| PMF1 | Epi3 | turquoise | 0.078870451 | 0.054609672 | 0.038170277 | 0.141643867 | 0.025937098 | 0.009810903 | 0.06742531 | 0.062875286 | 0.082558463 |
| CCT3 | Epi3 | turquoise | 0.128552692 | 0.068110819 | 0.105905883 | 0.183488321 | 0.089142576 | 0.024440831 | 0.06423621 | 0.071010506 | 0.141922213 |
| APOA1BP | Epi3 | turquoise | 0.115866868 | 0.082526002 | 0.102337132 | 0.109873698 | 0.055708961 | 0.016566503 | 0.052732638 | 0.079919355 | 0.074790746 |
| TAGLN2 | Epi3 | turquoise | 0.138825756 | 0.225600068 | 0.050063701 | 0.209205347 | 0.029942808 | 0.06165739 | 0.074949235 | 0.088796492 | 0.064535761 |
| IGSF8 | Epi3 | turquoise | 0.075183749 | 0.046485337 | 0.056945053 | 0.110356967 | 0.044655892 | 0.009374759 | 0.045951558 | 0.029828858 | 0.082202642 |
| NCSTN | Epi3 | turquoise | 0.051987567 | 0.076018969 | 0.033938994 | 0.100490395 | -0.02390903 | 0.008750377 | 0.014333609 | 0.063081669 | 0.040921793 |
| PFDN2 | Epi3 | turquoise | 0.10353503 | 0.108297892 | 0.098471885 | 0.134467584 | 0.066165462 | 0.04024931 | 0.041631263 | 0.096735411 | 0.091747452 |
| NDUFS2 | Epi3 | turquoise | 0.094912838 | 0.123751738 | 0.054422645 | 0.190949642 | 0.001045187 | 0.023622378 | 0.066268347 | 0.093192654 | 0.073206849 |
| SDHC | Epi3 | turquoise | 0.086739184 | 0.035566347 | 0.054105344 | 0.156269367 | 0.060125994 | 0.021724203 | 0.080343478 | 0.021190711 | 0.115778538 |
| MGST3 | Epi3 | turquoise | 0.246899112 | 0.253075615 | 0.225771 | 0.481198156 | 0.019749977 | 0.050168951 | 0.107604994 | 0.30027132 | 0.253558167 |
| ALDH9A1 | Epi3 | turquoise | 0.139554743 | 0.092515761 | 0.106653334 | 0.215879607 | 0.025401768 | 0.06464055 | 0.082789335 | 0.108971519 | 0.104296558 |
| TMCO1 | Epi3 | turquoise | 0.139265423 | 0.083382885 | 0.105393052 | 0.180196566 | 0.040156391 | 0.027900586 | 0.074406144 | 0.111482285 | 0.103143312 |
| PRDX6 | Epi3 | turquoise | 0.164416439 | 0.096162635 | 0.100360068 | 0.255044076 | 0.129807641 | 0.031487142 | 0.178242406 | 0.050842371 | 0.167187465 |
| CACYBP | Epi3 | turquoise | 0.113782662 | 0.099243349 | 0.088435497 | 0.160299368 | 0.058985279 | 0.0183279 | 0.058593426 | 0.102740672 | 0.095481336 |
| QSOX1 | Epi3 | turquoise | 0.034742833 | 0.064319579 | 0.024481451 | 0.067318012 | 0.000850671 | 0.015066718 | 0.001071797 | 0.042883693 | 0.052276641 |
| CFH | Epi3 | turquoise | 0.063716021 | 0.117448047 | -0.003314158 | 0.163578878 | 0.011582633 | 0.01316595 | 0.085709299 | -0.019282416 | 0.091056801 |
| TMEM9 | Epi3 | turquoise | 0.092427368 | 0.088085124 | 0.04268228 | 0.164891657 | 0.027568488 | 0.018100847 | 0.079624462 | 0.048274589 | 0.085965704 |
| CSRP1 | Epi3 | turquoise | 0.108614778 | 0.097565895 | 0.079113019 | 0.116622121 | 0.017267552 | 0.044157953 | 0.036427455 | 0.114440157 | 0.044687051 |
| RNPEP | Epi3 | turquoise | 0.055356697 | 0.022249118 | 0.036424418 | 0.094083153 | 0.045364682 | 0.02497069 | 0.058753611 | 0.024965783 | 0.07469938 |
| CYB5R1 | Epi3 | turquoise | 0.066099256 | 0.073998922 | 0.041289103 | 0.150554567 | -0.001680727 | 0.032517457 | 0.039331135 | 0.06182709 | 0.065632279 |
| CD55 | Epi3 | turquoise | 0.07687629 | 0.073049446 | 0.056895089 | 0.116994121 | 0.060690903 | 0.052344796 | 0.058586595 | 0.019633037 | 0.089492369 |
| LYPLAL1 | Epi3 | turquoise | 0.068494997 | -0.004106189 | 0.089398227 | 0.119048181 | 0.022000539 | 0.017845758 | 0.022675017 | 0.084269277 | 0.089586605 |
| DEGS1 | Epi3 | turquoise | 0.090639164 | 0.085685683 | 0.081603359 | 0.248491555 | -0.008387977 | 0.012004493 | 0.059562631 | 0.116500689 | 0.139508306 |
| CNIH4 | Epi3 | turquoise | 0.131693761 | 0.149843846 | 0.110850287 | 0.207155914 | 0.031384449 | 0.050918153 | 0.031725204 | 0.118495632 | 0.110410954 |
| EPHX1 | Epi3 | turquoise | 0.095472744 | 0.20060412 | 0.017484862 | 0.221534036 | -0.044099768 | 0.030143615 | 0.036239778 | 0.138830139 | 0.026113519 |
| H3F3A | Epi3 | turquoise | 0.360369747 | 0.14252721 | 0.356804294 | 0.439077691 | 0.26885607 | 0.093115976 | 0.183482543 | 0.241154131 | 0.390909552 |
| ARF1 | Epi3 | turquoise | 0.130689166 | 0.093772829 | 0.06086985 | 0.170224323 | 0.060160154 | 0.045011361 | 0.097254535 | 0.043939988 | 0.103940817 |
| RNF187 | Epi3 | turquoise | 0.114256051 | 0.074388453 | 0.077068552 | 0.13842907 | 0.077498778 | 0.025014524 | 0.059722883 | 0.077760231 | 0.076611469 |
| ARV1 | Epi3 | turquoise | 0.063305893 | 0.004928549 | 0.066969563 | 0.113503756 | 0.04791983 | 0.012634711 | 0.06151215 | 0.015269636 | 0.110197654 |
| C1orf131 | Epi3 | turquoise | 0.061718993 | 0.031215657 | 0.044374553 | 0.092235456 | 0.03689576 | 0.015977466 | 0.049014292 | 0.022095358 | 0.056642804 |
| TSNAX | Epi3 | turquoise | 0.072118511 | 0.036068902 | 0.059260608 | 0.109709947 | 0.020018216 | 0.054308633 | 0.057272619 | 0.077977603 | 0.043778263 |
| SMYD3 | Epi3 | turquoise | 0.076509108 | 0.09473371 | 0.046963348 | 0.170164392 | 0.021463848 | 0.030949472 | 0.066492063 | 0.064613117 | 0.078822918 |
| TFB2M | Epi3 | turquoise | 0.045690102 | 0.025942764 | 0.029659082 | 0.085262894 | 0.005306058 | 0.012631699 | 0.028737059 | 0.023997733 | 0.044284767 |
| SCCPDH | Epi3 | turquoise | 0.093085242 | 0.054321648 | 0.034407061 | 0.154048162 | 0.061464171 | 0.006630453 | 0.102808527 | 0.01540305 | 0.106640584 |
| ADI1 | Epi3 | turquoise | 0.094854785 | 0.086090802 | 0.034702655 | 0.187188294 | 0.032567078 | 0.034551247 | 0.122926679 | 0.045295229 | 0.093207401 |
| YWHAQ | Epi3 | turquoise | 0.186131939 | 0.113230283 | 0.137183429 | 0.227908486 | 0.111154115 | 0.059644997 | 0.138472261 | 0.088413963 | 0.163485442 |
| PDIA6 | Epi3 | turquoise | 0.172911647 | 0.143724975 | 0.073905754 | 0.281550804 | 0.043164157 | 0.01309977 | 0.179011302 | 0.069718996 | 0.160016725 |
| DDX1 | Epi3 | turquoise | 0.104387928 | 0.050764277 | 0.112357058 | 0.150423025 | 0.045616872 | 0.025015295 | 0.027620544 | 0.089907146 | 0.104390128 |
| LAPTM4A | Epi3 | turquoise | 0.2068268 | 0.204367535 | 0.131376716 | 0.571709446 | 0.064308117 | 0.031245662 | 0.205282928 | 0.148423778 | 0.361429071 |
| SF3B6 | Epi3 | turquoise | 0.191783279 | 0.085244907 | 0.172754866 | 0.177700105 | 0.143594681 | 0.04541874 | 0.095898555 | 0.110802905 | 0.158596399 |
| HADHB | Epi3 | turquoise | 0.105074141 | 0.07017393 | 0.050464825 | 0.157635324 | 0.041574686 | 0.043110942 | 0.090627888 | 0.044131701 | 0.092444147 |
| ATRAID | Epi3 | turquoise | 0.174631003 | 0.122375719 | 0.13679277 | 0.278979893 | 0.067228436 | 0.049170789 | 0.101259729 | 0.153475193 | 0.187369154 |
| MPV17 | Epi3 | turquoise | 0.095181989 | 0.07058553 | 0.093900286 | 0.144446628 | 0.032439219 | 0.01163392 | 0.034838113 | 0.119879416 | 0.085227928 |
| NRBP1 | Epi3 | turquoise | 0.070409803 | 0.060831917 | 0.034056284 | 0.10836274 | 0.030762774 | 0.004515995 | 0.047158061 | 0.041439095 | 0.03498463 |
| BRE | Epi3 | turquoise | 0.06048857 | 0.06003013 | 0.049578334 | 0.130321548 | 0.014735559 | -0.003498318 | 0.058054139 | 0.053934979 | 0.064815021 |
| YPEL5 | Epi3 | turquoise | 0.176586697 | 0.151630906 | 0.131278324 | 0.149946552 | 0.064504495 | 0.04838813 | 0.062450407 | 0.148942964 | 0.073214095 |
| DPY30 | Epi3 | turquoise | 0.168869507 | 0.106477724 | 0.150640749 | 0.188198048 | 0.111324398 | 0.035804235 | 0.065227393 | 0.128118864 | 0.137990974 |
| YIPF4 | Epi3 | turquoise | 0.099229337 | 0.045822178 | 0.073830538 | 0.117065311 | 0.056139098 | 0.025299614 | 0.067448581 | 0.067799648 | 0.089483692 |
| CEBPZOS | Epi3 | turquoise | 0.096973135 | 0.045641104 | 0.071583667 | 0.105654955 | 0.039950247 | 0.016612126 | 0.053207363 | 0.071744947 | 0.068497745 |
| QPCT | Epi3 | turquoise | 0.117490956 | 0.176976685 | 0.105686643 | 0.248232605 | -0.081858926 | 0.02001871 | -0.040091054 | 0.277450435 | 0.043465255 |
| ATL2 | Epi3 | turquoise | 0.08338256 | 0.112899588 | -0.001052976 | 0.107585637 | 0.015920587 | 0.014781248 | 0.078468977 | 0.003132903 | 0.034220506 |
| SRSF7 | Epi3 | turquoise | 0.124260549 | 0.080211882 | 0.044895972 | 0.147981987 | 0.037590774 | 0.029431914 | 0.104552209 | 0.050246441 | 0.06478808 |
| MORN2 | Epi3 | turquoise | 0.099415669 | 0.051335249 | 0.083303738 | 0.138857735 | 0.030228376 | 0.021943744 | 0.076429981 | 0.072840231 | 0.085731532 |
| DYNC2LI1 | Epi3 | turquoise | 0.055291831 | 0.024175512 | 0.047414635 | 0.133103678 | 0.014206092 | 0.035316728 | 0.057676526 | 0.057919463 | 0.08333078 |
| PIGF | Epi3 | turquoise | 0.109407755 | 0.083854666 | 0.079280379 | 0.108360895 | 0.042238245 | 0.046513486 | 0.04096495 | 0.080757579 | 0.071168859 |
| CALM2 | Epi3 | turquoise | 0.374989477 | 0.258250216 | 0.376193093 | 0.535391507 | 0.184103049 | 0.099990176 | 0.107597926 | 0.379457616 | 0.389596207 |
| EPCAM | Epi3 | turquoise | 0.151221013 | 0.092075177 | 0.070251541 | 0.453830152 | 0.090464674 | 0.034973554 | 0.237569251 | -0.008926751 | 0.360756596 |
| EFEMP1 | Epi3 | turquoise | 0.098800124 | 0.0730143 | 0.09264821 | 0.169209122 | 0.096686358 | 0.009925732 | 0.093583188 | -0.001392896 | 0.168780459 |
| FANCL | Epi3 | turquoise | 0.040609146 | 0.029642684 | 0.040522707 | 0.099850416 | -0.002049778 | 0.034175619 | 0.019719418 | 0.060414125 | 0.052222729 |
| CCT4 | Epi3 | turquoise | 0.143593886 | 0.070423919 | 0.083992675 | 0.218060812 | 0.099649458 | 0.034745403 | 0.117322949 | 0.067518527 | 0.146521189 |
| COMMD1 | Epi3 | turquoise | 0.115827387 | 0.059783261 | 0.092449572 | 0.143837769 | 0.059336117 | 0.028966962 | 0.061314659 | 0.088015446 | 0.114463542 |
| MDH1 | Epi3 | turquoise | 0.123319598 | 0.111251042 | 0.061058601 | 0.315736682 | -0.01071811 | 0.021339651 | 0.1097524 | 0.119033655 | 0.133600626 |
| UGP2 | Epi3 | turquoise | 0.118760494 | 0.104634091 | 0.074059501 | 0.107151748 | 0.080170122 | 0.044315083 | 0.058156344 | 0.052664087 | 0.05680337 |
| RAB1A | Epi3 | turquoise | 0.179391105 | 0.130029169 | 0.102003858 | 0.224788904 | 0.078457838 | 0.035168241 | 0.12102502 | 0.099624204 | 0.133511837 |
| C1D | Epi3 | turquoise | 0.131105828 | 0.104999157 | 0.089120663 | 0.221646801 | 0.038499044 | 0.061536668 | 0.073025326 | 0.116282561 | 0.125505859 |
| ANXA4 | Epi3 | turquoise | 0.116540623 | 0.076169082 | 0.04186146 | 0.210926835 | 0.06332029 | 0.016623114 | 0.148171761 | 0.025212479 | 0.134960153 |
| NAGK | Epi3 | turquoise | 0.063370461 | 0.023805686 | 0.047000018 | 0.105411489 | 0.032769317 | 0.009244181 | 0.05968126 | 0.016588313 | 0.072820611 |
| CCT7 | Epi3 | turquoise | 0.053796591 | 0.048678286 | 0.033419067 | 0.158308507 | 0.007168681 | 0.01691396 | 0.04062488 | 0.077809737 | 0.085401292 |
| DUSP11 | Epi3 | turquoise | 0.031864848 | 0.015171531 | 0.004434393 | 0.064017741 | 0.000541088 | 0.030018419 | 0.027256663 | 0.010931429 | 0.028216443 |
| AUP1 | Epi3 | turquoise | 0.101212487 | 0.060396045 | 0.09709852 | 0.105622526 | 0.064473429 | 0.00640336 | 0.035849133 | 0.069052627 | 0.079142142 |
| HTRA2 | Epi3 | turquoise | 0.05308968 | 0.049239738 | 0.020233942 | 0.099804983 | -0.010547898 | -0.005430635 | 0.030019034 | 0.041389561 | 0.051271282 |
| SUCLG1 | Epi3 | turquoise | 0.076535611 | 0.079315466 | 0.006629563 | 0.228544048 | -0.023906899 | 0.014756476 | 0.097721805 | 0.065192506 | 0.100594591 |
| CAPG | Epi3 | turquoise | 0.157556436 | 0.143243275 | 0.155921753 | 0.370836236 | 0.084132817 | 0.038386866 | 0.100150138 | 0.143974197 | 0.259417359 |
| RNF181 | Epi3 | turquoise | 0.112311798 | 0.099895519 | 0.082398648 | 0.164377677 | 0.067422509 | 0.031387125 | 0.069520598 | 0.076888948 | 0.110261445 |
| MRPS5 | Epi3 | turquoise | 0.088195984 | 0.04672799 | 0.073572891 | 0.113753211 | 0.039749719 | 0.024029286 | 0.070730834 | 0.058587059 | 0.08358004 |
| COX5B | Epi3 | turquoise | 0.239675075 | 0.096454276 | 0.214807541 | 0.288146967 | 0.161015635 | 0.027697125 | 0.172250575 | 0.130477193 | 0.225869489 |
| UNC50 | Epi3 | turquoise | 0.104413285 | 0.122866853 | 0.069050187 | 0.221925328 | 0.010107426 | 0.024215941 | 0.063913846 | 0.111666951 | 0.107096315 |
| TXNDC9 | Epi3 | turquoise | 0.099641186 | 0.116295677 | 0.079767752 | 0.158744996 | 0.025478084 | 0.025999468 | 0.035201546 | 0.110585024 | 0.076272501 |
| PDCL3 | Epi3 | turquoise | 0.069422824 | 0.054322376 | 0.066190064 | 0.123182401 | 0.036060016 | 0.002645608 | 0.047683299 | 0.051083433 | 0.073926421 |
| RNF149 | Epi3 | turquoise | 0.062372384 | 0.081893263 | 0.024360523 | 0.116050133 | 0.004776032 | 0.002678651 | 0.044130743 | 0.057585705 | 0.046747782 |
| C2orf40 | Epi3 | turquoise | 0.10810769 | -0.052226242 | 0.098132783 | 0.37325389 | 0.069050043 | 0.021780785 | 0.1904768 | 0.101365115 | 0.327082453 |
| LINC00116 | Epi3 | turquoise | 0.148295752 | 0.055849591 | 0.162096224 | 0.145999192 | 0.105885626 | 0.036821024 | 0.044754069 | 0.107344651 | 0.136607447 |
| C2orf76 | Epi3 | turquoise | 0.078774499 | 0.008639837 | 0.083733867 | 0.08689632 | 0.025791946 | 0.007195158 | 0.034405156 | 0.066442758 | 0.066751749 |
| NIFK | Epi3 | turquoise | 0.112933439 | 0.037497377 | 0.086187735 | 0.148331884 | 0.048247519 | 0.027492505 | 0.080558185 | 0.065909988 | 0.094636949 |
| TSN | Epi3 | turquoise | 0.09750883 | 0.025607665 | 0.091869507 | 0.118357535 | 0.06690019 | 0.031080801 | 0.057839971 | 0.047784533 | 0.119320383 |
| WDR33 | Epi3 | turquoise | 0.093831972 | 0.055145747 | 0.05634752 | 0.080704414 | 0.048354579 | 0.019506382 | 0.030217413 | 0.052957128 | 0.052174434 |
| CCDC115 | Epi3 | turquoise | 0.086381167 | 0.010868685 | 0.062364332 | 0.127987459 | 0.062229509 | 0.0381592 | 0.066597221 | 0.040582194 | 0.112553735 |
| UBXN4 | Epi3 | turquoise | 0.130095324 | 0.115796978 | 0.087646076 | 0.172184864 | 0.059697517 | 0.018644876 | 0.072370609 | 0.064363671 | 0.112990555 |
| DARS | Epi3 | turquoise | 0.05452129 | 0.043007482 | 0.035270263 | 0.110021129 | 0.039266261 | 0.036546555 | 0.071174992 | 0.028573752 | 0.068502065 |
| HNMT | Epi3 | turquoise | 0.044447423 | 0.07333748 | 0.023402614 | 0.071644482 | 0.049199178 | 0.020110849 | 0.007903514 | 0.009075711 | 0.050866108 |
| ORC4 | Epi3 | turquoise | 0.084358652 | 0.051289172 | 0.05441795 | 0.118004008 | 0.060867142 | 0.036899797 | 0.044502271 | 0.051434564 | 0.071926777 |
| MMADHC | Epi3 | turquoise | 0.113149196 | 0.088056092 | 0.066915879 | 0.161584477 | 0.047918878 | 0.005605319 | 0.05504109 | 0.093271117 | 0.08182429 |
| ARL6IP6 | Epi3 | turquoise | 0.064458385 | -0.001148578 | 0.056475754 | 0.092478687 | 0.030659379 | -0.023921702 | 0.026341529 | 0.032004084 | 0.082024249 |
| DAPL1 | Epi3 | turquoise | 0.123552932 | 0.146102064 | 0.145698423 | 0.226802209 | -0.003654019 | 0.042805667 | -0.051537168 | 0.279891823 | 0.094299382 |
| 7-Mar | Epi3 | turquoise | 0.084902707 | 0.055888368 | 0.059263815 | 0.08520831 | 0.02917245 | 0.027399867 | 0.028850981 | 0.059313171 | 0.050175591 |
| TANK | Epi3 | turquoise | 0.142803474 | 0.110784407 | 0.117366162 | 0.157136735 | 0.033773075 | 0.04585098 | 0.027684709 | 0.147093285 | 0.066843186 |
| PSMD14 | Epi3 | turquoise | 0.127006726 | 0.098866476 | 0.102863105 | 0.155707102 | 0.051763466 | 0.03035578 | 0.036101531 | 0.0993297 | 0.090693219 |
| GCA | Epi3 | turquoise | 0.078991999 | 0.045030286 | 0.066947558 | 0.123039726 | 0.036709169 | 0.034084773 | 0.033043493 | 0.06381351 | 0.083358766 |
| SSB | Epi3 | turquoise | 0.170393959 | 0.061355108 | 0.15385874 | 0.262138246 | 0.074783076 | 0.026220382 | 0.103493187 | 0.120616534 | 0.216522624 |
| GORASP2 | Epi3 | turquoise | 0.086856725 | 0.031964717 | 0.074276864 | 0.12974108 | 0.064260645 | 0.009424993 | 0.053994115 | 0.056073034 | 0.105033597 |
| DYNC1I2 | Epi3 | turquoise | 0.161012038 | 0.107643958 | 0.141704801 | 0.16549885 | 0.094096034 | 0.038950471 | 0.035815683 | 0.11378874 | 0.122453018 |
| HAT1 | Epi3 | turquoise | 0.085636706 | 0.068677172 | 0.050899236 | 0.215199404 | 0.033817651 | 0.020333094 | 0.075065797 | 0.070405135 | 0.126173922 |
| CIR1 | Epi3 | turquoise | 0.085883117 | 0.03865761 | 0.052893042 | 0.145414512 | 0.053515834 | 0.01591904 | 0.079305523 | 0.050846581 | 0.091207221 |
| ATP5G3 | Epi3 | turquoise | 0.208158616 | 0.090110754 | 0.189681039 | 0.291465395 | 0.135138096 | 0.053025131 | 0.148396854 | 0.115541401 | 0.230982862 |
| HIBCH | Epi3 | turquoise | 0.057944538 | 0.040010672 | 0.044785394 | 0.151065976 | 0.022798939 | 0.003604483 | 0.023819818 | 0.060927159 | 0.087707313 |
| SF3B1 | Epi3 | turquoise | 0.077762396 | 0.054332033 | 0.043869896 | 0.116007944 | 0.023428548 | 0.041285233 | 0.044048799 | 0.046564621 | 0.055510988 |
| HSPD1 | Epi3 | turquoise | 0.118859961 | 0.1144384 | 0.054521438 | 0.161792362 | 0.063242653 | 0.019720633 | 0.092161937 | 0.047592836 | 0.096068946 |
| HSPE1 | Epi3 | turquoise | 0.218771353 | 0.180371012 | 0.178075006 | 0.252580558 | 0.083839877 | 0.063645942 | 0.095008111 | 0.163785398 | 0.144799568 |
| MOB4 | Epi3 | turquoise | 0.097241798 | 0.061528917 | 0.061979671 | 0.185052314 | 0.04670564 | 0.028874255 | 0.087977389 | 0.049738718 | 0.113602775 |
| BZW1 | Epi3 | turquoise | 0.103944594 | 0.08826401 | 0.056825113 | 0.17739609 | 0.042067042 | 0.050838588 | 0.089268966 | 0.030690387 | 0.127170125 |
| NDUFB3 | Epi3 | turquoise | 0.151950572 | 0.055350507 | 0.142335981 | 0.220751304 | 0.079502303 | 0.014123725 | 0.091383227 | 0.113416489 | 0.176779531 |
| NOP58 | Epi3 | turquoise | 0.066107755 | 0.034924977 | 0.02128241 | 0.106295941 | 0.028538382 | 0.020794467 | 0.054512656 | 0.020215767 | 0.064525 |
| CYP20A1 | Epi3 | turquoise | 0.075316762 | 0.055339216 | 0.046612932 | 0.081150963 | 0.005855108 | 0.060109791 | 0.051921757 | 0.037813663 | 0.050121686 |
| SPAG16 | Epi3 | turquoise | 0.072213195 | 0.065991881 | 0.037366463 | 0.092302682 | 0.049058539 | 0.013602295 | 0.049002041 | 0.055653947 | 0.035143458 |
| XRCC5 | Epi3 | turquoise | 0.124499105 | 0.054413054 | 0.066722409 | 0.178979322 | 0.044570131 | 0.0348648 | 0.104681501 | 0.048686352 | 0.124960832 |
| AAMP | Epi3 | turquoise | 0.068360506 | 0.072115094 | 0.013243204 | 0.141002819 | 0.010103016 | 0.001763835 | 0.076119503 | 0.045645164 | 0.055510015 |
| PNKD | Epi3 | turquoise | 0.15957336 | 0.085763828 | 0.108330396 | 0.18205657 | 0.090953424 | 0.037073659 | 0.115354235 | 0.117928894 | 0.092462249 |
| MRPL44 | Epi3 | turquoise | 0.034738701 | 0.018160759 | 0.003212936 | 0.091663105 | 0.019560283 | -0.000544156 | 0.077595306 | 0.008818319 | 0.039336401 |
| SP100 | Epi3 | turquoise | 0.068495622 | 0.070896516 | 0.039890008 | 0.091154563 | 0.04097435 | 0.021092502 | 0.035882468 | 0.027684214 | 0.04281101 |
| ITM2C | Epi3 | turquoise | 0.097356118 | 0.052088657 | 0.122666322 | 0.149358686 | 0.040181353 | 0.030895141 | -0.005886259 | 0.146760822 | 0.106980419 |
| NCL | Epi3 | turquoise | 0.163567385 | 0.065086361 | 0.098336431 | 0.165738225 | 0.058223142 | 0.070662101 | 0.083236492 | 0.081034244 | 0.118553371 |
| IQCA1 | Epi3 | turquoise | 0.067040278 | 0.030456083 | 0.039324395 | 0.098573332 | 0.011293423 | 0.029114624 | 0.019642254 | 0.035195192 | 0.076895109 |
| COPS8 | Epi3 | turquoise | 0.076915067 | 0.056985011 | 0.059093499 | 0.136663939 | 0.024470956 | 0.031784171 | 0.063855673 | 0.060687536 | 0.084008661 |
| ILKAP | Epi3 | turquoise | 0.081521233 | 0.036221568 | 0.069486722 | 0.108464087 | 0.032262881 | 0.025871823 | 0.029417933 | 0.088179593 | 0.067654711 |
| NDUFA10 | Epi3 | turquoise | 0.069659337 | 0.049620902 | 0.030782914 | 0.114040051 | 0.038255677 | 0.003444327 | 0.08206661 | 0.031687055 | 0.070951829 |
| DTYMK | Epi3 | turquoise | 0.076709169 | 0.061360025 | 0.066443036 | 0.1041178 | 0.033761073 | 0.01758888 | 0.033311712 | 0.072242358 | 0.066481161 |
| CRBN | Epi3 | turquoise | 0.078612809 | 0.023197182 | 0.058691705 | 0.097998285 | 0.041498846 | 0.03396462 | 0.031375851 | 0.054230087 | 0.078738088 |
| OGG1 | Epi3 | turquoise | 0.067767084 | 0.033266029 | 0.106936315 | 0.097120801 | 0.028542456 | 0.033911242 | 0.002501809 | 0.111352076 | 0.066217576 |
| RPUSD3 | Epi3 | turquoise | 0.050350468 | 0.035230204 | 0.033409247 | 0.093920755 | 0.018693737 | 0.004090389 | 0.04370026 | 0.037428535 | 0.048479637 |
| EMC3 | Epi3 | turquoise | 0.10798834 | 0.098020928 | 0.066578193 | 0.198950145 | -0.004757668 | 0.036847741 | 0.043345511 | 0.112312126 | 0.093211794 |
| SEC13 | Epi3 | turquoise | 0.072937395 | 0.055769485 | 0.038248906 | 0.122922642 | 0.01943025 | 0.018684773 | 0.028941557 | 0.032341662 | 0.054484223 |
| CCDC174 | Epi3 | turquoise | 0.033116042 | 0.026421254 | 0.001374352 | 0.086266226 | -0.015274599 | 0.009525014 | 0.031883155 | 0.023372113 | 0.045381576 |
| DYNC1LI1 | Epi3 | turquoise | 0.077131142 | 0.109007257 | 0.0566175 | 0.09890246 | 0.010668774 | 0.02054071 | -0.026161183 | 0.100006052 | 0.04080649 |
| GLB1 | Epi3 | turquoise | 0.085274322 | 0.071299221 | 0.052006187 | 0.163255724 | 0.012249982 | 0.015366024 | 0.073369719 | 0.06530069 | 0.069999723 |
| ACAA1 | Epi3 | turquoise | 0.084849886 | 0.083575887 | 0.052361119 | 0.128208939 | 0.033818513 | 0.027707911 | 0.067774405 | 0.065179632 | 0.065440327 |
| EIF1B | Epi3 | turquoise | 0.146026714 | 0.099112119 | 0.119997517 | 0.178774041 | 0.075652454 | 0.032271668 | 0.054342177 | 0.146049959 | 0.113979208 |
| HIGD1A | Epi3 | turquoise | 0.190891361 | 0.160391493 | 0.178455405 | 0.189900249 | 0.074294116 | 0.04884269 | 0.042802522 | 0.183509628 | 0.107664294 |
| EXOSC7 | Epi3 | turquoise | 0.067376423 | 0.039806856 | 0.025610296 | 0.125546427 | 0.025669745 | 0.018759002 | 0.08377423 | 0.013296766 | 0.072230312 |
| ELP6 | Epi3 | turquoise | 0.056006343 | 0.034524886 | 0.033323699 | 0.109943191 | 0.020542872 | 0.018300896 | 0.06568789 | 0.02259348 | 0.078943989 |
| UQCRC1 | Epi3 | turquoise | 0.068657833 | 0.073772097 | 0.024877435 | 0.152815408 | 0.006481605 | -5.23E-05 | 0.049406375 | 0.044906258 | 0.077055494 |
| P4HTM | Epi3 | turquoise | 0.05963409 | 0.065010979 | 0.015305347 | 0.119087929 | 0.006904108 | 0.009380716 | 0.049250949 | 0.039895708 | 0.029963739 |
| NDUFAF3 | Epi3 | turquoise | 0.187296499 | 0.12224252 | 0.167194883 | 0.254605643 | 0.106864521 | 0.045519988 | 0.097192227 | 0.132263744 | 0.167435455 |
| APEH | Epi3 | turquoise | 0.060696157 | 0.023791374 | 0.052383916 | 0.101439827 | 0.045548053 | 0.025785618 | 0.064138188 | 0.029588837 | 0.065074277 |
| CYB561D2 | Epi3 | turquoise | 0.059150546 | 0.041242452 | 0.028961431 | 0.094781639 | -0.00689203 | 0.024312285 | 0.035159515 | 0.056569015 | 0.047863348 |
| MANF | Epi3 | turquoise | 0.103894996 | 0.096731039 | 0.046094381 | 0.18587401 | 0.047984406 | 0.022758188 | 0.145636525 | 0.016410273 | 0.125080782 |
| TEX264 | Epi3 | turquoise | 0.068641301 | 0.102454897 | 0.01899138 | 0.143559326 | 0.004702768 | 0.03924934 | 0.057231609 | 0.057591432 | 0.040795777 |
| ABHD14A | Epi3 | turquoise | 0.141928454 | 0.097097261 | 0.132186079 | 0.164149399 | 0.060499285 | 0.025914071 | 0.012974547 | 0.155363105 | 0.101877306 |
| TWF2 | Epi3 | turquoise | 0.074463944 | 0.102936117 | 0.047407434 | 0.121037991 | 0.03130873 | 0.012564772 | 0.036354836 | 0.060585478 | 0.071228135 |
| GNL3 | Epi3 | turquoise | 0.027863984 | 0.001531294 | 0.016193312 | 0.050069781 | 0.01367925 | -0.004868238 | 0.030369226 | -0.005964961 | 0.036418864 |
| GLT8D1 | Epi3 | turquoise | 0.046016717 | 0.065109665 | 0.005066022 | 0.095713728 | -0.001780143 | 0.029988351 | 0.051012252 | 0.034805034 | 0.040854934 |
| SPCS1 | Epi3 | turquoise | 0.229530675 | 0.13106025 | 0.224934894 | 0.328711776 | 0.112077432 | 0.053932823 | 0.12157164 | 0.207700635 | 0.23415786 |
| ARF4 | Epi3 | turquoise | 0.225858055 | 0.132275825 | 0.168095342 | 0.246862945 | 0.123519613 | 0.056561589 | 0.121965837 | 0.119320523 | 0.193652802 |
| PDHB | Epi3 | turquoise | 0.075107997 | 0.069772474 | 0.051593614 | 0.143424384 | 0.043513623 | 0.017661829 | 0.057265164 | 0.049453566 | 0.083966218 |
| PSMD6 | Epi3 | turquoise | 0.040673206 | 0.063268711 | 0.019243992 | 0.129826551 | -0.009034567 | 0.017608646 | 0.047469518 | 0.054510787 | 0.069614979 |
| UBA3 | Epi3 | turquoise | 0.029759125 | 0.038814589 | 0.014046291 | 0.100163952 | -0.015496936 | 0.020141736 | 0.037798289 | 0.029894058 | 0.032233591 |
| ARL6IP5 | Epi3 | turquoise | 0.196498262 | 0.223989787 | 0.162296296 | 0.277749619 | 0.025119987 | 0.0810305 | 0.016469087 | 0.251465456 | 0.109095524 |
| C3orf38 | Epi3 | turquoise | 0.036940221 | 0.023482308 | 0.013217422 | 0.096457591 | 0.023200991 | 0.006541738 | 0.036944572 | 0.012741732 | 0.058617969 |
| ST3GAL6 | Epi3 | turquoise | 0.081217426 | 0.077717926 | 0.111110935 | 0.092759846 | 0.018543818 | 0.022885077 | -0.039028921 | 0.136918007 | 0.065512739 |
| NIT2 | Epi3 | turquoise | 0.066543323 | 0.047590012 | 0.046695842 | 0.162614234 | 0.018740382 | 0.017239793 | 0.081199146 | 0.043874829 | 0.100985141 |
| TFG | Epi3 | turquoise | 0.095756531 | 0.075061529 | 0.047439339 | 0.148154412 | 0.023113045 | 0.016199154 | 0.065053195 | 0.06352051 | 0.076599525 |
| CD47 | Epi3 | turquoise | 0.166685414 | 0.167309791 | 0.095152764 | 0.248542883 | 0.083981506 | 0.134866723 | 0.109273164 | 0.077234424 | 0.172632995 |
| TIMMDC1 | Epi3 | turquoise | 0.100600317 | 0.044615646 | 0.077699992 | 0.103863708 | 0.051027419 | 0.046195206 | 0.052334912 | 0.076027147 | 0.061291402 |
| NDUFB4 | Epi3 | turquoise | 0.21766861 | 0.133694766 | 0.195053612 | 0.308042674 | 0.155483252 | 0.069923159 | 0.135035663 | 0.144013429 | 0.242194346 |
| FAM162A | Epi3 | turquoise | 0.142126901 | 0.061700028 | 0.165830462 | 0.163828005 | 0.133238354 | 0.025758746 | 0.052893925 | 0.099018416 | 0.166984469 |
| SEC22A | Epi3 | turquoise | 0.040098926 | 0.05116342 | 0.035741934 | 0.1242003 | 0.007576537 | 0.012268662 | 0.033686651 | 0.055779306 | 0.053815933 |
| SLC41A3 | Epi3 | turquoise | 0.061601816 | 0.020563668 | 0.032689406 | 0.097824569 | 0.028071131 | 0.016460729 | 0.052653908 | 0.02564783 | 0.076550451 |
| RAB7A | Epi3 | turquoise | 0.158241307 | 0.122565139 | 0.118072745 | 0.208549226 | 0.077681998 | 0.03696524 | 0.092188096 | 0.115781521 | 0.132438031 |
| CNBP | Epi3 | turquoise | 0.192144023 | 0.114552327 | 0.117876922 | 0.182497498 | 0.129108926 | 0.05146547 | 0.08942214 | 0.091906259 | 0.129791573 |
| MRPL3 | Epi3 | turquoise | 0.09061133 | 0.065007479 | 0.079060805 | 0.139261691 | 0.038611556 | 0.017753209 | 0.033667189 | 0.080739583 | 0.090458425 |
| ARMC8 | Epi3 | turquoise | 0.059865179 | 0.018767576 | 0.058995118 | 0.072607648 | 0.015437499 | 0.014406972 | 0.035200745 | 0.043986567 | 0.066799181 |
| MRPS22 | Epi3 | turquoise | 0.027677534 | 0.03029123 | 0.014436771 | 0.089875594 | -0.0185256 | 0.014271088 | 0.033420686 | 0.019160535 | 0.034936074 |
| COPB2 | Epi3 | turquoise | 0.080886689 | 0.059479762 | 0.069060972 | 0.140134143 | 0.043768167 | 0.02537855 | 0.059626201 | 0.04791331 | 0.105487556 |
| ATP1B3 | Epi3 | turquoise | 0.10363633 | 0.029754533 | 0.085890467 | 0.147100629 | 0.078167873 | 0.046374845 | 0.096949098 | 0.015126156 | 0.154728076 |
| PLSCR1 | Epi3 | turquoise | 0.087536274 | 0.140801893 | 0.05341353 | 0.110274424 | 0.014301816 | 0.021347206 | 0.036370295 | 0.061737255 | 0.037233825 |
| GYG1 | Epi3 | turquoise | 0.068226518 | 0.054609964 | 0.080314393 | 0.092603953 | 0.031235011 | 0.023982977 | -0.007424715 | 0.093902529 | 0.068244688 |
| RNF13 | Epi3 | turquoise | 0.126387196 | 0.112553958 | 0.110772275 | 0.159914659 | 0.025978138 | 0.033359971 | 0.019609438 | 0.153610772 | 0.075967104 |
| SELT | Epi3 | turquoise | 0.204444953 | 0.134818153 | 0.20684585 | 0.223569775 | 0.089673216 | 0.091006604 | 0.040356523 | 0.210451627 | 0.14766939 |
| MFSD1 | Epi3 | turquoise | 0.065328964 | 0.074970892 | 0.029666728 | 0.089463792 | 0.003386748 | 0.020589494 | 0.002841961 | 0.059588086 | 0.028874303 |
| BCHE | Epi3 | turquoise | 0.089440503 | -0.015712963 | 0.158173146 | 0.189574439 | 0.023014496 | 0.007385529 | 0.027938562 | 0.141219252 | 0.19677324 |
| SEC62 | Epi3 | turquoise | 0.234486235 | 0.138600984 | 0.14370488 | 0.278671789 | 0.124572386 | 0.068408313 | 0.178750214 | 0.118367433 | 0.168283299 |
| MRPL47 | Epi3 | turquoise | 0.086833447 | 0.045677652 | 0.060903179 | 0.148510456 | 0.058707487 | 0.003194281 | 0.092554745 | 0.032143817 | 0.108059745 |
| NDUFB5 | Epi3 | turquoise | 0.140829459 | 0.043975446 | 0.094959034 | 0.149849184 | 0.137190438 | 0.054958246 | 0.10310158 | 0.043637126 | 0.130559701 |
| PSMD2 | Epi3 | turquoise | 0.062659065 | 0.062788653 | 0.04032064 | 0.104393208 | 0.019386178 | 0.028465715 | 0.056336721 | 0.049676049 | 0.05162849 |
| MAGEF1 | Epi3 | turquoise | 0.089067148 | 0.068538194 | 0.081073885 | 0.138880269 | 0.031082943 | 0.027420766 | 0.057442247 | 0.067498326 | 0.076048482 |
| DNAJB11 | Epi3 | turquoise | 0.102989401 | 0.06263353 | 0.077506523 | 0.153393349 | 0.030275013 | 0.038078923 | 0.078618591 | 0.064112066 | 0.100693667 |
| MAEA | Epi3 | turquoise | 0.050018386 | 0.032864861 | 0.013657142 | 0.08309412 | -0.004043763 | 0.010998718 | 0.036403712 | 0.011575086 | 0.063229171 |
| MFSD10 | Epi3 | turquoise | 0.088441687 | 0.068259043 | 0.090939245 | 0.140496622 | 0.048447611 | 0.034870497 | 0.04037976 | 0.077593603 | 0.098384199 |
| LRPAP1 | Epi3 | turquoise | 0.099825286 | 0.129194159 | 0.059546595 | 0.268752947 | 0.015986904 | 0.026035907 | 0.099893001 | 0.101280016 | 0.150481202 |
| TMEM128 | Epi3 | turquoise | 0.051632989 | 0.045169117 | 0.039919846 | 0.119432024 | 0.02145654 | -0.009315762 | 0.043449352 | 0.039006378 | 0.077826206 |
| MRFAP1 | Epi3 | turquoise | 0.189303762 | 0.098804908 | 0.156360633 | 0.209354837 | 0.096468721 | 0.055688422 | 0.083181941 | 0.147088829 | 0.142506868 |
| GRPEL1 | Epi3 | turquoise | 0.045063695 | 0.048407872 | 0.024516492 | 0.101673258 | 0.019008346 | 0.012224546 | 0.035763816 | 0.038879216 | 0.051173171 |
| WDR1 | Epi3 | turquoise | 0.11305071 | 0.083839623 | 0.108473162 | 0.130912274 | 0.073196517 | 0.026817725 | 0.057789404 | 0.055466761 | 0.111175584 |
| QDPR | Epi3 | turquoise | 0.097543811 | 0.01556545 | 0.050552557 | 0.104476394 | 0.050470768 | 0.045591659 | 0.079426343 | 0.056513097 | 0.048983512 |
| LAP3 | Epi3 | turquoise | 0.044899782 | 0.03445748 | 0.01701293 | 0.13238999 | 0.01745759 | -0.010349205 | 0.076533191 | 0.007928789 | 0.078920419 |
| RBPJ | Epi3 | turquoise | 0.12063826 | 0.069792068 | 0.070448272 | 0.103666366 | 0.077648713 | 0.017762277 | 0.094841918 | 0.040439473 | 0.071709775 |
| GNPDA2 | Epi3 | turquoise | 0.070014939 | 0.037203442 | 0.035088443 | 0.109753627 | -0.002586232 | 0.029477041 | 0.043000555 | 0.066691511 | 0.036523773 |
| COMMD8 | Epi3 | turquoise | 0.069294924 | 0.029141381 | 0.026114245 | 0.099927439 | 0.026219214 | 0.014432634 | 0.066018937 | 0.02081181 | 0.065122489 |
| OCIAD1 | Epi3 | turquoise | 0.181003417 | 0.104592562 | 0.181183198 | 0.284421373 | 0.049705083 | 0.035910918 | 0.076163902 | 0.201248974 | 0.183609527 |
| FIP1L1 | Epi3 | turquoise | 0.064959878 | 0.026797983 | 0.052848398 | 0.084591487 | 0.030886744 | 0.037386765 | 0.049608603 | 0.046096097 | 0.064471372 |
| RCHY1 | Epi3 | turquoise | 0.101165387 | 0.054578085 | 0.081197286 | 0.123135802 | 0.080095608 | 0.035704768 | 0.086131309 | 0.041469027 | 0.111332694 |
| NUP54 | Epi3 | turquoise | 0.075983383 | 0.046087845 | 0.040503565 | 0.120097038 | 0.023690986 | 0.026373789 | 0.061012512 | 0.031716747 | 0.073178806 |
| MRPL1 | Epi3 | turquoise | 0.058932305 | 0.03383413 | 0.0209041 | 0.148944784 | 0.024048127 | 0.017879581 | 0.069726487 | 0.021756764 | 0.085410471 |
| ANXA3 | Epi3 | turquoise | 0.07612051 | 0.008802442 | 0.05254758 | 0.162618121 | 0.044620853 | 0.009877583 | 0.145870014 | -0.042394528 | 0.147665598 |
| HNRNPDL | Epi3 | turquoise | 0.145661544 | 0.093256247 | 0.097105888 | 0.148023003 | 0.121967388 | 0.071172779 | 0.098270773 | 0.048606809 | 0.127228109 |
| COPS4 | Epi3 | turquoise | 0.063682914 | 0.028470417 | 0.039876404 | 0.174950479 | -0.000519019 | 0.028480147 | 0.088626096 | 0.066408674 | 0.105522602 |
| MRPS18C | Epi3 | turquoise | 0.090651199 | 0.045432333 | 0.067821796 | 0.128042428 | 0.036936143 | 0.033225857 | 0.073034873 | 0.053517918 | 0.091443027 |
| HSD17B11 | Epi3 | turquoise | 0.073956278 | 0.089503633 | 0.034253421 | 0.125608589 | 0.014720787 | 0.002114481 | 0.027255952 | 0.070342659 | 0.052892465 |
| EIF4E | Epi3 | turquoise | 0.139146725 | 0.118732528 | 0.111149365 | 0.263611791 | 0.029620345 | 0.013782752 | 0.055207393 | 0.149264748 | 0.125528385 |
| ADH5 | Epi3 | turquoise | 0.085776468 | 0.011118716 | 0.046334532 | 0.179445777 | 0.025319328 | -0.003719285 | 0.093075791 | 0.046049883 | 0.123968562 |
| BDH2 | Epi3 | turquoise | 0.140803718 | 0.085469755 | 0.112355895 | 0.159666608 | 0.083677591 | 0.022600547 | 0.090389209 | 0.087426919 | 0.127753862 |
| PPA2 | Epi3 | turquoise | 0.105238951 | 0.060663053 | 0.086611551 | 0.237495306 | 0.053124085 | 0.009711774 | 0.092946107 | 0.045708468 | 0.173156457 |
| AIMP1 | Epi3 | turquoise | 0.099461795 | 0.083637544 | 0.078598282 | 0.184184966 | 0.040650712 | 0.01892976 | 0.032888215 | 0.087200088 | 0.107709462 |
| PAPSS1 | Epi3 | turquoise | 0.045560827 | 0.025046952 | 0.019347976 | 0.098492266 | -4.33E-05 | 0.014774731 | 0.062348598 | 0.043437818 | 0.059021467 |
| OSTC | Epi3 | turquoise | 0.195546191 | 0.096901073 | 0.157885932 | 0.22403661 | 0.139805928 | 0.061056489 | 0.130775455 | 0.102423516 | 0.203830068 |
| CFI | Epi3 | turquoise | 0.113248344 | 0.168085419 | 0.077994174 | 0.224505697 | 0.037825809 | 0.030816361 | 0.085678633 | 0.066968449 | 0.147450301 |
| GAR1 | Epi3 | turquoise | 0.046712065 | 0.052062089 | 0.013721489 | 0.070866178 | 0.011728405 | 0.033041062 | 0.037210939 | 0.039414247 | 0.030240059 |
| LARP7 | Epi3 | turquoise | 0.113152742 | 0.055933306 | 0.103538605 | 0.1065263 | 0.064499562 | 0.049702351 | 0.051823137 | 0.05911269 | 0.0736637 |
| ANXA5 | Epi3 | turquoise | 0.208128067 | 0.113607032 | 0.192179287 | 0.348453633 | 0.087751308 | 0.02976687 | 0.119288505 | 0.148788921 | 0.275109652 |
| NDUFC1 | Epi3 | turquoise | 0.232745146 | 0.122993864 | 0.272480258 | 0.255847358 | 0.193481827 | 0.08073568 | 0.064483571 | 0.186614226 | 0.231416653 |
| MGST2 | Epi3 | turquoise | 0.109899763 | 0.058950132 | 0.089120852 | 0.15886358 | 0.036993666 | 0.039072291 | 0.05582478 | 0.085612686 | 0.122965518 |
| SCOC | Epi3 | turquoise | 0.152163083 | 0.089584541 | 0.124979448 | 0.154414635 | 0.091630978 | 0.039827914 | 0.092726489 | 0.101961917 | 0.114924624 |
| ANAPC10 | Epi3 | turquoise | 0.029555376 | 0.034317535 | 0.015564747 | 0.084653955 | 0.002347401 | 0.014070472 | 0.041145902 | 0.025620819 | 0.042214307 |
| ARFIP1 | Epi3 | turquoise | 0.076078691 | 0.058709493 | 0.052561167 | 0.079118424 | 0.021309166 | 0.000334605 | 0.048518444 | 0.056030182 | 0.048578055 |
| PLRG1 | Epi3 | turquoise | 0.042631299 | 0.045555618 | 0.02048902 | 0.096548591 | 0.009810547 | 0.022126389 | 0.027209132 | 0.036416926 | 0.054294954 |
| MAP9 | Epi3 | turquoise | 0.066417113 | 0.000167914 | 0.051077268 | 0.082411431 | 0.040958414 | -0.004982916 | 0.051219705 | 0.026119787 | 0.077606431 |
| PPID | Epi3 | turquoise | 0.039068097 | 0.017255917 | 0.025718352 | 0.087190707 | 0.004287739 | 0.005241322 | 0.03103013 | 0.016896261 | 0.05176786 |
| MSMO1 | Epi3 | turquoise | 0.087013604 | 0.137061719 | 0.059509654 | 0.147568219 | 0.001143386 | 0.044772539 | 0.012986174 | 0.088404748 | 0.075621921 |
| C4orf27 | Epi3 | turquoise | 0.068508243 | 0.050906892 | 0.037121242 | 0.104630941 | 0.0289998 | 0.004428388 | 0.047697938 | 0.042073542 | 0.05138716 |
| HMGB2 | Epi3 | turquoise | 0.068824278 | 0.057227577 | -0.001794868 | 0.103197622 | -0.012455013 | 0.026473212 | 0.075573079 | 0.03650063 | 0.013496432 |
| SLC25A4 | Epi3 | turquoise | 0.057967142 | 0.04763212 | 0.068695035 | 0.129476045 | 0.009207944 | 0.013528587 | 0.025716646 | 0.085651848 | 0.058483036 |
| UFSP2 | Epi3 | turquoise | 0.087564794 | 0.064250666 | 0.065729415 | 0.114378213 | 0.042337631 | 0.055834349 | 0.059977094 | 0.064906516 | 0.071154326 |
| FRG1 | Epi3 | turquoise | 0.063348701 | 0.065399256 | 0.04510608 | 0.156490626 | -0.002655208 | 0.017607531 | 0.026178087 | 0.067338 | 0.083163393 |
| SDHA | Epi3 | turquoise | 0.020666137 | 0.033577473 | -0.036650374 | 0.108976104 | -0.028110954 | -0.011972934 | 0.072438276 | -0.010775063 | 0.018782247 |
| PDCD6 | Epi3 | turquoise | 0.1143465 | 0.094797905 | 0.092851519 | 0.214946986 | 0.065215382 | 0.051555687 | 0.08513151 | 0.085080729 | 0.132626787 |
| MED10 | Epi3 | turquoise | 0.065885268 | 0.041881472 | 0.053398916 | 0.1138597 | 0.028902132 | 0.009250089 | 0.051292281 | 0.073517062 | 0.067630159 |
| CCT5 | Epi3 | turquoise | 0.050286168 | 0.038601925 | 0.03319558 | 0.148409778 | 0.019297299 | 0.012195874 | 0.088757601 | 0.004609536 | 0.10000401 |
| ZNF622 | Epi3 | turquoise | 0.043454923 | 0.056857069 | 0.002702877 | 0.094709037 | -0.005741789 | 0.019845078 | 0.037675893 | 0.028731279 | 0.04719484 |
| SUB1 | Epi3 | turquoise | 0.244377416 | 0.114858307 | 0.220143731 | 0.257206193 | 0.163990304 | 0.0694347 | 0.131530464 | 0.156949554 | 0.228410229 |
| BRIX1 | Epi3 | turquoise | 0.063954417 | 0.052111577 | 0.048211272 | 0.132373084 | 0.031182788 | -0.004200207 | 0.072086863 | 0.02897838 | 0.091548071 |
| FBXO4 | Epi3 | turquoise | 0.027491591 | 0.017056018 | -0.015013284 | 0.091254094 | -0.001974611 | 0.041318248 | 0.064099897 | 0.002156609 | 0.024017096 |
| SEPP1 | Epi3 | turquoise | 0.089847874 | 0.092548317 | 0.017015601 | 0.162433665 | 0.087436923 | 0.035987221 | 0.094457958 | 0.024148839 | 0.099338414 |
| C5orf28 | Epi3 | turquoise | 0.052637369 | 0.030712591 | 0.041841093 | 0.099267913 | -0.009479282 | 0.002726474 | 0.052986704 | 0.061972508 | 0.046593084 |
| NDUFS4 | Epi3 | turquoise | 0.096494754 | 0.030855629 | 0.081029189 | 0.156675242 | 0.086879934 | 0.018300179 | 0.055114098 | 0.065113878 | 0.109442578 |
| GPX8 | Epi3 | turquoise | 0.065404007 | 0.064668392 | 0.033667335 | 0.116883868 | 0.006787638 | 0.005050729 | 0.057921812 | 0.049938929 | 0.045291801 |
| DIMT1 | Epi3 | turquoise | 0.05808743 | 0.032896238 | 0.020915248 | 0.088830938 | 0.038448353 | 0.006713307 | 0.063049732 | -0.004961424 | 0.054643297 |
| MRPS36 | Epi3 | turquoise | 0.14757121 | 0.061917856 | 0.13593851 | 0.143888082 | 0.129165435 | 0.033919903 | 0.06913638 | 0.064861171 | 0.135384063 |
| AK6 | Epi3 | turquoise | 0.092806816 | 0.038991491 | 0.104665299 | 0.128534002 | 0.054937864 | 0.011104806 | 0.04820221 | 0.083879999 | 0.093580805 |
| HEXB | Epi3 | turquoise | 0.055288336 | 0.087230228 | 0.012557726 | 0.171150993 | -0.023023686 | -0.00795276 | 0.042638868 | 0.072434551 | 0.055187054 |
| SCAMP1 | Epi3 | turquoise | 0.088051484 | 0.094028503 | 0.064782967 | 0.125625232 | 0.033162328 | 0.011140633 | 0.027030645 | 0.088250459 | 0.070236517 |
| CETN3 | Epi3 | turquoise | 0.072679667 | 0.051354242 | 0.052911639 | 0.150156284 | 0.019240504 | 0.019787138 | 0.051181383 | 0.069388096 | 0.088343015 |
| REEP5 | Epi3 | turquoise | 0.143191276 | 0.091614858 | 0.089762926 | 0.315844179 | 0.054759634 | 0.039987224 | 0.180745558 | 0.056405755 | 0.217444358 |
| COMMD10 | Epi3 | turquoise | 0.071953152 | 0.059099808 | 0.04825789 | 0.15893853 | 0.016806134 | 0.02238948 | 0.047947069 | 0.076233099 | 0.092946851 |
| HSD17B4 | Epi3 | turquoise | 0.029598842 | 0.002000421 | 0.007787531 | 0.105199983 | -0.002517518 | 0.011132793 | 0.061629208 | 0.001734654 | 0.055481962 |
| SNX2 | Epi3 | turquoise | 0.080651825 | 0.060729671 | 0.010074626 | 0.187984068 | -0.005056419 | 0.024864599 | 0.119921092 | 0.033403659 | 0.082234553 |
| PPIC | Epi3 | turquoise | 0.182073321 | 0.064131145 | 0.225353544 | 0.312563222 | 0.111663385 | 0.049124685 | 0.092255611 | 0.140938252 | 0.290082602 |
| ALDH7A1 | Epi3 | turquoise | 0.059721846 | 0.04517228 | -0.003657899 | 0.124101222 | -0.001696325 | 0.036924829 | 0.09545837 | 0.022963695 | 0.054244792 |
| C5orf15 | Epi3 | turquoise | 0.108722121 | 0.150267301 | 0.056803468 | 0.217241667 | -0.017851293 | 0.019923088 | 0.037834255 | 0.100340114 | 0.107568746 |
| VDAC1 | Epi3 | turquoise | 0.153721085 | 0.081708664 | 0.12675193 | 0.192247332 | 0.103553607 | 0.064235778 | 0.086855413 | 0.097673537 | 0.158909585 |
| SKP1 | Epi3 | turquoise | 0.25417451 | 0.118844411 | 0.205271901 | 0.476344073 | 0.116452672 | 0.043839792 | 0.205108636 | 0.188450378 | 0.322486439 |
| SAR1B | Epi3 | turquoise | 0.075635805 | 0.089821516 | 0.069385313 | 0.143289529 | -0.005618041 | 0.028409082 | 0.029695394 | 0.099223966 | 0.068236467 |
| TXNDC15 | Epi3 | turquoise | 0.049898283 | 0.012611051 | 0.028240478 | 0.124305919 | 0.004213197 | 0.000898603 | 0.048612787 | 0.043991168 | 0.055051647 |
| SMAD5 | Epi3 | turquoise | 0.071012082 | 0.028210588 | 0.054070771 | 0.0667176 | 0.040045324 | 0.017958771 | 0.038702957 | 0.055820402 | 0.049405938 |
| NME5 | Epi3 | turquoise | 0.090225497 | 0.062206279 | 0.053407018 | 0.151560182 | 0.015973313 | 0.001381189 | 0.075099805 | 0.057799032 | 0.08405797 |
| HSPA9 | Epi3 | turquoise | 0.059428796 | 0.05596338 | 0.003013561 | 0.09959562 | -0.01081522 | 0.000253628 | 0.06544958 | 0.012133911 | 0.033622009 |
| SIL1 | Epi3 | turquoise | 0.094932801 | 0.113893749 | 0.05025879 | 0.159945806 | 0.005456066 | 0.006211734 | 0.046272238 | 0.083515936 | 0.062256882 |
| PAIP2 | Epi3 | turquoise | 0.127051434 | 0.041627514 | 0.067528883 | 0.142592116 | 0.060959317 | 0.023654355 | 0.104623798 | 0.060139491 | 0.073604762 |
| PFDN1 | Epi3 | turquoise | 0.119197338 | 0.06627229 | 0.102459406 | 0.136696061 | 0.068486424 | 0.008849987 | 0.091360881 | 0.074507804 | 0.107874556 |
| IK | Epi3 | turquoise | 0.080242942 | 0.067006543 | 0.030810262 | 0.133277752 | 0.006610785 | 0.035575981 | 0.053595962 | 0.079602839 | 0.058935446 |
| ZMAT2 | Epi3 | turquoise | 0.090739677 | 0.077465962 | 0.053158759 | 0.115800731 | 0.039849061 | 0.01748668 | 0.049116921 | 0.053008188 | 0.070060894 |
| NDFIP1 | Epi3 | turquoise | 0.20367933 | 0.178192002 | 0.152989883 | 0.291239277 | 0.017812054 | 0.058151499 | 0.062716068 | 0.235320998 | 0.135053213 |
| ATOX1 | Epi3 | turquoise | 0.185787032 | 0.086846527 | 0.168153316 | 0.167943482 | 0.098068682 | 0.048906151 | 0.112618124 | 0.152690287 | 0.105683885 |
| MRPL22 | Epi3 | turquoise | 0.087886614 | 0.066131227 | 0.072047806 | 0.165548077 | 0.042527175 | 0.017419374 | 0.069493626 | 0.050932629 | 0.110592697 |
| MED7 | Epi3 | turquoise | 0.06848341 | 0.056612682 | 0.021165857 | 0.133657104 | 0.022236333 | 0.020157684 | 0.041556142 | 0.035334057 | 0.070424569 |
| TTC1 | Epi3 | turquoise | 0.098116908 | 0.07116025 | 0.089024963 | 0.118734466 | 0.050639405 | 0.044923259 | 0.023526089 | 0.097124189 | 0.067063327 |
| NUDCD2 | Epi3 | turquoise | 0.098104796 | 0.042294352 | 0.087237772 | 0.105812101 | 0.065548261 | 0.023205663 | 0.056388889 | 0.073323137 | 0.086789198 |
| ATP6V0E1 | Epi3 | turquoise | 0.244527985 | 0.203462905 | 0.207552686 | 0.335572717 | 0.101885133 | 0.055084488 | 0.114419382 | 0.211815912 | 0.199261697 |
| LMAN2 | Epi3 | turquoise | 0.103255327 | 0.126993054 | 0.059591301 | 0.251082065 | 0.013226903 | 0.004983513 | 0.079113836 | 0.085170002 | 0.139442474 |
| DBN1 | Epi3 | turquoise | 0.061847754 | 0.112329691 | 0.054866822 | 0.103974786 | -0.019597552 | 0.015768154 | -0.002190468 | 0.091007452 | 0.039486125 |
| TMED9 | Epi3 | turquoise | 0.154950517 | 0.163522688 | 0.11709976 | 0.260242565 | 0.03922292 | 0.033005935 | 0.076861892 | 0.126808228 | 0.136211619 |
| CLK4 | Epi3 | turquoise | 0.03959435 | 0.020601967 | 0.012819185 | 0.098465313 | -0.01191781 | 0.004860176 | 0.022732009 | 0.034414291 | 0.036595034 |
| SQSTM1 | Epi3 | turquoise | 0.161664695 | 0.131545243 | 0.104867821 | 0.27544665 | 0.029123437 | 0.025952306 | 0.076257033 | 0.147128633 | 0.140367782 |
| SERPINB1 | Epi3 | turquoise | 0.079300282 | 0.143476339 | 0.033879992 | 0.160941162 | -0.015588734 | 0.023600361 | 0.036634277 | 0.085591281 | 0.035583281 |
| SERPINB6 | Epi3 | turquoise | 0.112873113 | 0.089168924 | 0.066374435 | 0.148772554 | 0.051261928 | 0.032517793 | 0.052172408 | 0.070584161 | 0.08351249 |
| PSMG4 | Epi3 | turquoise | 0.069133828 | 0.075666366 | 0.037490791 | 0.085221649 | 0.040030585 | 0.016798106 | 0.056515054 | 0.046492066 | 0.029896759 |
| ECI2 | Epi3 | turquoise | 0.085750641 | 0.049900335 | 0.06969336 | 0.116485347 | 0.033653782 | 0.026301136 | 0.051064376 | 0.072939323 | 0.070050623 |
| EEF1E1 | Epi3 | turquoise | 0.088611192 | 0.065000233 | 0.070591473 | 0.126966517 | 0.040114567 | 0.013941295 | 0.04248678 | 0.057208522 | 0.082652059 |
| TMEM14C | Epi3 | turquoise | 0.178575657 | 0.072374869 | 0.17502602 | 0.178064329 | 0.112504089 | 0.063398157 | 0.092993972 | 0.129013504 | 0.154999258 |
| NOL7 | Epi3 | turquoise | 0.08449178 | 0.042884313 | 0.086285723 | 0.094589658 | 0.042508569 | 0.059245931 | 0.029594454 | 0.061327697 | 0.066767431 |
| TPMT | Epi3 | turquoise | 0.106730856 | 0.076723706 | 0.066300235 | 0.150157446 | 0.02531615 | 0.037678597 | 0.079909658 | 0.090569075 | 0.062335601 |
| HLA-A | Epi3 | turquoise | 0.241695494 | 0.530746984 | 0.092052088 | 0.513165692 | -0.046315445 | 0.049631576 | 0.117354976 | 0.247814567 | 0.170439311 |
| ABCF1 | Epi3 | turquoise | 0.084941786 | 0.061502001 | 0.066381455 | 0.065503955 | 0.044313878 | 0.02144095 | 0.005577431 | 0.058497724 | 0.020302157 |
| GPANK1 | Epi3 | turquoise | 0.063511007 | 0.065189146 | 0.019480851 | 0.067980874 | 0.02940536 | 0.016162608 | 0.027809642 | 0.029921003 | 0.032570142 |
| CSNK2B | Epi3 | turquoise | 0.100717701 | 0.045790849 | 0.05006675 | 0.173500115 | 0.094743704 | 0.013551451 | 0.12659621 | 0.037307491 | 0.113358588 |
| NEU1 | Epi3 | turquoise | 0.044497755 | 0.097483519 | 0.007669961 | 0.159041287 | -0.031899437 | 0.006944842 | 0.062080923 | 0.055439623 | 0.047972364 |
| NELFE | Epi3 | turquoise | 0.084505459 | 0.055028676 | 0.052878344 | 0.141079504 | 0.047212351 | 0.020507465 | 0.08414753 | 0.05075084 | 0.091187517 |
| SLC39A7 | Epi3 | turquoise | 0.108708676 | 0.075033459 | 0.079049105 | 0.150973172 | 0.049129187 | 0.014872147 | 0.081759804 | 0.065221939 | 0.105584259 |
| HSD17B8 | Epi3 | turquoise | 0.037215686 | 0.002228558 | 0.009673632 | 0.132633793 | 0.018786927 | -0.014727313 | 0.084993739 | 0.013619724 | 0.07841187 |
| WDR46 | Epi3 | turquoise | 0.05839264 | 0.020753897 | 0.047897192 | 0.101487703 | 0.015649037 | 0.013989533 | 0.032335787 | 0.053650277 | 0.061487893 |
| PFDN6 | Epi3 | turquoise | 0.076825606 | 0.029308614 | 0.054193608 | 0.145822213 | 0.034845531 | -0.002414101 | 0.064955171 | 0.050969609 | 0.077746362 |
| CUTA | Epi3 | turquoise | 0.221341523 | 0.114113649 | 0.199680703 | 0.319889148 | 0.129180815 | 0.06637564 | 0.10413204 | 0.174920915 | 0.220947877 |
| SNRPC | Epi3 | turquoise | 0.095478244 | 0.07092367 | 0.042075898 | 0.186003938 | 0.03181803 | 0.012007499 | 0.095204734 | 0.054600658 | 0.091177749 |
| GLO1 | Epi3 | turquoise | 0.118948169 | 0.088665115 | 0.056757286 | 0.160104502 | 0.061922661 | 0.032811137 | 0.101050309 | 0.051839434 | 0.094468241 |
| OARD1 | Epi3 | turquoise | 0.115892458 | 0.031353228 | 0.104081679 | 0.096128373 | 0.082216532 | 0.041817963 | 0.057128464 | 0.083074492 | 0.079223777 |
| TBCC | Epi3 | turquoise | 0.077122222 | 0.050640472 | 0.040461784 | 0.108918226 | 0.035796409 | 0.022783763 | 0.067504122 | 0.035570457 | 0.070403908 |
| MEA1 | Epi3 | turquoise | 0.107219752 | 0.081797154 | 0.081253968 | 0.149887182 | 0.053775059 | 0.013267332 | 0.079043579 | 0.084032283 | 0.093682374 |
| MRPL2 | Epi3 | turquoise | 0.078796146 | 0.043095947 | 0.097042758 | 0.121901403 | 0.020836919 | 0.022487572 | 0.012091073 | 0.100153112 | 0.076742198 |
| DNPH1 | Epi3 | turquoise | 0.074555324 | 0.111599244 | 0.016862679 | 0.10501332 | 0.039701058 | 0.016012965 | 0.052674667 | 0.023379922 | 0.029095382 |
| MRPS18A | Epi3 | turquoise | 0.062427084 | 0.063706764 | 0.0413653 | 0.129411037 | 0.030654109 | 0.043323579 | 0.07762579 | 0.032284049 | 0.085624889 |
| HSP90AB1 | Epi3 | turquoise | 0.210850561 | 0.111253472 | 0.122048527 | 0.319857264 | 0.138120634 | 0.076711698 | 0.114154276 | 0.131532849 | 0.199019391 |
| SLC35B2 | Epi3 | turquoise | 0.072962312 | 0.060100868 | 0.051268789 | 0.095369974 | 0.022078931 | -0.001841824 | 0.034379456 | 0.036148914 | 0.078950518 |
| ENPP4 | Epi3 | turquoise | 0.040081154 | 0.05979643 | 0.042034794 | 0.068484467 | -0.01576691 | 0.028104006 | -0.025246758 | 0.093069259 | 0.03510241 |
| ENPP5 | Epi3 | turquoise | 0.087610291 | 0.094725656 | 0.045233741 | 0.159881606 | -0.00052422 | -0.011891842 | 0.073953429 | 0.057842249 | 0.071810458 |
| GSTA4 | Epi3 | turquoise | 0.090586715 | 0.086782265 | 0.047940392 | 0.14753308 | 0.018663864 | 0.046704303 | 0.068478756 | 0.06237648 | 0.083680253 |
| BAG2 | Epi3 | turquoise | 0.050611123 | 0.016600226 | 0.023747239 | 0.082589307 | 0.032258487 | 0.012810244 | 0.056638595 | 0.020177061 | 0.050478515 |
| LMBRD1 | Epi3 | turquoise | 0.113237186 | 0.087474724 | 0.087449761 | 0.221863571 | 0.063586995 | 0.062367764 | 0.064618572 | 0.095463518 | 0.155638188 |
| SNX14 | Epi3 | turquoise | 0.095736612 | 0.072057817 | 0.068219054 | 0.097312148 | 0.05410984 | 0.042766645 | 0.028565973 | 0.061274694 | 0.073589367 |
| SLC35A1 | Epi3 | turquoise | 0.050304322 | 0.103999493 | 0.022734653 | 0.116886867 | -0.031848141 | 0.000702018 | 0.028764053 | 0.044674792 | 0.049675608 |
| UFL1 | Epi3 | turquoise | 0.06673929 | 0.023369895 | 0.049447514 | 0.084463922 | 0.009026639 | 0.042693962 | 0.027354731 | 0.042438694 | 0.05968006 |
| C6orf203 | Epi3 | turquoise | 0.030150046 | 0.02585319 | 0.032285647 | 0.080117204 | 0.004034188 | 0.003049518 | 0.007088318 | 0.030704935 | 0.043348817 |
| CD164 | Epi3 | turquoise | 0.127620854 | 0.111589127 | -0.026274314 | 0.264577667 | -0.042837831 | 0.036090774 | 0.222092541 | 0.030383975 | 0.068509355 |
| RPF2 | Epi3 | turquoise | 0.087681042 | 0.010200568 | 0.08723439 | 0.102422161 | 0.055500316 | 0.020339703 | 0.065648961 | 0.033199813 | 0.10492105 |
| HDAC2 | Epi3 | turquoise | 0.087194321 | 0.028216478 | 0.046333105 | 0.120995572 | 0.061710173 | -0.002850019 | 0.111828282 | 0.005831384 | 0.102511779 |
| NT5DC1 | Epi3 | turquoise | 0.071684342 | 0.035831649 | 0.056264156 | 0.102216636 | 0.055615908 | 0.012807208 | 0.061871672 | 0.019080931 | 0.096615998 |
| ASF1A | Epi3 | turquoise | 0.090771977 | 0.033843662 | 0.083283242 | 0.094597484 | 0.039372951 | 0.039643699 | 0.029172669 | 0.073989429 | 0.075996503 |
| SERINC1 | Epi3 | turquoise | 0.107522246 | 0.07627126 | 0.097153721 | 0.125561405 | 0.040291156 | 0.040483215 | 0.029746308 | 0.086970062 | 0.070856214 |
| SMPDL3A | Epi3 | turquoise | 0.091290893 | 0.086073873 | 0.062222527 | 0.15866589 | 0.014292281 | 0.013153627 | 0.036193781 | 0.089090006 | 0.08252177 |
| TRMT11 | Epi3 | turquoise | 0.045073387 | 0.045517423 | 0.028506865 | 0.098521865 | -0.004825547 | -0.007484872 | 0.034720786 | 0.038531469 | 0.051585826 |
| ECHDC1 | Epi3 | turquoise | 0.070272557 | 0.036944293 | 0.03449574 | 0.109660352 | 0.036834272 | 0.018913278 | 0.063420813 | 0.033823975 | 0.068362951 |
| PEX7 | Epi3 | turquoise | 0.059869572 | 0.077944877 | 0.016519777 | 0.101566863 | 0.004903558 | 0.001958112 | 0.017768075 | 0.064327104 | 0.017156262 |
| IFNGR1 | Epi3 | turquoise | 0.097716862 | 0.119796836 | 0.032311521 | 0.215759389 | 0.02563876 | 0.036799343 | 0.103033465 | 0.046008338 | 0.121105132 |
| PERP | Epi3 | turquoise | 0.168505903 | 0.201627687 | 0.057479286 | 0.158978058 | 0.151626176 | 0.060770769 | 0.104505117 | 0.013733745 | 0.107734885 |
| CCDC28A | Epi3 | turquoise | 0.092761693 | 0.054653453 | 0.064720662 | 0.096666387 | 0.037257937 | 0.032823355 | 0.049545645 | 0.065792875 | 0.051535651 |
| AIG1 | Epi3 | turquoise | 0.095613626 | 0.062430083 | 0.059865629 | 0.189390681 | 0.03355964 | 0.007697855 | 0.067779051 | 0.093336271 | 0.10428932 |
| FUCA2 | Epi3 | turquoise | 0.068648027 | 0.059548291 | 0.050395358 | 0.100267023 | 0.014252785 | -0.007344616 | 0.050530179 | 0.045586726 | 0.055977003 |
| SF3B5 | Epi3 | turquoise | 0.165436551 | 0.05315591 | 0.144528781 | 0.182106245 | 0.133907541 | 0.053906364 | 0.119333391 | 0.089278689 | 0.160013815 |
| PPIL4 | Epi3 | turquoise | 0.060706774 | 0.037739947 | 0.029893794 | 0.108295051 | 0.006779072 | 0.035746452 | 0.053644367 | 0.021087176 | 0.056968816 |
| GINM1 | Epi3 | turquoise | 0.091966095 | 0.029076308 | 0.055430194 | 0.129854982 | 0.05121947 | 0.024065274 | 0.078908662 | 0.042424368 | 0.096880899 |
| PCMT1 | Epi3 | turquoise | 0.132375034 | 0.157587546 | 0.085843697 | 0.250923808 | -2.79E-05 | 0.028123047 | 0.058522455 | 0.141577784 | 0.117161614 |
| ARMT1 | Epi3 | turquoise | 0.063010789 | 0.069962107 | 0.047204776 | 0.176458343 | 0.008191837 | 0.000588418 | 0.052387187 | 0.0539898 | 0.093382067 |
| TMEM242 | Epi3 | turquoise | 0.062837207 | 0.063400233 | 0.048650251 | 0.103624297 | 0.014084507 | -0.001825273 | 0.019894049 | 0.07457072 | 0.055696344 |
| GTF2H5 | Epi3 | turquoise | 0.174547416 | 0.093665686 | 0.173307744 | 0.183762965 | 0.084014717 | 0.071339938 | 0.062102523 | 0.148840436 | 0.133573406 |
| SFT2D1 | Epi3 | turquoise | 0.175008394 | 0.111858505 | 0.169666416 | 0.19194602 | 0.064061287 | 0.055815315 | 0.037295501 | 0.182932359 | 0.116444733 |
| PSMB1 | Epi3 | turquoise | 0.176298077 | 0.138706389 | 0.133419309 | 0.339569654 | 0.069007947 | 0.022056787 | 0.1326491 | 0.128325244 | 0.196725997 |
| ACTB | Epi3 | turquoise | 0.312648523 | 0.388942784 | 0.148158496 | 0.515085056 | 0.07589145 | 0.072223921 | 0.219166531 | 0.164616048 | 0.268698459 |
| KDELR2 | Epi3 | turquoise | 0.145666524 | 0.076550763 | 0.082560957 | 0.156785217 | 0.100433724 | 0.047541906 | 0.094069564 | 0.029686243 | 0.135492872 |
| RPA3 | Epi3 | turquoise | 0.100921775 | 0.047538966 | 0.06739819 | 0.126016328 | 0.058764692 | 0.012528376 | 0.094584633 | 0.037708788 | 0.105444143 |
| NDUFA4 | Epi3 | turquoise | 0.357842436 | 0.15461746 | 0.375994932 | 0.426407667 | 0.226634447 | 0.078426319 | 0.170800026 | 0.282057228 | 0.381810385 |
| TSPAN13 | Epi3 | turquoise | 0.082940168 | 0.099732895 | 0.046339153 | 0.119467565 | 0.048934789 | -0.011852711 | 0.030678655 | 0.061528323 | 0.057685652 |
| MALSU1 | Epi3 | turquoise | 0.087179373 | 0.083163391 | 0.042393525 | 0.124620537 | 0.039647503 | 0.009966076 | 0.081099469 | 0.029588383 | 0.06682428 |
| HNRNPA2B1 | Epi3 | turquoise | 0.152557925 | 0.104789163 | 0.10933628 | 0.264227558 | 0.044146163 | 0.071292358 | 0.121631093 | 0.102028483 | 0.170392134 |
| CBX3 | Epi3 | turquoise | 0.17622385 | 0.113899661 | 0.165229212 | 0.180735954 | 0.138197172 | 0.072294215 | 0.045259659 | 0.136385472 | 0.141244827 |
| HIBADH | Epi3 | turquoise | 0.094655949 | 0.023184562 | 0.12021475 | 0.138583029 | 0.067566062 | 0.029055865 | 0.062188297 | 0.061250641 | 0.139517273 |
| CPVL | Epi3 | turquoise | 0.069991438 | 0.009460317 | 0.085228424 | 0.36858407 | -0.005212518 | 0.014369605 | 0.081603014 | 0.142884829 | 0.266534382 |
| RP9 | Epi3 | turquoise | 0.055349386 | 0.035767842 | 0.034781041 | 0.086606152 | 0.053627618 | 0.018513256 | 0.078328614 | 0.004569429 | 0.077891329 |
| SEPT7 | Epi3 | turquoise | 0.129764004 | 0.102003577 | 0.093064024 | 0.208908224 | 0.043217227 | 0.049183166 | 0.04979794 | 0.120486905 | 0.129708574 |
| PSMA2 | Epi3 | turquoise | 0.150226511 | 0.112638661 | 0.106228963 | 0.234981132 | 0.096561712 | 0.046391342 | 0.102608872 | 0.092868027 | 0.15100742 |
| MRPL32 | Epi3 | turquoise | 0.067600106 | 0.046351603 | 0.033761662 | 0.130764004 | 0.026993123 | 0.026153274 | 0.055942683 | 0.049343409 | 0.09100227 |
| BLVRA | Epi3 | turquoise | 0.089990291 | 0.064715724 | 0.069710936 | 0.123521912 | 0.060341126 | 0.005021743 | 0.042009364 | 0.076347412 | 0.084804152 |
| DBNL | Epi3 | turquoise | 0.052197867 | 0.033814007 | 0.031597221 | 0.091344623 | 0.039593325 | 0.001316272 | 0.033697033 | 0.019265496 | 0.065478783 |
| DDX56 | Epi3 | turquoise | 0.036121045 | 0.03120907 | -0.002772331 | 0.089748594 | -0.00048397 | 0.014169709 | 0.081349008 | -0.009688147 | 0.040076111 |
| PPIA | Epi3 | turquoise | 0.286815823 | 0.218186331 | 0.242445908 | 0.521016245 | 0.153788252 | 0.048240322 | 0.183333157 | 0.249014433 | 0.348575015 |
| CCT6A | Epi3 | turquoise | 0.074401075 | 0.031417475 | 0.036398495 | 0.111222746 | 0.070385133 | 0.027019044 | 0.068486655 | 0.00151585 | 0.082187361 |
| CHCHD2 | Epi3 | turquoise | 0.249546178 | 0.119874406 | 0.234086381 | 0.30813268 | 0.169251174 | 0.04289015 | 0.1448794 | 0.191918993 | 0.234928827 |
| ASL | Epi3 | turquoise | 0.051710562 | 0.031979671 | 0.063068422 | 0.106287319 | 0.01568696 | 0.011223312 | 0.016679823 | 0.069328592 | 0.08242053 |
| SBDS | Epi3 | turquoise | 0.13953703 | 0.092849033 | 0.114869975 | 0.168552708 | 0.056101743 | 0.059239067 | 0.069836722 | 0.106738794 | 0.115160824 |
| WBSCR22 | Epi3 | turquoise | 0.101516853 | 0.067942053 | 0.073317015 | 0.184624865 | 0.028357878 | 0.029896155 | 0.063112843 | 0.112298601 | 0.099385789 |
| ABHD11 | Epi3 | turquoise | 0.054025312 | 0.0365305 | -0.015507002 | 0.105385309 | 0.024969821 | 0.024653119 | 0.118076367 | -0.022065779 | 0.069705058 |
| POR | Epi3 | turquoise | 0.062088314 | 0.071612531 | 0.043062469 | 0.210761509 | 0.005074401 | 0.018470804 | 0.092374981 | 0.024196152 | 0.12966831 |
| MDH2 | Epi3 | turquoise | 0.083268719 | 0.079212148 | 0.027995317 | 0.186513511 | 0.013580903 | 0.03456022 | 0.09257369 | 0.018558671 | 0.113380014 |
| HSPB1 | Epi3 | turquoise | 0.167537091 | 0.155234913 | 0.102094901 | 0.230997324 | 0.056635525 | 0.028384176 | 0.132575271 | 0.098462491 | 0.138887682 |
| SRI | Epi3 | turquoise | 0.206905335 | 0.15958419 | 0.217640991 | 0.283452213 | 0.114527084 | 0.068892477 | 0.05812105 | 0.180536209 | 0.222098444 |
| BET1 | Epi3 | turquoise | 0.092903404 | 0.05026631 | 0.071787471 | 0.088294029 | 0.065646266 | 0.004241835 | 0.029445305 | 0.072130889 | 0.074066183 |
| SGCE | Epi3 | turquoise | 0.075589261 | 0.031897409 | 0.083996209 | 0.150651647 | 0.028539731 | 0.057978101 | 0.025437195 | 0.086562524 | 0.106217099 |
| PON2 | Epi3 | turquoise | 0.116470352 | 0.039911332 | 0.1378035 | 0.147571166 | 0.065163988 | 0.030403579 | 0.046282417 | 0.11641738 | 0.137084662 |
| ATP5J2 | Epi3 | turquoise | 0.219923918 | 0.109801299 | 0.239441211 | 0.327886515 | 0.140278629 | 0.062636268 | 0.107726397 | 0.176892472 | 0.264016852 |
| COPS6 | Epi3 | turquoise | 0.070103357 | 0.062180772 | 0.066506357 | 0.167626729 | 0.034150216 | 0.024407241 | 0.058450694 | 0.067543452 | 0.109678768 |
| TRIP6 | Epi3 | turquoise | 0.107145656 | 0.065784909 | 0.093319005 | 0.187031323 | 0.058164241 | 0.033642654 | 0.084967443 | 0.056314888 | 0.147012106 |
| AP1S1 | Epi3 | turquoise | 0.142208064 | 0.091650764 | 0.135623023 | 0.152434176 | 0.089038002 | 0.045378046 | 0.038453855 | 0.106292425 | 0.109808982 |
| PMPCB | Epi3 | turquoise | 0.06977638 | 0.020712308 | 0.029483928 | 0.111947652 | 0.048643678 | 0.027313508 | 0.078473621 | 0.015408868 | 0.074837354 |
| PSMC2 | Epi3 | turquoise | 0.110999333 | 0.099156607 | 0.09350943 | 0.208543979 | 0.03323453 | 0.043148082 | 0.043332219 | 0.10952165 | 0.141849659 |
| HBP1 | Epi3 | turquoise | 0.075805168 | 0.066247782 | 0.020754377 | 0.123958844 | 0.025230911 | 0.013626186 | 0.068631242 | 0.021158077 | 0.057086473 |
| BCAP29 | Epi3 | turquoise | 0.095669491 | 0.022756198 | 0.046387557 | 0.186594447 | 0.052572372 | 0.025708215 | 0.124583668 | 0.018562572 | 0.126120418 |
| DLD | Epi3 | turquoise | 0.075828361 | 0.045069626 | 0.047076848 | 0.09942201 | 0.020837686 | 0.040622297 | 0.057942013 | 0.040087725 | 0.061104719 |
| DNAJB9 | Epi3 | turquoise | 0.144261802 | 0.066264148 | 0.095938756 | 0.200794483 | 0.060524031 | 0.061835098 | 0.092467986 | 0.090072115 | 0.13827295 |
| IMMP2L | Epi3 | turquoise | 0.039407896 | -0.004683262 | 0.030436209 | 0.086590908 | 0.033159689 | -0.016506049 | 0.052462597 | 0.001740013 | 0.093212897 |
| CAPZA2 | Epi3 | turquoise | 0.189510024 | 0.166213955 | 0.118750512 | 0.313220948 | 0.045084925 | 0.070093761 | 0.113358601 | 0.181962726 | 0.17770716 |
| NDUFA5 | Epi3 | turquoise | 0.149863854 | 0.012213112 | 0.121103959 | 0.222346093 | 0.096884619 | 0.015979222 | 0.15986524 | 0.070761786 | 0.18374975 |
| CALU | Epi3 | turquoise | 0.088324565 | 0.014595596 | 0.080349565 | 0.107648388 | 0.067782301 | 0.048194289 | 0.096996375 | 0.01973347 | 0.114023137 |
| AKR1B1 | Epi3 | turquoise | 0.119753315 | 0.161438788 | 0.054676883 | 0.170080247 | 0.061896816 | 0.03660878 | 0.092429983 | 0.043820356 | 0.098742801 |
| C7orf55 | Epi3 | turquoise | 0.113151378 | 0.005639964 | 0.109757393 | 0.179680452 | 0.101007635 | -0.012899522 | 0.120610972 | 0.051507464 | 0.153275488 |
| SSBP1 | Epi3 | turquoise | 0.186272386 | 0.066041589 | 0.199135047 | 0.224702103 | 0.147949273 | 0.052371535 | 0.098357958 | 0.093841932 | 0.233939466 |
| GSTK1 | Epi3 | turquoise | 0.115048929 | 0.066141313 | 0.060879838 | 0.174938173 | 0.09281337 | 0.033967859 | 0.151426545 | 0.010775122 | 0.123736195 |
| PDIA4 | Epi3 | turquoise | 0.112599129 | 0.124143042 | 0.01772159 | 0.224880353 | 0.000434798 | 0.046196209 | 0.147680597 | 0.044071944 | 0.109526276 |
| ASMTL | Epi3 | turquoise | 0.041334664 | 0.022258298 | 0.02470555 | 0.0795699 | 0.012977361 | 0.000703022 | 0.033892343 | 0.025745705 | 0.058543501 |
| GEMIN8 | Epi3 | turquoise | 0.057909718 | 0.030189637 | 0.042509416 | 0.0753342 | 0.027555339 | 0.02115995 | 0.024640127 | 0.048631245 | 0.039497669 |
| PIR | Epi3 | turquoise | 0.022790022 | 0.092210596 | 0.019322756 | 0.096797545 | -0.084127648 | -0.010074226 | -0.082098117 | 0.16644828 | -0.047446057 |
| RBBP7 | Epi3 | turquoise | 0.099008348 | 0.102093642 | 0.076552107 | 0.138710282 | 0.023003165 | 0.041070287 | 0.046503176 | 0.105507672 | 0.060796255 |
| PDHA1 | Epi3 | turquoise | 0.049082575 | 0.027781018 | 0.035369977 | 0.13372669 | 0.014493966 | -0.002827576 | 0.074962673 | 0.02908349 | 0.074939721 |
| PRDX4 | Epi3 | turquoise | 0.103304009 | 0.10886 | 0.064516534 | 0.202178352 | 0.046152594 | -0.005198792 | 0.056950859 | 0.064337046 | 0.119491947 |
| DYNLT3 | Epi3 | turquoise | 0.095247084 | 0.074037083 | 0.094745023 | 0.112891087 | 0.037140525 | 0.042182463 | 0.022192862 | 0.073949141 | 0.075803948 |
| ATP6AP2 | Epi3 | turquoise | 0.14180746 | 0.07947261 | 0.068225423 | 0.388578007 | 0.042233688 | 0.027633787 | 0.193360873 | 0.058949858 | 0.271243809 |
| NDUFB11 | Epi3 | turquoise | 0.152940968 | 0.064884368 | 0.089101586 | 0.246416549 | 0.114457043 | 0.041021099 | 0.192844011 | 0.015990421 | 0.189985736 |
| TIMP1 | Epi3 | turquoise | 0.221322287 | 0.435450148 | 0.194760912 | 0.518880901 | -0.073306366 | 0.066833265 | -0.070512756 | 0.452859649 | 0.167205831 |
| PRAF2 | Epi3 | turquoise | 0.08003991 | 0.03830178 | 0.075556675 | 0.099556975 | 0.063805113 | 0.032076634 | 0.050458996 | 0.050907702 | 0.09604317 |
| HSD17B10 | Epi3 | turquoise | 0.072426816 | 0.065468928 | 0.004383909 | 0.142130738 | 0.045176567 | -0.001494151 | 0.121763985 | 0.002593732 | 0.073729072 |
| MAGED2 | Epi3 | turquoise | 0.063209151 | 0.061342569 | 0.007183794 | 0.263526455 | -0.031315522 | -0.017467548 | 0.128384833 | 0.033577654 | 0.124484467 |
| MAGEH1 | Epi3 | turquoise | 0.040665697 | -0.007703539 | 0.004174424 | 0.071013362 | 0.001954672 | 0.000706273 | 0.086715275 | -0.007993483 | 0.042026772 |
| UBQLN2 | Epi3 | turquoise | 0.085359471 | 0.053530626 | 0.03784644 | 0.107301843 | 0.053940499 | 0.016010936 | 0.074261818 | 0.024763216 | 0.088600663 |
| YIPF6 | Epi3 | turquoise | 0.103792064 | 0.05807967 | 0.071152796 | 0.117024196 | 0.060220124 | 0.04078643 | 0.053725625 | 0.064548034 | 0.076603762 |
| PIN4 | Epi3 | turquoise | 0.11438213 | 0.037775835 | 0.111391849 | 0.104946548 | 0.075425581 | 0.058112825 | 0.046094237 | 0.074911701 | 0.092721207 |
| PBDC1 | Epi3 | turquoise | 0.063799263 | 0.063475484 | 0.052128178 | 0.115977953 | 0.029031663 | 0.02281585 | 0.041202715 | 0.073380148 | 0.073635708 |
| COX7B | Epi3 | turquoise | 0.277607373 | 0.113086794 | 0.298581417 | 0.247196918 | 0.169230019 | 0.083139853 | 0.104527918 | 0.192213709 | 0.240550194 |
| PGK1 | Epi3 | turquoise | 0.114005144 | 0.105092469 | 0.043635465 | 0.283369032 | 0.014388211 | 0.026985909 | 0.155583149 | 0.048145711 | 0.160416414 |
| SH3BGRL | Epi3 | turquoise | 0.174839743 | 0.025828859 | 0.183167249 | 0.194334035 | 0.122921881 | 0.036575418 | 0.098186366 | 0.095046271 | 0.198390459 |
| TSPAN6 | Epi3 | turquoise | 0.057617433 | 0.005661825 | 0.00743776 | 0.155632064 | 0.04492776 | 0.011554963 | 0.129922139 | -0.0323257 | 0.122855619 |
| HNRNPH2 | Epi3 | turquoise | 0.102358983 | 0.101121997 | 0.060435292 | 0.190050463 | 0.024835436 | 0.024523055 | 0.073032553 | 0.075763663 | 0.09738038 |
| ARMCX1 | Epi3 | turquoise | 0.060570949 | 0.00842322 | 0.027435076 | 0.082554259 | 0.035652443 | -0.002493339 | 0.089500092 | 0.018306131 | 0.062144525 |
| BEX4 | Epi3 | turquoise | 0.170500447 | 0.092576443 | 0.131756034 | 0.211240747 | 0.061295943 | 0.056239993 | 0.067181641 | 0.177947238 | 0.131652527 |
| TCEAL4 | Epi3 | turquoise | 0.16272325 | 0.078167665 | 0.119649173 | 0.181446198 | 0.054787729 | 0.042463228 | 0.080228236 | 0.13486217 | 0.121998209 |
| PLS3 | Epi3 | turquoise | 0.144895562 | 0.13522061 | 0.053798913 | 0.21396396 | 0.071903733 | 0.042136428 | 0.127378822 | 0.057381389 | 0.118217183 |
| PGRMC1 | Epi3 | turquoise | 0.16406028 | 0.22313401 | 0.10173309 | 0.222579154 | 0.068185781 | 0.033881127 | 0.046483424 | 0.105456985 | 0.120437265 |
| UPF3B | Epi3 | turquoise | 0.063815019 | 0.047630637 | 0.02824237 | 0.088421157 | 0.016537931 | -0.001645455 | 0.058656545 | 0.026856655 | 0.03326397 |
| NDUFA1 | Epi3 | turquoise | 0.183357144 | 0.103446476 | 0.162436521 | 0.235465484 | 0.133424849 | 0.068026822 | 0.142518079 | 0.088833463 | 0.192809312 |
| NKAP | Epi3 | turquoise | 0.064214087 | 0.027842233 | 0.067643004 | 0.072182836 | 0.050207527 | 0.015106502 | 0.026996931 | 0.038448376 | 0.059875726 |
| LAMP2 | Epi3 | turquoise | 0.155708308 | 0.149101442 | 0.20427517 | 0.359637624 | 0.003994664 | 0.020695176 | 0.020960116 | 0.200981017 | 0.258815329 |
| MCTS1 | Epi3 | turquoise | 0.073860824 | 0.02673847 | 0.074035431 | 0.124129852 | 0.056595768 | 0.033452935 | 0.034592945 | 0.046251 | 0.104524586 |
| C1GALT1C1 | Epi3 | turquoise | 0.048395426 | 0.105348854 | 0.025534821 | 0.298776792 | -0.060338617 | -0.00813415 | 0.048855495 | 0.111555145 | 0.139396799 |
| ARHGAP36 | Epi3 | turquoise | 0.037484642 | -0.003364362 | 0.064697985 | 0.164323358 | 0.032195612 | 0.028601292 | 0.019440574 | 0.082738521 | 0.129083724 |
| HPRT1 | Epi3 | turquoise | 0.094171011 | 0.030984994 | 0.093423247 | 0.14052364 | 0.037593838 | 0.024692482 | 0.062099673 | 0.080248223 | 0.117141179 |
| VMA21 | Epi3 | turquoise | 0.078067648 | 0.056006786 | 0.062173467 | 0.065974225 | 0.045272767 | 0.026693961 | 0.033171788 | 0.060860693 | 0.037104611 |
| CETN2 | Epi3 | turquoise | 0.126695917 | 0.0281123 | 0.119114491 | 0.240986754 | 0.074184916 | 0.027080459 | 0.114203896 | 0.083938152 | 0.192807187 |
| BCAP31 | Epi3 | turquoise | 0.143436506 | 0.097738723 | 0.123079709 | 0.255166971 | 0.039998979 | 0.033896973 | 0.07003558 | 0.140917755 | 0.149571021 |
| IDH3G | Epi3 | turquoise | 0.074540598 | 0.049212912 | 0.038086685 | 0.184430816 | 0.034129367 | 0.033587475 | 0.082234384 | 0.040164392 | 0.121342439 |
| SSR4 | Epi3 | turquoise | 0.298761679 | 0.363536489 | 0.205870397 | 0.382103575 | 0.112795891 | 0.098357587 | 0.116531971 | 0.24090029 | 0.191682322 |
| ATP6AP1 | Epi3 | turquoise | 0.074001906 | 0.066897704 | 0.026997133 | 0.213260717 | 0.010816514 | -0.010794283 | 0.094381693 | 0.046534744 | 0.114395615 |
| VBP1 | Epi3 | turquoise | 0.108020909 | 0.059782223 | 0.083807137 | 0.165825924 | 0.072692931 | 0.028995269 | 0.07856757 | 0.081796326 | 0.1154945 |
| FDFT1 | Epi3 | turquoise | 0.059916839 | 0.044622831 | 0.023332575 | 0.104085161 | 0.009374019 | 0.023100325 | 0.066531483 | 0.037163673 | 0.034550962 |
| PDGFRL | Epi3 | turquoise | 0.068056886 | 0.079428817 | 0.006378904 | 0.218056158 | 0.003887683 | 0.01277758 | 0.082708677 | 0.028980462 | 0.123392053 |
| SH2D4A | Epi3 | turquoise | 0.071676331 | 0.052002174 | 0.062381747 | 0.081452065 | 0.056160093 | 0.012173675 | 0.01392034 | 0.036891149 | 0.054873562 |
| ATP6V1B2 | Epi3 | turquoise | 0.101083988 | 0.089584782 | 0.070516875 | 0.127943857 | 0.007410531 | 0.032106355 | 0.012625766 | 0.119934052 | 0.053889269 |
| R3HCC1 | Epi3 | turquoise | 0.041172832 | 0.031926609 | 0.013172423 | 0.062143488 | 0.012890995 | 0.004234799 | 0.025170738 | 0.043356828 | 0.021785381 |
| BNIP3L | Epi3 | turquoise | 0.095803619 | 0.028178314 | 0.076070909 | 0.105024086 | 0.084088498 | 0.043398834 | 0.067878098 | 0.050196571 | 0.088926447 |
| CLU | Epi3 | turquoise | 0.221033504 | 0.324794983 | 0.152713845 | 0.596090447 | -0.012739845 | 0.052249872 | 0.103950027 | 0.288167535 | 0.324063335 |
| SARAF | Epi3 | turquoise | 0.229894175 | 0.148521208 | 0.192306572 | 0.29995307 | 0.100568498 | 0.070037914 | 0.082978429 | 0.185557458 | 0.194019598 |
| LEPROTL1 | Epi3 | turquoise | 0.0957754 | 0.110464401 | 0.089541001 | 0.129515397 | 0.031909471 | 0.03265643 | 0.046879649 | 0.086376888 | 0.072079689 |
| DCTN6 | Epi3 | turquoise | 0.144871621 | 0.118944485 | 0.100278893 | 0.212573761 | 0.057530204 | 0.0266023 | 0.055566207 | 0.114263401 | 0.120020844 |
| UBXN8 | Epi3 | turquoise | 0.055750415 | 0.029644664 | 0.043929372 | 0.106802436 | 0.011543458 | 0.019806723 | 0.023144401 | 0.067480767 | 0.052869658 |
| FNTA | Epi3 | turquoise | 0.078435367 | 0.051137158 | 0.038825855 | 0.134421565 | 0.020337037 | 0.019195555 | 0.038146367 | 0.056158928 | 0.081658653 |
| ATP6V1H | Epi3 | turquoise | 0.049436746 | 0.0204429 | 0.019708921 | 0.106307648 | 0.002155824 | 0.014077011 | 0.074817476 | 0.007765119 | 0.074288061 |
| MRPL15 | Epi3 | turquoise | 0.050212207 | 0.018893171 | 0.03680951 | 0.094544705 | 0.04795068 | 0.01217328 | 0.048524434 | 0.014275215 | 0.065594046 |
| SDCBP | Epi3 | turquoise | 0.094765748 | 0.082596519 | -0.016490697 | 0.241434529 | -0.022175651 | 0.017746472 | 0.189172728 | 0.005329887 | 0.109955834 |
| RAB2A | Epi3 | turquoise | 0.194575443 | 0.118195033 | 0.145842712 | 0.218098944 | 0.093852386 | 0.076215398 | 0.092383009 | 0.128612974 | 0.14442958 |
| MRPS28 | Epi3 | turquoise | 0.120435267 | 0.086486936 | 0.138352751 | 0.119440088 | 0.03081076 | 0.06877262 | 0.003476145 | 0.145917378 | 0.076171295 |
| IMPA1 | Epi3 | turquoise | 0.05747563 | 0.037629574 | 0.067148032 | 0.109534596 | 0.007945528 | 0.022781865 | 0.025108698 | 0.078881329 | 0.073136296 |
| DECR1 | Epi3 | turquoise | 0.118011695 | 0.052729618 | 0.097401166 | 0.185289919 | 0.056440438 | 0.040463341 | 0.081604839 | 0.082291745 | 0.129715494 |
| COX6C | Epi3 | turquoise | 0.278834355 | 0.11858229 | 0.289006526 | 0.341720071 | 0.189418663 | 0.075808972 | 0.150705457 | 0.185685467 | 0.301500008 |
| DCAF13 | Epi3 | turquoise | 0.050387741 | 0.058099412 | 0.01994131 | 0.132241648 | -0.01349621 | 0.033799743 | 0.059722407 | 0.035528059 | 0.062133854 |
| EMC2 | Epi3 | turquoise | 0.061580526 | 0.044379285 | 0.015853636 | 0.169844316 | 0.014463398 | 0.016433662 | 0.074284773 | 0.035596915 | 0.07925476 |
| TNFRSF11B | Epi3 | turquoise | 0.056536214 | 0.092916766 | 0.006670795 | 0.147787486 | -0.083076853 | 0.009747226 | 0.051683244 | 0.127527549 | -0.012521827 |
| MRPL13 | Epi3 | turquoise | 0.097550842 | 0.062364461 | 0.070117697 | 0.19844248 | 0.047883283 | -0.00072307 | 0.095745361 | 0.049909281 | 0.143797229 |
| NDUFB9 | Epi3 | turquoise | 0.169059614 | 0.143595071 | 0.09257463 | 0.263476199 | 0.076444156 | 0.037308958 | 0.122073284 | 0.091908677 | 0.163177951 |
| GSDMD | Epi3 | turquoise | 0.079506233 | 0.070253211 | 0.057061504 | 0.120230329 | 0.036413929 | 0.020572332 | 0.042436556 | 0.046129174 | 0.065215495 |
| TSTA3 | Epi3 | turquoise | 0.122374383 | 0.087593446 | 0.123730849 | 0.153529337 | 0.078439517 | 0.06011675 | 0.028341635 | 0.102045495 | 0.114799036 |
| PUF60 | Epi3 | turquoise | 0.082017997 | 0.063605072 | 0.04719873 | 0.189488435 | 0.030052992 | 0.047807917 | 0.086685425 | 0.048155826 | 0.120291574 |
| GPAA1 | Epi3 | turquoise | 0.123688213 | 0.086848227 | 0.120731903 | 0.140531592 | 0.077792303 | 0.050264298 | 0.041523352 | 0.111968939 | 0.128426755 |
| CYC1 | Epi3 | turquoise | 0.101664913 | 0.062798553 | 0.065934895 | 0.15970628 | 0.037247945 | 0.028543071 | 0.06283636 | 0.077125166 | 0.09982505 |
| VPS28 | Epi3 | turquoise | 0.138127903 | 0.057248437 | 0.116516546 | 0.231944329 | 0.094002928 | 0.051744472 | 0.126646778 | 0.083272792 | 0.156249156 |
| CBWD1 | Epi3 | turquoise | 0.114443455 | 0.048995121 | 0.086012069 | 0.113698 | 0.086135354 | 0.046860163 | 0.055838612 | 0.063220828 | 0.093824717 |
| PLGRKT | Epi3 | turquoise | 0.103227748 | 0.136074233 | 0.093635138 | 0.136062399 | 0.004107141 | 0.020426626 | 0.003056285 | 0.129205977 | 0.064388127 |
| TMEM261 | Epi3 | turquoise | 0.117914393 | 0.041876523 | 0.114352857 | 0.16176184 | 0.094166781 | 0.042709255 | 0.096134772 | 0.069527374 | 0.132610661 |
| RRAGA | Epi3 | turquoise | 0.099795453 | 0.059116684 | 0.062627744 | 0.201200214 | 0.025332848 | -0.006934489 | 0.100418806 | 0.05409739 | 0.148267859 |
| PLIN2 | Epi3 | turquoise | 0.085126768 | 0.107810507 | 0.072852865 | 0.132046563 | 0.012460806 | 0.015528108 | -0.011251395 | 0.119636156 | 0.059537114 |
| NDUFB6 | Epi3 | turquoise | 0.115704943 | 0.082243209 | 0.098764257 | 0.174089197 | 0.056115037 | -0.005315422 | 0.079000189 | 0.102206475 | 0.119709974 |
| DNAJA1 | Epi3 | turquoise | 0.125050672 | 0.114981599 | 0.066235079 | 0.262948627 | 0.030686701 | 0.028739626 | 0.12135948 | 0.091881698 | 0.13415299 |
| SMU1 | Epi3 | turquoise | 0.037728397 | 0.016064862 | 0.006152661 | 0.094765819 | 0.006981103 | 0.040304479 | 0.048295622 | 0.012326977 | 0.053078637 |
| CHMP5 | Epi3 | turquoise | 0.160761052 | 0.148773559 | 0.121629797 | 0.298293496 | 0.040624503 | 0.021588071 | 0.057293142 | 0.178535597 | 0.154186634 |
| RPP25L | Epi3 | turquoise | 0.056567162 | 0.067435826 | 0.012687164 | 0.11260839 | 0.001453347 | 0.022683004 | 0.081626078 | 0.037458579 | 0.032890481 |
| DCTN3 | Epi3 | turquoise | 0.107532699 | 0.058450035 | 0.086810888 | 0.16879738 | 0.065213394 | 0.014921363 | 0.09234938 | 0.071219093 | 0.13095059 |
| GALT | Epi3 | turquoise | 0.030334423 | 0.068790029 | -0.005928189 | 0.092392043 | -0.004608197 | -0.015003036 | 0.055614796 | 0.026700599 | 0.022583184 |
| VCP | Epi3 | turquoise | 0.075861682 | 0.063972154 | 0.022917141 | 0.161584031 | 0.01891882 | 0.01386733 | 0.111834527 | 0.020548599 | 0.082264586 |
| STOML2 | Epi3 | turquoise | 0.118896867 | 0.094872645 | 0.085948952 | 0.14676326 | 0.078682558 | 0.043995583 | 0.050329127 | 0.070678214 | 0.085802384 |
| HINT2 | Epi3 | turquoise | 0.104168779 | 0.063338185 | 0.083193997 | 0.116795017 | 0.04758444 | 0.033536814 | 0.037366146 | 0.090160413 | 0.070999695 |
| GRHPR | Epi3 | turquoise | 0.07384444 | 0.037212128 | 0.06225891 | 0.13611591 | 0.067138624 | 0.017449231 | 0.060391448 | 0.041183886 | 0.100862859 |
| ALDH1A1 | Epi3 | turquoise | 0.052695044 | 0.054659745 | -0.059024923 | 0.22154502 | -0.04188041 | 5.92E-05 | 0.197965364 | -0.035458306 | 0.085446416 |
| ANXA1 | Epi3 | turquoise | 0.214976986 | 0.10139827 | 0.238938753 | 0.421603555 | 0.117982602 | 0.033658345 | 0.124605355 | 0.182847315 | 0.382515741 |
| HNRNPK | Epi3 | turquoise | 0.155902243 | 0.117508109 | 0.07729653 | 0.195603003 | 0.064764161 | 0.057360895 | 0.113078254 | 0.071718034 | 0.124087309 |
| CTSL | Epi3 | turquoise | 0.080146836 | 0.071779479 | 0.006169011 | 0.165859831 | -0.002134129 | 0.024031312 | 0.108545432 | 0.04541768 | 0.065864044 |
| FBP1 | Epi3 | turquoise | 0.070216038 | 0.082756268 | 0.017432307 | 0.147947764 | 0.032999899 | 0.010313077 | 0.087656095 | -0.007749485 | 0.088076812 |
| XPA | Epi3 | turquoise | 0.059338202 | -0.003009934 | 0.06394721 | 0.092813366 | 0.0200677 | 0.000726028 | 0.052908655 | 0.045401214 | 0.07240707 |
| NANS | Epi3 | turquoise | 0.055705886 | 0.03195131 | 0.03165201 | 0.089386073 | 0.004875209 | 0.019053108 | 0.03624329 | 0.050359196 | 0.036149443 |
| ALG2 | Epi3 | turquoise | 0.067595082 | 0.059865867 | 0.045293803 | 0.132830853 | 0.043919654 | 0.034686482 | 0.05038665 | 0.046439191 | 0.09074289 |
| ERP44 | Epi3 | turquoise | 0.085473608 | 0.109766042 | 0.029489959 | 0.222225513 | -0.019676064 | -0.013518233 | 0.098347181 | 0.063124236 | 0.097434007 |
| MRPL50 | Epi3 | turquoise | 0.111267395 | 0.072279311 | 0.074269575 | 0.132595936 | 0.030754921 | 0.044575874 | 0.065726362 | 0.069029004 | 0.075268376 |
| NIPSNAP3A | Epi3 | turquoise | 0.04276819 | 0.017327298 | 0.028485522 | 0.108855148 | 0.021126539 | 0.009297592 | 0.085568936 | 0.012940619 | 0.079187998 |
| TMEM38B | Epi3 | turquoise | 0.068003905 | 0.01782375 | 0.063195179 | 0.113730237 | -0.009822099 | 0.01368961 | 0.035508578 | 0.091159868 | 0.059781699 |
| PTGR1 | Epi3 | turquoise | 0.056930153 | 0.030907347 | 0.0219898 | 0.103629462 | 0.020237914 | 0.020773575 | 0.052030083 | 0.023296189 | 0.05306245 |
| POLE3 | Epi3 | turquoise | 0.058501413 | 0.027424183 | 0.039261598 | 0.075466503 | 0.044512754 | 0.016366458 | 0.057670953 | 0.028524044 | 0.064767458 |
| ATP6V1G1 | Epi3 | turquoise | 0.216186597 | 0.126381624 | 0.156128262 | 0.231336811 | 0.115840438 | 0.062225286 | 0.099029404 | 0.148290594 | 0.166767359 |
| PSMD5 | Epi3 | turquoise | 0.066816018 | 0.055067738 | 0.050577342 | 0.065987341 | 0.029565354 | 0.009698204 | 0.020809349 | 0.046940707 | 0.029552947 |
| RAB14 | Epi3 | turquoise | 0.127536834 | 0.090885162 | 0.129319299 | 0.147515919 | 0.063332097 | 0.018951304 | 0.045745201 | 0.104281066 | 0.112666378 |
| GSN | Epi3 | turquoise | 0.151786076 | 0.220008328 | 0.181748261 | 0.287129674 | -0.018000932 | 0.02516756 | -0.074114191 | 0.288730712 | 0.119418086 |
| NDUFA8 | Epi3 | turquoise | 0.109377133 | 0.07318724 | 0.071057606 | 0.169788739 | 0.04317912 | 0.03479976 | 0.082554657 | 0.071305828 | 0.106201655 |
| PSMB7 | Epi3 | turquoise | 0.090633415 | 0.048915481 | 0.064133835 | 0.187957259 | 0.060961038 | 0.007326716 | 0.094639449 | 0.060061459 | 0.118198299 |
| HSPA5 | Epi3 | turquoise | 0.132147221 | 0.096525885 | 0.007373461 | 0.261045728 | -0.02713368 | 0.021001342 | 0.243589884 | -0.002249626 | 0.116976761 |
| SWI5 | Epi3 | turquoise | 0.131886445 | 0.143044518 | 0.108760356 | 0.166362271 | 0.008171125 | 0.06408563 | 0.016955482 | 0.182458421 | 0.047315257 |
| PHYHD1 | Epi3 | turquoise | 0.029941228 | 0.020707583 | -0.036122059 | 0.090926925 | 0.005908349 | 0.01466424 | 0.091309176 | -0.024890264 | 0.019470567 |
| TOR1A | Epi3 | turquoise | 0.061147109 | 0.076626543 | 0.024841489 | 0.08392199 | 0.018609851 | 0.000107665 | 0.034944135 | 0.040121939 | 0.027814761 |
| TMEM141 | Epi3 | turquoise | 0.131976438 | 0.053045985 | 0.096537011 | 0.157753243 | 0.093630004 | 0.034809178 | 0.088123168 | 0.063872272 | 0.115875401 |
| PHPT1 | Epi3 | turquoise | 0.244730573 | 0.176478717 | 0.251594021 | 0.276018426 | 0.135185121 | 0.075100223 | 0.033045216 | 0.236259525 | 0.200237648 |
| EDF1 | Epi3 | turquoise | 0.22767347 | 0.11096398 | 0.192781226 | 0.285021206 | 0.205871687 | 0.070936356 | 0.163972599 | 0.119197014 | 0.236194782 |
| TUBB4B | Epi3 | turquoise | 0.141645181 | 0.190435201 | 0.107212814 | 0.33471889 | -0.00514234 | 0.01568323 | 0.050101396 | 0.16166599 | 0.165785941 |
| ARRDC1 | Epi3 | turquoise | 0.06416676 | 0.086989864 | 0.04908884 | 0.103243298 | 0.043380068 | 0.00786502 | 0.06671243 | 0.030252508 | 0.073567438 |
| RIC8A | Epi3 | turquoise | 0.029744792 | 0.018636175 | 0.024236448 | 0.077647362 | 0.00737559 | 0.000385665 | 0.038219467 | 0.008800206 | 0.033805879 |
| PSMD13 | Epi3 | turquoise | 0.07248475 | 0.048653781 | 0.048500099 | 0.143502284 | 0.017830444 | 0.001758469 | 0.053310926 | 0.044395496 | 0.096512378 |
| SIGIRR | Epi3 | turquoise | 0.034263718 | 0.039244123 | -0.009732501 | 0.093415092 | 0.029238365 | 0.005281132 | 0.05748273 | -0.020426389 | 0.065377212 |
| RNH1 | Epi3 | turquoise | 0.126093793 | 0.08522235 | 0.08406136 | 0.241429464 | 0.047149494 | 0.016449057 | 0.101478759 | 0.100191509 | 0.147463074 |
| TMEM80 | Epi3 | turquoise | 0.03753642 | 0.020608885 | -0.001448898 | 0.093620469 | -0.005872808 | 0.009548417 | 0.058198683 | 0.004629805 | 0.05598202 |
| TALDO1 | Epi3 | turquoise | 0.12217313 | 0.036923106 | 0.079015602 | 0.302383147 | 0.082284943 | 0.007262222 | 0.162169695 | 0.045526518 | 0.229848261 |
| PNPLA2 | Epi3 | turquoise | 0.078751876 | 0.051278765 | 0.061507203 | 0.123902077 | 0.056090528 | 0.031888441 | 0.048060718 | 0.057836054 | 0.090085686 |
| CD151 | Epi3 | turquoise | 0.185122483 | 0.200042869 | 0.167430408 | 0.345874859 | 0.064266589 | 0.060989247 | 0.074286419 | 0.177792678 | 0.237258699 |
| TSPAN4 | Epi3 | turquoise | 0.100112586 | 0.042303908 | 0.091102019 | 0.178878334 | 0.069833599 | 0.022255331 | 0.072514326 | 0.058935272 | 0.159653836 |
| CTSD | Epi3 | turquoise | 0.132395114 | 0.186641143 | 0.038415661 | 0.276322864 | -0.017698531 | 0.029966286 | 0.080601959 | 0.098076346 | 0.110203302 |
| SLC22A18 | Epi3 | turquoise | 0.083500537 | 0.115947654 | 0.059238173 | 0.163627024 | 0.005510193 | 0.027624685 | 0.017350939 | 0.109371596 | 0.071190215 |
| ILK | Epi3 | turquoise | 0.088120009 | 0.046574376 | 0.087331324 | 0.163433428 | 0.060586335 | 0.051630701 | 0.088034851 | 0.052477977 | 0.138107402 |
| MRPL17 | Epi3 | turquoise | 0.095932238 | 0.075269254 | 0.076260352 | 0.124712088 | 0.056708392 | 0.032546099 | 0.068406672 | 0.066732232 | 0.096340855 |
| TMEM9B | Epi3 | turquoise | 0.15476993 | 0.130103604 | 0.129321934 | 0.180288242 | 0.07277739 | 0.027558872 | 0.065105713 | 0.114285418 | 0.119713939 |
| COPB1 | Epi3 | turquoise | 0.100191059 | 0.048024524 | 0.053733935 | 0.139433288 | 0.015097746 | 0.02578151 | 0.05344064 | 0.050129886 | 0.078139383 |
| PSMA1 | Epi3 | turquoise | 0.15566679 | 0.104838848 | 0.101887853 | 0.272278287 | 0.080272495 | 0.067635072 | 0.137062396 | 0.107986871 | 0.162887013 |
| NUCB2 | Epi3 | turquoise | 0.077774419 | 0.060741154 | 0.014472771 | 0.125937837 | -0.005432674 | -0.000642788 | 0.08922804 | 0.021166956 | 0.063186073 |
| TSG101 | Epi3 | turquoise | 0.080385577 | 0.097622794 | 0.03304158 | 0.180315247 | -0.001525024 | 0.019704718 | 0.058847657 | 0.070896305 | 0.082020593 |
| MUC15 | Epi3 | turquoise | 0.11757031 | 0.141719627 | 0.04189945 | 0.235129222 | -0.018465956 | 0.020408923 | 0.038747169 | 0.159985453 | 0.064892181 |
| EIF3M | Epi3 | turquoise | 0.122652517 | 0.001989576 | 0.08116654 | 0.135215605 | 0.134803549 | 0.041341485 | 0.087467127 | 0.017457632 | 0.136517203 |
| CSTF3 | Epi3 | turquoise | 0.072968708 | 0.024299347 | 0.075171126 | 0.107649132 | 0.00284994 | 0.023506218 | 0.046974402 | 0.082559694 | 0.066564138 |
| CD59 | Epi3 | turquoise | 0.257993338 | 0.323244567 | 0.184370039 | 0.381602263 | 0.066991156 | 0.069627789 | 0.123812809 | 0.228887168 | 0.222201 |
| FBXO3 | Epi3 | turquoise | 0.071799209 | 0.051566676 | 0.037555249 | 0.107231249 | 0.012050688 | 0.013538442 | 0.058558967 | 0.040135903 | 0.059470041 |
| CAT | Epi3 | turquoise | 0.079820035 | 0.017579693 | 0.046562447 | 0.248589529 | 0.018417691 | 0.007475668 | 0.110649993 | 0.069017536 | 0.163262341 |
| APIP | Epi3 | turquoise | 0.071499558 | 0.04099616 | 0.068497041 | 0.104633103 | 0.022090925 | 0.006761668 | 0.024458866 | 0.083905285 | 0.052705163 |
| COMMD9 | Epi3 | turquoise | 0.061762978 | 0.037205568 | 0.004723765 | 0.100939755 | 0.023509961 | 0.045829804 | 0.075178879 | 0.019151361 | 0.048611549 |
| HSD17B12 | Epi3 | turquoise | 0.101975376 | 0.057647215 | 0.06510565 | 0.124456298 | 0.042267395 | 0.021521945 | 0.040970732 | 0.051865584 | 0.087213676 |
| C11orf49 | Epi3 | turquoise | 0.087299167 | 0.046285498 | 0.091749304 | 0.132535466 | 0.054017144 | 0.005428498 | 0.063825819 | 0.081412872 | 0.09703138 |
| PSMC3 | Epi3 | turquoise | 0.107625357 | 0.091357868 | 0.085292601 | 0.178961034 | 0.047074111 | 0.005436842 | 0.062218435 | 0.080625165 | 0.114177908 |
| NDUFS3 | Epi3 | turquoise | 0.093613707 | 0.084688322 | 0.061684181 | 0.209951616 | 0.033645337 | 0.038240404 | 0.065963329 | 0.070739138 | 0.129969542 |
| SSRP1 | Epi3 | turquoise | 0.038913999 | 0.046688574 | 0.031206166 | 0.054382403 | 0.03516933 | 0.032896817 | 0.02033099 | 0.015145602 | 0.033819346 |
| SERPING1 | Epi3 | turquoise | 0.065220021 | 0.082098662 | 0.022358207 | 0.108728442 | 0.042903017 | 0.023794641 | 0.080756515 | -0.002040862 | 0.066208999 |
| MED19 | Epi3 | turquoise | 0.080643736 | 0.057438355 | 0.045200381 | 0.116483022 | 0.012237911 | 0.042749709 | 0.013890454 | 0.039284494 | 0.067183422 |
| TMX2 | Epi3 | turquoise | 0.075800533 | 0.04212572 | 0.053258372 | 0.129140407 | 0.019321061 | 0.025925933 | 0.048683575 | 0.062542399 | 0.102141974 |
| PRPF19 | Epi3 | turquoise | 0.045799318 | 0.027688858 | 0.024792056 | 0.08555906 | 0.018882045 | -0.005076297 | 0.01967132 | 0.062433006 | 0.053519506 |
| TMEM109 | Epi3 | turquoise | 0.080286783 | 0.055313923 | 0.056223836 | 0.110833152 | 0.020572854 | 0.037911345 | 0.031189021 | 0.08040583 | 0.058378004 |
| SDHAF2 | Epi3 | turquoise | 0.068709875 | 0.046343639 | 0.030881105 | 0.09697683 | 0.021647185 | 0.020413967 | 0.06359529 | 0.03884968 | 0.045327558 |
| TMEM258 | Epi3 | turquoise | 0.243656602 | 0.088916142 | 0.249228759 | 0.258921447 | 0.24670923 | 0.05757343 | 0.139800824 | 0.134802325 | 0.262011145 |
| B3GAT3 | Epi3 | turquoise | 0.082162705 | 0.132984709 | 0.048141805 | 0.120720284 | -0.000419367 | 0.013752215 | 7.28E-05 | 0.09321273 | 0.023303995 |
| POLR2G | Epi3 | turquoise | 0.107576738 | 0.03605247 | 0.077550659 | 0.232726917 | 0.030162116 | -0.006111264 | 0.098996617 | 0.08304369 | 0.148853746 |
| TMEM179B | Epi3 | turquoise | 0.110971622 | 0.091595617 | 0.099696846 | 0.205722712 | 0.054195667 | 0.035141091 | 0.029753213 | 0.102471053 | 0.155441321 |
| SLC3A2 | Epi3 | turquoise | 0.111912466 | 0.093457719 | 0.089390128 | 0.242762492 | -0.006626862 | 0.027845861 | 0.04035272 | 0.150807078 | 0.124072244 |
| RTN3 | Epi3 | turquoise | 0.075303116 | 0.027501682 | 0.000271763 | 0.183775059 | 0.018708586 | 0.006427128 | 0.155921101 | -0.017717483 | 0.109445722 |
| OTUB1 | Epi3 | turquoise | 0.053122974 | 0.056769941 | 0.035474762 | 0.149274542 | 0.018865147 | 0.029010025 | 0.046695498 | 0.052798349 | 0.060832196 |
| MACROD1 | Epi3 | turquoise | 0.03768106 | 0.049050736 | -0.020705571 | 0.076996321 | 0.014299731 | 0.003466661 | 0.095226546 | -0.005896091 | -0.000933244 |
| STIP1 | Epi3 | turquoise | 0.052527869 | 0.059907672 | 0.025430767 | 0.11232767 | -0.008217994 | 0.032542202 | 0.036842264 | 0.048495565 | 0.04093157 |
| FKBP2 | Epi3 | turquoise | 0.20990065 | 0.137765259 | 0.180135385 | 0.296899161 | 0.089807892 | 0.071322059 | 0.137554654 | 0.151082325 | 0.199861674 |
| BAD | Epi3 | turquoise | 0.14794269 | 0.067011937 | 0.126774542 | 0.131492148 | 0.10260335 | 0.047070414 | 0.092633837 | 0.084094697 | 0.109880782 |
| TRMT112 | Epi3 | turquoise | 0.205664727 | 0.110532505 | 0.219179907 | 0.273339699 | 0.176947109 | 0.069066104 | 0.076812708 | 0.16749091 | 0.228696979 |
| ARL2 | Epi3 | turquoise | 0.101731306 | 0.077387883 | 0.077165575 | 0.116422835 | 0.027576023 | 0.035935123 | 0.04572398 | 0.092424165 | 0.059690786 |
| SSSCA1 | Epi3 | turquoise | 0.081043623 | 0.020106359 | 0.055125373 | 0.159025532 | 0.040097784 | 0.032034838 | 0.086626068 | 0.039548919 | 0.110252015 |
| KAT5 | Epi3 | turquoise | 0.061260044 | 0.04771501 | 0.040743997 | 0.091137443 | 0.02152554 | 0.017347195 | 0.006881063 | 0.058965036 | 0.047212699 |
| CFL1 | Epi3 | turquoise | 0.351313481 | 0.256829255 | 0.325933262 | 0.428573439 | 0.189988837 | 0.112462873 | 0.123027395 | 0.268400406 | 0.314156695 |
| EFEMP2 | Epi3 | turquoise | 0.095938889 | 0.041704187 | 0.068600308 | 0.141794095 | 0.062170378 | 0.027506355 | 0.080573871 | 0.047235735 | 0.117881651 |
| BANF1 | Epi3 | turquoise | 0.194448076 | 0.098019985 | 0.205340265 | 0.220960888 | 0.146246069 | 0.051439525 | 0.093747422 | 0.145465752 | 0.201533156 |
| SF3B2 | Epi3 | turquoise | 0.089772125 | 0.062991622 | 0.045069371 | 0.112642638 | 0.030896268 | 0.022210813 | 0.041247029 | 0.046417518 | 0.057340582 |
| YIF1A | Epi3 | turquoise | 0.155889655 | 0.085223361 | 0.135515624 | 0.150892106 | 0.106610882 | 0.085135552 | 0.073803554 | 0.068313379 | 0.126909867 |
| B4GAT1 | Epi3 | turquoise | 0.03282785 | 0.049661384 | 0.006602276 | 0.110095219 | -0.012405086 | -1.67E-06 | 0.020293463 | 0.066905543 | 0.03229131 |
| CCS | Epi3 | turquoise | 0.05753967 | 0.0293381 | 0.021700651 | 0.124910166 | 0.000189673 | 0.024330456 | 0.086210306 | 0.023975155 | 0.045205833 |
| PPP1CA | Epi3 | turquoise | 0.14872198 | 0.08618115 | 0.130794555 | 0.257930101 | 0.076911136 | 0.059938809 | 0.10062632 | 0.118758097 | 0.188511403 |
| CORO1B | Epi3 | turquoise | 0.111494134 | 0.079061379 | 0.081523557 | 0.177572073 | 0.06387079 | 0.040979932 | 0.07937591 | 0.066171949 | 0.12093862 |
| AIP | Epi3 | turquoise | 0.103098246 | 0.038951923 | 0.079534968 | 0.160157872 | 0.081721769 | 0.014828374 | 0.083355928 | 0.065496666 | 0.119964849 |
| GSTP1 | Epi3 | turquoise | 0.213123828 | 0.206792965 | 0.042502397 | 0.404130946 | 0.150049745 | 0.054957222 | 0.326899275 | -0.02881826 | 0.236414865 |
| NDUFV1 | Epi3 | turquoise | 0.086844922 | 0.061384332 | 0.018914371 | 0.123721168 | 0.036680633 | 0.000366012 | 0.081829593 | 0.021156076 | 0.060734595 |
| NDUFS8 | Epi3 | turquoise | 0.187206983 | 0.105603801 | 0.19062049 | 0.238442251 | 0.125216618 | 0.033010123 | 0.078324273 | 0.145988505 | 0.185005973 |
| MRPL21 | Epi3 | turquoise | 0.100802951 | 0.055052374 | 0.073425772 | 0.133698251 | 0.064202907 | 0.047844005 | 0.087813362 | 0.046480283 | 0.10265634 |
| DHCR7 | Epi3 | turquoise | 0.074964666 | 0.077551425 | 0.083218713 | 0.093374713 | 0.001277534 | 0.015347279 | -0.035488241 | 0.105620604 | 0.05046613 |
| LAMTOR1 | Epi3 | turquoise | 0.196417876 | 0.116151819 | 0.163557079 | 0.170252183 | 0.138233129 | 0.059608931 | 0.0767861 | 0.122877958 | 0.126600746 |
| FOLR1 | Epi3 | turquoise | 0.061301916 | 0.149511567 | 0.001663626 | 0.21600505 | -0.075092155 | -0.01065592 | 0.054044695 | 0.107494371 | 0.039930846 |
| MRPL48 | Epi3 | turquoise | 0.100299206 | 0.08538249 | 0.066851544 | 0.132435237 | 0.044730968 | 0.018721849 | 0.054079172 | 0.060963435 | 0.085545798 |
| SPCS2 | Epi3 | turquoise | 0.150922577 | 0.159752487 | 0.088338612 | 0.336878164 | 0.038140434 | 0.04611384 | 0.137000788 | 0.121176054 | 0.172140998 |
| SERPINH1 | Epi3 | turquoise | 0.074159535 | 0.076072942 | 0.036843212 | 0.109589401 | -0.002207321 | 0.035339605 | 0.054369103 | 0.058558645 | 0.048344663 |
| AAMDC | Epi3 | turquoise | 0.095881766 | 0.028325473 | 0.092441809 | 0.127057693 | 0.077019671 | 0.021546473 | 0.079874654 | 0.065606073 | 0.117028361 |
| NDUFC2 | Epi3 | turquoise | 0.30680689 | 0.132636285 | 0.331140309 | 0.387797144 | 0.175390412 | 0.055447403 | 0.128460483 | 0.252385734 | 0.328729391 |
| ALG8 | Epi3 | turquoise | 0.043654777 | 0.059693331 | 0.023320602 | 0.139250366 | 0.000493824 | 0.001762685 | 0.064093042 | 0.055940505 | 0.04563458 |
| CREBZF | Epi3 | turquoise | 0.064510397 | 0.024444786 | 0.068153092 | 0.077994459 | 0.01983728 | 0.049331723 | 0.010252166 | 0.080966173 | 0.038021907 |
| C11orf54 | Epi3 | turquoise | 0.044172505 | 0.026259089 | 0.031956984 | 0.10652228 | -0.008715966 | 0.01485624 | 0.043707115 | 0.07161638 | 0.049598112 |
| AASDHPPT | Epi3 | turquoise | 0.050274059 | 0.030106202 | 0.037142724 | 0.087301567 | 0.02815663 | 0.0232194 | 0.031048903 | 0.049407172 | 0.065119276 |
| ACAT1 | Epi3 | turquoise | 0.04791667 | 0.098025449 | -0.00511849 | 0.129690137 | -0.045822611 | 0.018376226 | 0.061816498 | 0.024191839 | 0.021197743 |
| RDX | Epi3 | turquoise | 0.103903086 | 0.07001734 | 0.057598059 | 0.130531321 | 0.032478229 | 0.053903638 | 0.084554682 | 0.06563663 | 0.090968286 |
| SDHD | Epi3 | turquoise | 0.096741322 | 0.049186077 | 0.039130539 | 0.184719547 | 0.029036922 | 0.05077091 | 0.097529148 | 0.052391141 | 0.099691975 |
| CADM1 | Epi3 | turquoise | 0.161622735 | 0.109168455 | 0.186501 | 0.179815371 | 0.097102536 | 0.05153237 | 0.024990511 | 0.130482574 | 0.168845445 |
| TRAPPC4 | Epi3 | turquoise | 0.082755387 | 0.076176936 | 0.0653366 | 0.160418537 | 0.044504472 | 0.034846777 | 0.083854595 | 0.061171809 | 0.103443783 |
| SC5D | Epi3 | turquoise | 0.040492163 | 0.069454814 | 0.018742624 | 0.073203454 | -0.008578482 | 0.019772035 | 0.036864427 | 0.036825587 | 0.023957666 |
| HSPA8 | Epi3 | turquoise | 0.178016249 | 0.115484327 | 0.156506856 | 0.322405788 | 0.072434808 | 0.042352516 | 0.142131542 | 0.145683033 | 0.203766057 |
| SIAE | Epi3 | turquoise | 0.081437319 | 0.067119878 | 0.069274939 | 0.153948663 | 0.006084546 | 0.02208638 | 0.043020744 | 0.080353895 | 0.092162462 |
| SPA17 | Epi3 | turquoise | 0.058461857 | 0.04031955 | 0.061917219 | 0.081088223 | 0.023229747 | 0.029893841 | 0.022887213 | 0.06944178 | 0.049409286 |
| THYN1 | Epi3 | turquoise | 0.093299428 | 0.049535398 | 0.07688388 | 0.116752199 | 0.046837374 | 0.022968755 | 0.047982842 | 0.092495976 | 0.07465688 |
| IDI1 | Epi3 | turquoise | 0.055679879 | 0.009778598 | 0.03550951 | 0.080071209 | 0.03575053 | 0.011615822 | 0.060305432 | 0.011756315 | 0.066982334 |
| ATP5C1 | Epi3 | turquoise | 0.144693177 | 0.067299945 | 0.113741339 | 0.275275754 | 0.078939356 | 0.040984151 | 0.13356942 | 0.077446797 | 0.204783687 |
| NUDT5 | Epi3 | turquoise | 0.097751313 | 0.089808281 | 0.062881973 | 0.139029932 | 0.038668965 | 0.023108348 | 0.062037651 | 0.062920352 | 0.071334368 |
| CDC123 | Epi3 | turquoise | 0.075594042 | 0.081270715 | 0.036393221 | 0.167227668 | 0.021392479 | 0.020610705 | 0.060744436 | 0.061700696 | 0.083895673 |
| PHYH | Epi3 | turquoise | 0.044780156 | 0.031520364 | 0.000926966 | 0.08082646 | -0.006092246 | 0.005305454 | 0.064629232 | 0.018522983 | 0.00789174 |
| PRPF18 | Epi3 | turquoise | 0.114619266 | 0.090476357 | 0.066622429 | 0.149383907 | 0.024999354 | 0.028800839 | 0.035264903 | 0.092140491 | 0.064643362 |
| COMMD3 | Epi3 | turquoise | 0.051564533 | 0.053905976 | 0.037570151 | 0.109215494 | -0.007253055 | 0.002850214 | 0.036775378 | 0.07186822 | 0.036126889 |
| ITGB1 | Epi3 | turquoise | 0.158443206 | 0.133396977 | 0.092654186 | 0.155566523 | 0.105472289 | 0.069124358 | 0.1068311 | 0.041062531 | 0.115591972 |
| HNRNPF | Epi3 | turquoise | 0.130536052 | 0.097536006 | 0.090827963 | 0.140384211 | 0.067319708 | 0.074039073 | 0.062928804 | 0.075664652 | 0.081530571 |
| CISD1 | Epi3 | turquoise | 0.076196371 | 0.023454046 | 0.048873365 | 0.093519615 | 0.026746703 | 0.016970233 | 0.049153818 | 0.046970916 | 0.065396498 |
| TFAM | Epi3 | turquoise | 0.095984835 | 0.04821209 | 0.073091534 | 0.115950894 | 0.060322463 | 0.012220876 | 0.055934266 | 0.064426494 | 0.092487007 |
| HERC4 | Epi3 | turquoise | 0.048945689 | 0.028530713 | 0.043661108 | 0.071226922 | 0.005382582 | 0.031165704 | 0.031466175 | 0.040349217 | 0.045283667 |
| HNRNPH3 | Epi3 | turquoise | 0.116486999 | 0.043230158 | 0.090155563 | 0.116065235 | 0.07716029 | 0.037739091 | 0.06641066 | 0.061845136 | 0.076980536 |
| KIF1BP | Epi3 | turquoise | 0.059557764 | 0.033764137 | 0.043889087 | 0.106202718 | 0.014708268 | 0.011654631 | 0.038211691 | 0.028352987 | 0.064704056 |
| VPS26A | Epi3 | turquoise | 0.106920133 | 0.094609356 | 0.058701403 | 0.147439852 | 0.038516091 | 0.020152171 | 0.063929787 | 0.085454763 | 0.084906435 |
| PPA1 | Epi3 | turquoise | 0.208806212 | 0.194365206 | 0.16550095 | 0.312041877 | 0.096599262 | 0.067571458 | 0.101179752 | 0.169831411 | 0.188615492 |
| PCBD1 | Epi3 | turquoise | 0.174871777 | 0.086872447 | 0.159529991 | 0.159009232 | 0.135006237 | 0.037533965 | 0.060061322 | 0.125682318 | 0.110117093 |
| PSAP | Epi3 | turquoise | 0.201474327 | 0.221714034 | 0.098117968 | 0.3872884 | 0.056825904 | 0.083782315 | 0.133616031 | 0.135387795 | 0.22327542 |
| ANXA7 | Epi3 | turquoise | 0.110249004 | 0.10624885 | 0.066698569 | 0.221156987 | 0.004928436 | 0.032718644 | 0.088892085 | 0.088565941 | 0.123385043 |
| CHCHD1 | Epi3 | turquoise | 0.093014929 | 0.075430255 | 0.079412106 | 0.110172874 | 0.051114806 | 0.01838244 | 0.028930836 | 0.076305541 | 0.07224686 |
| COMTD1 | Epi3 | turquoise | 0.075482553 | 0.069993114 | 0.051319625 | 0.112820456 | 0.029659426 | 0.010324694 | 0.038216053 | 0.055539406 | 0.056943256 |
| GHITM | Epi3 | turquoise | 0.139851125 | 0.093506449 | 0.088483105 | 0.208591177 | 0.072454507 | 0.024951525 | 0.104138025 | 0.07058688 | 0.138002033 |
| MINPP1 | Epi3 | turquoise | 0.050617563 | 0.087732566 | 0.016692112 | 0.120910775 | -0.028025363 | 0.000700012 | 0.017119475 | 0.092368544 | 0.011414269 |
| ATAD1 | Epi3 | turquoise | 0.072915819 | 0.075397047 | 0.050345129 | 0.13728666 | 0.038965292 | 0.015334492 | 0.041259125 | 0.038179269 | 0.091308086 |
| LIPA | Epi3 | turquoise | 0.074839086 | 0.105929157 | 0.041788487 | 0.104960884 | 0.015876904 | 0.050816065 | 0.041471588 | 0.0513903 | 0.035063647 |
| RPP30 | Epi3 | turquoise | 0.047809312 | 0.064227984 | 0.02914181 | 0.089585365 | 0.021500068 | 0.024297603 | 0.022831509 | 0.023379509 | 0.043106152 |
| 5-Mar | Epi3 | turquoise | 0.088177097 | 0.041196696 | 0.075095114 | 0.088689592 | 0.040894925 | 0.00465719 | 0.043305486 | 0.069165905 | 0.065679903 |
| TCTN3 | Epi3 | turquoise | 0.070243714 | 0.023351362 | 0.060361904 | 0.073636383 | 0.026715502 | 0.04349559 | 0.051568673 | 0.029515499 | 0.070856025 |
| PGAM1 | Epi3 | turquoise | 0.119497912 | 0.095437432 | 0.075505748 | 0.192921596 | 0.053402313 | 0.042148074 | 0.084008801 | 0.08098301 | 0.116554122 |
| NDUFB8 | Epi3 | turquoise | 0.157132378 | 0.091018114 | 0.134778959 | 0.231903403 | 0.115063623 | 0.02603027 | 0.086260612 | 0.094247846 | 0.181135219 |
| MRPL43 | Epi3 | turquoise | 0.109800312 | 0.044791306 | 0.085774456 | 0.176511472 | 0.105644865 | 0.031577294 | 0.08435358 | 0.049507961 | 0.13788256 |
| ACTR1A | Epi3 | turquoise | 0.049420837 | 0.038296109 | 0.047112669 | 0.080950004 | 0.023378785 | 0.002548523 | 0.014486768 | 0.044182105 | 0.068387864 |
| ARL3 | Epi3 | turquoise | 0.115813128 | 0.072467009 | 0.090951159 | 0.147882701 | 0.035027002 | 0.021333024 | 0.022149482 | 0.125533878 | 0.063529557 |
| BORCS7 | Epi3 | turquoise | 0.144580191 | 0.053479989 | 0.095474721 | 0.152663005 | 0.076955027 | 0.041864577 | 0.111007518 | 0.10943463 | 0.087250068 |
| GSTO1 | Epi3 | turquoise | 0.103414498 | 0.072682388 | 0.082827545 | 0.221890516 | 0.052291239 | 0.005434747 | 0.07546769 | 0.113647502 | 0.125798912 |
| SMC3 | Epi3 | turquoise | 0.058065987 | 0.057000657 | 0.035443134 | 0.088455639 | 0.009755128 | 0.011969207 | 0.015899707 | 0.048612733 | 0.050091585 |
| PDCD4 | Epi3 | turquoise | 0.147671419 | 0.080935754 | 0.127803142 | 0.153998847 | 0.084819032 | 0.100349062 | 0.066262412 | 0.11684863 | 0.111764882 |
| PRDX3 | Epi3 | turquoise | 0.140466696 | 0.094565987 | 0.101512753 | 0.265360708 | 0.048813098 | 0.020037173 | 0.102449027 | 0.118393471 | 0.152885808 |
| NSMCE4A | Epi3 | turquoise | 0.060640667 | 0.044549199 | 0.030917878 | 0.084621144 | 0.015851771 | 0.00620534 | 0.046749124 | 0.021349555 | 0.056784726 |
| OAT | Epi3 | turquoise | 0.060774035 | 0.060953268 | -0.00026803 | 0.236351211 | -0.010697245 | -0.002400132 | 0.11949819 | 0.025215525 | 0.123528869 |
| MGMT | Epi3 | turquoise | 0.068718621 | 0.034083985 | 0.043920628 | 0.1653193 | 0.026630206 | 0.02815201 | 0.06433248 | 0.03093626 | 0.098886883 |
| GLRX3 | Epi3 | turquoise | 0.068775661 | 0.044142003 | 0.049520698 | 0.097872323 | 0.038309112 | 0.051782857 | 0.062430847 | 0.026581169 | 0.061694308 |
| ECHS1 | Epi3 | turquoise | 0.139658223 | 0.13614081 | 0.115345437 | 0.281610496 | 0.001656722 | 0.004773499 | 0.066826703 | 0.183680197 | 0.131358413 |
| FKBP4 | Epi3 | turquoise | 0.070412464 | 0.049942096 | 0.017188607 | 0.107962591 | 0.010098613 | 0.003629927 | 0.081127178 | 0.002904268 | 0.052834115 |
| NDUFA9 | Epi3 | turquoise | 0.090153275 | 0.059837407 | 0.06522457 | 0.183150109 | 0.02685699 | 0.001736452 | 0.084615253 | 0.062937237 | 0.122093472 |
| CD9 | Epi3 | turquoise | 0.151501416 | 0.253671007 | 0.063351908 | 0.524372954 | -0.031784354 | 0.036704412 | 0.135325242 | 0.178492027 | 0.237883487 |
| TAPBPL | Epi3 | turquoise | 0.034676971 | 0.059695996 | -0.022003352 | 0.104312845 | -0.029642784 | 0.022123645 | 0.068569714 | 0.014164261 | 0.022084912 |
| GAPDH | Epi3 | turquoise | 0.382090894 | 0.363710371 | 0.329092899 | 0.512901951 | 0.194561774 | 0.109238807 | 0.076652237 | 0.390404871 | 0.30304526 |
| ING4 | Epi3 | turquoise | 0.060699719 | 0.020816562 | 0.037093749 | 0.104201723 | 0.040681149 | 0.019456372 | 0.033953548 | 0.031102511 | 0.065063806 |
| TPI1 | Epi3 | turquoise | 0.200373601 | 0.215033964 | 0.126871185 | 0.397176659 | 0.04354324 | 0.049897319 | 0.12377433 | 0.192104065 | 0.200938073 |
| EMG1 | Epi3 | turquoise | 0.062859119 | 0.043930799 | 0.055994433 | 0.135077716 | 0.053963869 | 0.00623758 | 0.036026619 | 0.054235881 | 0.09139019 |
| MAGOHB | Epi3 | turquoise | 0.07867845 | 0.036261711 | 0.068950193 | 0.114165761 | 0.055506598 | 0.01735521 | 0.046550923 | 0.034315384 | 0.09820833 |
| HEBP1 | Epi3 | turquoise | 0.077341469 | 0.032505942 | 0.028983229 | 0.137470196 | 0.047480482 | 0.035759534 | 0.111621926 | 0.00927414 | 0.086854265 |
| H2AFJ | Epi3 | turquoise | 0.135692044 | 0.055602133 | 0.154164482 | 0.150181378 | 0.085207105 | 0.010776725 | 0.026908436 | 0.127898017 | 0.136036498 |
| MGP | Epi3 | turquoise | 0.119003711 | 0.255823499 | 0.064917361 | 0.271419773 | -0.094277642 | 0.049972825 | -0.023866244 | 0.262432458 | 0.021534122 |
| STRAP | Epi3 | turquoise | 0.174839101 | 0.080684626 | 0.142117422 | 0.163666708 | 0.118879319 | 0.083789493 | 0.077930472 | 0.111620978 | 0.124078958 |
| MGST1 | Epi3 | turquoise | 0.330410178 | 0.272612048 | 0.304046952 | 0.503084562 | 0.16362405 | 0.08297778 | 0.140799952 | 0.262062028 | 0.369440414 |
| LDHB | Epi3 | turquoise | 0.213047214 | -0.023320656 | 0.228040238 | 0.369721159 | 0.159059014 | 0.050985915 | 0.157863922 | 0.144061174 | 0.346114678 |
| MED21 | Epi3 | turquoise | 0.076209191 | 0.07240924 | 0.043976487 | 0.143094556 | 0.027484887 | 0.008626874 | 0.054076426 | 0.068855466 | 0.092673178 |
| MRPS35 | Epi3 | turquoise | 0.072654183 | 0.060891766 | 0.044058587 | 0.119204068 | 0.017874812 | 0.02947867 | 0.0891019 | 0.043879627 | 0.0699802 |
| ERGIC2 | Epi3 | turquoise | 0.12860029 | 0.096334004 | 0.104490457 | 0.119224656 | 0.049423476 | 0.036286759 | 0.037537677 | 0.102178845 | 0.082144204 |
| PPHLN1 | Epi3 | turquoise | 0.055404545 | 0.001105406 | 0.053047656 | 0.098387991 | 0.038841469 | 0.017314739 | 0.069610757 | 0.017377689 | 0.081171392 |
| PUS7L | Epi3 | turquoise | 0.076895131 | 0.040702023 | 0.057372909 | 0.089591335 | 0.018415534 | 0.014384847 | 0.019275474 | 0.061419874 | 0.050245417 |
| PRKAG1 | Epi3 | turquoise | 0.079008322 | 0.025237644 | 0.047384335 | 0.134718762 | 0.045752612 | 0.017569946 | 0.084221432 | 0.036640567 | 0.094618397 |
| TUBA1B | Epi3 | turquoise | 0.139485558 | 0.115052933 | 0.121130406 | 0.348804102 | 0.033596598 | 0.032748491 | 0.100966812 | 0.144009156 | 0.230470356 |
| TUBA1A | Epi3 | turquoise | 0.075331314 | 0.074945504 | 0.052515943 | 0.117317855 | 0.040459536 | 0.028253337 | 0.070717516 | 0.031443553 | 0.083459205 |
| TUBA1C | Epi3 | turquoise | 0.083424296 | 0.061166539 | 0.07749626 | 0.12858707 | 0.053589773 | 0.01964128 | 0.025434476 | 0.058592308 | 0.094698627 |
| TMBIM6 | Epi3 | turquoise | 0.243824226 | 0.255069934 | 0.158147111 | 0.354808798 | 0.056880857 | 0.081783633 | 0.129025268 | 0.158407884 | 0.190997122 |
| AQP5 | Epi3 | turquoise | 0.071710824 | -0.009751833 | 0.094491938 | 0.086393664 | 0.09386705 | 0.029772159 | 0.032633079 | 0.051703154 | 0.139106288 |
| KRT18 | Epi3 | turquoise | 0.144300953 | 0.256532738 | -0.000789828 | 0.304443466 | -0.009616302 | 0.038622473 | 0.154537098 | 0.064314717 | 0.082680019 |
| C12orf10 | Epi3 | turquoise | 0.092791538 | 0.03517349 | 0.065080526 | 0.116909914 | 0.077940624 | 0.041202181 | 0.051940988 | 0.045229813 | 0.09326033 |
| COPZ1 | Epi3 | turquoise | 0.126935739 | 0.06241939 | 0.078679928 | 0.200397656 | 0.073444304 | 0.045959279 | 0.134161138 | 0.032463335 | 0.155760548 |
| METTL7B | Epi3 | turquoise | 0.055323497 | 0.089121082 | 0.066080941 | 0.153899276 | -0.015077447 | 0.007052752 | -0.048428659 | 0.157587798 | 0.072787066 |
| CD63 | Epi3 | turquoise | 0.327025066 | 0.378494374 | 0.28246023 | 0.636795762 | 0.094338162 | 0.072897474 | 0.1083746 | 0.366563263 | 0.375125721 |
| ORMDL2 | Epi3 | turquoise | 0.112800061 | 0.082892408 | 0.110182128 | 0.187579464 | 0.059151312 | 0.031139283 | 0.043682351 | 0.082824929 | 0.134534407 |
| PA2G4 | Epi3 | turquoise | 0.100773245 | 0.053656992 | 0.042621336 | 0.147954449 | 0.052491702 | 0.038410465 | 0.086904577 | 0.025324716 | 0.106452505 |
| MYL6 | Epi3 | turquoise | 0.338565103 | 0.232593264 | 0.301032602 | 0.497485324 | 0.225172753 | 0.08243043 | 0.240312828 | 0.198320989 | 0.391996744 |
| ATP5B | Epi3 | turquoise | 0.126898788 | 0.099110698 | 0.053008408 | 0.333286518 | 0.032515295 | 0.03604047 | 0.169793964 | 0.069122411 | 0.185510604 |
| PTGES3 | Epi3 | turquoise | 0.227268913 | 0.138311235 | 0.177989604 | 0.256620734 | 0.124559733 | 0.063497634 | 0.089925091 | 0.171474508 | 0.147617333 |
| DCTN2 | Epi3 | turquoise | 0.068769448 | 0.019315765 | 0.076775387 | 0.13344233 | 0.077705448 | 0.013633558 | 0.064397654 | 0.019249026 | 0.112734364 |
| OS9 | Epi3 | turquoise | 0.063301356 | 0.038510974 | 0.024358351 | 0.119756296 | -0.002639368 | 0.03061674 | 0.052417345 | 0.038794013 | 0.066908713 |
| TMBIM4 | Epi3 | turquoise | 0.202820491 | 0.193870738 | 0.1913185 | 0.384878272 | 0.055959686 | 0.07193194 | 0.071601424 | 0.229817819 | 0.224729762 |
| CPSF6 | Epi3 | turquoise | 0.042909743 | 0.029190272 | 0.022681362 | 0.075215318 | 0.013838889 | 0.014701586 | 0.031239459 | 0.009437437 | 0.044550505 |
| CCT2 | Epi3 | turquoise | 0.084665842 | 0.051677344 | 0.040376271 | 0.152903011 | 0.035950208 | 0.022631426 | 0.090395249 | 0.029454186 | 0.09105477 |
| KRR1 | Epi3 | turquoise | 0.053715688 | 0.019271134 | 0.037155078 | 0.104457075 | 0.019038195 | -0.019914495 | 0.060542373 | 0.036777954 | 0.071354641 |
| CSRP2 | Epi3 | turquoise | 0.069122499 | 0.027971935 | 0.009940817 | 0.148834548 | 0.019576218 | 0.017785248 | 0.139470384 | -0.001106703 | 0.086756439 |
| MGAT4C | Epi3 | turquoise | 0.13555004 | 0.034237836 | 0.114150615 | 0.157546403 | 0.013269048 | 0.051283991 | 0.059190906 | 0.146917517 | 0.100327926 |
| NDUFA12 | Epi3 | turquoise | 0.113586898 | 0.03651227 | 0.096341651 | 0.21382526 | 0.087171491 | 0.015223612 | 0.127255919 | 0.060525669 | 0.166633595 |
| METAP2 | Epi3 | turquoise | 0.146625249 | 0.103424092 | 0.102077063 | 0.136484204 | 0.088528564 | 0.035368345 | 0.059996158 | 0.11058755 | 0.071781309 |
| LTA4H | Epi3 | turquoise | 0.089722663 | 0.059254896 | 0.033876436 | 0.09659465 | 0.094066694 | 0.020244429 | 0.086783701 | -0.00252089 | 0.079787094 |
| ACTR6 | Epi3 | turquoise | 0.056178534 | 0.067050154 | 0.039214397 | 0.131398266 | -0.002919979 | 0.014745931 | 0.030009669 | 0.073840273 | 0.073283513 |
| ARL1 | Epi3 | turquoise | 0.126980775 | 0.089564947 | 0.097389619 | 0.213997721 | 0.064828513 | 0.019274842 | 0.10427047 | 0.066459848 | 0.169824492 |
| CCDC53 | Epi3 | turquoise | 0.081560795 | 0.030733244 | 0.053142773 | 0.122423411 | 0.053453231 | 0.000982841 | 0.088566934 | 0.02977141 | 0.075798355 |
| NUP37 | Epi3 | turquoise | 0.052620827 | 0.059133025 | 0.012325934 | 0.146656687 | -0.006676059 | 0.017742019 | 0.059556397 | 0.053962492 | 0.076476093 |
| MMAB | Epi3 | turquoise | 0.072159165 | 0.035932279 | 0.047543459 | 0.106037088 | 0.055023057 | 0.009202598 | 0.070031695 | 0.041234615 | 0.072904195 |
| ARPC3 | Epi3 | turquoise | 0.206903038 | 0.143818686 | 0.169112055 | 0.308801996 | 0.100631091 | 0.049332862 | 0.105871274 | 0.157203621 | 0.216085289 |
| GPN3 | Epi3 | turquoise | 0.067131094 | 0.064485533 | 0.062031108 | 0.147446305 | 0.008753625 | 0.020824765 | 0.022896959 | 0.094903745 | 0.07948517 |
| VPS29 | Epi3 | turquoise | 0.174028326 | 0.138077903 | 0.143621127 | 0.243553778 | 0.060926514 | 0.05814574 | 0.051255886 | 0.164360907 | 0.148164603 |
| TCTN1 | Epi3 | turquoise | 0.090440217 | 0.029470052 | 0.061968791 | 0.110711886 | 0.036653011 | 0.033908288 | 0.077163782 | 0.051789795 | 0.081421864 |
| ERP29 | Epi3 | turquoise | 0.125999516 | 0.140235641 | 0.063764013 | 0.242882605 | 0.089135829 | 0.025489988 | 0.104187011 | 0.052967189 | 0.150795205 |
| FBXO21 | Epi3 | turquoise | 0.091264629 | 0.0334108 | 0.068641166 | 0.107761952 | 0.039721011 | 0.079517624 | 0.053828466 | 0.062494895 | 0.073963018 |
| PEBP1 | Epi3 | turquoise | 0.258680441 | 0.102891972 | 0.148605422 | 0.439815235 | 0.13257644 | 0.057963837 | 0.315900102 | 0.104844588 | 0.284484934 |
| COX6A1 | Epi3 | turquoise | 0.188443866 | 0.130637488 | 0.191951849 | 0.31227286 | 0.116698265 | 0.055935785 | 0.133633468 | 0.131011907 | 0.239233838 |
| COQ5 | Epi3 | turquoise | 0.047653735 | 0.017361348 | 0.015849395 | 0.104129598 | -0.000365834 | 0.009845795 | 0.04912662 | 0.034561224 | 0.066344362 |
| POP5 | Epi3 | turquoise | 0.085831207 | 0.052181405 | 0.084405963 | 0.126621488 | 0.058376799 | 0.046886487 | 0.062554058 | 0.071230271 | 0.091655164 |
| C12orf65 | Epi3 | turquoise | 0.049841437 | 0.027529534 | 0.047300094 | 0.069535523 | 0.000404148 | 0.017130356 | 0.02184531 | 0.052730977 | 0.033181594 |
| SNRNP35 | Epi3 | turquoise | 0.026603851 | 0.022547176 | 0.001055087 | 0.083119891 | 9.08E-05 | 0.005717794 | 0.059072479 | -0.012769276 | 0.047100112 |
| UBC | Epi3 | turquoise | 0.228836187 | 0.218993361 | 0.15575954 | 0.324264072 | 0.012534676 | 0.060133688 | 0.094797573 | 0.224396294 | 0.1521301 |
| RAN | Epi3 | turquoise | 0.186441491 | 0.091309274 | 0.158842022 | 0.277349143 | 0.108414947 | 0.045541469 | 0.110305596 | 0.114230196 | 0.224560629 |
| CRYL1 | Epi3 | turquoise | 0.088484148 | 0.087215022 | 0.042838855 | 0.144423556 | 0.018863993 | 0.024867618 | 0.075984338 | 0.070964314 | 0.054736523 |
| IFT88 | Epi3 | turquoise | 0.039780756 | 0.029285209 | 0.041122836 | 0.053904387 | 0.006919865 | -0.002292435 | 0.023096973 | 0.033257654 | 0.02927331 |
| N6AMT2 | Epi3 | turquoise | 0.060254271 | 0.040558456 | 0.049762302 | 0.073262631 | 0.026571969 | 0.041681518 | 0.024420239 | 0.054538368 | 0.050409227 |
| SAP18 | Epi3 | turquoise | 0.169360633 | 0.117403095 | 0.135186399 | 0.250629968 | 0.099002488 | 0.041053575 | 0.089126161 | 0.110228955 | 0.167483879 |
| MICU2 | Epi3 | turquoise | 0.06999734 | 0.058042407 | 0.05481428 | 0.167493532 | 0.000370246 | 0.017026631 | 0.030785545 | 0.088247967 | 0.0946649 |
| ALG5 | Epi3 | turquoise | 0.131265416 | 0.131945437 | 0.114161702 | 0.208221604 | 0.025667655 | 0.048809851 | 0.044314132 | 0.110170422 | 0.118651257 |
| EXOSC8 | Epi3 | turquoise | 0.028575757 | 0.034996441 | 0.007403651 | 0.091153111 | -0.0221064 | 0.032441042 | 0.044257795 | 0.036559433 | 0.03915574 |
| MRPS31 | Epi3 | turquoise | 0.058940696 | 0.023760805 | 0.036861061 | 0.118188476 | 0.023473457 | -0.008280824 | 0.06611457 | 0.02706724 | 0.07086041 |
| DNAJC15 | Epi3 | turquoise | 0.12045834 | 0.08565653 | 0.104655944 | 0.159887768 | 0.074339398 | 0.034502673 | 0.08087279 | 0.068164113 | 0.134103406 |
| ESD | Epi3 | turquoise | 0.127072806 | 0.000582085 | 0.078991794 | 0.263834045 | 0.089677854 | 0.031064141 | 0.151378866 | 0.053219134 | 0.19480093 |
| SUCLA2 | Epi3 | turquoise | 0.061099989 | 0.039075818 | 0.029492687 | 0.12717989 | 0.009874547 | 0.007354599 | 0.073405375 | 0.026357451 | 0.081379659 |
| MED4 | Epi3 | turquoise | 0.114224737 | 0.071795466 | 0.076309062 | 0.158940283 | 0.056526801 | 0.019473087 | 0.079248262 | 0.061323455 | 0.112960102 |
| ITM2B | Epi3 | turquoise | 0.251479922 | 0.264530009 | 0.137743969 | 0.626609464 | 0.016683579 | 0.047750764 | 0.214567224 | 0.205772811 | 0.354722865 |
| SUGT1 | Epi3 | turquoise | 0.069625176 | 0.007796263 | 0.050876601 | 0.080287276 | 0.039923065 | 0.008362047 | 0.079673378 | 0.012179606 | 0.089411984 |
| CLN5 | Epi3 | turquoise | 0.103880668 | 0.062651502 | 0.098712474 | 0.223422216 | 0.014012108 | 0.040091029 | 0.075685935 | 0.109034636 | 0.123809858 |
| SCEL | Epi3 | turquoise | 0.120351281 | 0.136450484 | 0.078600204 | 0.095344325 | 0.043486172 | 0.046839348 | 0.037237679 | 0.058084903 | 0.050031118 |
| TM9SF2 | Epi3 | turquoise | 0.083617903 | 0.055156181 | 0.057598458 | 0.181272002 | 0.023428405 | 0.015166304 | 0.060433883 | 0.05733568 | 0.123071521 |
| ANKRD10 | Epi3 | turquoise | 0.068397259 | 0.0607837 | 0.027369403 | 0.086154519 | 0.0253555 | 0.050226996 | 0.037543027 | 0.050113733 | 0.036837951 |
| PCID2 | Epi3 | turquoise | 0.030337462 | 0.023783 | 0.009861861 | 0.109211063 | 0.004007547 | 0.002574616 | 0.049444209 | 0.028935125 | 0.04168374 |
| LAMP1 | Epi3 | turquoise | 0.113130432 | 0.089363988 | 0.032227097 | 0.272486161 | 0.038206929 | 0.012210423 | 0.142870701 | 0.023792278 | 0.170103231 |
| HNRNPC | Epi3 | turquoise | 0.143515403 | 0.103294204 | 0.103338519 | 0.163183547 | 0.059343865 | 0.050998354 | 0.054223901 | 0.121607104 | 0.097982113 |
| DAD1 | Epi3 | turquoise | 0.18249218 | 0.109761793 | 0.176948943 | 0.324377326 | 0.081242754 | 0.052106244 | 0.11600246 | 0.15251283 | 0.233808411 |
| MRPL52 | Epi3 | turquoise | 0.166492928 | 0.093257887 | 0.179181629 | 0.213274664 | 0.132037484 | 0.050411698 | 0.08711058 | 0.119966572 | 0.186695077 |
| PSMB5 | Epi3 | turquoise | 0.119874422 | 0.065879021 | 0.082649192 | 0.196399506 | 0.069624353 | 0.053102892 | 0.110721633 | 0.052993163 | 0.137119705 |
| C14orf119 | Epi3 | turquoise | 0.118440153 | 0.133926891 | 0.092305437 | 0.196901514 | 0.029721309 | 0.042264318 | 0.053935002 | 0.121193742 | 0.104419693 |
| NGDN | Epi3 | turquoise | 0.064358547 | 0.05653902 | 0.020723417 | 0.104479435 | 0.003104595 | 0.034749267 | 0.069020948 | 0.035697516 | 0.044172576 |
| AP1G2 | Epi3 | turquoise | 0.026713245 | 0.021276399 | 0.003169469 | 0.060132872 | -0.001680806 | 0.028293927 | 0.038596406 | 0.028572649 | 0.030263403 |
| DHRS4L2 | Epi3 | turquoise | 0.081719088 | 0.055214097 | 0.072372561 | 0.113824318 | 0.063956372 | 0.003158626 | 0.041397645 | 0.05732272 | 0.080592153 |
| PSME1 | Epi3 | turquoise | 0.175586632 | 0.190209031 | 0.091302911 | 0.307036436 | 0.092874181 | 0.050878466 | 0.104762407 | 0.110675146 | 0.169483991 |
| PSME2 | Epi3 | turquoise | 0.15437659 | 0.192862514 | 0.100382879 | 0.214861575 | 0.055418747 | 0.04161529 | 0.099251328 | 0.093697751 | 0.110458597 |
| SCFD1 | Epi3 | turquoise | 0.059327782 | 0.032819017 | 0.043990011 | 0.124584788 | 0.01678842 | 0.019313114 | 0.066148327 | 0.035584916 | 0.073720986 |
| EAPP | Epi3 | turquoise | 0.133703477 | 0.098023706 | 0.08705407 | 0.180582655 | 0.046839868 | 0.042112676 | 0.063174735 | 0.098518758 | 0.091056066 |
| SNX6 | Epi3 | turquoise | 0.092676486 | 0.065804313 | 0.088578148 | 0.124621555 | 0.046266516 | 0.016394534 | 0.032957649 | 0.076701784 | 0.082863617 |
| SRP54 | Epi3 | turquoise | 0.053882153 | 0.067124954 | 0.028158904 | 0.107642354 | 0.001113888 | 0.000652611 | 0.055766745 | 0.044509898 | 0.055024218 |
| PSMA6 | Epi3 | turquoise | 0.057489101 | 0.055961244 | 0.028333878 | 0.095901519 | 0.029346657 | -0.001834972 | 0.043285778 | 0.024434359 | 0.051096426 |
| MBIP | Epi3 | turquoise | 0.160107344 | 0.132067739 | 0.149862544 | 0.266263049 | 0.069919517 | 0.050531956 | 0.058005666 | 0.120651844 | 0.184072215 |
| FKBP3 | Epi3 | turquoise | 0.097971674 | 0.072971188 | 0.061399526 | 0.142678703 | 0.066365521 | 0.019624048 | 0.074432248 | 0.044625481 | 0.091084565 |
| TMX1 | Epi3 | turquoise | 0.099619175 | 0.105437722 | 0.049966907 | 0.160175455 | 0.027054639 | 0.032597838 | 0.081321817 | 0.045657109 | 0.080657113 |
| C14orf166 | Epi3 | turquoise | 0.137553763 | 0.064750699 | 0.096396648 | 0.212824915 | 0.111063102 | 0.044351981 | 0.106342578 | 0.054377909 | 0.157985481 |
| PSMC6 | Epi3 | turquoise | 0.079651364 | 0.054783775 | 0.05515621 | 0.185066788 | 0.018717925 | 0.016200078 | 0.078703701 | 0.05231825 | 0.117419631 |
| CNIH1 | Epi3 | turquoise | 0.10855903 | 0.074862638 | 0.107029582 | 0.127908671 | 0.087666882 | 0.025058701 | 0.005619968 | 0.105194215 | 0.08943975 |
| CGRRF1 | Epi3 | turquoise | 0.080546416 | 0.067418958 | 0.058979987 | 0.122996385 | 0.003043822 | 0.016261671 | 0.012147519 | 0.082436683 | 0.061953842 |
| LGALS3 | Epi3 | turquoise | 0.183141142 | 0.10823345 | 0.238873382 | 0.34917631 | 0.161565733 | 0.043864206 | 0.046431052 | 0.172537309 | 0.331369716 |
| ACTR10 | Epi3 | turquoise | 0.086090351 | 0.066513763 | 0.04739012 | 0.272158043 | 0.021359411 | -0.003345075 | 0.102251744 | 0.071202019 | 0.158061614 |
| PSMA3 | Epi3 | turquoise | 0.127740189 | 0.142087906 | 0.109671998 | 0.257566226 | 0.010316052 | 0.046442613 | 0.071650759 | 0.138658722 | 0.135564145 |
| JKAMP | Epi3 | turquoise | 0.107236934 | 0.117990467 | 0.068404023 | 0.239289827 | -0.009804886 | 0.006719569 | 0.06622501 | 0.121499329 | 0.107602799 |
| DHRS7 | Epi3 | turquoise | 0.1225724 | 0.082602586 | 0.049154514 | 0.231283867 | 0.031888447 | 0.011758308 | 0.162927158 | 0.023065911 | 0.146830128 |
| SNAPC1 | Epi3 | turquoise | 0.053329008 | 0.036092212 | 0.06204407 | 0.092288462 | 0.02180756 | 0.020818609 | 0.012635056 | 0.06742052 | 0.050654649 |
| ATP6V1D | Epi3 | turquoise | 0.129998257 | 0.102128907 | 0.085617811 | 0.157981919 | 0.045797321 | 0.028597859 | 0.044590875 | 0.100161296 | 0.073780567 |
| ARG2 | Epi3 | turquoise | 0.066066316 | 0.070611551 | 0.050678993 | 0.171871529 | -0.007380985 | -0.010979935 | 0.059991708 | 0.076999565 | 0.088886108 |
| ERH | Epi3 | turquoise | 0.209509183 | 0.154819637 | 0.165106541 | 0.306488566 | 0.081837833 | 0.033910093 | 0.096666293 | 0.155367647 | 0.193635396 |
| MED6 | Epi3 | turquoise | 0.065885232 | 0.041701353 | 0.033030498 | 0.100142706 | 0.031019328 | 0.032360795 | 0.052865179 | 0.044548477 | 0.067253597 |
| ISCA2 | Epi3 | turquoise | 0.139341559 | 0.079378942 | 0.143874198 | 0.181025509 | 0.068082675 | 0.032506789 | 0.025776329 | 0.13983723 | 0.116372944 |
| EIF2B2 | Epi3 | turquoise | 0.042013556 | -0.006124629 | 0.003145851 | 0.096090184 | 0.033167032 | 0.034518011 | 0.10660708 | -0.014440976 | 0.064419793 |
| TMED10 | Epi3 | turquoise | 0.144728068 | 0.042490239 | 0.135972072 | 0.258558845 | 0.084043792 | 0.028208721 | 0.124359683 | 0.08904743 | 0.234765814 |
| IFT43 | Epi3 | turquoise | 0.077777337 | 0.048288326 | 0.049784011 | 0.123354229 | 0.028537594 | 0.018068443 | 0.058705239 | 0.05749504 | 0.079397563 |
| AHSA1 | Epi3 | turquoise | 0.068865751 | 0.016697712 | 0.062406549 | 0.118410596 | 0.046980318 | -0.007579955 | 0.066198607 | 0.038240102 | 0.09488672 |
| SLIRP | Epi3 | turquoise | 0.223173473 | 0.155444896 | 0.216264452 | 0.233079019 | 0.156079226 | 0.058158179 | 0.085525106 | 0.168883768 | 0.181078428 |
| SNW1 | Epi3 | turquoise | 0.048088713 | 0.052992526 | 0.014685243 | 0.086081396 | 0.014984846 | 0.005191221 | 0.035210031 | 0.016752568 | 0.033973457 |
| PSMC1 | Epi3 | turquoise | 0.089914179 | 0.074065658 | 0.062314906 | 0.150323841 | 0.064479993 | -0.000243519 | 0.053100416 | 0.056319257 | 0.085431723 |
| LGMN | Epi3 | turquoise | 0.077898062 | 0.091127966 | -0.00034561 | 0.152841616 | -0.004181018 | 0.015807916 | 0.110864794 | 0.036915446 | 0.049583444 |
| DDX24 | Epi3 | turquoise | 0.174619413 | 0.122883845 | 0.136170494 | 0.226335658 | 0.050111907 | 0.067317954 | 0.072191712 | 0.156242216 | 0.124035604 |
| HSP90AA1 | Epi3 | turquoise | 0.247780141 | 0.247574902 | 0.180095195 | 0.402775864 | 0.015164448 | 0.056911644 | 0.097043201 | 0.24564146 | 0.201605664 |
| CINP | Epi3 | turquoise | 0.039151646 | 0.035424023 | 0.01725042 | 0.086239329 | 0.015523743 | 0.009195308 | 0.045225534 | 0.016101025 | 0.05420139 |
| EIF5 | Epi3 | turquoise | 0.127088821 | 0.107791854 | 0.064297026 | 0.134887132 | 0.021121661 | 0.034703907 | 0.099394486 | 0.078296019 | 0.049652204 |
| ZFYVE21 | Epi3 | turquoise | 0.099627654 | 0.087013183 | 0.057495885 | 0.133546148 | 0.0398504 | 0.011144262 | 0.052667254 | 0.071967675 | 0.065165983 |
| NDN | Epi3 | turquoise | 0.045761657 | 0.027335115 | 0.015457618 | 0.088834065 | 0.018932104 | 0.007803883 | 0.055584264 | 0.025923813 | 0.053546153 |
| SCG5 | Epi3 | turquoise | 0.079955831 | 0.093468578 | 0.035603565 | 0.16158049 | 0.006989571 | 0.038643659 | 0.032977836 | 0.045496117 | 0.095963546 |
| EMC7 | Epi3 | turquoise | 0.106793926 | 0.094841915 | 0.082779281 | 0.220731944 | 0.035266291 | 0.018748376 | 0.082308085 | 0.096560743 | 0.128920969 |
| SRP14 | Epi3 | turquoise | 0.322077635 | 0.133865822 | 0.31297807 | 0.41318728 | 0.194035713 | 0.105184128 | 0.172456837 | 0.266805727 | 0.322514915 |
| DNAJC17 | Epi3 | turquoise | 0.048767867 | 0.075873041 | 0.004228051 | 0.100585594 | -0.00374882 | 0.027111201 | 0.020904541 | 0.049695554 | 0.016187191 |
| SPINT1 | Epi3 | turquoise | 0.143150536 | 0.213219162 | 0.079576224 | 0.180040221 | 0.068031443 | 0.033617252 | 0.042142642 | 0.072710763 | 0.114940568 |
| SNAP23 | Epi3 | turquoise | 0.123851706 | 0.013624484 | 0.166452645 | 0.169958871 | 0.108428644 | 0.053947302 | 0.019172242 | 0.118878176 | 0.172414773 |
| CCNDBP1 | Epi3 | turquoise | 0.075987455 | 0.086449381 | 0.022756849 | 0.171551506 | 0.032142569 | 0.032950198 | 0.072811049 | 0.056441534 | 0.073693951 |
| PDIA3 | Epi3 | turquoise | 0.141034674 | 0.187631058 | 0.01802306 | 0.339796488 | -0.032228693 | 0.018310238 | 0.18964205 | 0.044269808 | 0.152333773 |
| MFAP1 | Epi3 | turquoise | 0.059154661 | 0.058840948 | 0.003419263 | 0.117388271 | -0.014922182 | 0.013112293 | 0.06187448 | 0.032421728 | 0.039980966 |
| DUT | Epi3 | turquoise | 0.148461533 | 0.093081643 | 0.106383505 | 0.181598691 | 0.059013208 | 0.063658598 | 0.078766095 | 0.118003077 | 0.102678724 |
| COPS2 | Epi3 | turquoise | 0.07351948 | 0.0046 | 0.040289784 | 0.107476888 | 0.079356099 | 0.026197877 | 0.053757092 | 0.034402153 | 0.07197093 |
| SPPL2A | Epi3 | turquoise | 0.098900403 | 0.067689413 | 0.083481083 | 0.129608699 | 0.046786861 | 0.027529846 | 0.060041685 | 0.05985671 | 0.092408109 |
| CCPG1 | Epi3 | turquoise | 0.145434457 | 0.083990071 | 0.110913386 | 0.16575839 | 0.029002108 | 0.062590427 | 0.059649246 | 0.133005255 | 0.101225499 |
| ANXA2 | Epi3 | turquoise | 0.209583962 | 0.295972846 | 0.128442466 | 0.430627026 | 0.055703223 | 0.043061292 | 0.146062689 | 0.137146696 | 0.249611422 |
| PPIB | Epi3 | turquoise | 0.217433488 | 0.207523296 | 0.164833602 | 0.486401868 | 0.084711219 | 0.034240098 | 0.191951108 | 0.148554735 | 0.329232177 |
| HACD3 | Epi3 | turquoise | 0.152920311 | 0.07872363 | 0.129553824 | 0.300427045 | 0.048496469 | 0.044550574 | 0.098150952 | 0.129836384 | 0.214574741 |
| RAB11A | Epi3 | turquoise | 0.164324128 | 0.059790232 | 0.16895335 | 0.25786191 | 0.049982954 | 0.047578144 | 0.0551035 | 0.199289859 | 0.191361277 |
| PKM | Epi3 | turquoise | 0.190681155 | 0.32898224 | 0.104597881 | 0.373393235 | -0.036812592 | 0.053505376 | 0.036338233 | 0.247957796 | 0.114505965 |
| HEXA | Epi3 | turquoise | 0.070678525 | 0.10702101 | 0.025215146 | 0.233993199 | -0.042412022 | -0.013685527 | 0.051701144 | 0.090479979 | 0.093672768 |
| NPTN | Epi3 | turquoise | 0.154050508 | 0.139273853 | 0.130846951 | 0.251456898 | 0.073233093 | 0.054257018 | 0.080021528 | 0.125763721 | 0.159873063 |
| ULK3 | Epi3 | turquoise | 0.029533248 | 0.03959088 | 0.017650699 | 0.031561835 | -0.018175948 | 0.005729764 | 0.007005954 | 0.041954812 | 0.002891796 |
| SCAMP2 | Epi3 | turquoise | 0.068627209 | 0.039432896 | 0.052320611 | 0.11510473 | 0.03131742 | 0.009244724 | 0.038797589 | 0.026557474 | 0.069160262 |
| COX5A | Epi3 | turquoise | 0.153743259 | 0.085786161 | 0.124398017 | 0.198063067 | 0.099685583 | 0.028487922 | 0.113610251 | 0.06720511 | 0.149199648 |
| COMMD4 | Epi3 | turquoise | 0.059254671 | 0.071360762 | 0.016445298 | 0.10467943 | 0.002794281 | -0.002066067 | 0.053334771 | 0.043877904 | 0.036726732 |
| SNUPN | Epi3 | turquoise | 0.067582626 | 0.078451181 | 0.054105275 | 0.120746398 | -0.000807725 | 0.015783207 | 0.024837341 | 0.074474499 | 0.063144619 |
| IMP3 | Epi3 | turquoise | 0.132175148 | 0.132956511 | 0.127624902 | 0.178763094 | 0.042078246 | 0.032401949 | 0.01576194 | 0.136691419 | 0.106652339 |
| ETFA | Epi3 | turquoise | 0.083024293 | 0.010111298 | 0.04646908 | 0.128034328 | 0.071514813 | 0.014495409 | 0.10813434 | -0.020732707 | 0.123887249 |
| WDR61 | Epi3 | turquoise | 0.056223259 | 0.041707011 | -0.000365489 | 0.136376769 | -0.001126159 | 0.008320017 | 0.073175302 | 0.02375649 | 0.059471219 |
| PSMA4 | Epi3 | turquoise | 0.172978283 | 0.144084313 | 0.116662925 | 0.346572867 | 0.068097252 | 0.011914008 | 0.129936645 | 0.127950532 | 0.214607895 |
| MORF4L1 | Epi3 | turquoise | 0.250621111 | 0.175370067 | 0.231056368 | 0.285700439 | 0.132486891 | 0.104985177 | 0.060141962 | 0.266425253 | 0.174353518 |
| CTSH | Epi3 | turquoise | 0.089034362 | 0.064130275 | 0.163783281 | 0.264958131 | 0.024134531 | 0.003791806 | -0.060006809 | 0.201520479 | 0.192952498 |
| TMED3 | Epi3 | turquoise | 0.171265933 | 0.133153234 | 0.149768631 | 0.215863605 | 0.11348934 | 0.061049144 | 0.056116143 | 0.121760776 | 0.16518708 |
| ZFAND6 | Epi3 | turquoise | 0.194905032 | 0.144927478 | 0.169468627 | 0.232448339 | 0.079409913 | 0.076465476 | 0.038231835 | 0.193262065 | 0.134115835 |
| FAH | Epi3 | turquoise | 0.041981292 | 0.081289805 | 0.021214377 | 0.101052196 | -0.014170926 | 0.016532706 | -0.000243983 | 0.05806308 | 0.02880452 |
| MESDC2 | Epi3 | turquoise | 0.125235281 | 0.057852279 | 0.076788864 | 0.227595727 | 0.054611827 | 0.028667023 | 0.14547101 | 0.024872542 | 0.173723794 |
| FAM103A1 | Epi3 | turquoise | 0.100430506 | 0.047600883 | 0.083253519 | 0.09521181 | 0.060907924 | 0.042018084 | 0.039097998 | 0.067559774 | 0.076195982 |
| MRPL46 | Epi3 | turquoise | 0.073435815 | 0.052976051 | 0.0591108 | 0.110330809 | 0.046863508 | 0.00910845 | 0.058883482 | 0.061454719 | 0.059635691 |
| MRPS11 | Epi3 | turquoise | 0.069110958 | 0.040787702 | 0.039487917 | 0.090307071 | 0.039258395 | 0.022502802 | 0.056878686 | 0.026024459 | 0.048770967 |
| MFGE8 | Epi3 | turquoise | 0.129146623 | 0.196814914 | 0.090221322 | 0.232422589 | 0.009759879 | 0.031503777 | -0.009853406 | 0.172386372 | 0.08578989 |
| CIB1 | Epi3 | turquoise | 0.175318871 | 0.149909869 | 0.177299306 | 0.249914926 | 0.067429027 | 0.062924329 | 0.025724676 | 0.195265917 | 0.148248858 |
| VIMP | Epi3 | turquoise | 0.119359216 | 0.072373786 | 0.087580322 | 0.201287349 | 0.056321991 | 0.007595635 | 0.080150982 | 0.051094525 | 0.14448446 |
| SNRPA1 | Epi3 | turquoise | 0.066286489 | 0.04003556 | 0.040772476 | 0.088440459 | 0.009687114 | 0.005702077 | 0.028334241 | 0.065233849 | 0.035841968 |
| TM2D3 | Epi3 | turquoise | 0.077259115 | 0.061898868 | 0.045888169 | 0.150415329 | 0.001435049 | 0.010654002 | 0.037778166 | 0.077531275 | 0.059942994 |
| POLR3K | Epi3 | turquoise | 0.056090767 | 0.01952112 | 0.047677267 | 0.0677203 | 0.031531275 | 0.026118985 | 0.042900372 | 0.032216975 | 0.053600361 |
| MRPL28 | Epi3 | turquoise | 0.084964175 | 0.102727301 | 0.039450082 | 0.127269716 | 0.023185989 | -0.002278552 | 0.052679197 | 0.048035453 | 0.061547838 |
| RHOT2 | Epi3 | turquoise | 0.045012346 | 0.053994642 | 0.020177735 | 0.072317594 | 0.008371653 | 0.015571499 | 0.015312273 | 0.051577618 | 0.037322599 |
| NME3 | Epi3 | turquoise | 0.127574344 | 0.138192728 | 0.120275218 | 0.192175115 | 0.007207217 | 0.046983101 | -0.006299856 | 0.177465943 | 0.076398051 |
| MRPS34 | Epi3 | turquoise | 0.124613951 | 0.074755043 | 0.095945298 | 0.146808507 | 0.076971659 | 0.044555657 | 0.064107227 | 0.085440431 | 0.102651442 |
| NUBP2 | Epi3 | turquoise | 0.067397765 | 0.064726458 | 0.037105781 | 0.100825498 | 0.046134488 | 0.018552971 | 0.063591715 | 0.023119257 | 0.051041087 |
| NDUFB10 | Epi3 | turquoise | 0.15196411 | 0.092538834 | 0.106050512 | 0.17061432 | 0.101517007 | 0.045144374 | 0.071527021 | 0.080255902 | 0.131350832 |
| ECI1 | Epi3 | turquoise | 0.080335737 | 0.038154542 | 0.067969679 | 0.10714873 | 0.053698031 | 0.025222903 | 0.050033498 | 0.04644673 | 0.06856147 |
| NMRAL1 | Epi3 | turquoise | 0.040040243 | 0.038555879 | 0.014269888 | 0.108100019 | 0.019388229 | -0.004321715 | 0.048250567 | 0.008217966 | 0.072613288 |
| NUDT16L1 | Epi3 | turquoise | 0.118035073 | 0.109288773 | 0.087295864 | 0.166628404 | 0.040129588 | 0.018296595 | 0.046120669 | 0.114541094 | 0.082976789 |
| C16orf89 | Epi3 | turquoise | 0.051726219 | 0.086314685 | -0.073942889 | 0.230791716 | -0.020434653 | -0.008542243 | 0.162452327 | -0.034577957 | 0.063206129 |
| NUBP1 | Epi3 | turquoise | 0.069242704 | 0.052780836 | 0.05863498 | 0.09219925 | 0.025725773 | 0.043738527 | 0.017935202 | 0.06868556 | 0.046576851 |
| RSL1D1 | Epi3 | turquoise | 0.103865157 | 0.028602726 | 0.065815359 | 0.115273348 | 0.101160546 | 0.030242006 | 0.085458009 | 0.027543252 | 0.102369375 |
| FOPNL | Epi3 | turquoise | 0.056305898 | 0.001075086 | 0.039666826 | 0.066382828 | 0.037379923 | 0.021221713 | 0.043153914 | 0.02202735 | 0.05902901 |
| ARL6IP1 | Epi3 | turquoise | 0.179546073 | 0.097933456 | 0.115447196 | 0.214175771 | 0.086976871 | 0.064070217 | 0.106357615 | 0.103443592 | 0.145548179 |
| UQCRC2 | Epi3 | turquoise | 0.058179736 | 0.001635555 | 0.023022039 | 0.15119617 | 0.063119177 | -0.026500154 | 0.128692937 | -0.022004041 | 0.127665944 |
| NDUFAB1 | Epi3 | turquoise | 0.152755309 | 0.1111931 | 0.128966849 | 0.172493724 | 0.093089098 | 0.06164999 | 0.061668913 | 0.115237174 | 0.116558303 |
| NSMCE1 | Epi3 | turquoise | 0.114609256 | 0.072152104 | 0.089997964 | 0.147593675 | 0.037360689 | 0.031979939 | 0.060108821 | 0.092279522 | 0.075892484 |
| CLN3 | Epi3 | turquoise | 0.045667046 | 0.03626615 | 0.014607142 | 0.09190466 | -0.014901235 | 0.001367034 | 0.067816203 | 0.033804131 | 0.03717903 |
| SULT1A1 | Epi3 | turquoise | 0.100848711 | 0.037097415 | 0.124244639 | 0.122989011 | 0.028446377 | 0.045841244 | -0.006440711 | 0.137369907 | 0.093225136 |
| TUFM | Epi3 | turquoise | 0.077558827 | 0.064971105 | 0.039503762 | 0.192360364 | 0.0423814 | 0.027674021 | 0.067256837 | 0.060742789 | 0.094331346 |
| MVP | Epi3 | turquoise | 0.084323102 | 0.155868503 | 0.069417228 | 0.162676818 | -0.005714825 | 0.022085669 | -0.004278661 | 0.130497982 | 0.063236513 |
| ALDOA | Epi3 | turquoise | 0.230736812 | 0.216921394 | 0.146614065 | 0.424283272 | 0.079731555 | 0.060816906 | 0.160367882 | 0.167889413 | 0.230937045 |
| PPP4C | Epi3 | turquoise | 0.114995491 | 0.094007139 | 0.111133263 | 0.14763376 | 0.061866325 | 0.042600228 | 0.053056849 | 0.096925443 | 0.10560517 |
| CD2BP2 | Epi3 | turquoise | 0.077828363 | 0.037205808 | 0.060060688 | 0.112404392 | 0.044642196 | -0.008830801 | 0.06034625 | 0.061442618 | 0.070703636 |
| DCTPP1 | Epi3 | turquoise | 0.097458061 | 0.067240816 | 0.074000403 | 0.127630232 | 0.034525433 | 0.019563786 | 0.007640442 | 0.095659149 | 0.067557056 |
| PRSS8 | Epi3 | turquoise | 0.059447855 | 0.053587019 | 0.011278926 | 0.138621223 | 0.037684722 | 0.010081013 | 0.112257379 | -0.029955377 | 0.094546626 |
| TGFB1I1 | Epi3 | turquoise | 0.066768482 | 0.035394349 | 0.073819813 | 0.095208889 | 0.062384669 | 0.037317758 | 0.013581066 | 0.057280169 | 0.09280831 |
| DNAJA2 | Epi3 | turquoise | 0.105277686 | 0.060631778 | 0.084504331 | 0.13664437 | 0.060713456 | 0.030975179 | 0.076676505 | 0.083816309 | 0.077469322 |
| ITFG1 | Epi3 | turquoise | 0.094075834 | 0.082224862 | 0.065148838 | 0.149047844 | 0.025902925 | 0.014044802 | 0.048579294 | 0.083221514 | 0.082789687 |
| HERPUD1 | Epi3 | turquoise | 0.118278972 | 0.140170548 | 0.010717702 | 0.255882513 | -0.036964815 | 0.043376333 | 0.13774462 | 0.081750853 | 0.074935841 |
| COQ9 | Epi3 | turquoise | 0.060477997 | 0.049940412 | 0.023122196 | 0.10575986 | 0.012931926 | -0.004470295 | 0.054719427 | 0.022285008 | 0.040639746 |
| POLR2C | Epi3 | turquoise | 0.050002731 | 0.065955828 | 0.018072709 | 0.114464879 | 0.004964255 | -0.009059355 | 0.061992307 | 0.044022207 | 0.038713821 |
| NAE1 | Epi3 | turquoise | 0.101533214 | 0.110362443 | 0.070616658 | 0.176290555 | -0.011060042 | 0.026606811 | 0.026964825 | 0.126237582 | 0.084514892 |
| FAM96B | Epi3 | turquoise | 0.169804736 | 0.125464058 | 0.153099223 | 0.201275963 | 0.10838118 | 0.019855482 | 0.079198371 | 0.123864974 | 0.137015074 |
| ATP6V0D1 | Epi3 | turquoise | 0.094030994 | 0.097727087 | 0.048618568 | 0.22650249 | -0.002576642 | 0.002553885 | 0.076231532 | 0.076492224 | 0.1091034 |
| PSMB10 | Epi3 | turquoise | 0.026076181 | 0.050007781 | 0.010890078 | 0.076668882 | -0.003064779 | -0.000250198 | 0.026875791 | 0.01918577 | 0.043185879 |
| NQO1 | Epi3 | turquoise | 0.076586231 | 0.125526753 | 0.074094686 | 0.161603722 | -0.052713388 | 0.019458181 | -0.064989245 | 0.182943924 | 0.032742628 |
| NOB1 | Epi3 | turquoise | 0.052346566 | 0.03658092 | 0.014905928 | 0.08132699 | 0.038878771 | 0.018804081 | 0.039215055 | 0.018975189 | 0.039177332 |
| PSMD7 | Epi3 | turquoise | 0.072923759 | 0.076998585 | 0.04817646 | 0.101113923 | 0.019643938 | 0.031539946 | 0.043645222 | 0.050851735 | 0.041520834 |
| GABARAPL2 | Epi3 | turquoise | 0.211413778 | 0.106593055 | 0.194405683 | 0.281412905 | 0.105736942 | 0.072357019 | 0.054200948 | 0.224425051 | 0.178928671 |
| KARS | Epi3 | turquoise | 0.066556521 | 0.043285772 | 0.032310494 | 0.104281628 | 0.043016128 | -0.003091468 | 0.076195057 | 0.017933916 | 0.072401828 |
| TERF2IP | Epi3 | turquoise | 0.133259699 | 0.094766771 | 0.086815521 | 0.232192678 | 0.034786306 | 0.042863567 | 0.074573726 | 0.108223344 | 0.116121614 |
| MPHOSPH6 | Epi3 | turquoise | 0.043275613 | -0.009589737 | 0.02151363 | 0.080550416 | 0.014245235 | -0.00509235 | 0.049603502 | 0.019680535 | 0.059866461 |
| HSBP1 | Epi3 | turquoise | 0.154460856 | 0.056993977 | 0.107489785 | 0.188962889 | 0.118580986 | 0.024386778 | 0.170154521 | 0.043070139 | 0.146449351 |
| CYBA | Epi3 | turquoise | 0.233333299 | 0.236718266 | 0.205248417 | 0.398813429 | 0.045858843 | 0.070732691 | 0.066824954 | 0.268575658 | 0.207838316 |
| APRT | Epi3 | turquoise | 0.114001618 | 0.076055738 | 0.097618103 | 0.192656711 | 0.091842481 | 0.0101743 | 0.090114134 | 0.066063656 | 0.143191253 |
| TRAPPC2L | Epi3 | turquoise | 0.09669715 | 0.067718651 | 0.070171795 | 0.139209865 | 0.074424377 | 0.030216666 | 0.080670035 | 0.038598418 | 0.100202198 |
| TCF25 | Epi3 | turquoise | 0.138935712 | 0.086409391 | 0.073168663 | 0.171031338 | 0.077401512 | 0.035821763 | 0.104036248 | 0.072002799 | 0.101296406 |
| GLOD4 | Epi3 | turquoise | 0.09287402 | 0.097788044 | 0.055789461 | 0.192219706 | -0.001974336 | 0.020136609 | 0.079764778 | 0.070766038 | 0.083537025 |
| UBE2G1 | Epi3 | turquoise | 0.091952536 | 0.061398607 | 0.078127274 | 0.095110842 | 0.046613201 | 0.04649247 | 0.047936104 | 0.068446563 | 0.071828519 |
| MED11 | Epi3 | turquoise | 0.069595247 | 0.078970678 | 0.041658246 | 0.106717126 | 0.024607742 | 0.003159315 | 0.054688292 | 0.052654713 | 0.060466638 |
| PSMB6 | Epi3 | turquoise | 0.138428899 | 0.099433745 | 0.115483843 | 0.265784788 | 0.065233221 | 0.058449983 | 0.080560818 | 0.11347037 | 0.178340689 |
| PFN1 | Epi3 | turquoise | 0.308359649 | 0.195808446 | 0.32583709 | 0.354073501 | 0.266980045 | 0.078679828 | 0.115387335 | 0.209385185 | 0.344638638 |
| SPAG7 | Epi3 | turquoise | 0.109311244 | 0.069312382 | 0.078027608 | 0.155975548 | 0.092151074 | 0.01666282 | 0.096031841 | 0.050039448 | 0.103061463 |
| C1QBP | Epi3 | turquoise | 0.114201523 | 0.127864647 | 0.098166161 | 0.206016005 | 0.03165685 | 0.0146179 | 0.05288351 | 0.126319855 | 0.109098852 |
| DERL2 | Epi3 | turquoise | 0.073463544 | 0.081861472 | 0.037360293 | 0.133057642 | 0.011790869 | 0.013236143 | 0.080370897 | 0.047778541 | 0.078335075 |
| MED31 | Epi3 | turquoise | 0.108604119 | 0.108505311 | 0.107865922 | 0.140378843 | -0.001731838 | 0.058078633 | -0.019219331 | 0.156981138 | 0.05394847 |
| ACADVL | Epi3 | turquoise | 0.111607378 | 0.07967254 | 0.080894019 | 0.180618444 | 0.040299317 | 0.037378414 | 0.054852555 | 0.086724941 | 0.117210238 |
| CLDN7 | Epi3 | turquoise | 0.192577157 | 0.225761802 | 0.122178671 | 0.249729018 | 0.132235854 | 0.048155109 | 0.112963073 | 0.038749417 | 0.195243726 |
| GPS2 | Epi3 | turquoise | 0.062985696 | 0.043982721 | 0.048376304 | 0.06819633 | 0.012296889 | 0.019652598 | 0.03786197 | 0.042052157 | 0.036319793 |
| EIF4A1 | Epi3 | turquoise | 0.109442529 | 0.127223905 | 0.064745958 | 0.162258828 | 0.024121902 | 0.014727808 | 0.028190662 | 0.103140842 | 0.060612124 |
| MPDU1 | Epi3 | turquoise | 0.054989676 | 0.034481205 | 0.028299534 | 0.089164554 | 0.023327081 | 0.016834383 | 0.050851859 | 0.02322985 | 0.061418354 |
| STX8 | Epi3 | turquoise | 0.080769277 | 0.039118328 | 0.072912105 | 0.140659173 | 0.067495853 | 0.038839215 | 0.073406986 | 0.031884273 | 0.116445767 |
| UBB | Epi3 | turquoise | 0.305304446 | 0.207688365 | 0.247677812 | 0.492926994 | 0.116681344 | 0.099287744 | 0.206799863 | 0.24470125 | 0.323479931 |
| COPS3 | Epi3 | turquoise | 0.06018302 | 0.043898183 | 0.030698747 | 0.101143002 | 0.029405066 | 0.018177315 | 0.047250525 | 0.01953368 | 0.059741248 |
| RASD1 | Epi3 | turquoise | 0.045383665 | 0.029290257 | 0.030685141 | 0.102372515 | -0.017254076 | 0.010387469 | 0.017834464 | 0.080792812 | 0.040200488 |
| ATPAF2 | Epi3 | turquoise | 0.060769909 | 0.03686283 | 0.024653423 | 0.059018066 | 0.019042713 | 0.003079767 | 0.031351433 | 0.015494537 | 0.020176453 |
| TVP23B | Epi3 | turquoise | 0.071047217 | 0.031262287 | 0.05196135 | 0.120582252 | 0.050454674 | 0.018544901 | 0.074968567 | 0.037377069 | 0.093500317 |
| B9D1 | Epi3 | turquoise | 0.07868872 | 0.037722217 | 0.054852765 | 0.133102527 | 0.030294019 | 0.018231956 | 0.062022071 | 0.028790274 | 0.089287853 |
| DHRS7B | Epi3 | turquoise | 0.060976281 | 0.044714306 | 0.023353991 | 0.103119708 | 0.011155624 | 0.028046343 | 0.064987306 | 0.023189391 | 0.058534383 |
| TMEM199 | Epi3 | turquoise | 0.050234077 | 0.05539831 | 0.039157676 | 0.098551383 | 0.013334148 | 0.007258449 | 0.02656692 | 0.042254458 | 0.063093754 |
| SDF2 | Epi3 | turquoise | 0.101190965 | 0.095621625 | 0.088806072 | 0.163273915 | 0.039992817 | 0.048843762 | 0.022887832 | 0.10955209 | 0.08144897 |
| ZNF207 | Epi3 | turquoise | 0.098880032 | 0.056132573 | 0.062330977 | 0.117310255 | 0.049275075 | 0.037018555 | 0.059138274 | 0.058017877 | 0.055197614 |
| PSMD11 | Epi3 | turquoise | 0.069211499 | 0.045900791 | 0.033397454 | 0.097748389 | 0.014083274 | 0.01497671 | 0.050420771 | 0.052044443 | 0.055395461 |
| PSMB3 | Epi3 | turquoise | 0.187694314 | 0.151077317 | 0.153202988 | 0.315434365 | 0.065694211 | 0.073473276 | 0.096949567 | 0.166331248 | 0.190758784 |
| PSMD3 | Epi3 | turquoise | 0.058610344 | 0.050700853 | 0.025710609 | 0.112089418 | -0.004829241 | 0.013522789 | 0.057158857 | 0.033056916 | 0.038234511 |
| EIF1 | Epi3 | turquoise | 0.346212033 | 0.227861811 | 0.281694477 | 0.363324139 | 0.238231628 | 0.084797136 | 0.157886922 | 0.218312152 | 0.240657294 |
| RAB5C | Epi3 | turquoise | 0.134998067 | 0.091242837 | 0.099636706 | 0.141645864 | 0.081069808 | 0.065893853 | 0.060613872 | 0.085582934 | 0.095971746 |
| COASY | Epi3 | turquoise | 0.066074678 | 0.067097913 | 0.043478661 | 0.106557676 | 0.022228822 | 0.020764025 | 0.048818403 | 0.057919642 | 0.055239264 |
| MLX | Epi3 | turquoise | 0.07366584 | 0.066033863 | 0.037772109 | 0.099053489 | 0.03274184 | 0.024353027 | 0.018495816 | 0.052291807 | 0.052567234 |
| VPS25 | Epi3 | turquoise | 0.077783143 | 0.042785311 | 0.059325533 | 0.14610762 | 0.018574501 | 0.0366092 | 0.032623843 | 0.079733357 | 0.068345129 |
| COA3 | Epi3 | turquoise | 0.122902512 | 0.072416419 | 0.128981396 | 0.137846443 | 0.068927911 | 0.048875324 | 0.048437894 | 0.120432174 | 0.102288449 |
| ARL4D | Epi3 | turquoise | 0.09662657 | 0.089606926 | 0.00874666 | 0.113468385 | 0.034007881 | 0.015043585 | 0.084956759 | -0.004305101 | 0.061684327 |
| TMEM101 | Epi3 | turquoise | 0.017048223 | 0.045583962 | -0.006966036 | 0.066135716 | -3.01E-05 | 0.008770434 | 0.049989711 | 0.000202964 | 0.012292645 |
| GRN | Epi3 | turquoise | 0.13080008 | 0.19697347 | 0.072627752 | 0.251784899 | -0.028226587 | 0.056144819 | 0.060137617 | 0.145513245 | 0.083644213 |
| NMT1 | Epi3 | turquoise | 0.148839391 | 0.14732625 | 0.084874226 | 0.162512984 | 0.051036891 | 0.051889478 | 0.051188906 | 0.100677792 | 0.079326357 |
| SCRN2 | Epi3 | turquoise | 0.052082305 | 0.020733019 | 0.02415033 | 0.095680591 | 0.012551006 | 0.011386837 | 0.032734062 | 0.021877325 | 0.061200279 |
| CDK5RAP3 | Epi3 | turquoise | 0.049911326 | 0.011846154 | 0.023384943 | 0.131112871 | 0.017841162 | -0.014008635 | 0.086213081 | 0.00929407 | 0.092890039 |
| ATP5G1 | Epi3 | turquoise | 0.148508346 | 0.044250982 | 0.139681415 | 0.191095111 | 0.096731705 | 0.030807893 | 0.120391435 | 0.078435567 | 0.171890808 |
| PHB | Epi3 | turquoise | 0.115681712 | 0.072382596 | 0.075722436 | 0.152492971 | 0.058784789 | 0.041767249 | 0.081036301 | 0.069377886 | 0.086231913 |
| SLC35B1 | Epi3 | turquoise | 0.092858243 | 0.060920713 | 0.067689421 | 0.110348661 | 0.031674312 | 0.033508569 | 0.047889159 | 0.059904345 | 0.086201885 |
| PDK2 | Epi3 | turquoise | 0.049461688 | 0.018202328 | 0.021934789 | 0.0957783 | 0.013653745 | 0.003169179 | 0.076873643 | 0.022627752 | 0.058721357 |
| MRPL27 | Epi3 | turquoise | 0.14063921 | 0.125209069 | 0.12582644 | 0.151749467 | 0.070577246 | 0.044338229 | 0.038954492 | 0.098343433 | 0.114325434 |
| LUC7L3 | Epi3 | turquoise | 0.105787996 | 0.038119834 | 0.079221908 | 0.127639755 | 0.048215121 | 0.065399377 | 0.065835224 | 0.054327501 | 0.085655906 |
| NME1 | Epi3 | turquoise | 0.101466635 | 0.091444064 | 0.090172259 | 0.174528292 | 0.048281979 | 0.016396417 | 0.056209392 | 0.092528354 | 0.11386353 |
| SCPEP1 | Epi3 | turquoise | 0.101944299 | 0.076856469 | 0.036927761 | 0.283879897 | 0.003339495 | 0.013608486 | 0.149294293 | 0.049822675 | 0.157719489 |
| MRPS23 | Epi3 | turquoise | 0.076209084 | 0.067451508 | 0.049651874 | 0.139838489 | 0.035607254 | 0.017151662 | 0.070610142 | 0.047839469 | 0.074261028 |
| SUPT4H1 | Epi3 | turquoise | 0.089630237 | 0.059324843 | 0.071559905 | 0.110933948 | 0.054119797 | 0.006501425 | 0.048801475 | 0.044897617 | 0.084760824 |
| CCDC47 | Epi3 | turquoise | 0.09524285 | 0.116059909 | 0.056015226 | 0.109689383 | -0.00618086 | 0.038212617 | 0.015682389 | 0.079627981 | 0.036559679 |
| DDX5 | Epi3 | turquoise | 0.224509844 | 0.220305335 | 0.129595422 | 0.286206461 | 0.017855543 | 0.070282753 | 0.087705999 | 0.188007056 | 0.131058746 |
| ABCA5 | Epi3 | turquoise | 0.054600784 | 0.012485974 | 0.059844604 | 0.099424617 | 0.017526541 | 0.024869883 | 0.036513135 | 0.05541827 | 0.107927177 |
| ICT1 | Epi3 | turquoise | 0.054740327 | 0.042237358 | 0.038372364 | 0.094570415 | 0.0378516 | 0.048235407 | 0.047911599 | 0.022370731 | 0.082236725 |
| ATP5H | Epi3 | turquoise | 0.137896622 | 0.068188921 | 0.109584545 | 0.217458798 | 0.089665149 | 0.001290109 | 0.124527579 | 0.075256743 | 0.159441758 |
| NT5C | Epi3 | turquoise | 0.121346247 | 0.079194817 | 0.097998155 | 0.134871689 | 0.090305859 | 0.025959029 | 0.058324804 | 0.094373209 | 0.095457429 |
| MRPS7 | Epi3 | turquoise | 0.091358388 | 0.102117299 | 0.042967847 | 0.119363968 | 0.030951487 | 0.003478592 | 0.047687023 | 0.050729983 | 0.055391514 |
| SAP30BP | Epi3 | turquoise | 0.088019461 | 0.063513319 | 0.043410619 | 0.119061312 | 0.01890346 | 0.055042326 | 0.065308963 | 0.05820353 | 0.049487795 |
| WBP2 | Epi3 | turquoise | 0.071753932 | 0.080968053 | 0.051896019 | 0.132511665 | 0.020765154 | 0.012000098 | 0.032155882 | 0.077886295 | 0.048717048 |
| PRPSAP1 | Epi3 | turquoise | 0.037212143 | 0.023785078 | -0.014137021 | 0.075506009 | 0.001694759 | 0.010597075 | 0.077159532 | -0.004918713 | 0.034011778 |
| METTL23 | Epi3 | turquoise | 0.068816901 | 0.040856556 | 0.042575775 | 0.116165631 | 0.036695917 | 0.009174391 | 0.050041563 | 0.040477186 | 0.065347216 |
| SRSF2 | Epi3 | turquoise | 0.138578841 | 0.074593512 | 0.109321457 | 0.124591663 | 0.063382528 | 0.041631616 | 0.061587235 | 0.098905804 | 0.07956195 |
| SYNGR2 | Epi3 | turquoise | 0.136387317 | 0.140438546 | 0.10708892 | 0.170400852 | 0.048281194 | 0.02412658 | 0.031894671 | 0.123791846 | 0.088177785 |
| LGALS3BP | Epi3 | turquoise | 0.16936667 | 0.304373569 | 0.098320567 | 0.431147636 | -0.019307641 | 0.01212994 | 0.073959987 | 0.18665898 | 0.209040572 |
| EIF4A3 | Epi3 | turquoise | 0.098615374 | 0.106496823 | 0.034471642 | 0.167302519 | 0.021780947 | 0.073813382 | 0.074643486 | 0.057292526 | 0.064381143 |
| ACTG1 | Epi3 | turquoise | 0.303592196 | 0.199537463 | 0.184436205 | 0.355082562 | 0.243708494 | 0.078740499 | 0.237042055 | 0.07032227 | 0.300588031 |
| ASPSCR1 | Epi3 | turquoise | 0.049016102 | 0.054169132 | -0.000263296 | 0.090323527 | -0.025605536 | -0.002619486 | 0.071782704 | 0.034364077 | 0.027840839 |
| DCXR | Epi3 | turquoise | 0.08136263 | 0.030632941 | 0.034059539 | 0.187136431 | 0.015732682 | 0.010261306 | 0.123159418 | 0.02679129 | 0.100823859 |
| GPS1 | Epi3 | turquoise | 0.052623127 | 0.024948562 | 0.034539968 | 0.09515294 | 0.032551288 | 0.009475078 | 0.045177941 | 0.008229537 | 0.08655035 |
| USP14 | Epi3 | turquoise | 0.075604045 | 0.02995844 | 0.074819038 | 0.083274318 | 0.044927535 | 0.03192278 | 0.014475493 | 0.049691799 | 0.078609216 |
| ENOSF1 | Epi3 | turquoise | 0.078218267 | 0.023264898 | 0.069752271 | 0.087010285 | 0.022423066 | 0.025694114 | 0.049459712 | 0.053816812 | 0.057249389 |
| MYL12A | Epi3 | turquoise | 0.287812545 | 0.201043909 | 0.29448352 | 0.425138811 | 0.169129979 | 0.079353009 | 0.090937023 | 0.235032198 | 0.356065249 |
| MYL12B | Epi3 | turquoise | 0.284591131 | 0.154429622 | 0.268503997 | 0.510630893 | 0.158385608 | 0.051409752 | 0.207590836 | 0.178397138 | 0.410473401 |
| NDUFV2 | Epi3 | turquoise | 0.164751091 | 0.136847303 | 0.120017252 | 0.191455337 | 0.115939856 | 0.047577912 | 0.109278283 | 0.096646421 | 0.135886904 |
| VAPA | Epi3 | turquoise | 0.217555402 | 0.116090032 | 0.181339506 | 0.208790757 | 0.137990819 | 0.058735513 | 0.101387363 | 0.163631119 | 0.15635799 |
| CHMP1B | Epi3 | turquoise | 0.117702711 | 0.059963838 | 0.058684124 | 0.149637381 | 0.092500966 | 0.021947587 | 0.162893423 | -0.007685467 | 0.113455877 |
| PSMG2 | Epi3 | turquoise | 0.127645622 | 0.052793073 | 0.089951056 | 0.170760898 | 0.085132639 | -0.001639373 | 0.097341603 | 0.052922462 | 0.132875651 |
| INO80C | Epi3 | turquoise | 0.065086318 | 0.056911997 | 0.060568571 | 0.056102165 | 0.026442367 | 0.03443641 | -0.005906822 | 0.079425921 | 0.024434243 |
| ATP5A1 | Epi3 | turquoise | 0.117571289 | 0.063800131 | 0.042799707 | 0.216685243 | 0.059734989 | 0.030393485 | 0.155216657 | 0.033640605 | 0.122999895 |
| C18orf32 | Epi3 | turquoise | 0.127536843 | 0.114755797 | 0.105990481 | 0.153793971 | 0.061073765 | 0.043101187 | 0.017402609 | 0.124120941 | 0.084137294 |
| ACAA2 | Epi3 | turquoise | 0.080237437 | 0.033051628 | 0.052889509 | 0.181633462 | 0.040997524 | 0.023909489 | 0.098591941 | 0.047928729 | 0.110746516 |
| NARS | Epi3 | turquoise | 0.097661501 | 0.068369433 | 0.056697643 | 0.124278557 | 0.027842234 | 0.037088722 | 0.038910283 | 0.061911867 | 0.067527235 |
| SEC11C | Epi3 | turquoise | 0.130570715 | 0.112387226 | 0.066056884 | 0.187738799 | 0.061358822 | 0.029729576 | 0.105761313 | 0.051174694 | 0.091418826 |
| LMAN1 | Epi3 | turquoise | 0.103315939 | 0.049548892 | 0.079439775 | 0.141589454 | 0.054138699 | 0.024598298 | 0.090964439 | 0.053500254 | 0.122528358 |
| CNDP2 | Epi3 | turquoise | 0.097063331 | 0.045770929 | 0.060896576 | 0.123552002 | 0.033586281 | 0.022818778 | 0.082170017 | 0.049205441 | 0.088508713 |
| PSMF1 | Epi3 | turquoise | 0.086338114 | 0.06446361 | 0.071893579 | 0.083376121 | 0.065966381 | 0.023276464 | 0.021556002 | 0.041295764 | 0.078524761 |
| NSFL1C | Epi3 | turquoise | 0.061715541 | 0.049996365 | 0.029210992 | 0.083638988 | 0.029315813 | 0.027428497 | 0.055370883 | 0.033487821 | 0.041711367 |
| SNRPB | Epi3 | turquoise | 0.085890329 | 0.083235607 | 0.045379522 | 0.141302007 | 0.063405001 | -0.007394282 | 0.062535796 | 0.031304235 | 0.083355297 |
| NOP56 | Epi3 | turquoise | 0.071042107 | 0.048457496 | 0.021886851 | 0.112725322 | 0.017760471 | 0.061765828 | 0.091224303 | 0.024017169 | 0.062791812 |
| IDH3B | Epi3 | turquoise | 0.061116032 | 0.066023026 | 0.021302879 | 0.158060814 | 0.000634411 | 0.018923021 | 0.06521136 | 0.055606245 | 0.077095023 |
| MRPS26 | Epi3 | turquoise | 0.083598842 | 0.052384779 | 0.027805781 | 0.142072129 | 0.052725939 | -0.007158686 | 0.10531313 | 0.019332134 | 0.087459088 |
| DDRGK1 | Epi3 | turquoise | 0.09739014 | 0.107306224 | 0.068079843 | 0.159593665 | 0.039889229 | 0.025469414 | 0.036287818 | 0.07147484 | 0.092964573 |
| ITPA | Epi3 | turquoise | 0.114387916 | 0.070733051 | 0.071936413 | 0.148864932 | 0.063422367 | 0.024808863 | 0.090676019 | 0.051288585 | 0.104048138 |
| CDC25B | Epi3 | turquoise | 0.062448936 | 0.080846008 | 0.038999851 | 0.057796806 | 0.025917033 | 0.012907926 | -0.006673153 | 0.06667427 | 0.021453041 |
| PRNP | Epi3 | turquoise | 0.114242963 | 0.098897341 | 0.086603691 | 0.176076598 | 0.052829894 | 0.059962528 | 0.079463962 | 0.077577853 | 0.113436952 |
| TMEM230 | Epi3 | turquoise | 0.180146329 | 0.074934128 | 0.150671404 | 0.190529951 | 0.113999491 | 0.049125093 | 0.13179121 | 0.112228997 | 0.156795596 |
| PCNA | Epi3 | turquoise | 0.070845882 | 0.084652461 | 0.022621758 | 0.116884725 | -0.002507741 | 0.002409205 | 0.03641793 | 0.055394277 | 0.049509738 |
| SNRPB2 | Epi3 | turquoise | 0.133669703 | 0.104111386 | 0.110994657 | 0.262880571 | 0.040809644 | 0.031565679 | 0.082501052 | 0.120297682 | 0.154459059 |
| PCSK2 | Epi3 | turquoise | 0.135915363 | 0.116873419 | 0.151277715 | 0.182267213 | 0.084186045 | 0.03929449 | 0.002303837 | 0.152223085 | 0.145787692 |
| SNX5 | Epi3 | turquoise | 0.13567443 | 0.056276299 | 0.127900166 | 0.189801156 | 0.085166684 | 0.043073486 | 0.076130862 | 0.105163255 | 0.142560954 |
| SEC23B | Epi3 | turquoise | 0.050759583 | 0.074904855 | 0.02651408 | 0.076866503 | 0.008138946 | 0.000665424 | 0.037633702 | 0.039517407 | 0.013303871 |
| LINC00493 | Epi3 | turquoise | 0.146458736 | 0.089269785 | 0.123253264 | 0.168539345 | 0.115492198 | 0.048941537 | 0.076131914 | 0.091141913 | 0.123795001 |
| NAA20 | Epi3 | turquoise | 0.11608557 | 0.082039448 | 0.068422203 | 0.20815909 | 0.041170221 | 0.039470216 | 0.088089403 | 0.084748726 | 0.11253597 |
| XRN2 | Epi3 | turquoise | 0.097194554 | 0.03489182 | 0.051321713 | 0.141665857 | 0.040954869 | 0.046815797 | 0.11450828 | 0.021724395 | 0.10273426 |
| CST3 | Epi3 | turquoise | 0.267856384 | 0.325752404 | 0.158997894 | 0.463389441 | 0.100711444 | 0.065294871 | 0.155532288 | 0.204115501 | 0.242032896 |
| HM13 | Epi3 | turquoise | 0.104904447 | 0.089617325 | 0.071163006 | 0.231080123 | 0.024953811 | 0.008393197 | 0.100107036 | 0.073680753 | 0.136751487 |
| PDRG1 | Epi3 | turquoise | 0.030250049 | 0.031878658 | 0.001647204 | 0.074689227 | -0.013119447 | -0.017156439 | 0.036698108 | 0.024163865 | 0.020667229 |
| DYNLRB1 | Epi3 | turquoise | 0.196594749 | 0.118695766 | 0.182382085 | 0.222180352 | 0.122209486 | 0.043874437 | 0.075901253 | 0.15120004 | 0.162721028 |
| GSS | Epi3 | turquoise | 0.05363221 | 0.072014864 | 0.044825544 | 0.129977143 | 0.01361227 | 0.015871063 | 0.037661933 | 0.084056587 | 0.040758363 |
| EIF6 | Epi3 | turquoise | 0.105368862 | 0.08498956 | 0.059966348 | 0.251159923 | 0.040538426 | 0.026656001 | 0.098866064 | 0.06246473 | 0.144358154 |
| ERGIC3 | Epi3 | turquoise | 0.161157424 | 0.065374619 | 0.148710509 | 0.193897997 | 0.165576956 | -0.002920317 | 0.110212042 | 0.070275743 | 0.181609056 |
| CPNE1 | Epi3 | turquoise | 0.058032928 | 0.009428989 | 0.014564349 | 0.096131628 | 0.036175606 | 0.032605411 | 0.076947976 | 0.012554312 | 0.053979295 |
| SCAND1 | Epi3 | turquoise | 0.136452424 | 0.099104235 | 0.127585949 | 0.162522578 | 0.10183865 | 0.026797148 | 0.063562985 | 0.11858782 | 0.116589664 |
| RPN2 | Epi3 | turquoise | 0.123744845 | 0.071558363 | 0.094483103 | 0.217303465 | 0.051645551 | 0.040048079 | 0.078767602 | 0.067582709 | 0.165902446 |
| MANBAL | Epi3 | turquoise | 0.117645769 | 0.110829676 | 0.089021523 | 0.144974647 | 0.032932252 | 0.026189293 | 0.043991127 | 0.11680092 | 0.065581574 |
| PIGT | Epi3 | turquoise | 0.075495028 | 0.08611806 | 0.030347866 | 0.189658948 | -0.01440684 | 0.001852769 | 0.072808391 | 0.062875587 | 0.091476597 |
| DNTTIP1 | Epi3 | turquoise | 0.071516481 | 0.064026936 | 0.053867377 | 0.125942532 | 0.03477069 | 0.004956149 | 0.014082567 | 0.060033232 | 0.0711307 |
| CTSA | Epi3 | turquoise | 0.097795503 | 0.162575484 | 0.065473044 | 0.205366879 | -0.004720125 | 0.014137276 | 0.041771363 | 0.117210851 | 0.085786077 |
| CD40 | Epi3 | turquoise | 0.097828206 | 0.134412203 | 0.066083645 | 0.151383083 | 0.021902864 | 0.025788945 | 0.003141772 | 0.106849147 | 0.065418299 |
| DPM1 | Epi3 | turquoise | 0.079531825 | 0.090117072 | 0.046965436 | 0.23884673 | 0.01797004 | 0.036677536 | 0.078259993 | 0.064957873 | 0.136064738 |
| RTFDC1 | Epi3 | turquoise | 0.08401338 | 0.039700374 | 0.060445574 | 0.149269747 | 0.051952932 | 0.023732909 | 0.042641262 | 0.069151076 | 0.093699506 |
| PSMA7 | Epi3 | turquoise | 0.169755982 | 0.140086224 | 0.148205818 | 0.232651661 | 0.119805419 | 0.036943897 | 0.069501484 | 0.11772378 | 0.166169767 |
| ADRM1 | Epi3 | turquoise | 0.093403934 | 0.09397094 | 0.064848026 | 0.166929962 | 0.053028245 | 0.018999772 | 0.066625193 | 0.068372319 | 0.084445814 |
| STMN3 | Epi3 | turquoise | 0.063411218 | 0.089897207 | 0.0016577 | 0.083479477 | 0.008001754 | 0.023624508 | 0.050153291 | 0.013173982 | 0.024252072 |
| PLPP2 | Epi3 | turquoise | 0.072645676 | 0.127596313 | 0.024503303 | 0.092008503 | 0.051627562 | 0.017463825 | 0.003070138 | 0.044862668 | 0.032899374 |
| TPGS1 | Epi3 | turquoise | 0.073565257 | 0.070269156 | 0.054227553 | 0.092476343 | 0.038533177 | 0.016540604 | 0.030507741 | 0.068028262 | 0.035133435 |
| BSG | Epi3 | turquoise | 0.141573073 | 0.101009614 | 0.087040203 | 0.32745044 | 0.037362974 | 0.040113952 | 0.144973852 | 0.073810338 | 0.211867144 |
| WDR18 | Epi3 | turquoise | 0.039563101 | 0.015569891 | 0.025335421 | 0.09527017 | 0.041391169 | -0.014047126 | 0.035930504 | 0.012458164 | 0.059725686 |
| ATP5D | Epi3 | turquoise | 0.216473371 | 0.129766509 | 0.182622251 | 0.245643119 | 0.172907142 | 0.081293735 | 0.112737576 | 0.141533021 | 0.16625565 |
| NDUFS7 | Epi3 | turquoise | 0.086521152 | 0.072963679 | 0.058415226 | 0.190693619 | 0.042098509 | 0.02060309 | 0.083000069 | 0.076185255 | 0.119583205 |
| GAMT | Epi3 | turquoise | 0.034295704 | 0.042244698 | -0.027237772 | 0.068904422 | 0.006502871 | 0.006968388 | 0.071008913 | 0.00036725 | 0.00953512 |
| SF3A2 | Epi3 | turquoise | 0.05396365 | 0.043657214 | 0.025180471 | 0.09178441 | 0.04147067 | 0.02172405 | 0.04621172 | 0.020758895 | 0.042721551 |
| OAZ1 | Epi3 | turquoise | 0.259654529 | 0.171860566 | 0.251522629 | 0.331599356 | 0.195487523 | 0.070179734 | 0.113015379 | 0.196481957 | 0.259971337 |
| SLC39A3 | Epi3 | turquoise | 0.083063934 | 0.053409186 | 0.065345225 | 0.055736944 | 0.058053837 | 0.034868523 | 0.011882532 | 0.070929802 | 0.03132971 |
| STAP2 | Epi3 | turquoise | 0.042533565 | 0.034920279 | 0.002380174 | 0.078602581 | 0.028566818 | -0.003929686 | 0.052069838 | 0.001924719 | 0.064416855 |
| MYDGF | Epi3 | turquoise | 0.14104985 | 0.119877081 | 0.123161003 | 0.20319903 | 0.060879031 | 0.059826125 | 0.077130139 | 0.118992052 | 0.13014482 |
| PLIN3 | Epi3 | turquoise | 0.100610914 | 0.094708747 | 0.082309918 | 0.160339761 | 0.035182581 | 0.026237466 | 0.057724041 | 0.087097526 | 0.102566047 |
| C19orf70 | Epi3 | turquoise | 0.160487845 | 0.134825576 | 0.130991245 | 0.194679312 | 0.076180252 | 0.052493987 | 0.066966979 | 0.124743069 | 0.124574289 |
| NDUFA11 | Epi3 | turquoise | 0.224184149 | 0.079190277 | 0.220338908 | 0.230496276 | 0.185839699 | 0.065438735 | 0.112116252 | 0.130749648 | 0.223147757 |
| CAPS | Epi3 | turquoise | 0.05931327 | 0.124194237 | 0.009860779 | 0.1222807 | 0.000747347 | 0.034016714 | 0.039113968 | 0.072137668 | 0.02083255 |
| CLPP | Epi3 | turquoise | 0.105515099 | 0.078538245 | 0.079893263 | 0.123340464 | 0.06281409 | 0.028664233 | 0.04853385 | 0.061397887 | 0.083856878 |
| GPR108 | Epi3 | turquoise | 0.106849318 | 0.069337111 | 0.07412967 | 0.128155499 | 0.037511222 | 0.030663672 | 0.029089468 | 0.083090153 | 0.065156275 |
| XAB2 | Epi3 | turquoise | 0.058591697 | 0.053044695 | 0.027185359 | 0.106988817 | 0.021159135 | 0.013941706 | 0.040350264 | 0.056936237 | 0.040985133 |
| PET100 | Epi3 | turquoise | 0.174947205 | 0.096621075 | 0.154562099 | 0.229292931 | 0.126743336 | 0.078467474 | 0.13412582 | 0.088739766 | 0.187262059 |
| STXBP2 | Epi3 | turquoise | 0.067136168 | 0.07542281 | 0.030900176 | 0.131359066 | 0.021640517 | 0.009139144 | 0.049227701 | 0.049572562 | 0.060809267 |
| TIMM44 | Epi3 | turquoise | 0.066744531 | 0.029192958 | 0.048227172 | 0.109494908 | 0.03944408 | 0.014861385 | 0.072591915 | 0.023161738 | 0.099171196 |
| CERS4 | Epi3 | turquoise | 0.072713775 | -0.014931932 | 0.067180685 | 0.110387268 | 0.070965573 | 0.023039958 | 0.043572078 | 0.03917012 | 0.110651977 |
| NDUFA7 | Epi3 | turquoise | 0.12318821 | 0.066227081 | 0.096369692 | 0.141282983 | 0.096177811 | 0.02593277 | 0.065499013 | 0.05624687 | 0.108695388 |
| RAB11B | Epi3 | turquoise | 0.165996501 | 0.090104032 | 0.100844986 | 0.210634675 | 0.103890881 | 0.028300795 | 0.144650944 | 0.048761931 | 0.150877232 |
| HNRNPM | Epi3 | turquoise | 0.086007601 | 0.059193678 | 0.016248658 | 0.128706696 | 0.021746072 | 0.018155698 | 0.097519257 | 0.035790125 | 0.069255968 |
| CDC37 | Epi3 | turquoise | 0.067486179 | 0.055541575 | 0.039411786 | 0.119131169 | 0.024644364 | 0.022818428 | 0.057844309 | 0.040444664 | 0.074404494 |
| AP1M2 | Epi3 | turquoise | 0.065244469 | 0.040534931 | 0.009681784 | 0.138976065 | 0.062597155 | -0.017170822 | 0.135974503 | -0.045924964 | 0.110393175 |
| TMED1 | Epi3 | turquoise | 0.073699595 | 0.06598359 | 0.074762601 | 0.139819771 | 0.032332615 | 0.017505039 | 0.017090084 | 0.078859789 | 0.094271018 |
| TMEM205 | Epi3 | turquoise | 0.123709481 | 0.106866852 | 0.073002203 | 0.207478423 | 0.068009856 | 0.034730984 | 0.119120047 | 0.069877428 | 0.12898721 |
| WDR83OS | Epi3 | turquoise | 0.203317242 | 0.081762919 | 0.21089829 | 0.237215972 | 0.175422158 | 0.066141835 | 0.116918643 | 0.142610418 | 0.220407896 |
| DHPS | Epi3 | turquoise | 0.083653739 | 0.03891438 | 0.05567364 | 0.127300559 | 0.0609307 | 0.019359259 | 0.07577259 | 0.052060026 | 0.092304606 |
| ASNA1 | Epi3 | turquoise | 0.112060418 | 0.083260244 | 0.075189079 | 0.18580962 | 0.061619425 | 0.020977848 | 0.094341952 | 0.059823907 | 0.125648111 |
| PRDX2 | Epi3 | turquoise | 0.204250251 | 0.169508813 | 0.149421889 | 0.372917466 | 0.082427088 | 0.046280053 | 0.144195699 | 0.180967336 | 0.207745543 |
| DNASE2 | Epi3 | turquoise | 0.094044414 | 0.05366074 | 0.043444316 | 0.151413988 | 0.009849033 | 0.010879373 | 0.077710063 | 0.056144793 | 0.06750877 |
| FARSA | Epi3 | turquoise | 0.061923015 | 0.049398089 | 0.02190512 | 0.120673809 | 0.028532319 | 0.011754194 | 0.035475538 | 0.035571493 | 0.07506227 |
| CALR | Epi3 | turquoise | 0.135538773 | 0.106050014 | 0.015546722 | 0.253202372 | 0.00630615 | 0.092793377 | 0.232239256 | -0.022895406 | 0.12575231 |
| GADD45GIP1 | Epi3 | turquoise | 0.13383715 | 0.067840546 | 0.124362655 | 0.179402709 | 0.112738944 | 0.027495691 | 0.072778257 | 0.099495628 | 0.147156858 |
| TECR | Epi3 | turquoise | 0.070476207 | 0.072325644 | 0.023098066 | 0.245954112 | 0.005191454 | 0.003301167 | 0.097380189 | 0.05081761 | 0.111104931 |
| NDUFB7 | Epi3 | turquoise | 0.202259074 | 0.133948566 | 0.20318845 | 0.198280778 | 0.129023835 | 0.048275643 | 0.065583105 | 0.163800114 | 0.153725348 |
| ILVBL | Epi3 | turquoise | 0.077725889 | 0.077312603 | 0.034990827 | 0.153537544 | -0.002911679 | 0.051280243 | 0.057278922 | 0.065536045 | 0.076114824 |
| SMIM7 | Epi3 | turquoise | 0.104543438 | 0.039751577 | 0.083227128 | 0.124211102 | 0.071683713 | 0.019434668 | 0.066859459 | 0.060260549 | 0.089652324 |
| OCEL1 | Epi3 | turquoise | 0.060023053 | 0.011438652 | 0.0502949 | 0.091981969 | 0.053081924 | 0.021485402 | 0.051535281 | 0.03429055 | 0.079273589 |
| MVB12A | Epi3 | turquoise | 0.076077637 | 0.094226521 | 0.0339203 | 0.13755065 | 0.045405433 | 0.026782527 | 0.054646339 | 0.055371785 | 0.064724684 |
| ISYNA1 | Epi3 | turquoise | 0.088864799 | 0.11954289 | 0.04326025 | 0.140069455 | 0.006019242 | 0.010460817 | 0.012242949 | 0.098914601 | 0.043157942 |
| COPE | Epi3 | turquoise | 0.159120571 | 0.095609432 | 0.138928206 | 0.310932362 | 0.070586011 | 0.04196065 | 0.114235592 | 0.123565686 | 0.212837591 |
| RFXANK | Epi3 | turquoise | 0.096382462 | 0.043613364 | 0.087859218 | 0.128877752 | 0.059339733 | 0.012998802 | 0.053612028 | 0.078422411 | 0.107549484 |
| NDUFA13 | Epi3 | turquoise | 0.264217114 | 0.179179061 | 0.239162507 | 0.329248618 | 0.153575493 | 0.077583758 | 0.125238455 | 0.2217988 | 0.220670433 |
| UQCRFS1 | Epi3 | turquoise | 0.131269239 | 0.142810934 | 0.060631825 | 0.153957332 | 0.053120181 | 0.041085843 | 0.104389741 | 0.066664535 | 0.063964576 |
| POP4 | Epi3 | turquoise | 0.077283217 | 0.047093769 | 0.070405608 | 0.145899044 | 0.042046762 | 0.009650723 | 0.063515259 | 0.04454249 | 0.10600736 |
| PEPD | Epi3 | turquoise | 0.038703631 | 0.047208923 | 0.001085313 | 0.141558761 | 0.009170139 | 0.025079282 | 0.06994066 | 0.006787758 | 0.080434261 |
| LSR | Epi3 | turquoise | 0.110785205 | 0.116614524 | 0.083295194 | 0.146373111 | 0.054604549 | 0.027156383 | 0.037461083 | 0.068336305 | 0.094852858 |
| TMEM147 | Epi3 | turquoise | 0.161126928 | 0.087474403 | 0.122140135 | 0.169431907 | 0.102354292 | 0.025362902 | 0.122287353 | 0.090981896 | 0.111100276 |
| RBM42 | Epi3 | turquoise | 0.03419892 | 0.048924856 | -0.008771017 | 0.142095343 | -0.005452248 | -0.010803992 | 0.052186656 | 0.022805985 | 0.053402699 |
| PSENEN | Epi3 | turquoise | 0.110355818 | 0.092695479 | 0.100663819 | 0.145767401 | 0.061657812 | 0.036331712 | 0.050890987 | 0.097405347 | 0.094885175 |
| TBCB | Epi3 | turquoise | 0.112825412 | 0.116368237 | 0.096162605 | 0.179488997 | 0.031133316 | 0.054823762 | 0.031114269 | 0.113467156 | 0.104313503 |
| PSMD8 | Epi3 | turquoise | 0.122598932 | 0.106038651 | 0.096730087 | 0.259799975 | 0.057714306 | 0.027204108 | 0.092704134 | 0.091049188 | 0.169858115 |
| ECH1 | Epi3 | turquoise | 0.088833396 | 0.066413836 | 0.058945456 | 0.274190572 | 0.040308309 | 0.01315855 | 0.110271135 | 0.060754743 | 0.197859745 |
| MED29 | Epi3 | turquoise | 0.070565781 | 0.06629142 | 0.055409327 | 0.095016476 | 0.028455482 | 0.003794691 | 0.006310052 | 0.058721556 | 0.050678518 |
| TIMM50 | Epi3 | turquoise | 0.046186472 | 0.032233985 | 0.014135334 | 0.089197803 | 0.041727898 | -0.005531623 | 0.061647517 | 0.007769682 | 0.046221548 |
| PSMC4 | Epi3 | turquoise | 0.038241113 | 0.051945265 | 0.020980446 | 0.177199284 | -0.026302048 | 0.020628699 | 0.067509139 | 0.038701414 | 0.086267156 |
| PLD3 | Epi3 | turquoise | 0.1676833 | 0.130767349 | 0.166597327 | 0.290015963 | 0.043713653 | 0.060443898 | 0.034543638 | 0.234257945 | 0.182536662 |
| BLVRB | Epi3 | turquoise | 0.085427775 | 0.060892568 | 0.065089495 | 0.173190583 | 0.037554745 | 0.024066231 | 0.066845612 | 0.078400675 | 0.103094975 |
| RABAC1 | Epi3 | turquoise | 0.115233905 | 0.072800046 | 0.046550866 | 0.184987767 | 0.060946201 | 0.031937534 | 0.171732251 | 0.031988697 | 0.114527247 |
| CLPTM1 | Epi3 | turquoise | 0.045506771 | 0.041746805 | 0.040913383 | 0.090206976 | 0.018859249 | 0.04236257 | 0.037807362 | 0.038963853 | 0.055058681 |
| TRAPPC6A | Epi3 | turquoise | 0.092733845 | 0.008442729 | 0.048112751 | 0.121522617 | 0.063912453 | 0.035369539 | 0.106170676 | 0.011971563 | 0.088114699 |
| SNRPD2 | Epi3 | turquoise | 0.203680639 | 0.091656269 | 0.191427687 | 0.22863073 | 0.223253388 | 0.074351893 | 0.092707185 | 0.136207313 | 0.194564685 |
| PPP5C | Epi3 | turquoise | 0.041523949 | 0.038727746 | 0.024195634 | 0.068022018 | 0.007406532 | 0.007328797 | -0.01115746 | 0.051775245 | 0.020639756 |
| AP2S1 | Epi3 | turquoise | 0.162231287 | 0.161748546 | 0.138974459 | 0.198666828 | 0.077282275 | 0.031633221 | 0.083439939 | 0.13925127 | 0.126792605 |
| SAE1 | Epi3 | turquoise | 0.068933932 | 0.022849781 | 0.041989509 | 0.09374662 | 0.036043911 | 0.00468865 | 0.090706532 | 0.008308438 | 0.061479184 |
| NAPA | Epi3 | turquoise | 0.066006268 | 0.035692601 | 0.039427848 | 0.151669483 | 0.032365438 | 0.012649759 | 0.079038542 | 0.033603686 | 0.104690509 |
| KDELR1 | Epi3 | turquoise | 0.121864608 | 0.057072383 | 0.078892809 | 0.140215731 | 0.13456757 | 0.032961596 | 0.08458758 | 0.042943036 | 0.126886271 |
| NUCB1 | Epi3 | turquoise | 0.095711925 | 0.088957815 | 0.029644927 | 0.157777444 | 0.02243373 | 0.016970649 | 0.079830889 | 0.035966092 | 0.082618597 |
| RUVBL2 | Epi3 | turquoise | 0.04600823 | 0.041241337 | 0.011872105 | 0.120279147 | 0.012028826 | -0.010809356 | 0.056109403 | 0.018576163 | 0.050211243 |
| FCGRT | Epi3 | turquoise | 0.112933081 | 0.08100231 | 0.038894477 | 0.205347538 | 0.055862701 | 0.033343157 | 0.149478451 | 0.015712385 | 0.135447597 |
| NOSIP | Epi3 | turquoise | 0.08298944 | 0.049215781 | 0.055571713 | 0.130287056 | 0.049718372 | 0.013407653 | 0.065713618 | 0.063195106 | 0.079847935 |
| PRRG2 | Epi3 | turquoise | 0.069913735 | 0.077731511 | 0.03242971 | 0.103403247 | 0.033751362 | 0.006741724 | 0.034040608 | 0.032540061 | 0.059531795 |
| PRMT1 | Epi3 | turquoise | 0.063441146 | 0.053504107 | 0.03924922 | 0.200099413 | -0.005051549 | -0.005318501 | 0.084889595 | 0.042500833 | 0.095308874 |
| PTOV1 | Epi3 | turquoise | 0.09658032 | 0.041784931 | 0.039005378 | 0.1259533 | 0.071218302 | 0.041422569 | 0.119377365 | 0.003011707 | 0.079768886 |
| CLEC11A | Epi3 | turquoise | 0.06273271 | 0.044853576 | 0.067689713 | 0.09191621 | 0.001145412 | 0.003829445 | -0.014457084 | 0.121113708 | 0.022864087 |
| ETFB | Epi3 | turquoise | 0.121889065 | 0.075584699 | 0.082430663 | 0.196925553 | 0.056221328 | 0.03199912 | 0.089312685 | 0.089503326 | 0.124127388 |
| PPP2R1A | Epi3 | turquoise | 0.089262019 | 0.048962508 | 0.031273358 | 0.183910844 | 0.04098236 | 0.032745292 | 0.09725865 | 0.028270496 | 0.110777523 |
| NDUFA3 | Epi3 | turquoise | 0.228637785 | 0.124335584 | 0.234082444 | 0.280597343 | 0.162797879 | 0.083160987 | 0.094615812 | 0.169332759 | 0.234642424 |
| LENG1 | Epi3 | turquoise | 0.065958145 | 0.019751542 | 0.026720898 | 0.101432403 | 0.029110311 | 0.003855631 | 0.038880013 | 0.032998057 | 0.041260194 |
| TMC4 | Epi3 | turquoise | 0.062804418 | 0.030900698 | 0.034611288 | 0.110351957 | 0.050726952 | -0.005947919 | 0.070675698 | -0.00626153 | 0.097331358 |
| TSEN34 | Epi3 | turquoise | 0.116924996 | 0.106487354 | 0.076641575 | 0.136683642 | 0.055791737 | 0.010751374 | 0.051374747 | 0.085221611 | 0.080231582 |
| TRAPPC2P1 | Epi3 | turquoise | 0.060461268 | 0.035103381 | 0.031775257 | 0.110157376 | 0.013299539 | -0.00437354 | 0.056301383 | 0.018330512 | 0.042560766 |
| CHMP2A | Epi3 | turquoise | 0.177867644 | 0.13442533 | 0.170385819 | 0.24548168 | 0.088114052 | 0.045520642 | 0.05179422 | 0.178308499 | 0.155259016 |
| ATP6V1E1 | Epi3 | turquoise | 0.090737903 | 0.072969967 | 0.069862571 | 0.151307771 | 0.044892857 | 0.023431226 | 0.064618175 | 0.066087051 | 0.086804851 |
| BID | Epi3 | turquoise | 0.05443311 | 0.056422579 | 0.04729054 | 0.07182481 | 0.039759132 | 0.013042721 | 0.021668853 | 0.066003873 | 0.037666617 |
| UFD1L | Epi3 | turquoise | 0.09754052 | 0.049609073 | 0.103490618 | 0.110007832 | 0.060227503 | 0.036839556 | 0.03001541 | 0.076514153 | 0.079640104 |
| COMT | Epi3 | turquoise | 0.127063974 | 0.136511783 | 0.092247862 | 0.203006439 | 0.044940562 | 0.042160409 | 0.040558351 | 0.119397625 | 0.110801475 |
| UBE2L3 | Epi3 | turquoise | 0.128121307 | 0.098016181 | 0.118624925 | 0.17438118 | 0.052212741 | 0.041618832 | 0.074242863 | 0.102406269 | 0.116634684 |
| SMARCB1 | Epi3 | turquoise | 0.113942065 | 0.115774369 | 0.089275552 | 0.118150969 | 0.059476962 | 0.025851199 | 0.02528659 | 0.098526429 | 0.06703732 |
| MIF | Epi3 | turquoise | 0.31832291 | 0.252189828 | 0.25341352 | 0.348027704 | 0.214077979 | 0.079202894 | 0.126471814 | 0.202123772 | 0.232846041 |
| DDT | Epi3 | turquoise | 0.172389793 | 0.066136597 | 0.17676126 | 0.181265733 | 0.159112236 | 0.034921658 | 0.089989179 | 0.109780446 | 0.173231514 |
| SNRPD3 | Epi3 | turquoise | 0.101911223 | 0.040726655 | 0.081507801 | 0.183923614 | 0.049754411 | 0.002407052 | 0.109292117 | 0.065692961 | 0.132466139 |
| XBP1 | Epi3 | turquoise | 0.155389209 | 0.125980339 | 0.048715973 | 0.20278545 | 0.064300405 | 0.032952172 | 0.161758548 | 0.026126115 | 0.099511517 |
| EWSR1 | Epi3 | turquoise | 0.07075943 | 0.053945159 | 0.051063382 | 0.106583181 | 0.018785031 | 0.012492638 | 0.043847474 | 0.071607607 | 0.051821717 |
| ZMAT5 | Epi3 | turquoise | 0.04290622 | 0.038337988 | 0.025535567 | 0.070999503 | 0.025002672 | 0.011436896 | 0.042708826 | 0.031368872 | 0.034146097 |
| UQCR10 | Epi3 | turquoise | 0.250854641 | 0.131260945 | 0.257437383 | 0.213094148 | 0.167541901 | 0.064116253 | 0.117555716 | 0.170476435 | 0.203103937 |
| GATSL3 | Epi3 | turquoise | 0.057361269 | 0.079831193 | 0.066863302 | 0.107711439 | 0.000307535 | 0.011320049 | -0.025523254 | 0.101406404 | 0.050645482 |
| SELM | Epi3 | turquoise | 0.159583682 | 0.148544892 | 0.185608929 | 0.243634468 | 0.008497787 | 0.058301095 | -0.003674857 | 0.299768174 | 0.094303933 |
| RTCB | Epi3 | turquoise | 0.040051566 | 0.055005176 | 0.011843223 | 0.121576475 | 0.007068455 | 0.004708682 | 0.047740053 | 0.030918583 | 0.050504241 |
| RBX1 | Epi3 | turquoise | 0.229545496 | 0.151921013 | 0.217842024 | 0.26847706 | 0.158691995 | 0.068097662 | 0.074410323 | 0.172020486 | 0.21435989 |
| ACO2 | Epi3 | turquoise | 0.040587003 | 0.017008728 | 0.038581808 | 0.097571675 | -0.011376501 | 0.011547907 | 0.06324236 | 0.030657818 | 0.062182417 |
| PMM1 | Epi3 | turquoise | 0.043517628 | 0.036011214 | 0.021638078 | 0.085314057 | 0.007490949 | 0.017483555 | 0.042028689 | 0.02703064 | 0.047291166 |
| XRCC6 | Epi3 | turquoise | 0.121507385 | 0.090384816 | 0.06563775 | 0.262130476 | 0.026603448 | 0.021776181 | 0.106831947 | 0.079553605 | 0.150495007 |
| SNU13 | Epi3 | turquoise | 0.199169579 | 0.11940798 | 0.184848225 | 0.24640787 | 0.135209082 | 0.040740576 | 0.087715838 | 0.138846592 | 0.184678748 |
| NDUFA6 | Epi3 | turquoise | 0.135961401 | 0.073734123 | 0.123616114 | 0.193126373 | 0.091344908 | 0.048251152 | 0.057727554 | 0.100213257 | 0.156194855 |
| CYB5R3 | Epi3 | turquoise | 0.071158237 | 0.035571372 | 0.029525455 | 0.115929122 | 0.044314332 | 0.029114098 | 0.062183008 | 0.014972104 | 0.072115549 |
| MCAT | Epi3 | turquoise | 0.017520624 | 0.028827769 | 0.003669332 | 0.061383859 | 0.000141767 | 0.006989829 | 0.058774762 | 0.00475631 | 0.02728249 |
| TSPO | Epi3 | turquoise | 0.174701344 | 0.096631272 | 0.20825397 | 0.265749309 | 0.118666056 | 0.023288388 | 0.022762758 | 0.198410223 | 0.230111822 |
| SAMM50 | Epi3 | turquoise | 0.035998639 | 0.011721154 | 0.005943435 | 0.094650935 | 0.017527774 | 0.008595934 | 0.076967797 | -0.015643654 | 0.057903555 |
| ATXN10 | Epi3 | turquoise | 0.083153731 | 0.044389899 | 0.064620581 | 0.150120058 | 0.035352024 | 0.010329351 | 0.068223916 | 0.05471206 | 0.100957013 |
| CRELD2 | Epi3 | turquoise | 0.089982861 | 0.029723529 | 0.055919173 | 0.106805629 | 0.048396089 | 0.026699964 | 0.07565758 | 0.014105732 | 0.082764082 |
| LMF2 | Epi3 | turquoise | 0.0498387 | 0.075168831 | 0.02448995 | 0.097060952 | -0.005424123 | 0.012769535 | 0.034323939 | 0.050895548 | 0.028135851 |
| CXADR | Epi3 | turquoise | 0.127879328 | 0.132167333 | 0.112467738 | 0.18982046 | 0.032446426 | 0.064533366 | 0.014901049 | 0.130516567 | 0.098181144 |
| ATP5J | Epi3 | turquoise | 0.245641789 | 0.104442642 | 0.244468663 | 0.235663248 | 0.1587654 | 0.041152248 | 0.114745087 | 0.167352205 | 0.221714379 |
| CCT8 | Epi3 | turquoise | 0.106697993 | 0.063793521 | 0.095360828 | 0.190057654 | 0.087996013 | 0.008414537 | 0.045392208 | 0.092998664 | 0.14287297 |
| SOD1 | Epi3 | turquoise | 0.249435221 | 0.137917748 | 0.221159151 | 0.319960798 | 0.130311751 | 0.057130501 | 0.119806212 | 0.215295857 | 0.2052896 |
| C21orf59 | Epi3 | turquoise | 0.084642739 | 0.071413921 | 0.045144267 | 0.121473894 | 0.036638943 | 0.011094409 | 0.05749771 | 0.048901084 | 0.065408672 |
| TMEM50B | Epi3 | turquoise | 0.157191671 | 0.062380904 | 0.155603887 | 0.190318998 | 0.072672203 | 0.053315639 | 0.080217407 | 0.107380401 | 0.169552269 |
| SON | Epi3 | turquoise | 0.17517455 | 0.105318322 | 0.124296394 | 0.161617989 | 0.098852333 | 0.078843984 | 0.050894253 | 0.096235432 | 0.119814187 |
| ATP5O | Epi3 | turquoise | 0.135733084 | 0.029294773 | 0.096548296 | 0.186543727 | 0.131404983 | 0.015336358 | 0.124195169 | 0.040690837 | 0.155903241 |
| CBR1 | Epi3 | turquoise | 0.092233123 | 0.105888917 | 0.037572411 | 0.141826455 | 0.001355416 | 0.031572959 | 0.067466295 | 0.082999121 | 0.056245668 |
| PIGP | Epi3 | turquoise | 0.090511145 | 0.047555275 | 0.069724029 | 0.127846853 | 0.05357562 | 0.026411154 | 0.057983259 | 0.091111443 | 0.083640464 |
| PSMG1 | Epi3 | turquoise | 0.096246585 | 0.033061115 | 0.060993715 | 0.130819932 | 0.069160242 | 0.028007174 | 0.086541111 | 0.017522164 | 0.110049869 |
| NDUFV3 | Epi3 | turquoise | 0.108355358 | 0.058153668 | 0.074826227 | 0.119344324 | 0.061995007 | 0.049973597 | 0.075327333 | 0.066802218 | 0.073670509 |
| PFKL | Epi3 | turquoise | 0.045728279 | 0.006078181 | 0.042288681 | 0.086716173 | 0.033001157 | -0.007485787 | 0.026285534 | 0.020923013 | 0.094296495 |
| YBEY | Epi3 | turquoise | 0.0800979 | 0.048589058 | 0.066063755 | 0.106847 | 0.049963549 | 0.02209523 | 0.025526643 | 0.054606324 | 0.062247708 |
| PEX10 | Epi4 | blue | 0.045614356 | 0.000618559 | 0.048269138 | 0.032589565 | 0.069817005 | -0.007637879 | 0.036326752 | 0.024403582 | 0.05535607 |
| RPL22 | Epi4 | blue | 0.295567926 | -0.024988927 | 0.307399889 | 0.146751037 | 0.557670842 | 0.101091576 | 0.193302431 | 0.031109343 | 0.325298306 |
| VPS13D | Epi4 | blue | 0.050670612 | -0.029367793 | 0.070027927 | 0.016609497 | 0.078506898 | 0.005404817 | 0.006549376 | -0.005992183 | 0.073369347 |
| DHRS3 | Epi4 | blue | 0.101334488 | -0.15629186 | 0.185643166 | 0.042036 | 0.2616527 | 0.325005272 | 0.095129074 | -0.06903751 | 0.295638222 |
| RPL11 | Epi4 | blue | 0.401306141 | 0.036532309 | 0.388434787 | 0.191299005 | 0.697966155 | 0.126224849 | 0.252376541 | 0.059496877 | 0.388462415 |
| ARID1A | Epi4 | blue | 0.081511825 | 0.012585326 | 0.073085228 | 0.01574893 | 0.086066361 | 0.012806437 | 0.032330388 | 0.017721355 | 0.053254585 |
| YBX1 | Epi4 | blue | 0.238320835 | -0.028313228 | 0.18289108 | 0.11956362 | 0.330499499 | 0.085967938 | 0.209169172 | -0.03829164 | 0.248154706 |
| RPS8 | Epi4 | blue | 0.396972208 | 0.0871508 | 0.330495713 | 0.130165492 | 0.676937345 | 0.129841867 | 0.233490428 | 0.027803748 | 0.310819443 |
| EPS15 | Epi4 | blue | 0.057092925 | -0.000623653 | 0.07489699 | -0.013624922 | 0.0396648 | 0.016595393 | -0.002476388 | 0.028647823 | 0.031279592 |
| PLPP3 | Epi4 | blue | 0.194196454 | -0.033606704 | 0.206054914 | 0.112441619 | 0.245115805 | 0.05654656 | 0.12098126 | -0.005774842 | 0.258620214 |
| LMO4 | Epi4 | blue | 0.13463394 | 0.029152127 | 0.11361833 | 0.053293673 | 0.193870301 | 0.065421817 | 0.078900854 | -0.008970371 | 0.138318752 |
| RPL5 | Epi4 | blue | 0.315349644 | -0.058415523 | 0.278692972 | 0.1598969 | 0.596662411 | 0.105736213 | 0.25988559 | -0.013871852 | 0.351709489 |
| ARHGAP29 | Epi4 | blue | 0.067906244 | -0.051649752 | 0.050762236 | 0.023114188 | 0.08424206 | 0.045374739 | 0.098825165 | -0.043504808 | 0.099654266 |
| RPS27 | Epi4 | blue | 0.392852339 | 0.063318401 | 0.377591065 | 0.13702107 | 0.772218407 | 0.124394307 | 0.240010182 | -0.01482353 | 0.379625264 |
| SHE | Epi4 | blue | 0.036824598 | -0.045379876 | 0.073125854 | -0.001208588 | 0.088003393 | 0.011582258 | 0.036904867 | -0.003115165 | 0.070450101 |
| TSTD1 | Epi4 | blue | 0.23159426 | 0.067495808 | 0.226027702 | 0.239207706 | 0.296500163 | 0.079765341 | 0.141819119 | 0.059461 | 0.303733851 |
| PCNXL2 | Epi4 | blue | 0.065931279 | -0.016234058 | 0.088446373 | 0.018178675 | 0.087133269 | 0.024342235 | 0.046680547 | -0.014012435 | 0.087514609 |
| TOMM20 | Epi4 | blue | 0.130934801 | 0.033670172 | 0.10618622 | 0.089076655 | 0.176538745 | 0.02422422 | 0.085743123 | 0.037970272 | 0.111287214 |
| RPS7 | Epi4 | blue | 0.309833918 | 0.068244297 | 0.268033229 | 0.173098752 | 0.520333691 | 0.097259695 | 0.188166676 | 0.024389542 | 0.293330158 |
| PTRHD1 | Epi4 | blue | 0.148687457 | 0.00768331 | 0.139645585 | 0.103461301 | 0.200713232 | 0.035848631 | 0.11547766 | -0.002333888 | 0.180791182 |
| SPTBN1 | Epi4 | blue | 0.091822054 | 0.001073102 | 0.113550041 | 0.031234259 | 0.067427355 | 0.03984709 | 0.022274129 | 0.057717024 | 0.082605623 |
| RPS27A | Epi4 | blue | 0.417906934 | 0.061118035 | 0.399123327 | 0.210504086 | 0.672305542 | 0.140467899 | 0.243722538 | 0.08329958 | 0.376037585 |
| AAK1 | Epi4 | blue | 0.060009705 | -0.01585006 | 0.027541351 | 0.003884115 | 0.04782371 | -0.008812828 | 0.108241051 | -0.0544371 | 0.030970333 |
| MXD1 | Epi4 | blue | 0.033780156 | 0.009925522 | 0.020578383 | -0.015109451 | 0.006209852 | 0.018581153 | -0.008191507 | -0.008019199 | 3.91E-05 |
| SLC4A5 | Epi4 | blue | 0.027533969 | -0.074536782 | 0.084764347 | -0.006711662 | 0.050641989 | -0.029164336 | -0.001907766 | 0.011361822 | 0.063454443 |
| EVA1A | Epi4 | blue | 0.083138881 | -0.017273563 | 0.10721973 | 0.027021934 | 0.144597082 | -0.011679222 | 0.047862029 | -0.012392463 | 0.129702416 |
| TMSB10 | Epi4 | blue | 0.380913951 | 0.280122052 | 0.348857471 | 0.113051423 | 0.413447869 | 0.121358313 | 0.073073038 | 0.112214586 | 0.237182775 |
| RPL31 | Epi4 | blue | 0.351728057 | -0.03496096 | 0.382676168 | 0.185270625 | 0.661097272 | 0.10283202 | 0.229231989 | 0.046324312 | 0.421618144 |
| ST6GAL2 | Epi4 | blue | 0.138334512 | -0.04636048 | 0.180752613 | 0.056130507 | 0.176716946 | 0.053961599 | 0.049280771 | 0.035902414 | 0.166154035 |
| MALL | Epi4 | blue | 0.105025855 | 0.02872753 | 0.069697073 | 0.035108067 | 0.160136726 | 0.040431269 | 0.107937129 | -0.06846059 | 0.136960405 |
| MZT2B | Epi4 | blue | 0.148008988 | -0.075605827 | 0.169427127 | 0.069929383 | 0.202156326 | 0.043357667 | 0.145690973 | -0.00743841 | 0.182800954 |
| KLHL23 | Epi4 | blue | 0.078319208 | -0.03149733 | 0.112085875 | 0.014892629 | 0.094600572 | 0.048975792 | 0.016987574 | 0.037173764 | 0.086037608 |
| PDE1A | Epi4 | blue | 0.040877419 | -0.071513249 | 0.009222915 | 0.040857282 | 0.067600134 | -0.002445254 | 0.100745769 | -0.046230429 | 0.083233861 |
| GULP1 | Epi4 | blue | 0.075782007 | -0.082164511 | 0.136079843 | 0.033010809 | 0.15488043 | 0.033540776 | 0.058868984 | -0.023724422 | 0.170421343 |
| EEF1B2 | Epi4 | blue | 0.194160754 | 0.052915653 | 0.142719327 | 0.101883847 | 0.313093332 | 0.075144795 | 0.149977901 | -0.008087287 | 0.163899637 |
| FN1 | Epi4 | blue | 0.195000013 | 0.129989888 | 0.134542593 | 0.042313635 | 0.225860516 | 0.060468362 | 0.105381103 | -0.069091474 | 0.134450889 |
| RPL37A | Epi4 | blue | 0.40561059 | 0.017763827 | 0.437379809 | 0.145841289 | 0.714332373 | 0.1134654 | 0.225843728 | 0.027104255 | 0.431791509 |
| SERPINE2 | Epi4 | blue | 0.068130672 | -0.114657216 | 0.10889251 | 0.10707496 | 0.182762351 | 0.000346744 | 0.143468172 | -0.085150652 | 0.26920976 |
| RPL32 | Epi4 | blue | 0.378616764 | 0.049479905 | 0.330066364 | 0.169360447 | 0.71389457 | 0.116078246 | 0.265737833 | -0.014574521 | 0.366968045 |
| RPL15 | Epi4 | blue | 0.404121226 | 0.015630344 | 0.40532674 | 0.183186415 | 0.715103068 | 0.139892065 | 0.227712078 | 0.075590842 | 0.401207017 |
| SLC25A38 | Epi4 | blue | 0.047748595 | -0.025020984 | 0.042932144 | 0.033289761 | 0.08313649 | -0.01191008 | 0.048174315 | -0.035974237 | 0.079164146 |
| RPSA | Epi4 | blue | 0.326701927 | 0.153783227 | 0.267248655 | 0.200264874 | 0.477090822 | 0.127547828 | 0.165993916 | 0.092676964 | 0.242505751 |
| RPL14 | Epi4 | blue | 0.319837003 | 0.051998877 | 0.254151767 | 0.143061787 | 0.573237853 | 0.107026897 | 0.21630541 | -0.029131415 | 0.291175564 |
| TMA7 | Epi4 | blue | 0.297984494 | 0.054892891 | 0.38147376 | 0.131841904 | 0.374044439 | 0.0867397 | 0.090173108 | 0.142438938 | 0.271395065 |
| RPL29 | Epi4 | blue | 0.307804215 | 0.001609171 | 0.303355956 | 0.092802726 | 0.574807172 | 0.090951449 | 0.189919523 | 0.004698521 | 0.291893526 |
| FHIT | Epi4 | blue | 0.09107861 | -0.006166017 | 0.101605856 | 0.062716303 | 0.111596677 | 0.006399706 | 0.074341355 | 0.005199182 | 0.113964057 |
| ADAMTS9 | Epi4 | blue | 0.067646199 | -0.061656504 | 0.139418692 | 0.022082211 | 0.11308797 | 0.013124486 | 0.022798625 | 0.022935343 | 0.146077484 |
| RPL24 | Epi4 | blue | 0.391202123 | 0.146201344 | 0.361414241 | 0.203540657 | 0.596281649 | 0.109031314 | 0.178244128 | 0.117084547 | 0.314500536 |
| HEG1 | Epi4 | blue | 0.096562835 | 0.016241585 | 0.053682351 | 0.03544682 | 0.089775546 | 0.04021662 | 0.077884244 | -0.005165157 | 0.066301672 |
| CHST2 | Epi4 | blue | 0.065231279 | -0.031380197 | 0.10227033 | 0.00293462 | 0.121821603 | 0.047662812 | 0.019459849 | -0.008040905 | 0.102127169 |
| TIPARP | Epi4 | blue | 0.078032151 | 0.017142009 | 0.097847521 | 0.021562348 | 0.08577025 | 0.02951495 | -0.000222245 | 0.025019131 | 0.074943912 |
| GNB4 | Epi4 | blue | 0.071566146 | -0.022724536 | 0.087888227 | 0.040660163 | 0.111907027 | 0.044031708 | 0.07031214 | -0.019671347 | 0.120669228 |
| EIF4A2 | Epi4 | blue | 0.231873141 | 0.010079396 | 0.176943126 | 0.19383193 | 0.335716344 | 0.058703859 | 0.141418647 | 0.052283059 | 0.246328625 |
| CLDN16 | Epi4 | blue | 0.157620485 | -0.06889795 | 0.197679186 | 0.121615561 | 0.184157773 | 0.036705651 | 0.067492344 | 0.078536594 | 0.241626218 |
| RPL35A | Epi4 | blue | 0.369838444 | 0.036794377 | 0.337752144 | 0.181343671 | 0.657387471 | 0.096495721 | 0.264354999 | 0.019814371 | 0.355225566 |
| FAM200B | Epi4 | blue | 0.126243781 | 0.002782416 | 0.147122776 | 0.054352665 | 0.146700312 | 0.046876782 | 0.03769156 | 0.088627622 | 0.105774448 |
| RPL9 | Epi4 | blue | 0.379741762 | 0.036388594 | 0.357748483 | 0.177653188 | 0.630105597 | 0.112748599 | 0.223736971 | 0.058897586 | 0.35245579 |
| ATP8A1 | Epi4 | blue | 0.060588052 | 0.031533863 | 0.043057062 | 0.043109421 | 0.100972743 | -0.006349713 | 0.073193259 | -0.057175779 | 0.092682577 |
| UBA6-AS1 | Epi4 | blue | 0.0272317 | -0.032696251 | 0.043974919 | 0.010324906 | 0.064808122 | 0.022725261 | 0.022019386 | -0.018652985 | 0.069443963 |
| CCNI | Epi4 | blue | 0.28458376 | 0.047426534 | 0.218642508 | 0.071951209 | 0.350411057 | 0.097120414 | 0.12842664 | 0.082364023 | 0.140884065 |
| PPM1K | Epi4 | blue | 0.113080337 | -0.101312245 | 0.140709294 | 0.073654626 | 0.219191079 | 0.052569982 | 0.14268728 | -0.093775659 | 0.267822034 |
| NPNT | Epi4 | blue | 0.162473932 | -0.09367052 | 0.227641232 | 0.093203222 | 0.186018538 | 0.070866706 | 0.121582362 | 0.036972762 | 0.258377085 |
| RPL34 | Epi4 | blue | 0.433384389 | 0.013371855 | 0.441064079 | 0.141598396 | 0.754460591 | 0.128874569 | 0.231404202 | 0.046021655 | 0.400035036 |
| SNHG8 | Epi4 | blue | 0.176737621 | -0.048512073 | 0.134945799 | 0.085208628 | 0.348507639 | 0.029197914 | 0.192190476 | -0.0388603 | 0.199748501 |
| PDE5A | Epi4 | blue | 0.109389637 | -0.006385588 | 0.127939181 | 0.051898077 | 0.133659449 | 0.06646297 | 0.065599831 | 0.009670368 | 0.159692365 |
| JADE1 | Epi4 | blue | 0.069739523 | -0.030103787 | 0.056052055 | 0.022750391 | 0.081176488 | 0.033957902 | 0.066365665 | -0.013815927 | 0.07056933 |
| RPS3A | Epi4 | blue | 0.389524077 | -0.030062591 | 0.39001348 | 0.189729785 | 0.676839882 | 0.117713407 | 0.251445778 | 0.049608667 | 0.396199218 |
| MIR4458HG | Epi4 | blue | 0.092919918 | -0.062787067 | 0.11748694 | 0.050282953 | 0.135730896 | 0.039335779 | 0.067344221 | 0.026983193 | 0.125558571 |
| SNHG18 | Epi4 | blue | 0.034446758 | -0.056384802 | 0.021607742 | -7.88E-05 | 0.101862098 | 0.031560663 | 0.088542327 | -0.058747365 | 0.079402803 |
| BASP1 | Epi4 | blue | 0.08330621 | 0.000359009 | 0.095750952 | -0.004145362 | 0.106741833 | 0.022007449 | 0.01549953 | -0.001400228 | 0.08360028 |
| RPL37 | Epi4 | blue | 0.366392745 | 0.060880193 | 0.383172983 | 0.168549726 | 0.648884615 | 0.115693135 | 0.19242438 | 0.043449519 | 0.365135495 |
| BTF3 | Epi4 | blue | 0.233034797 | 0.014523894 | 0.186797624 | 0.201598093 | 0.35393153 | 0.076629611 | 0.208638979 | 0.02219243 | 0.277079924 |
| NSA2 | Epi4 | blue | 0.110199493 | 0.010072298 | 0.078308425 | 0.060074597 | 0.171160841 | 0.031101447 | 0.075264064 | 0.00549711 | 0.102052993 |
| PDE8B | Epi4 | blue | 0.178090578 | 0.020656347 | 0.218669201 | 0.068372917 | 0.161956541 | 0.051736816 | 0.039873633 | 0.095959962 | 0.172811019 |
| TBCA | Epi4 | blue | 0.214594274 | 0.031443362 | 0.204587168 | 0.180995928 | 0.232526319 | 0.042560007 | 0.120977485 | 0.090196428 | 0.230218182 |
| RPS23 | Epi4 | blue | 0.333055825 | -0.020317932 | 0.318383077 | 0.120506345 | 0.687210944 | 0.095001034 | 0.238159629 | -0.052251604 | 0.371573072 |
| COX7C | Epi4 | blue | 0.337784771 | 0.004261742 | 0.35373645 | 0.244389911 | 0.505228072 | 0.074676075 | 0.257590199 | 0.063779957 | 0.40093017 |
| TMEM161B-AS1 | Epi4 | blue | 0.049319775 | -0.039326693 | 0.066686945 | 0.033161545 | 0.080906335 | -0.004208941 | 0.050936047 | 0.01041325 | 0.073204213 |
| EPB41L4A-AS1 | Epi4 | blue | 0.068772295 | -0.038391044 | 0.049307795 | 0.020430321 | 0.123531771 | 0.012318834 | 0.04421009 | -0.011229662 | 0.057242665 |
| SLC27A6 | Epi4 | blue | 0.044924452 | -0.067489274 | 0.040215273 | 0.032504886 | 0.10318294 | -0.002081513 | 0.062980502 | -0.064951168 | 0.115103167 |
| RPS14 | Epi4 | blue | 0.414831022 | 0.084948904 | 0.38481164 | 0.17581548 | 0.712894447 | 0.123779757 | 0.25856918 | 0.051830853 | 0.353497219 |
| NPM1 | Epi4 | blue | 0.185653456 | -0.060642183 | 0.132715313 | 0.172799972 | 0.372358736 | 0.028483899 | 0.203881682 | -0.039468301 | 0.264543153 |
| GNB2L1 | Epi4 | blue | 0.302940074 | 0.020762437 | 0.240433154 | 0.143680479 | 0.559444369 | 0.094972746 | 0.249008175 | -0.061209617 | 0.319134882 |
| LYRM4 | Epi4 | blue | 0.084001737 | -0.003915341 | 0.084011022 | 0.011855144 | 0.147975948 | 0.029365975 | 0.064151394 | 0.005779146 | 0.074779074 |
| MCUR1 | Epi4 | blue | 0.092761335 | 0.017533729 | 0.090061864 | 0.024289091 | 0.111242319 | 0.057116369 | 0.052498793 | 0.011832878 | 0.067503643 |
| ID4 | Epi4 | blue | 0.102516803 | -0.071537547 | 0.033192112 | 0.094253801 | 0.077386956 | 0.006137758 | 0.257586442 | -0.121215709 | 0.133782193 |
| SOX4 | Epi4 | blue | 0.143359652 | 0.047763314 | 0.055295324 | 0.037220809 | 0.194964653 | 0.066545081 | 0.14959796 | -0.097176414 | 0.101356613 |
| HIST1H1C | Epi4 | blue | 0.028779906 | -0.0739565 | 0.012760097 | 0.047927315 | 0.095347888 | -0.02010554 | 0.10067536 | -0.083431748 | 0.096441006 |
| HIST1H2AE | Epi4 | blue | 0.013149477 | -0.056364553 | 0.037573727 | 0.005562925 | 0.067922605 | -0.009339696 | 0.022370163 | -0.007601925 | 0.050675921 |
| C6orf48 | Epi4 | blue | 0.166873294 | -0.122925488 | 0.148921858 | 0.057296546 | 0.3722421 | 0.032446555 | 0.200429358 | -0.085245765 | 0.253755549 |
| RPS18 | Epi4 | blue | 0.438897426 | 0.153687142 | 0.367196164 | 0.181675476 | 0.724197741 | 0.135046096 | 0.272355003 | 0.052667261 | 0.326846767 |
| HMGA1 | Epi4 | blue | 0.059572946 | 0.005144444 | 0.063874638 | 0.00732237 | 0.103916549 | 0.023470066 | 0.03805831 | 0.003185955 | 0.051009573 |
| RPS10 | Epi4 | blue | 0.332632511 | 0.082987438 | 0.289900403 | 0.209914289 | 0.488048756 | 0.107103655 | 0.193715499 | 0.062837966 | 0.289530441 |
| RPL10A | Epi4 | blue | 0.353886561 | 0.08892871 | 0.307232401 | 0.174872063 | 0.625718279 | 0.129327898 | 0.20278592 | 0.059338988 | 0.306715089 |
| VEGFA | Epi4 | blue | 0.075221336 | -0.083277391 | 0.139373738 | -0.021462406 | 0.093273768 | 0.035657783 | 0.014279436 | 0.031335337 | 0.105889826 |
| EEF1A1 | Epi4 | blue | 0.459209235 | 0.030426548 | 0.419667408 | 0.129656662 | 0.785909317 | 0.154613068 | 0.249700026 | 0.04247283 | 0.39054118 |
| FILIP1 | Epi4 | blue | 0.046066328 | -0.074670818 | 0.103434562 | 0.031860537 | 0.110166562 | -0.00565419 | 0.028373325 | -0.031190935 | 0.135749674 |
| HMGN3 | Epi4 | blue | 0.197019959 | -0.066941017 | 0.275679433 | 0.124512236 | 0.302514292 | 0.047802185 | 0.116794205 | 0.012241825 | 0.31157744 |
| LCA5 | Epi4 | blue | 0.031803011 | -0.006543387 | 0.025980467 | 0.009117529 | 0.08619181 | 0.028055511 | 0.051315111 | -0.020509953 | 0.060893341 |
| CD24 | Epi4 | blue | 0.21523527 | 0.003266834 | 0.097465412 | 0.174448318 | 0.243782657 | 0.088743124 | 0.282195813 | -0.087155446 | 0.243414469 |
| STX7 | Epi4 | blue | 0.069709339 | 0.004908354 | 0.059503528 | 0.033844369 | 0.082677621 | 0.016828084 | 0.032078636 | 3.90E-05 | 0.066596554 |
| RPS12 | Epi4 | blue | 0.397742993 | 0.073339773 | 0.380923428 | 0.137572153 | 0.739301693 | 0.143102826 | 0.248243072 | 0.013240376 | 0.380503085 |
| PHACTR2 | Epi4 | blue | 0.072341019 | -0.021008046 | 0.056083545 | 0.03342879 | 0.075305152 | 0.100314543 | 0.062150268 | -0.013535455 | 0.068014089 |
| PPP1R14C | Epi4 | blue | 0.079157711 | -0.043177053 | 0.029485776 | 0.051161218 | 0.125006732 | 0.040102625 | 0.147296238 | -0.075931789 | 0.107493542 |
| RPS6KA2 | Epi4 | blue | 0.062944674 | 0.007170737 | 0.065141975 | 0.016439535 | 0.041111203 | 0.026656216 | 0.019043079 | 0.020906951 | 0.03919678 |
| CDCA7L | Epi4 | blue | 0.033010335 | -0.081382219 | 0.061982944 | -0.002728106 | 0.104861937 | 0.042715416 | 0.056964043 | -0.041590825 | 0.090666844 |
| TOMM7 | Epi4 | blue | 0.286523279 | 0.078710335 | 0.337642731 | 0.157553089 | 0.398630327 | 0.090138363 | 0.074049816 | 0.182578126 | 0.225742274 |
| MEPCE | Epi4 | blue | 0.052044312 | -0.010157846 | 0.054019145 | 0.007757436 | 0.054233808 | 0.019365495 | 0.017784774 | 0.037629556 | 0.023543394 |
| CREB3L2 | Epi4 | blue | 0.08618717 | 0.003907858 | 0.052057121 | 0.036572176 | 0.07265527 | 0.045593551 | 0.091335899 | -0.026071968 | 0.080864944 |
| EPHB6 | Epi4 | blue | 0.029353138 | -0.044346893 | 0.008535138 | 0.0164516 | 0.042293958 | 0.017378781 | 0.067799165 | -0.031031677 | 0.042884479 |
| SLC25A6 | Epi4 | blue | 0.253936643 | 0.019045692 | 0.17981922 | 0.131232086 | 0.377312955 | 0.097216065 | 0.218739255 | -0.022976096 | 0.220649917 |
| TMSB4X | Epi4 | blue | 0.234945272 | -0.157913422 | 0.370464052 | 0.070312038 | 0.443952154 | 0.072822857 | 0.165816206 | -0.07525341 | 0.453063444 |
| SSX1 | Epi4 | blue | 0.047477393 | -0.059572948 | 0.056122944 | 0.011503802 | 0.133383507 | 0.031357521 | 0.091413661 | -0.057687023 | 0.121439234 |
| LINC01420 | Epi4 | blue | 0.184711703 | 0.008137143 | 0.213071332 | 0.113980429 | 0.192902809 | 0.025345433 | 0.103487713 | 0.096994424 | 0.180475849 |
| RPS4X | Epi4 | blue | 0.428659721 | 0.356345477 | 0.381584387 | 0.141903154 | 0.569170156 | 0.165842967 | 0.013815894 | 0.264177832 | 0.161187214 |
| RPL36A | Epi4 | blue | 0.259513873 | 0.068541899 | 0.17136534 | 0.162946651 | 0.407136334 | 0.07898657 | 0.201073597 | -0.013556284 | 0.208793344 |
| BEX5 | Epi4 | blue | 0.060663864 | -0.063700695 | 0.035244553 | 0.047984498 | 0.11689754 | -0.016303026 | 0.096577437 | -0.026725615 | 0.096442608 |
| UBE2A | Epi4 | blue | 0.170701552 | 0.04091589 | 0.19205366 | 0.10793091 | 0.164650211 | 0.073206049 | 0.031874363 | 0.091987076 | 0.16359609 |
| RPL39 | Epi4 | blue | 0.386654522 | -0.039731144 | 0.416153218 | 0.165812725 | 0.781320712 | 0.094132397 | 0.289371065 | -0.0241268 | 0.472267929 |
| MMGT1 | Epi4 | blue | 0.082245206 | 0.028832602 | 0.069938987 | 0.045625587 | 0.076764108 | 0.017003082 | 0.060542986 | 0.033879614 | 0.068221858 |
| RPL10 | Epi4 | blue | 0.384651621 | 0.084726312 | 0.315413441 | 0.147267069 | 0.745548728 | 0.134147421 | 0.256717329 | -0.032855912 | 0.354728109 |
| LPL | Epi4 | blue | 0.079535807 | -0.107756077 | 0.163086199 | 0.062721818 | 0.126481924 | 0.024736545 | 0.066455113 | 0.024833772 | 0.20233635 |
| PPP2CB | Epi4 | blue | 0.118560963 | 0.043002915 | 0.111896968 | 0.05055208 | 0.107929321 | 0.036662398 | 0.070618835 | 0.026389976 | 0.107735195 |
| UNC5D | Epi4 | blue | 0.036327171 | -0.01079527 | 0.065440663 | 0.007346942 | 0.067485768 | 0.023322949 | 0.023636869 | -0.017516165 | 0.077193099 |
| HOOK3 | Epi4 | blue | 0.05740454 | 0.009072656 | 0.01243963 | 0.027325794 | 0.048264962 | 0.049542759 | 0.080835675 | -0.038328389 | 0.053066092 |
| RPS20 | Epi4 | blue | 0.343852512 | 0.082708731 | 0.341749196 | 0.12979257 | 0.531874239 | 0.094166602 | 0.149181361 | 0.086423171 | 0.295112514 |
| RPL7 | Epi4 | blue | 0.332650062 | -0.056520967 | 0.258699225 | 0.154311432 | 0.60159938 | 0.111731829 | 0.28757756 | -0.04083745 | 0.321001285 |
| PDP1 | Epi4 | blue | 0.068330967 | 0.053241673 | 0.06888069 | 0.027108543 | 0.071830073 | -0.005350292 | 0.035867328 | 0.020584918 | 0.058472992 |
| RPL30 | Epi4 | blue | 0.327385927 | 0.006923268 | 0.321796063 | 0.168116858 | 0.610929579 | 0.105163784 | 0.241642382 | 0.018774279 | 0.346603212 |
| PABPC1 | Epi4 | blue | 0.21200062 | 0.062552991 | 0.117007749 | 0.033234903 | 0.309022004 | 0.078283644 | 0.124008668 | -0.038967206 | 0.093276153 |
| ZNF706 | Epi4 | blue | 0.184544284 | 0.057010369 | 0.186256363 | 0.109659669 | 0.173832664 | 0.053436625 | 0.042731687 | 0.110679703 | 0.134510871 |
| EIF3E | Epi4 | blue | 0.203837084 | -0.064288735 | 0.181084796 | 0.155900359 | 0.391913883 | 0.075595215 | 0.185952212 | -0.004019385 | 0.275169708 |
| EIF3H | Epi4 | blue | 0.128347777 | -0.00410771 | 0.105019682 | 0.080876184 | 0.231204592 | 0.028442859 | 0.112212758 | -0.012155123 | 0.154975723 |
| COL14A1 | Epi4 | blue | 0.062150559 | -0.066948691 | 0.05367612 | 0.035198817 | 0.099574021 | -0.009125412 | 0.071004138 | -0.039181021 | 0.118145419 |
| ZHX2 | Epi4 | blue | 0.021714509 | -0.03172792 | 0.041735669 | -0.00293468 | 0.051526399 | 0.025945578 | 0.018058905 | -0.018140021 | 0.052291466 |
| TRIB1 | Epi4 | blue | 0.090159981 | 0.000668149 | 0.040884589 | 0.021654075 | 0.130183054 | 0.017666761 | 0.135219107 | -0.093687397 | 0.088304999 |
| ST3GAL1 | Epi4 | blue | 0.106017082 | 0.034349126 | 0.081196646 | 0.034391018 | 0.088330871 | 0.07491018 | 0.0508967 | 0.019525879 | 0.057327504 |
| EEF1D | Epi4 | blue | 0.257615214 | 0.133242189 | 0.228937516 | 0.080268447 | 0.397126687 | 0.093066712 | 0.083063941 | 0.086331262 | 0.15155197 |
| RPL8 | Epi4 | blue | 0.378203128 | 0.133449252 | 0.297834388 | 0.156684688 | 0.602867243 | 0.110800011 | 0.229797095 | 0.030158567 | 0.283361656 |
| BNC2 | Epi4 | blue | 0.036283657 | -0.050818363 | 0.072840597 | -0.003514762 | 0.086998376 | 0.022126611 | 0.024391806 | -0.01954841 | 0.090107774 |
| RPS6 | Epi4 | blue | 0.404914478 | 0.0385983 | 0.366704674 | 0.179000631 | 0.727585339 | 0.145235429 | 0.270869161 | 0.009248135 | 0.391784641 |
| EPB41L4B | Epi4 | blue | 0.039256023 | -0.00103765 | 0.016752501 | -0.001101873 | 0.055045991 | -0.008481373 | 0.070327241 | -0.042357099 | 0.023929364 |
| MIR181A2HG | Epi4 | blue | 0.065862772 | -0.041606893 | 0.065109249 | 0.031996068 | 0.10993334 | 0.007547898 | 0.096808255 | -0.031739201 | 0.116587837 |
| RPL35 | Epi4 | blue | 0.370514597 | 0.104190862 | 0.364885715 | 0.179146585 | 0.59250125 | 0.104838491 | 0.16715026 | 0.111063774 | 0.305817779 |
| RPL12 | Epi4 | blue | 0.414168482 | 0.101545119 | 0.422443145 | 0.112027189 | 0.707194446 | 0.130677583 | 0.148959273 | 0.12558868 | 0.318031668 |
| FNBP1 | Epi4 | blue | 0.058454905 | -0.022158399 | 0.03366355 | 0.026640155 | 0.060060227 | 0.005684972 | 0.076556231 | -0.025456221 | 0.069914454 |
| RPL7A | Epi4 | blue | 0.350896965 | 0.057100814 | 0.308717105 | 0.143826575 | 0.618931416 | 0.119018844 | 0.217490085 | 0.020165111 | 0.303272703 |
| RPLP2 | Epi4 | blue | 0.404218249 | 0.108883527 | 0.419349306 | 0.15491296 | 0.693563447 | 0.130204163 | 0.195282698 | 0.062044473 | 0.369135384 |
| EIF3F | Epi4 | blue | 0.130963534 | -0.014136699 | 0.108543048 | 0.082628962 | 0.248096563 | 0.068796668 | 0.120230114 | -0.01561897 | 0.138412064 |
| RPL27A | Epi4 | blue | 0.382138748 | 0.008127024 | 0.38539462 | 0.20220216 | 0.712496783 | 0.106934741 | 0.24704595 | 0.009299092 | 0.422236088 |
| LINC00958 | Epi4 | blue | 0.085771764 | 0.004726679 | 0.105178235 | 0.02779138 | 0.126237816 | 0.049615615 | 0.009949437 | 0.064027255 | 0.06289447 |
| RRAS2 | Epi4 | blue | 0.047106936 | -0.005864352 | 0.021694096 | 0.028490756 | 0.056599748 | -0.000298683 | 0.059676255 | -0.022020928 | 0.043662459 |
| RPS13 | Epi4 | blue | 0.339606703 | 0.004813976 | 0.343026157 | 0.12700706 | 0.599797522 | 0.097308108 | 0.208430912 | 0.039637943 | 0.335689877 |
| PRRG4 | Epi4 | blue | 0.081497081 | 0.00339919 | 0.07904079 | 0.039340781 | 0.123174247 | 0.036880057 | 0.085624534 | -0.03142512 | 0.109504107 |
| LRRC4C | Epi4 | blue | -0.0030726 | -0.032486247 | 0.015343861 | -0.008296828 | 0.075457852 | 0.011049466 | 0.002425594 | -0.063812104 | 0.039236438 |
| CTNND1 | Epi4 | blue | 0.118974385 | 0.06139926 | 0.063998935 | 0.030740214 | 0.099665437 | 0.059559313 | 0.099208126 | 0.000108664 | 0.065534592 |
| FTH1 | Epi4 | blue | 0.470193841 | 0.193119096 | 0.475414483 | 0.182291065 | 0.596679016 | 0.161809555 | 0.108350901 | 0.202216267 | 0.352331402 |
| FAU | Epi4 | blue | 0.366553004 | 0.074201614 | 0.347374515 | 0.244099429 | 0.59369666 | 0.119962886 | 0.227492717 | 0.049582073 | 0.381711323 |
| CST6 | Epi4 | blue | 0.116017598 | 0.020662651 | 0.074959493 | 0.08501641 | 0.172664805 | 0.030752736 | 0.146980855 | -0.091725736 | 0.183603566 |
| GAL3ST3 | Epi4 | blue | 0.052536668 | -0.052515467 | 0.029665553 | 0.029821665 | 0.082848342 | 0.009642195 | 0.099370728 | -0.063212401 | 0.094174076 |
| UCP2 | Epi4 | blue | 0.126074848 | -0.070971836 | 0.130200296 | 0.050238444 | 0.243532994 | 0.032818754 | 0.186229477 | -0.088548737 | 0.220476825 |
| PGM2L1 | Epi4 | blue | 0.068847368 | 0.011167277 | 0.057610322 | 0.040870884 | 0.105642782 | 0.018708736 | 0.071285694 | -0.050247169 | 0.102539916 |
| RPS3 | Epi4 | blue | 0.339818034 | 0.051452381 | 0.277177053 | 0.150657217 | 0.618378758 | 0.117079096 | 0.250843611 | 0.002575552 | 0.310465957 |
| TMEM123 | Epi4 | blue | 0.143514148 | -0.004422073 | 0.06173345 | 0.1099328 | 0.150465402 | 0.054550958 | 0.206384912 | -0.058080887 | 0.142419513 |
| ATP5L | Epi4 | blue | 0.293670695 | 0.028053165 | 0.308953719 | 0.216291964 | 0.343142498 | 0.084422833 | 0.185387744 | 0.123015005 | 0.296870598 |
| RPS25 | Epi4 | blue | 0.386629066 | 0.075207047 | 0.386782988 | 0.169305149 | 0.653226255 | 0.132311543 | 0.198325023 | 0.082381518 | 0.339258499 |
| ETS1 | Epi4 | blue | 0.045893944 | 0.049239027 | 0.05910537 | 0.016204853 | 0.026128226 | 0.047788822 | -0.008955443 | 0.017375992 | 0.026621017 |
| OPCML | Epi4 | blue | 0.09143874 | 0.014666678 | 0.079656 | 0.029956782 | 0.151488924 | 0.065859972 | 0.063933207 | -0.041917656 | 0.111510284 |
| CELF2 | Epi4 | blue | 0.106912742 | 0.031653578 | 0.12516678 | 0.031802179 | 0.106668977 | 0.03642872 | 0.029402484 | 0.040058079 | 0.091495555 |
| VIM | Epi4 | blue | 0.338534272 | -0.018295492 | 0.448891717 | 0.246884341 | 0.405450399 | 0.099268939 | 0.100906506 | 0.217901078 | 0.40716441 |
| PLXDC2 | Epi4 | blue | 0.048847174 | 0.002120775 | 0.057150099 | 0.028493914 | 0.059113646 | 0.023839759 | 0.028055264 | 0.030892077 | 0.056228363 |
| MAPK8 | Epi4 | blue | 0.020228565 | -0.029608908 | 0.024822151 | 0.005509378 | 0.043676491 | 0.014076451 | 0.030218139 | -0.020604496 | 0.028168339 |
| ARID5B | Epi4 | blue | 0.065535456 | -0.036931545 | 0.022192395 | 0.008619644 | 0.166248239 | 0.02201933 | 0.123942524 | -0.109984858 | 0.130586464 |
| RPS24 | Epi4 | blue | 0.351159755 | -0.025349078 | 0.391168255 | 0.148671628 | 0.678334945 | 0.111373249 | 0.178367093 | 0.041175016 | 0.405327625 |
| PDLIM1 | Epi4 | blue | 0.210284791 | -0.016496521 | 0.245423125 | 0.188921072 | 0.329506272 | 0.061719539 | 0.156607543 | 0.006810476 | 0.358347171 |
| RAB11FIP2 | Epi4 | blue | 0.041001094 | -0.026637559 | 0.055952719 | -0.008259352 | 0.055342267 | 0.018959726 | 0.026823619 | 0.010623567 | 0.053832612 |
| PTPRE | Epi4 | blue | 0.067185075 | -0.017662967 | 0.120579311 | 0.000176927 | 0.097800502 | 0.000485665 | 0.004326829 | 0.026531686 | 0.088853545 |
| TCERG1L | Epi4 | blue | 0.04932646 | -0.101155257 | 0.083155486 | 0.012506146 | 0.150194849 | 0.023462024 | 0.086122497 | -0.083336148 | 0.149558452 |
| PRMT8 | Epi4 | blue | 0.017037803 | -0.094083236 | 0.06704039 | 0.007360345 | 0.133952881 | 0.02601721 | 0.051406441 | -0.097286421 | 0.143859682 |
| PTMS | Epi4 | blue | 0.170972675 | -0.016203848 | 0.186952614 | 0.036493887 | 0.238449945 | 0.054148029 | 0.05237577 | 0.059498776 | 0.164039188 |
| YBX3 | Epi4 | blue | 0.144820405 | 0.083054628 | 0.099787197 | 0.018210348 | 0.13015679 | 0.042643052 | 0.075335891 | 0.007472778 | 0.058352129 |
| ERP27 | Epi4 | blue | 0.094197538 | 0.047116387 | 0.045264631 | 0.039322958 | 0.143047037 | 0.03660737 | 0.087690406 | -0.066096316 | 0.095889735 |
| BHLHE41 | Epi4 | blue | 0.109061037 | -0.005746815 | 0.100659245 | 0.04220847 | 0.106243426 | 0.043726248 | 0.060228638 | 0.010788234 | 0.092896094 |
| RP11-476D10.1 | Epi4 | blue | 0.059388482 | -0.013437448 | 0.07639844 | 0.049385456 | 0.074455358 | 0.045286645 | 0.022683446 | 0.026153286 | 0.084002335 |
| RP11-469H8.6 | Epi4 | blue | 0.056583739 | -0.063364444 | 0.104527866 | 0.023812508 | 0.123099239 | 0.023785033 | 0.011780766 | 0.029365844 | 0.109176934 |
| METTL7A | Epi4 | blue | 0.075425528 | -0.000292209 | 0.027177733 | 0.067522642 | 0.051228718 | 0.013056791 | 0.12422216 | -0.049721479 | 0.089456791 |
| PFDN5 | Epi4 | blue | 0.286036 | 0.025499854 | 0.292811401 | 0.199219143 | 0.469588208 | 0.083277562 | 0.16083093 | 0.066374941 | 0.31825555 |
| PCBP2 | Epi4 | blue | 0.213088685 | 0.063655434 | 0.187040312 | 0.128596512 | 0.226462896 | 0.076315425 | 0.116170226 | 0.074106639 | 0.154497661 |
| ATP5G2 | Epi4 | blue | 0.192920412 | 0.05756028 | 0.134655697 | 0.158310867 | 0.275939938 | 0.044822765 | 0.166886893 | 0.01321993 | 0.169651897 |
| HNRNPA1 | Epi4 | blue | 0.283230196 | 0.054275035 | 0.232439915 | 0.13875838 | 0.402137053 | 0.079679621 | 0.157052323 | 0.025521732 | 0.242874014 |
| RPL41 | Epi4 | blue | 0.537553587 | 0.192816946 | 0.56417875 | 0.190260169 | 0.777688232 | 0.180677125 | 0.145474995 | 0.238051269 | 0.374576533 |
| MYL6B | Epi4 | blue | 0.12929929 | 0.022740783 | 0.149870542 | 0.067468419 | 0.157131132 | 0.047093074 | 0.037548511 | 0.066970221 | 0.113709216 |
| NACA | Epi4 | blue | 0.298190967 | 0.068853217 | 0.252765769 | 0.181387908 | 0.443134982 | 0.08588394 | 0.19091544 | 0.074124606 | 0.245544748 |
| NAP1L1 | Epi4 | blue | 0.178519306 | 0.000662922 | 0.173397901 | 0.103516735 | 0.227753623 | 0.065578756 | 0.070075369 | 0.081006685 | 0.169795081 |
| RPL6 | Epi4 | blue | 0.3174453 | -0.016740867 | 0.311984931 | 0.157098449 | 0.573028213 | 0.100815445 | 0.206261662 | 0.037594906 | 0.333499661 |
| C12orf49 | Epi4 | blue | 0.127594549 | 0.002600845 | 0.113317681 | 0.052828447 | 0.178575076 | 0.214938732 | 0.089099288 | -0.03661201 | 0.151124397 |
| RPLP0 | Epi4 | blue | 0.338113104 | 0.141085632 | 0.231786351 | 0.123195275 | 0.492905442 | 0.115192562 | 0.237029765 | -0.037376029 | 0.245838135 |
| PIWIL1 | Epi4 | blue | 0.038511289 | -0.08613834 | 0.104050522 | -0.000957292 | 0.067094064 | 0.008904073 | -0.005791211 | 0.032657418 | 0.093346574 |
| RPL21 | Epi4 | blue | 0.454202221 | 0.063498806 | 0.407531975 | 0.184718514 | 0.734240543 | 0.147500451 | 0.244566914 | 0.084736082 | 0.378312996 |
| TPT1 | Epi4 | blue | 0.457549589 | 0.117165274 | 0.46331858 | 0.165209334 | 0.637142525 | 0.149581157 | 0.150966198 | 0.203804633 | 0.331895127 |
| COMMD6 | Epi4 | blue | 0.277880566 | 0.044279183 | 0.276845375 | 0.179048535 | 0.336915726 | 0.078915631 | 0.166487076 | 0.089647396 | 0.242727348 |
| LMO7-AS1 | Epi4 | blue | 0.060677723 | -0.028258253 | 0.061567657 | 0.010220357 | 0.110494939 | 0.011859215 | 0.060051709 | -0.004574534 | 0.068275374 |
| FAM177A1 | Epi4 | blue | 0.148274166 | 0.034308609 | 0.159710019 | 0.094553234 | 0.225182042 | 0.044903462 | 0.092853024 | -0.001530608 | 0.197806956 |
| RPS29 | Epi4 | blue | 0.419052958 | 0.079215864 | 0.443724028 | 0.158427754 | 0.721984373 | 0.126511038 | 0.199947163 | 0.055647037 | 0.402341143 |
| RPL36AL | Epi4 | blue | 0.206227153 | 0.063341135 | 0.177055406 | 0.09131868 | 0.328189685 | 0.055891945 | 0.103011787 | 0.059578195 | 0.136242276 |
| TMEM30B | Epi4 | blue | 0.123959829 | 0.037976275 | 0.067762717 | 0.075312516 | 0.12785361 | 0.037898839 | 0.092363223 | -0.031523594 | 0.111940951 |
| GTF2A1 | Epi4 | blue | 0.066804745 | 0.004199279 | 0.037048478 | 0.011649859 | 0.07181211 | 0.024939282 | 0.071740089 | -0.008737962 | 0.051463409 |
| BTBD7 | Epi4 | blue | 0.047365135 | -0.007040816 | 0.044412626 | -0.001331891 | 0.067960918 | 0.036523276 | 0.023829569 | -0.000776189 | 0.048092795 |
| SERF2 | Epi4 | blue | 0.432628307 | 0.087528648 | 0.462174531 | 0.206419929 | 0.48010845 | 0.127506895 | 0.196421991 | 0.18206751 | 0.361646592 |
| DUOX2 | Epi4 | blue | 0.082048413 | -0.150607489 | 0.198044115 | 0.014103984 | 0.145541058 | 0.00821461 | 0.03632688 | 0.024376368 | 0.20343462 |
| FBN1 | Epi4 | blue | 0.027575947 | -0.030497797 | 0.025326698 | 0.013118377 | 0.064623185 | 0.003953819 | 0.05729107 | -0.033515247 | 0.053013027 |
| RSL24D1 | Epi4 | blue | 0.14720547 | -0.005997911 | 0.109888854 | 0.136564912 | 0.22725215 | 0.056477329 | 0.123346968 | 0.031756019 | 0.158674279 |
| SNX22 | Epi4 | blue | 0.061444845 | -0.055425708 | 0.09050786 | 0.019719172 | 0.157351665 | 0.032251902 | 0.088711883 | -0.103327222 | 0.163562844 |
| RPL4 | Epi4 | blue | 0.293421769 | 0.023551797 | 0.270101887 | 0.110967096 | 0.507604412 | 0.092459544 | 0.169299512 | 0.032269008 | 0.253662287 |
| RPLP1 | Epi4 | blue | 0.429652659 | 0.068237668 | 0.431607107 | 0.155056097 | 0.734715153 | 0.131987603 | 0.261194894 | 0.003489998 | 0.431452243 |
| RPS17 | Epi4 | blue | 0.365849491 | 0.101062245 | 0.317880625 | 0.164854542 | 0.604966476 | 0.106147886 | 0.228572925 | 0.030198611 | 0.31220909 |
| RPS2 | Epi4 | blue | 0.331638828 | 0.151963588 | 0.166336507 | 0.131387464 | 0.477637106 | 0.107613091 | 0.280875377 | -0.048208633 | 0.184821807 |
| SNHG19 | Epi4 | blue | 0.053894524 | -0.070432184 | -0.007444421 | 0.052124587 | 0.144701224 | 0.025380931 | 0.17207793 | -0.106374751 | 0.0873584 |
| RPS15A | Epi4 | blue | 0.372955304 | 0.020631658 | 0.357997016 | 0.130939463 | 0.724805555 | 0.104758349 | 0.252626719 | -0.035575762 | 0.372609319 |
| MAF | Epi4 | blue | 0.091295678 | 0.023488189 | 0.06343692 | 0.026779835 | 0.130345373 | 0.063540445 | 0.049445034 | -0.02653348 | 0.101285688 |
| GCSH | Epi4 | blue | 0.165032701 | -0.101051548 | 0.16663757 | 0.158684405 | 0.197235024 | 0.033353201 | 0.216143718 | -0.02837217 | 0.271127786 |
| COX4I1 | Epi4 | blue | 0.258558704 | 0.092365117 | 0.269241431 | 0.260749859 | 0.31298971 | 0.063805039 | 0.14988424 | 0.130944767 | 0.279481043 |
| RPL13 | Epi4 | blue | 0.430868723 | 0.156163034 | 0.384578284 | 0.160617328 | 0.707162938 | 0.14554399 | 0.187588829 | 0.095699731 | 0.312135746 |
| RPL26 | Epi4 | blue | 0.434184168 | 0.064669564 | 0.391153332 | 0.184465148 | 0.733921405 | 0.131885792 | 0.272818658 | 0.045078612 | 0.384701246 |
| RPL23A | Epi4 | blue | 0.35163113 | 0.058443362 | 0.357579038 | 0.149060287 | 0.630980104 | 0.08814299 | 0.161282683 | 0.071035703 | 0.330644827 |
| RPL23 | Epi4 | blue | 0.322139604 | 0.08913565 | 0.317744818 | 0.135453342 | 0.514194728 | 0.082251051 | 0.166552077 | 0.067944282 | 0.278364169 |
| RPL19 | Epi4 | blue | 0.432526672 | 0.120961061 | 0.418225319 | 0.227510395 | 0.638670766 | 0.147527778 | 0.228054795 | 0.12094023 | 0.387652208 |
| RPL27 | Epi4 | blue | 0.344758442 | 0.07861556 | 0.386607477 | 0.197279143 | 0.513367222 | 0.109945204 | 0.173177678 | 0.129970324 | 0.34898512 |
| MYL4 | Epi4 | blue | 0.041056422 | -0.046525765 | -0.007924997 | 0.038165117 | 0.096365238 | 0.021469788 | 0.142008138 | -0.089879091 | 0.086544801 |
| RPL38 | Epi4 | blue | 0.337173798 | 0.023735107 | 0.358870611 | 0.159457632 | 0.614673955 | 0.076743817 | 0.204434648 | 0.074223088 | 0.359641091 |
| SUMO2 | Epi4 | blue | 0.331435916 | 0.067983163 | 0.392585994 | 0.194866257 | 0.345313987 | 0.096066754 | 0.068423546 | 0.222104301 | 0.292785424 |
| LIPG | Epi4 | blue | 0.035956908 | -0.0751906 | -0.011902049 | 0.02648117 | 0.05158807 | -0.018857885 | 0.126545329 | -0.092613416 | 0.076656455 |
| MACROD2 | Epi4 | blue | 0.16786269 | -0.103230475 | 0.274223649 | 0.089979219 | 0.25837615 | 0.057973517 | 0.086883992 | 0.048882068 | 0.288155634 |
| FLRT3 | Epi4 | blue | 0.137550346 | -0.024222307 | 0.165606889 | 0.062131716 | 0.204847398 | 0.05225671 | 0.063810983 | -0.020258064 | 0.198538565 |
| ZFAS1 | Epi4 | blue | 0.205543376 | -0.032133083 | 0.157945874 | 0.044521367 | 0.42173118 | 0.054328052 | 0.18085719 | -0.086294166 | 0.202364443 |
| RPS21 | Epi4 | blue | 0.316779238 | 0.064589254 | 0.330560493 | 0.137206505 | 0.569083029 | 0.103023492 | 0.136184221 | 0.075258456 | 0.304128475 |
| PPDPF | Epi4 | blue | 0.37524394 | 0.136630596 | 0.345778256 | 0.165636688 | 0.398780577 | 0.101361476 | 0.171619706 | 0.138371117 | 0.248500541 |
| RPS15 | Epi4 | blue | 0.385868856 | 0.081152834 | 0.375458578 | 0.217356016 | 0.647490111 | 0.115036575 | 0.209928047 | 0.106185316 | 0.358914129 |
| EEF2 | Epi4 | blue | 0.217669275 | 0.040619978 | 0.133557763 | 0.095708437 | 0.326007579 | 0.059148554 | 0.177724618 | -0.008631928 | 0.148158065 |
| RPL36 | Epi4 | blue | 0.429811637 | 0.241889963 | 0.425624654 | 0.191382916 | 0.635954535 | 0.134990944 | 0.106642739 | 0.193125272 | 0.295251148 |
| ALKBH7 | Epi4 | blue | 0.118670102 | -0.025063737 | 0.098325041 | 0.122809777 | 0.176602744 | 0.040959437 | 0.129268326 | -3.70E-05 | 0.163127552 |
| SLC25A23 | Epi4 | blue | 0.077374267 | 0.000124075 | 0.076932456 | -0.010704098 | 0.103202252 | 0.022514329 | 0.07838518 | 0.000348969 | 0.049699247 |
| RPS28 | Epi4 | blue | 0.488059414 | 0.297928609 | 0.483890922 | 0.202364554 | 0.65768492 | 0.156007042 | 0.113680575 | 0.258594959 | 0.297519122 |
| RPL18A | Epi4 | blue | 0.357685381 | 0.072221754 | 0.288725037 | 0.172073248 | 0.638229891 | 0.122479932 | 0.26588701 | -0.032063392 | 0.331273732 |
| UBA52 | Epi4 | blue | 0.342715419 | 0.075500469 | 0.371784473 | 0.220065372 | 0.504186597 | 0.101497508 | 0.16547262 | 0.120283051 | 0.347551992 |
| ZNF431 | Epi4 | blue | 0.053576953 | -0.019053143 | 0.031518585 | 0.02256053 | 0.079896261 | 0.03218107 | 0.064617739 | -0.038430418 | 0.058042639 |
| C19orf33 | Epi4 | blue | 0.197058419 | 0.107799316 | 0.176748942 | 0.070856326 | 0.265617569 | 0.054271032 | 0.101984339 | -0.006345723 | 0.175022922 |
| MAP4K1 | Epi4 | blue | 1.70E-05 | -0.085215903 | 0.082384833 | -0.002338035 | 0.097228649 | -0.00028143 | -0.003672796 | -0.043531723 | 0.124555113 |
| RPS16 | Epi4 | blue | 0.31983445 | 0.057439411 | 0.309943032 | 0.119652674 | 0.61611225 | 0.102477982 | 0.204924149 | -0.023794156 | 0.329283015 |
| FBL | Epi4 | blue | 0.050539354 | -0.013217634 | 0.044867862 | 0.037843375 | 0.092120866 | 0.029930695 | 0.072447172 | -0.023540771 | 0.073937685 |
| RPS19 | Epi4 | blue | 0.34454569 | 0.265985436 | 0.278916268 | 0.119010634 | 0.564612861 | 0.093442601 | 0.103931659 | 0.036684867 | 0.200735944 |
| GLTSCR2 | Epi4 | blue | 0.136262365 | -0.007912439 | 0.115450761 | 0.074591501 | 0.289633276 | 0.044144249 | 0.105703726 | -0.010041432 | 0.152491405 |
| RPL18 | Epi4 | blue | 0.329981583 | 0.081406612 | 0.309292583 | 0.17680891 | 0.557995479 | 0.121071131 | 0.204265204 | 0.05774823 | 0.296599531 |
| RPL13A | Epi4 | blue | 0.427379854 | 0.113837093 | 0.389455942 | 0.150508906 | 0.769543726 | 0.150949842 | 0.238708673 | 0.023261489 | 0.369288294 |
| RPS11 | Epi4 | blue | 0.240077304 | 0.024374562 | 0.222791892 | 0.149294317 | 0.382609103 | 0.057530404 | 0.190605557 | 0.01243451 | 0.262167314 |
| RPS9 | Epi4 | blue | 0.39706555 | 0.177161932 | 0.322498256 | 0.175827886 | 0.636059199 | 0.137023445 | 0.215953931 | 0.053270522 | 0.305461651 |
| RPL28 | Epi4 | blue | 0.433533527 | 0.213572017 | 0.442960046 | 0.163142613 | 0.692145134 | 0.133872815 | 0.154069754 | 0.127396065 | 0.339332415 |
| RPS5 | Epi4 | blue | 0.356746978 | 0.113966518 | 0.296281139 | 0.153850583 | 0.595592352 | 0.128976142 | 0.22063509 | 0.022036685 | 0.291503889 |
| RPS4Y1 | Epi4 | blue | 0.199010829 | 0.095437747 | 0.135519414 | 0.144546002 | 0.327684196 | 0.100824995 | 0.148773966 | 0.02825981 | 0.171513581 |
| HIRA | Epi4 | blue | 0.020448638 | -0.060234506 | 0.0008607 | 0.013911499 | 0.048266495 | -0.017012686 | 0.088654161 | -0.07355715 | 0.055806811 |
| EIF3L | Epi4 | blue | 0.149902706 | 0.004732744 | 0.097786508 | 0.094778381 | 0.252372465 | 0.056397708 | 0.119212949 | 0.001729824 | 0.142983188 |
| RPL3 | Epi4 | blue | 0.456512703 | 0.102336982 | 0.41710139 | 0.168024436 | 0.725927037 | 0.164100765 | 0.236789859 | 0.082122815 | 0.374314152 |
| SMDT1 | Epi4 | blue | 0.198496578 | 0.020220467 | 0.212366204 | 0.11926206 | 0.241404282 | 0.065566554 | 0.082103562 | 0.10019696 | 0.169587588 |
| LINC01315 | Epi4 | blue | 0.113048061 | -0.117069138 | 0.21175174 | 0.024887395 | 0.170562782 | 0.044872796 | -0.002374724 | 0.104809276 | 0.170898917 |
| MIR99AHG | Epi4 | blue | 0.041558388 | -0.053546984 | 0.064195737 | 0.01607894 | 0.053796369 | 0.006094214 | 0.024891221 | 0.023168065 | 0.06887068 |
| TIAM1 | Epi4 | blue | 0.105024233 | -0.063774437 | 0.168212224 | 0.005994544 | 0.151964578 | 0.051482803 | 0.040014912 | 0.002162128 | 0.180697406 |
| HMGN1 | Epi4 | blue | 0.355738286 | 0.126256487 | 0.365257346 | 0.099071856 | 0.321320598 | 0.124465204 | 0.042346425 | 0.21660794 | 0.190528946 |
| C1orf174 | Epi5 | yellow | 0.039210433 | 0.021301317 | 0.011425316 | 0.034467836 | 0.028374501 | 0.108792924 | 0.032911699 | 0.019543942 | 0.022613669 |
| CDC42 | Epi5 | yellow | 0.174000223 | 0.096004287 | 0.169636635 | 0.076208101 | 0.108817183 | 0.727070082 | 0.015774085 | 0.122638766 | 0.097702736 |
| NIPAL3 | Epi5 | yellow | 0.065425316 | 0.012809122 | 0.062552067 | 0.016876701 | 0.037038952 | 0.115097457 | -0.005266482 | 0.044632015 | 0.024464499 |
| FAM76A | Epi5 | yellow | 0.058349087 | 0.010096856 | 0.06907628 | 0.029422762 | 0.034524192 | 0.324231517 | 0.033223501 | 0.035780898 | 0.047249786 |
| EYA3 | Epi5 | yellow | 0.0392874 | 0.007946536 | 0.029152486 | 0.020115307 | 0.030492945 | 0.152123663 | 0.012888886 | 0.022549714 | 0.032847517 |
| SDC3 | Epi5 | yellow | 0.064851912 | 0.05440863 | 0.092193941 | 0.034039381 | 0.033031485 | 0.052905594 | -0.022673747 | 0.086261269 | 0.05009229 |
| SYNC | Epi5 | yellow | 0.048423829 | 0.037646563 | 0.045665471 | 0.043776083 | 0.028283061 | 0.139280901 | -0.029190166 | 0.066001909 | -0.000848875 |
| THRAP3 | Epi5 | yellow | 0.108660675 | 0.053243628 | 0.080948733 | 0.100838979 | 0.067139106 | 0.170077649 | 0.060255904 | 0.052857304 | 0.082868761 |
| GPBP1L1 | Epi5 | yellow | 0.092870969 | 0.031419456 | 0.086200441 | 0.025484734 | 0.078223391 | 0.03790142 | -0.003169332 | 0.048791183 | 0.030331451 |
| NFIA | Epi5 | yellow | 0.128343542 | 0.051834192 | 0.113371794 | 0.044259639 | 0.072695827 | 0.136096241 | 0.023904705 | 0.103258253 | 0.012914432 |
| INADL | Epi5 | yellow | 0.055454973 | 0.023290804 | 0.035544874 | 0.029576978 | 0.036326573 | 0.049460586 | 0.037574306 | 0.010446266 | 0.034834517 |
| BCL10 | Epi5 | yellow | 0.050511307 | 0.074834089 | 0.025571169 | 0.026143045 | 0.014124081 | 0.201872488 | 0.013492956 | 0.050007162 | 0.00705535 |
| F3 | Epi5 | yellow | 0.107957168 | 0.100906477 | 0.126021964 | 0.126597828 | 0.008549055 | 0.260372518 | -0.029826106 | 0.194303623 | 0.065334948 |
| CNN3 | Epi5 | yellow | 0.183635443 | 0.1752505 | 0.131222178 | 0.149081541 | 0.091446397 | 0.389207619 | 0.032446213 | 0.113688599 | 0.102954181 |
| CAPZA1 | Epi5 | yellow | 0.162133752 | 0.117566667 | 0.158360444 | 0.133842521 | 0.096574558 | 0.388759719 | 0.014945753 | 0.140974372 | 0.099270301 |
| TRIM33 | Epi5 | yellow | 0.086543335 | 0.052659172 | 0.074942298 | 0.051435131 | 0.063731276 | 0.063401565 | 0.0308708 | 0.051375608 | 0.035984374 |
| SEC22B | Epi5 | yellow | 0.07891264 | 0.024170222 | 0.038382237 | 0.057299804 | 0.061246996 | 0.328170862 | 0.039693587 | 0.009449229 | 0.045156601 |
| OTUD7B | Epi5 | yellow | 0.036505076 | 0.007629657 | 0.036259838 | 0.00370826 | 0.031797124 | 0.169237361 | 0.024307664 | 0.012289249 | 0.014845384 |
| ANP32E | Epi5 | yellow | 0.093816776 | 0.058821549 | 0.088679293 | 0.052446553 | 0.04170613 | 0.177661017 | 0.004256446 | 0.101684563 | 0.044577716 |
| C1orf56 | Epi5 | yellow | 0.070929192 | 0.065804074 | 0.056615025 | 0.032042977 | 0.024725211 | 0.704448663 | -0.015480949 | 0.082843208 | 0.021263341 |
| CDC42SE1 | Epi5 | yellow | 0.079629289 | 0.075622859 | 0.066168092 | 0.020568213 | 0.031349395 | 0.642596374 | -0.030157478 | 0.088666397 | 0.016021264 |
| RIT1 | Epi5 | yellow | 0.068267458 | 0.046452732 | 0.044554097 | 0.053370335 | 0.025006402 | 0.154639981 | 0.032324145 | 0.045619765 | 0.044056728 |
| ATP1B1 | Epi5 | yellow | 0.091273554 | 0.117467527 | 0.030138357 | 0.051538249 | 0.003879307 | 0.72293118 | 0.008333676 | 0.081515498 | -0.01390903 |
| PIGC | Epi5 | yellow | 0.084573626 | 0.051446517 | 0.065370379 | 0.069841018 | 0.047707324 | 0.046663266 | 0.052841777 | 0.054232841 | 0.040457067 |
| RASAL2 | Epi5 | yellow | 0.073975413 | 0.066331613 | 0.095767956 | 0.029130168 | 0.018311378 | 0.075347205 | -0.022968361 | 0.099004497 | 0.00996063 |
| TOR1AIP2 | Epi5 | yellow | 0.096876923 | 0.06127832 | 0.09089171 | 0.040579822 | 0.048120368 | 0.296796387 | -0.008893306 | 0.09952061 | 0.020543649 |
| RNF2 | Epi5 | yellow | 0.065526318 | 0.022719604 | 0.044750696 | 0.044965528 | 0.0733947 | 0.116694205 | 0.062822836 | 0.002757376 | 0.047665703 |
| MDM4 | Epi5 | yellow | 0.047844528 | 0.004353588 | 0.042389498 | 0.020380449 | 0.029676571 | 0.385269969 | 0.021818545 | 0.014237358 | 0.033750558 |
| RBBP5 | Epi5 | yellow | 0.061457369 | 0.016427188 | 0.038158141 | 0.039332996 | 0.025601285 | 0.195800563 | 0.043202004 | 0.025757773 | 0.038317393 |
| NUCKS1 | Epi5 | yellow | 0.193630346 | 0.075675529 | 0.157916491 | 0.090975035 | 0.134609211 | 0.347502041 | 0.073396606 | 0.113306893 | 0.097617404 |
| RRP15 | Epi5 | yellow | 0.054018206 | 0.00735316 | 0.038049975 | 0.010793199 | 0.047218515 | 0.086748517 | 0.046132712 | 0.005886011 | 0.007115846 |
| BROX | Epi5 | yellow | 0.08474289 | 0.046863428 | 0.056952435 | 0.059903056 | 0.01586052 | 0.06943559 | 0.022068371 | 0.047264012 | 0.027979741 |
| ASAP2 | Epi5 | yellow | 0.071396784 | 0.039061392 | 0.083687459 | 0.020591711 | 0.050475916 | 0.065988652 | 0.020846006 | 0.049393942 | 0.030031155 |
| IAH1 | Epi5 | yellow | 0.121306345 | 0.098379039 | 0.10635438 | 0.119651076 | 0.089090248 | 0.286824165 | 0.024601758 | 0.10432447 | 0.105441828 |
| ADAM17 | Epi5 | yellow | 0.065684886 | 0.026324873 | 0.044514921 | 0.033814266 | 0.039019142 | 0.388033886 | -0.001664129 | 0.03240693 | 0.042504321 |
| SLC35F6 | Epi5 | yellow | 0.050446797 | 0.038087218 | 0.018322736 | 0.020738763 | 0.011413317 | 0.188345952 | 0.034360924 | 0.016013586 | 0.011723623 |
| PPP1CB | Epi5 | yellow | 0.147606035 | 0.088103196 | 0.111623751 | 0.061608776 | 0.136488897 | 0.654550541 | 0.04173478 | 0.053632836 | 0.081597717 |
| WDR43 | Epi5 | yellow | 0.030524615 | 0.031496314 | 0.01558278 | 0.018620481 | 0.005626851 | 0.180692382 | 0.012132681 | 0.014505154 | 0.02242632 |
| C2orf68 | Epi5 | yellow | 0.041445171 | 0.018470824 | 0.029998831 | 0.024749627 | 0.026992944 | 0.116674251 | 0.001376638 | 0.025653743 | 0.036352793 |
| LRP2 | Epi5 | yellow | 0.044351186 | -0.036641098 | 0.043290441 | 0.039507034 | 0.042109746 | 0.223403977 | 0.053629121 | 0.007087667 | 0.054794248 |
| TLK1 | Epi5 | yellow | 0.086515416 | 0.043339508 | 0.06233369 | 0.063350389 | 0.048525865 | 0.158076159 | 0.00674902 | 0.072644358 | 0.025223367 |
| PLEKHA3 | Epi5 | yellow | 0.117852041 | 0.099636828 | 0.096492172 | 0.056180218 | 0.052693249 | 0.091555373 | -0.022930093 | 0.09736376 | 0.0445602 |
| RQCD1 | Epi5 | yellow | 0.058646975 | 0.027831037 | 0.027179252 | 0.030083916 | 0.026540929 | 0.300107548 | 0.040857111 | 0.018569223 | 0.026101005 |
| FAM134A | Epi5 | yellow | 0.079851224 | 0.03600407 | 0.061950633 | 0.065778713 | 0.068259909 | 0.139969272 | 0.055098624 | 0.031381675 | 0.050767011 |
| B3GNT7 | Epi5 | yellow | 0.057357884 | 0.0989813 | 0.055700719 | 0.024541266 | -0.009585648 | 0.231967438 | -0.07247765 | 0.104948972 | -0.023893215 |
| PER2 | Epi5 | yellow | 0.047130992 | 0.038045955 | 0.021402287 | 0.014816965 | 0.014572456 | 0.288244027 | 0.004284456 | 0.031698423 | 0.00041972 |
| SETD5 | Epi5 | yellow | 0.05950973 | 0.043631285 | 0.038454996 | 0.02635204 | 0.004450617 | 0.315287479 | 0.016709974 | 0.025238957 | 0.006745666 |
| ARPC4 | Epi5 | yellow | 0.116080317 | 0.096766584 | 0.094614875 | 0.119159246 | 0.074558876 | 0.173822673 | 0.025818668 | 0.09285764 | 0.079829766 |
| XPC | Epi5 | yellow | 0.047992653 | 0.037454978 | 0.019577603 | 0.020444786 | 0.037306123 | 0.055198064 | 0.018856394 | 0.010051352 | 0.015781697 |
| CTNNB1 | Epi5 | yellow | 0.08568856 | 0.145993956 | 0.050645339 | 0.024046679 | -0.024156466 | 0.740292067 | -0.051260507 | 0.144212437 | -0.040603172 |
| TMEM42 | Epi5 | yellow | 0.05041375 | 0.019486651 | 0.029911835 | 0.047986994 | 0.048485311 | 0.049054084 | 0.06229173 | 0.009643301 | 0.048957431 |
| KIF9 | Epi5 | yellow | 0.09506838 | 0.066778355 | 0.074949566 | 0.07056491 | 0.036032763 | 0.07296986 | 0.060969587 | 0.069940056 | 0.03752959 |
| RBM6 | Epi5 | yellow | 0.043311502 | 0.002204718 | 0.019190888 | 0.016211077 | 0.023569459 | 0.284569988 | 0.026026943 | -0.007906827 | 0.034185421 |
| FOXP1 | Epi5 | yellow | 0.122095033 | 0.036926759 | 0.067417847 | 0.067573161 | 0.087663 | 0.163006446 | 0.085774701 | 0.018082665 | 0.072681084 |
| EIF4E3 | Epi5 | yellow | 0.049506626 | 0.033140175 | 0.04797841 | 0.009019981 | 0.025651209 | 0.037610186 | -0.011641388 | 0.043687402 | 0.015199895 |
| RAP2B | Epi5 | yellow | 0.063805492 | 0.108724213 | 0.003699682 | 0.017428033 | 0.049315236 | 0.146366854 | 0.033688859 | 0.00795451 | -0.019501749 |
| RSRC1 | Epi5 | yellow | 0.078241661 | 0.030690162 | 0.061827759 | 0.065604994 | 0.025314236 | 0.189044002 | 0.000723887 | 0.059444587 | 0.048723299 |
| GOLIM4 | Epi5 | yellow | 0.09434071 | 0.165565269 | 0.026005917 | 0.043802812 | 0.00600681 | 0.483872861 | 0.008718668 | 0.083817024 | -0.028883121 |
| SKIL | Epi5 | yellow | 0.05900295 | 0.059247513 | 0.02186063 | -0.010392834 | 0.038356145 | 0.076045016 | -0.02306722 | 0.039456436 | -0.021107558 |
| TBL1XR1 | Epi5 | yellow | 0.108156665 | 0.023056863 | 0.059924261 | 0.03827125 | 0.10315004 | 0.134222245 | 0.117781727 | -0.014355754 | 0.084385646 |
| DCUN1D1 | Epi5 | yellow | 0.073430026 | 0.040061169 | 0.050939091 | 0.047441935 | 0.04744293 | 0.18427108 | 0.05423604 | 0.045279001 | 0.02861377 |
| LIPH | Epi5 | yellow | 0.073081135 | 0.08251574 | 0.086588796 | 0.036271145 | 0.028383962 | 0.279479584 | -0.036209444 | 0.100800321 | 1.91E-05 |
| ETV5 | Epi5 | yellow | 0.112920922 | 0.05174485 | 0.132803648 | 0.04918273 | 0.073182783 | 0.114444271 | 0.013074007 | 0.083551605 | 0.091857447 |
| RNF168 | Epi5 | yellow | 0.046780419 | 0.064981653 | 0.016698369 | 0.019362166 | 0.007541453 | 0.080815958 | -0.011740152 | 0.057722543 | -0.000758201 |
| SENP5 | Epi5 | yellow | 0.033379947 | 0.024506816 | 0.025102655 | 0.02397527 | 0.003964355 | 0.12667839 | 0.010913477 | 0.022609165 | 0.017922975 |
| CTBP1 | Epi5 | yellow | 0.093162468 | 0.073040154 | 0.091797636 | 0.020790698 | 0.052023122 | 0.095136078 | 0.01192357 | 0.079470693 | 0.033884568 |
| TMEM33 | Epi5 | yellow | 0.090724851 | 0.035563397 | 0.089901245 | 0.045746388 | 0.055223883 | 0.10973624 | 0.001448315 | 0.061991045 | 0.055438315 |
| UBA6 | Epi5 | yellow | 0.045762661 | -0.003855219 | 0.030657298 | 0.00284194 | 0.034843961 | 0.210969347 | 0.02306764 | -0.00556628 | 0.032697353 |
| UNC5C | Epi5 | yellow | 0.037572776 | 0.045268014 | 0.025919741 | 0.025928868 | -0.007134467 | 0.029938552 | -0.005016755 | 0.035948526 | -0.005027578 |
| RAP1GDS1 | Epi5 | yellow | 0.082779663 | 0.033544126 | 0.086688186 | 0.034858644 | 0.069977592 | 0.199167709 | 0.023099851 | 0.048603114 | 0.057759373 |
| PPP3CA | Epi5 | yellow | 0.096307457 | 0.034750998 | 0.084483315 | 0.042672603 | 0.053095385 | 0.441406151 | 0.008582874 | 0.092519021 | 0.047852141 |
| 6-Mar | Epi5 | yellow | 0.125579904 | 0.068887445 | 0.086343558 | 0.065972337 | 0.062967415 | 0.192606006 | 0.043108713 | 0.068136384 | 0.044916503 |
| MYO10 | Epi5 | yellow | 0.052331791 | 0.047900361 | 0.021796429 | 0.023314234 | 0.028264923 | 0.032015195 | 0.049224247 | 0.021061884 | 0.018554518 |
| CDH6 | Epi5 | yellow | 0.101895283 | 0.024829162 | 0.059645132 | 0.042103778 | 0.103369105 | 0.152558386 | 0.049518329 | -0.029082445 | 0.086240051 |
| SMIM15 | Epi5 | yellow | 0.111412495 | 0.046442818 | 0.091012161 | 0.075849879 | 0.086965668 | 0.065327414 | 0.020909263 | 0.06833263 | 0.067036797 |
| BDP1 | Epi5 | yellow | 0.11003209 | 0.051251467 | 0.08468662 | 0.055132854 | 0.069936338 | 0.074105064 | 0.04882525 | 0.044669849 | 0.074821853 |
| SERINC5 | Epi5 | yellow | 0.061005235 | 0.045745089 | 0.028661735 | 0.028194657 | 0.04653388 | 0.194072284 | 0.006942194 | 0.022591645 | 0.027648935 |
| ARRDC3 | Epi5 | yellow | 0.14079346 | -0.054415893 | 0.108914404 | 0.08229178 | 0.186793325 | 0.221635721 | 0.172319215 | -0.059679214 | 0.176498361 |
| SLC12A2 | Epi5 | yellow | 0.079616822 | -0.011034974 | 0.080261581 | 0.018753436 | 0.089394222 | 0.241690778 | 0.033030849 | 0.020691111 | 0.070398482 |
| CDC42SE2 | Epi5 | yellow | 0.100166452 | 0.009324505 | 0.104568449 | 0.033291016 | 0.05842513 | 0.140150441 | -0.002958734 | 0.076913249 | 0.067011929 |
| FNIP1 | Epi5 | yellow | 0.058230274 | 0.015813875 | 0.040519535 | 0.023129214 | 0.021438388 | 0.148633819 | 0.019041759 | 0.019399942 | 0.021674933 |
| HSPA4 | Epi5 | yellow | 0.054637192 | 0.018357748 | 0.030479924 | 0.032996251 | 0.025514877 | 0.238350295 | 0.034650429 | 0.037460519 | 0.031735253 |
| CSNK1A1 | Epi5 | yellow | 0.187920442 | 0.09880742 | 0.164975754 | 0.086906668 | 0.139751993 | 0.281677035 | 0.032714741 | 0.10770494 | 0.118808197 |
| TNIP1 | Epi5 | yellow | 0.048887148 | 0.051297493 | 0.040063137 | -0.005765087 | 0.055648425 | 0.054014761 | 0.026153878 | 0.010879371 | -0.001447351 |
| HNRNPH1 | Epi5 | yellow | 0.067705791 | 0.02346981 | 0.038698305 | 0.037349864 | 0.029917969 | 0.766442192 | 0.01951629 | 0.047205492 | 0.037022201 |
| RREB1 | Epi5 | yellow | 0.069354678 | 0.007474278 | 0.076231358 | 0.026201088 | 0.035585278 | 0.270177464 | 0.010713544 | 0.039949688 | 0.017346889 |
| RNF144B | Epi5 | yellow | 0.08896542 | -0.085053191 | 0.081354545 | 0.031899549 | 0.157776178 | 0.356548543 | 0.121945732 | -0.070909909 | 0.152580101 |
| CDKAL1 | Epi5 | yellow | 0.078843503 | 0.031406721 | 0.055842607 | 0.038743518 | 0.082573008 | 0.271448187 | 0.045004348 | 0.010499605 | 0.056635137 |
| LRRC16A | Epi5 | yellow | 0.026533653 | -0.005826057 | 0.016626691 | -0.015511452 | 0.042277715 | 0.278018074 | 0.010058037 | -0.028307072 | 0.020203892 |
| TRIM38 | Epi5 | yellow | 0.071066954 | 0.035474117 | 0.052780891 | 0.041676192 | 0.040175081 | 0.087300294 | 0.044487889 | 0.045540179 | 0.031597625 |
| ZNRD1 | Epi5 | yellow | 0.045105003 | -0.00413865 | 0.033256242 | 0.065172508 | 0.046125308 | 0.046456881 | 0.02251984 | 0.025036595 | 0.063039757 |
| BAG6 | Epi5 | yellow | 0.066597803 | 0.064715805 | 0.029283079 | 0.077746407 | 0.030417336 | 0.05498726 | 0.045148785 | 0.029977845 | 0.049119844 |
| SRSF3 | Epi5 | yellow | 0.205015619 | 0.129716606 | 0.139920235 | 0.178407613 | 0.107634781 | 0.185477189 | 0.11418906 | 0.106418335 | 0.13015545 |
| PRDM1 | Epi5 | yellow | 0.07866446 | 0.047687561 | 0.098080509 | 0.013957298 | 0.013469331 | 0.361651882 | -0.029823632 | 0.096910267 | 0.01928597 |
| TSPYL1 | Epi5 | yellow | 0.145920532 | 0.053203414 | 0.132476877 | 0.033089742 | 0.107224265 | 0.595013548 | -0.00078873 | 0.104534618 | 0.06359523 |
| EZR | Epi5 | yellow | 0.140365592 | 0.11635467 | 0.078846461 | 0.145290478 | 0.071885091 | 0.263138951 | 0.089580043 | 0.049949538 | 0.128892882 |
| WTAP | Epi5 | yellow | 0.098452346 | 0.043096463 | 0.076485487 | 0.062714045 | 0.06875543 | 0.526092841 | 0.023381224 | 0.057920277 | 0.061022084 |
| COX19 | Epi5 | yellow | 0.032074978 | 0.022797324 | 0.014568227 | 0.00897924 | 0.008622548 | 0.025167256 | 0.014451814 | 0.018130115 | 0.000960241 |
| TRA2A | Epi5 | yellow | 0.099236691 | 0.031731168 | 0.052383947 | 0.052460915 | 0.038289279 | 0.472077021 | 0.044002654 | 0.049475071 | 0.041473115 |
| TMED4 | Epi5 | yellow | 0.111546184 | 0.019112773 | 0.095002047 | 0.146331155 | 0.084537544 | 0.337900749 | 0.084796062 | 0.045464277 | 0.15018498 |
| SUMF2 | Epi5 | yellow | 0.072984095 | 0.077710177 | 0.024384483 | 0.072380852 | 0.017508964 | 0.426172304 | 0.034316387 | 0.056754739 | 0.027989878 |
| PHKG1 | Epi5 | yellow | 0.034802889 | 0.061535206 | 0.011129743 | 0.005801871 | 0.002566704 | 0.542563899 | -0.008632876 | 0.035579166 | -0.009550556 |
| GIGYF1 | Epi5 | yellow | 0.057460088 | 0.028872042 | 0.035191299 | 0.039228399 | 0.03769628 | 0.318771639 | 0.016852293 | 0.033681065 | 0.012632358 |
| KMT2E | Epi5 | yellow | 0.107757031 | 0.055333874 | 0.081368526 | 0.045662564 | 0.038385115 | 0.100464649 | 0.028151279 | 0.076546245 | 0.022930095 |
| PNPLA8 | Epi5 | yellow | 0.07635286 | 0.056452124 | 0.035133876 | 0.049720436 | 0.012100376 | 0.072354678 | 0.032051515 | 0.042464396 | 0.026788169 |
| THAP5 | Epi5 | yellow | 0.071826357 | 0.027863259 | 0.04961431 | 0.043077153 | 0.052865062 | 0.29836322 | 0.01214831 | 0.035203484 | 0.045140767 |
| SND1 | Epi5 | yellow | 0.020482483 | -0.00074412 | 0.005610237 | 0.038887491 | 0.026161899 | 0.043554995 | 0.070256979 | -0.033897645 | 0.047752555 |
| EIF2S3 | Epi5 | yellow | 0.078505228 | 0.004946544 | 0.027310677 | 0.051162224 | 0.112654864 | 0.150876782 | 0.079434157 | 0.000720445 | 0.056182419 |
| ZFX | Epi5 | yellow | 0.063096561 | 0.047632884 | 0.034398579 | 0.037043712 | 0.026226067 | 0.181433033 | 0.010949232 | 0.036104957 | 0.039028041 |
| TSPYL2 | Epi5 | yellow | 0.050600057 | 0.045761451 | 0.05918142 | 0.026343666 | -0.013844424 | 0.046022686 | -0.009754233 | 0.083100623 | 0.003791697 |
| APOOL | Epi5 | yellow | 0.055462734 | 0.023687212 | 0.03944799 | 0.012331811 | 0.037678122 | 0.119122237 | 0.035622226 | 0.02438386 | 0.032773176 |
| RAP2C | Epi5 | yellow | 0.03930638 | -0.005683855 | 0.040925976 | 0.027889018 | 0.033305362 | 0.082353948 | 0.007633601 | 0.023119035 | 0.060798279 |
| PHF6 | Epi5 | yellow | 0.087063341 | 0.052768012 | 0.058710157 | 0.040823591 | 0.046648771 | 0.081042649 | 0.010909041 | 0.045323205 | 0.035362345 |
| ZDHHC2 | Epi5 | yellow | 0.105775374 | 0.065453794 | 0.101687812 | 0.041880305 | 0.081580196 | 0.478373354 | 0.016130959 | 0.065943922 | 0.063564992 |
| CNOT7 | Epi5 | yellow | 0.185321383 | 0.071145235 | 0.153447349 | 0.095141683 | 0.125553253 | 0.113857492 | 0.061186169 | 0.110911665 | 0.09860291 |
| STC1 | Epi5 | yellow | 0.056533318 | 0.146073209 | 0.019235099 | 0.02235882 | -0.0082321 | 0.176944889 | -0.038485604 | 0.074769037 | -0.035761769 |
| DOCK5 | Epi5 | yellow | 0.031667683 | 0.001703063 | 0.06229784 | -0.008784919 | 0.025357815 | 0.515238246 | -0.026698634 | 0.054722258 | 0.016008562 |
| PCMTD1 | Epi5 | yellow | 0.071276345 | 0.041258041 | 0.033155669 | 0.055940183 | 0.021445666 | 0.168405075 | 0.045110564 | 0.03822843 | 0.02611033 |
| TRPS1 | Epi5 | yellow | 0.034660455 | 0.005431175 | 0.025383639 | 0.025671799 | 0.014564032 | 0.168180169 | 0.027484985 | 0.020869186 | 0.022728133 |
| SAMD12 | Epi5 | yellow | 0.065167683 | 0.052032267 | 0.048699973 | 0.023425911 | 0.029338661 | 0.362474534 | -0.000915201 | 0.038584421 | 0.019092737 |
| B4GALT1 | Epi5 | yellow | 0.088779655 | 0.124601029 | 0.005453835 | 0.050334729 | 0.014883947 | 0.585608027 | 0.037245941 | 0.042908297 | -0.000307367 |
| ABCA1 | Epi5 | yellow | 0.074354093 | 0.058703828 | 0.043879495 | 0.013996391 | 0.028305944 | 0.064737702 | 0.021528925 | 0.05935705 | 0.004529844 |
| SLC44A1 | Epi5 | yellow | 0.130528242 | 0.06392706 | 0.077478443 | 0.042078502 | 0.07897923 | 0.090128723 | 0.052434087 | 0.049066964 | 0.044552299 |
| ZNF462 | Epi5 | yellow | 0.043578045 | -0.010252597 | 0.054980095 | -0.004121305 | 0.065379354 | 0.222691475 | 0.011276276 | 0.013906638 | 0.041349294 |
| SET | Epi5 | yellow | 0.142742839 | 0.019660824 | 0.120342165 | 0.028096429 | 0.147531275 | 0.520159416 | 0.059364028 | 0.014519371 | 0.098949295 |
| BRD3 | Epi5 | yellow | 0.072847737 | 0.028343597 | 0.043457514 | 0.043079336 | 0.031065448 | 0.093697805 | 0.060647374 | 0.020674236 | 0.029385338 |
| FAM111A | Epi5 | yellow | 0.070718773 | 0.066622637 | 0.031313671 | 0.037208177 | 0.028405302 | 0.075094298 | 0.027473299 | 0.0361433 | 0.010309643 |
| CTTN | Epi5 | yellow | 0.135424531 | 0.098437517 | 0.0991641 | 0.045940702 | 0.082162741 | 0.34099939 | -0.000824116 | 0.107683684 | 0.039990058 |
| PAFAH1B2 | Epi5 | yellow | 0.077560118 | 0.024951333 | 0.055758467 | 0.024566863 | 0.073250589 | 0.121368935 | 0.05507924 | 0.016100612 | 0.050645858 |
| KIAA1217 | Epi5 | yellow | 0.123965108 | 0.017111418 | 0.1412515 | 0.043034829 | 0.126793383 | 0.147695975 | 0.087827792 | 0.003369705 | 0.131970393 |
| REEP3 | Epi5 | yellow | 0.116254437 | 0.094689968 | 0.117699488 | 0.052073708 | 0.084265877 | 0.186713542 | 0.00930262 | 0.104182239 | 0.049943891 |
| SAR1A | Epi5 | yellow | 0.105872446 | 0.077511321 | 0.070134478 | 0.081742749 | 0.043442299 | 0.457431558 | 0.045963499 | 0.070137772 | 0.067036006 |
| SGPL1 | Epi5 | yellow | 0.039797708 | 0.051164166 | 0.024257156 | 0.02162391 | 0.011086853 | 0.069395258 | 0.020063723 | 0.001804071 | 0.004337008 |
| PTEN | Epi5 | yellow | 0.096770971 | 0.073627728 | 0.048464197 | 0.047418347 | 0.038918501 | 0.085246821 | 0.027647908 | 0.024780563 | 0.016877571 |
| EIF3A | Epi5 | yellow | 0.1281175 | 0.067223343 | 0.081580399 | 0.059673306 | 0.073241822 | 0.1082556 | 0.04152934 | 0.068474255 | 0.043831414 |
| TIAL1 | Epi5 | yellow | 0.085983391 | 0.056797075 | 0.054294844 | 0.050551917 | 0.070618024 | 0.206075051 | 0.044706835 | 0.023445975 | 0.030957712 |
| ETNK1 | Epi5 | yellow | 0.0964855 | 0.047600525 | 0.095446205 | 0.062030123 | 0.052646809 | 0.094887347 | 0.026321049 | 0.085219011 | 0.058819329 |
| ARID2 | Epi5 | yellow | 0.040447518 | 0.013108612 | 0.030453181 | 0.02617978 | 0.037761451 | 0.192775868 | -0.008025186 | 0.023855215 | 0.039267659 |
| SLC48A1 | Epi5 | yellow | 0.070328902 | 0.008061388 | 0.062457552 | 0.055064228 | 0.025926062 | 0.111770653 | 0.007058896 | 0.071596539 | 0.042897431 |
| SMARCD1 | Epi5 | yellow | 0.053074782 | 0.013713936 | 0.049738431 | 0.036986799 | 0.022661947 | 0.048942454 | 0.028382275 | 0.030714794 | 0.040602554 |
| TNS2 | Epi5 | yellow | 0.015021691 | 0.015579127 | 0.025079828 | -0.003648288 | -0.009614035 | 0.050020584 | -0.01894772 | 0.025644553 | -0.006414414 |
| R3HDM2 | Epi5 | yellow | 0.069757064 | 0.024346763 | 0.041745897 | 0.019812383 | 0.051323367 | 0.227471475 | 0.016112535 | 0.035012808 | 0.018652734 |
| TSPAN31 | Epi5 | yellow | 0.091637182 | 0.064327128 | 0.077979841 | 0.087366296 | 0.064862354 | 0.047724185 | 0.016041872 | 0.061209474 | 0.070036168 |
| RASSF3 | Epi5 | yellow | 0.050568226 | -0.01430943 | 0.080154196 | 0.009816269 | 0.035877747 | 0.304963139 | -0.007991866 | 0.064186871 | 0.049859395 |
| RITA1 | Epi5 | yellow | 0.045054537 | 0.004147033 | 0.029840484 | 0.028247252 | 0.035393079 | 0.054334631 | 0.011612651 | 0.00941584 | 0.047244064 |
| SUDS3 | Epi5 | yellow | 0.091726651 | 0.036378851 | 0.057681448 | 0.022980594 | 0.094791955 | 0.048072074 | 0.061388805 | 0.012261434 | 0.03885441 |
| RHOF | Epi5 | yellow | 0.076122848 | 0.028116709 | 0.077994622 | 0.063292562 | 0.059470519 | 0.081716483 | 0.04262705 | 0.051164773 | 0.071486696 |
| KMT5A | Epi5 | yellow | 0.05197283 | 0.001976395 | 0.035751909 | 0.028843923 | 0.051768808 | 0.368840341 | 0.040009382 | 0.006346823 | 0.050956263 |
| SHISA2 | Epi5 | yellow | 0.080080469 | -0.006192151 | 0.135458165 | 0.03465625 | 0.076593443 | 0.264277609 | -0.059462696 | 0.115391547 | 0.072379727 |
| PAN3 | Epi5 | yellow | 0.074091072 | 0.017075058 | 0.056979147 | 0.062339953 | 0.059957122 | 0.133863808 | 0.062948232 | 0.03503742 | 0.056574688 |
| PDS5B | Epi5 | yellow | 0.077331761 | 0.024911309 | 0.074374137 | 0.020701387 | 0.066411634 | 0.121886276 | 0.019572458 | 0.042020328 | 0.035800402 |
| DNAJC3 | Epi5 | yellow | 0.115579557 | 0.094366068 | 0.056527179 | 0.082709775 | 0.022313029 | 0.271673853 | 0.023731161 | 0.092280225 | 0.022223681 |
| TPP2 | Epi5 | yellow | 0.050733024 | 0.031172829 | 0.033660426 | 0.052936691 | 0.021462961 | 0.142057624 | 0.022697003 | 0.044535847 | 0.038906712 |
| STRN3 | Epi5 | yellow | 0.064658903 | 0.031649276 | 0.051876686 | 0.026123973 | 0.031885523 | 0.326971435 | -0.010469503 | 0.04973913 | 0.03066466 |
| ARF6 | Epi5 | yellow | 0.150940873 | 0.112139318 | 0.144026223 | 0.051310551 | 0.092216846 | 0.157441203 | 0.005024923 | 0.113161087 | 0.057450663 |
| ERO1A | Epi5 | yellow | 0.069792685 | 0.068243378 | 0.065919809 | 0.040049987 | 0.033354928 | 0.113337497 | -0.005910135 | 0.067020271 | 0.015623253 |
| PSMA3-AS1 | Epi5 | yellow | 0.070165462 | 0.013055677 | 0.064228931 | 0.045576034 | 0.050414398 | 0.139236095 | 0.018749303 | 0.042050101 | 0.04592127 |
| SUSD6 | Epi5 | yellow | 0.048401672 | 0.040321905 | 0.049929546 | 0.031985188 | 0.004486647 | 0.158683507 | -0.014357226 | 0.084958409 | -0.001318901 |
| SYNJ2BP | Epi5 | yellow | 0.078668187 | 0.057725884 | 0.031122046 | 0.071153421 | 0.027964867 | 0.143219121 | 0.048236958 | 0.022127172 | 0.045345585 |
| ELMSAN1 | Epi5 | yellow | 0.069534756 | 0.044496437 | 0.027064124 | 0.023903069 | 0.030619987 | 0.182120515 | 0.006619773 | 0.028203881 | 0.012326299 |
| IRF2BPL | Epi5 | yellow | 0.093168276 | 0.100411595 | 0.044123133 | 0.03766976 | 0.029544523 | 0.267006816 | 0.022357964 | 0.040988959 | 0.010493935 |
| SPTLC2 | Epi5 | yellow | 0.039814484 | 0.019467578 | 0.050335059 | 0.017223795 | 0.029522663 | 0.016960071 | 0.007329867 | 0.019905711 | 0.032991981 |
| PPP2R5C | Epi5 | yellow | 0.127496546 | 0.045119393 | 0.11480012 | 0.063121294 | 0.098734602 | 0.387838023 | 0.027716529 | 0.04915034 | 0.102493761 |
| CHP1 | Epi5 | yellow | 0.112320664 | 0.044953237 | 0.084264294 | 0.053475594 | 0.138624028 | 0.367329805 | 0.073534613 | 0.001014279 | 0.085303685 |
| MYO5C | Epi5 | yellow | 0.047943254 | 0.062044287 | 0.001463094 | 0.025070529 | 0.005147926 | 0.096223876 | 0.028392684 | 0.020277662 | -0.008527388 |
| IGDCC4 | Epi5 | yellow | 0.059225021 | -0.006171881 | 0.080684371 | 0.035682878 | 0.050865331 | 0.137789493 | 0.006306957 | 0.049787195 | 0.061759915 |
| TLE3 | Epi5 | yellow | 0.044516501 | 0.013356273 | 0.040755485 | 0.012679279 | 0.030514969 | 0.112213891 | -0.000504939 | 0.034776997 | 0.005786571 |
| TMEM159 | Epi5 | yellow | 0.061326253 | -0.000302171 | 0.026260355 | 0.045799385 | 0.059191502 | 0.17727185 | 0.046743081 | 0.00476648 | 0.061680292 |
| MAZ | Epi5 | yellow | 0.081666296 | 0.035610456 | 0.058790027 | 0.032005371 | 0.067654281 | 0.082239206 | 0.019913161 | 0.039122767 | 0.043601544 |
| AMFR | Epi5 | yellow | 0.050491581 | 0.076343814 | 0.042357289 | 0.01689521 | -0.015341043 | 0.342783094 | -0.02023459 | 0.079341984 | -0.01802321 |
| NFATC3 | Epi5 | yellow | 0.050552061 | -0.017617501 | 0.007800281 | 0.036801086 | 0.079127401 | 0.136463823 | 0.09558164 | -0.071233466 | 0.055805759 |
| EXOSC6 | Epi5 | yellow | 0.083072851 | 0.05794104 | 0.054161555 | 0.0417045 | 0.052144793 | 0.11975233 | 0.020053922 | 0.034422426 | 0.026814913 |
| AP1G1 | Epi5 | yellow | 0.059911388 | 0.058260223 | 0.043007583 | 0.019245673 | -0.00268262 | 0.152360283 | 0.003525222 | 0.056091676 | 0.00752229 |
| GLG1 | Epi5 | yellow | 0.080534256 | 0.047078632 | 0.055840848 | 0.049137778 | 0.020244317 | 0.119375164 | 0.03364658 | 0.033103513 | 0.018585805 |
| YWHAE | Epi5 | yellow | 0.240158026 | 0.125318248 | 0.209574229 | 0.205208784 | 0.188012197 | 0.262407365 | 0.107557229 | 0.143673168 | 0.178537382 |
| EIF5A | Epi5 | yellow | 0.102398348 | 0.075962278 | 0.091087042 | 0.084369931 | 0.034103522 | 0.438395906 | 0.021854372 | 0.085593519 | 0.067506669 |
| ZBTB4 | Epi5 | yellow | 0.043095224 | 0.031950422 | 0.04123029 | -0.014725121 | 0.029603864 | 0.022232735 | -0.009178709 | 0.017234385 | 0.010154912 |
| TP53 | Epi5 | yellow | 0.033618377 | 0.026020537 | 0.031909456 | 0.002762145 | 0.036641532 | 0.28024959 | 0.014091232 | 0.012504569 | 0.015639677 |
| LRRC75A | Epi5 | yellow | 0.04717364 | 0.038396852 | 0.035197893 | 0.027106086 | 0.039146314 | 0.236099434 | -0.002207324 | 0.026171026 | 0.033501973 |
| NF1 | Epi5 | yellow | 0.091372772 | 0.059179298 | 0.070534961 | 0.031304972 | 0.065057378 | 0.080123378 | 0.036474771 | 0.051556113 | 0.044318393 |
| NBR1 | Epi5 | yellow | 0.06743849 | 0.007712311 | 0.068867201 | 0.016461466 | 0.05373896 | 0.039715381 | 0.026440556 | 0.01983599 | 0.038170962 |
| LSM12 | Epi5 | yellow | 0.091816063 | 0.035574402 | 0.077300668 | 0.067875961 | 0.048821254 | 0.159534752 | 0.02240962 | 0.069134286 | 0.054898728 |
| SLC25A39 | Epi5 | yellow | 0.148083292 | 0.115558544 | 0.107776969 | 0.061662388 | 0.098042186 | 0.091215584 | 0.059984404 | 0.062031615 | 0.039619027 |
| DCAKD | Epi5 | yellow | 0.050601506 | 0.033903835 | 0.045277064 | 0.033538226 | 0.03532957 | 0.175397291 | 0.015451074 | 0.047601228 | 0.034666467 |
| KPNB1 | Epi5 | yellow | 0.072689636 | 0.070277642 | 0.041300652 | 0.044570778 | 0.028354794 | 0.082438707 | 0.012586824 | 0.036243023 | 0.023150061 |
| CALCOCO2 | Epi5 | yellow | 0.095675798 | 0.054241759 | 0.045839844 | 0.096787677 | 0.031889864 | 0.102197836 | 0.08089979 | 0.035297216 | 0.047926854 |
| ANKRD40 | Epi5 | yellow | 0.05701694 | 0.028792856 | 0.052350562 | 0.024113334 | 0.026032795 | 0.309061884 | -0.003261244 | 0.045620234 | 0.026332257 |
| RP11-294J22.6 | Epi5 | yellow | 0.051005836 | 0.012008908 | 0.056030121 | 0.021457415 | 0.030212135 | 0.333992421 | 0.00071989 | 0.054585635 | 0.029898007 |
| TOB1 | Epi5 | yellow | 0.073542647 | 0.074793867 | -0.01571777 | 0.067487563 | -0.011630272 | 0.100082692 | 0.099827045 | -0.018543799 | -0.004887643 |
| SKA2 | Epi5 | yellow | 0.068344742 | 0.025170473 | 0.022756297 | 0.056362644 | 0.047769987 | 0.108476362 | 0.064231627 | 0.009425623 | 0.053262249 |
| KCNJ2 | Epi5 | yellow | 0.108220106 | 0.169727667 | 0.078500335 | 0.047680414 | 0.018054916 | 0.469293198 | -0.049106748 | 0.144604681 | -0.025159008 |
| ACOX1 | Epi5 | yellow | 0.08431987 | 0.039355277 | 0.063354021 | 0.022258127 | 0.0652541 | 0.116206732 | 0.023793452 | 0.047280344 | 0.04351701 |
| SEPT9 | Epi5 | yellow | 0.054776967 | 0.064694354 | 0.042571823 | 0.012022297 | 0.028197211 | 0.066030899 | 0.011512961 | 0.054322476 | 0.00837935 |
| AFMID | Epi5 | yellow | 0.051903427 | 0.03845696 | 0.04776489 | 0.021668138 | 0.035221916 | 0.207797183 | -0.018836053 | 0.05293513 | 0.025193736 |
| RNF24 | Epi5 | yellow | 0.048679808 | 0.011833041 | 0.034699854 | 0.035326725 | 0.02482992 | 0.10612361 | 0.003945976 | 0.028587191 | 0.032149613 |
| RIN2 | Epi5 | yellow | 0.089238744 | 0.089711476 | 0.059614508 | 0.025175675 | 0.007983241 | 0.468546433 | -0.00310147 | 0.122720425 | -0.026559374 |
| LPIN3 | Epi5 | yellow | 0.013410723 | -0.003477862 | 0.007254652 | -0.00650592 | 0.004129728 | 0.137617545 | -0.002451768 | 0.020690807 | -0.007001008 |
| SRSF6 | Epi5 | yellow | 0.076640135 | 0.029339384 | 0.044295866 | 0.029982604 | 0.049519624 | 0.239730196 | 0.056353947 | 0.011818083 | 0.0431056 |
| SDC4 | Epi5 | yellow | 0.13740435 | 0.004173039 | 0.212673123 | 0.046145108 | 0.143268547 | 0.697624309 | -0.049792535 | 0.106689593 | 0.160996872 |
| PARD6B | Epi5 | yellow | 0.067798563 | 0.053663484 | 0.048413325 | 0.015597533 | 0.060391774 | 0.087412491 | 0.017041327 | 0.009037375 | 0.027536069 |
| ADNP | Epi5 | yellow | 0.096757831 | 0.06194175 | 0.058565121 | 0.022105405 | 0.067442259 | 0.245496768 | 0.023839848 | 0.035282303 | 0.025148438 |
| CSTF1 | Epi5 | yellow | 0.053056519 | 0.012130687 | 0.023231668 | 0.04571507 | 0.041831463 | 0.051362947 | 0.059027476 | 0.004464243 | 0.049854953 |
| OGFR | Epi5 | yellow | 0.059737258 | 0.062478288 | 0.027089374 | 0.060867219 | 0.015024214 | 0.055348268 | 0.048834928 | 0.037369961 | 0.016303428 |
| TIMM13 | Epi5 | yellow | 0.172314163 | 0.055031336 | 0.157284533 | 0.12352076 | 0.163522496 | 0.082425639 | 0.107140144 | 0.061483183 | 0.141021313 |
| SH3GL1 | Epi5 | yellow | 0.055336518 | 0.046771041 | 0.039696535 | 0.013636967 | 0.022939045 | 0.053383932 | -0.01285093 | 0.0523825 | -0.002404957 |
| UBXN6 | Epi5 | yellow | 0.095071517 | 0.042427179 | 0.084405033 | 0.066472976 | 0.086067857 | 0.106845074 | 0.052776191 | 0.038409816 | 0.071193899 |
| PTPRS | Epi5 | yellow | 0.043681622 | -0.039203098 | 0.033404192 | 0.027022569 | 0.047900046 | 0.543426462 | 0.041590601 | -0.013098807 | 0.059977577 |
| INSR | Epi5 | yellow | 0.060430073 | 0.016202934 | 0.048769552 | 0.005104079 | 0.027497746 | 0.406423467 | -0.027558376 | 0.061050826 | 0.006329255 |
| HOMER3 | Epi5 | yellow | 0.056463058 | -0.027508764 | 0.08378523 | 0.036892218 | 0.074073041 | 0.059970084 | 0.033624781 | 0.017421899 | 0.092036405 |
| GATAD2A | Epi5 | yellow | 0.068795214 | 0.064025025 | 0.048305108 | 0.020312126 | 0.041167493 | 0.045398844 | 0.017329712 | 0.036802658 | 0.013014024 |
| LINC00665 | Epi5 | yellow | 0.061052143 | 0.02222821 | 0.038419364 | 0.045350614 | 0.048261276 | 0.0494829 | 0.040332305 | 0.021098412 | 0.029931857 |
| ZC3H4 | Epi5 | yellow | 0.044332307 | 0.035738676 | 0.012808509 | 0.017287698 | 0.012466963 | 0.289776352 | 0.014902114 | 0.01589064 | 0.023836448 |
| EMC10 | Epi5 | yellow | 0.133458426 | 0.088146845 | 0.093756719 | 0.11634561 | 0.088388113 | 0.114515287 | 0.083954276 | 0.048496235 | 0.104737809 |
| CBX6 | Epi5 | yellow | 0.113366846 | 0.064473353 | 0.082314585 | 0.059044437 | 0.075087772 | 0.440582055 | 0.027744612 | 0.08947249 | 0.05045801 |
| APOBEC3C | Epi5 | yellow | 0.060172857 | 0.059883318 | 0.046176603 | 0.022107416 | 0.037696637 | 0.296959831 | 0.000184653 | 0.036476345 | 0.004029833 |
| TNRC6B | Epi5 | yellow | 0.135543668 | 0.084154341 | 0.099313184 | 0.053051178 | 0.02843583 | 0.333430528 | 0.018478718 | 0.107094991 | 0.01209156 |
| TOB2 | Epi5 | yellow | 0.115842622 | 0.073229084 | 0.096858079 | 0.077979074 | 0.04039021 | 0.052459467 | 0.025321233 | 0.101465855 | 0.045436789 |
| NUP50 | Epi5 | yellow | 0.022235765 | 0.004720713 | 0.016001898 | -0.007287474 | -0.010986974 | 0.098801548 | -0.001974411 | 0.006479555 | -0.003947245 |
| DENND6B | Epi5 | yellow | 0.03625495 | 0.060911471 | 0.04361651 | 0.038487039 | 0.013219885 | 0.048471698 | -0.016873842 | 0.057082711 | -0.000449347 |
| ID3 | Epi6 | green | 0.076052099 | -0.109932113 | -0.001893364 | 0.105694854 | 0.100747074 | 0.037176189 | 0.392428135 | -0.166847947 | 0.168461815 |
| STMN1 | Epi6 | green | 0.041316883 | 0.021145121 | -0.052110321 | 0.05907458 | 0.006719724 | 0.015695144 | 0.116068614 | -0.052176791 | 0.030336069 |
| UBXN11 | Epi6 | green | 0.078800746 | 0.050855405 | 0.035018547 | 0.058010657 | 0.025976753 | 0.032670094 | 0.073351534 | 0.035496362 | 0.030692706 |
| TMEM125 | Epi6 | green | 0.051781078 | 0.010339527 | 0.020023231 | 0.02802841 | 0.071714081 | 0.030153449 | 0.104361951 | -0.050516492 | 0.063183283 |
| TSPAN1 | Epi6 | green | 0.042844613 | 0.057113493 | -0.111260279 | 0.154272874 | 0.002743614 | -0.011763976 | 0.281591976 | -0.11996787 | 0.03738728 |
| TXNDC12 | Epi6 | green | 0.06218363 | 0.05313462 | 0.020822979 | 0.088771985 | 0.015973994 | 0.018543864 | 0.065918928 | 0.015276317 | 0.025517905 |
| HSPB11 | Epi6 | green | 0.08159261 | 0.05899652 | 0.032550702 | 0.136395592 | 0.042615812 | 0.04164109 | 0.141456078 | 0.000358603 | 0.097719962 |
| ACADM | Epi6 | green | 0.067262463 | 0.018071346 | 0.023132863 | 0.060329748 | 0.030219469 | 0.01401033 | 0.082536165 | 0.016854159 | 0.030107766 |
| DNAJB4 | Epi6 | green | 0.049721741 | 0.019846087 | -0.028823112 | 0.096123805 | -0.011605441 | 0.03178637 | 0.178056927 | -0.028312639 | 0.032505054 |
| GSTM4 | Epi6 | green | 0.038028591 | -0.001143932 | -0.01293027 | 0.047381393 | 0.005763759 | 0.023929892 | 0.120642977 | -0.030123613 | 0.028348568 |
| RORC | Epi6 | green | 0.019130247 | 0.018559843 | -0.030460785 | 0.034522319 | 0.029411219 | 0.015128502 | 0.078921494 | -0.02620472 | 0.019468871 |
| EFNA1 | Epi6 | green | 0.068245311 | 0.031907072 | -0.016073383 | 0.080938312 | 0.040592877 | 0.029035657 | 0.155804294 | -0.050513739 | 0.057847794 |
| SSR2 | Epi6 | green | 0.125579478 | 0.058714996 | 0.045506348 | 0.095986503 | 0.138503097 | 0.028666986 | 0.119609912 | -0.002455253 | 0.071472656 |
| RGS5 | Epi6 | green | 0.057914701 | 0.026068316 | -0.018813625 | 0.073125443 | 0.037660298 | 0.051392648 | 0.069076784 | -0.017012309 | 0.028366269 |
| CREG1 | Epi6 | green | 0.071505901 | 0.044154209 | 0.002946858 | 0.102954227 | 0.039440814 | 0.015843033 | 0.142486962 | -0.030153441 | 0.072689384 |
| GLUL | Epi6 | green | 0.10973032 | -0.02489693 | 0.02576124 | 0.147714793 | 0.108359899 | 0.04243816 | 0.239423225 | -0.072379367 | 0.137366942 |
| MARC1 | Epi6 | green | 0.041355141 | -0.009521196 | 0.010153893 | 0.06098886 | 0.035393576 | -7.48E-05 | 0.130878207 | -0.015433584 | 0.059486758 |
| TPO | Epi6 | green | -0.018942033 | -0.04766769 | -0.186083469 | 0.116087685 | -0.111217603 | -0.023873087 | 0.310905064 | -0.193502196 | -0.013497023 |
| ID2 | Epi6 | green | 0.107517255 | 0.016393601 | -0.000613755 | 0.13012168 | 0.090735821 | 0.036822897 | 0.302447768 | -0.071039198 | 0.115915035 |
| ZFP36L2 | Epi6 | green | 0.084355671 | -0.010693579 | -0.105986222 | 0.103979004 | 0.067256101 | 0.002887001 | 0.348930858 | -0.18818913 | 0.055896685 |
| TPRKB | Epi6 | green | 0.105016209 | 0.057913062 | 0.060219185 | 0.105159685 | 0.088020562 | 0.064150794 | 0.080655942 | 0.034035321 | 0.088221946 |
| THNSL2 | Epi6 | green | 0.047877503 | 0.01086107 | 0.014295805 | 0.05366382 | 0.027284102 | 0.005464943 | 0.058303926 | 0.000340837 | 0.037950461 |
| STARD7 | Epi6 | green | 0.06368483 | 0.00838176 | 0.031144477 | 0.030638327 | 0.067984012 | 0.029515045 | 0.079517062 | -0.01097261 | 0.045783931 |
| PAX8 | Epi6 | green | 0.075279662 | -0.069598472 | -0.027576592 | 0.072046999 | 0.087658757 | 0.012257785 | 0.306449319 | -0.14626477 | 0.110978322 |
| EPB41L5 | Epi6 | green | 0.028085395 | 0.018480273 | -0.015488952 | 0.057047987 | -0.007939857 | -0.019969586 | 0.10417556 | -0.022476182 | 0.034349998 |
| CYBRD1 | Epi6 | green | 0.116945438 | 0.064904457 | 0.011794019 | 0.094093129 | 0.059401024 | 0.046011537 | 0.195798716 | -0.000869178 | 0.0395783 |
| IGFBP5 | Epi6 | green | 0.080376739 | -0.031917411 | -0.047203945 | 0.016874292 | 0.130468028 | 0.034563643 | 0.216819944 | -0.130368013 | 0.049469903 |
| PTMA | Epi6 | green | 0.316598048 | 0.147314077 | 0.141784537 | 0.209560714 | 0.346074749 | 0.095522839 | 0.258655247 | 0.008178449 | 0.20735132 |
| SEPT2 | Epi6 | green | 0.121236572 | 0.061608919 | 0.083903353 | 0.124601644 | 0.073205132 | 0.065775873 | 0.087802478 | 0.036143733 | 0.105146275 |
| IP6K2 | Epi6 | green | 0.087846231 | 0.026624754 | 0.034506322 | 0.094327912 | 0.056946883 | 0.001153734 | 0.091723136 | 0.024304007 | 0.079730261 |
| IMPDH2 | Epi6 | green | 0.077856236 | 0.053752261 | 0.024610282 | 0.076041975 | 0.105769451 | 0.010382218 | 0.107223316 | -0.002941108 | 0.074494787 |
| TKT | Epi6 | green | 0.115796427 | 0.048518463 | 0.038810707 | 0.148750687 | 0.111319844 | 0.055881911 | 0.12667357 | 0.040019492 | 0.105541345 |
| MTRNR2L12 | Epi6 | green | 0.027606291 | -0.003884355 | -0.045457605 | 0.078890721 | -0.027517692 | 0.007049446 | 0.163831343 | -0.060773827 | 0.02139567 |
| ALCAM | Epi6 | green | 0.089073799 | 0.051532857 | 0.023292459 | 0.065152564 | 0.04885707 | 0.005817462 | 0.085698471 | 0.007416852 | 0.048478533 |
| ZBED2 | Epi6 | green | 0.044181825 | -0.06882331 | -0.017889342 | 0.045161769 | 0.085383648 | -0.012864233 | 0.248766014 | -0.129963395 | 0.094399319 |
| SLC12A8 | Epi6 | green | 0.0321301 | -0.021342239 | -0.046167837 | 0.071041824 | -0.01539175 | 0.007523267 | 0.163306204 | -0.069463174 | 0.027244845 |
| SNX4 | Epi6 | green | 0.036260249 | 0.032061164 | 0.004402639 | 0.080006097 | 0.008859635 | 0.025823998 | 0.06275203 | 0.022252694 | 0.014191961 |
| H1FX | Epi6 | green | 0.040338623 | 0.01728821 | -0.018724943 | 0.02875529 | 0.021426727 | 0.006553942 | 0.121113057 | -0.050040107 | 0.010552127 |
| PFN2 | Epi6 | green | 0.061626373 | -0.008593201 | -0.072615283 | 0.099901553 | 0.060487754 | -0.001956951 | 0.238819615 | -0.112701085 | 0.05558389 |
| HOPX | Epi6 | green | 0.153470413 | -0.049394961 | 0.056625726 | 0.171299451 | 0.176620186 | 0.037082708 | 0.336942698 | -0.070122847 | 0.213303024 |
| IGFBP7 | Epi6 | green | 0.027028923 | 0.017916593 | -0.11796012 | 0.095635567 | -0.030623537 | 0.032604203 | 0.217170961 | -0.127798137 | -0.003538422 |
| SCD5 | Epi6 | green | 0.062397336 | 0.078668491 | -0.042353397 | 0.059087129 | 0.04310548 | 0.023248439 | 0.158791905 | -0.06862498 | 0.009034405 |
| SORBS2 | Epi6 | green | 0.053309862 | 0.043004069 | -0.054384559 | 0.08560183 | -0.020359399 | -0.007292665 | 0.208778937 | -0.066595012 | 0.022625641 |
| MEF2C | Epi6 | green | 0.05805706 | 0.027514363 | 0.029747028 | 0.043110916 | 0.024413699 | 0.032172887 | 0.096374552 | 0.01517847 | 0.029337203 |
| TNFAIP8 | Epi6 | green | 0.031917421 | 0.008552156 | -0.027104322 | 0.060668234 | 0.017086141 | -0.01252843 | 0.083390027 | -0.042899901 | 0.042545859 |
| GRAMD3 | Epi6 | green | 0.059544102 | 0.00817871 | 0.007353201 | 0.068139876 | 0.08265435 | -0.002016296 | 0.16576362 | -0.072381217 | 0.080203585 |
| SPARC | Epi6 | green | 0.134720965 | 0.072527496 | 0.088558999 | 0.130992471 | 0.074849059 | 0.031376524 | 0.141229899 | 0.02259236 | 0.111045474 |
| BOD1 | Epi6 | green | 0.100901102 | 0.032595273 | 0.048728429 | 0.06937183 | 0.068501189 | 0.030842002 | 0.104571567 | 0.010932321 | 0.064663723 |
| THOC3 | Epi6 | green | 0.035076653 | 0.007572948 | -0.031550897 | 0.053848779 | 0.028559214 | 0.015724802 | 0.158342578 | -0.058649148 | 0.022092862 |
| CTC-338M12.5 | Epi6 | green | 0.044801818 | -0.019138412 | 0.015737464 | 0.03012606 | 0.048207067 | 0.001757037 | 0.083036629 | -0.002824014 | 0.023976538 |
| HSPA1A | Epi6 | green | 0.047956518 | 0.039790837 | -0.023631511 | 0.088708138 | -0.011952411 | 0.006383018 | 0.137344462 | -0.03730618 | 0.049593584 |
| HSPA1B | Epi6 | green | 0.051429867 | 0.036993246 | -0.018520604 | 0.075683591 | -0.012482169 | 0.017398698 | 0.130473292 | -0.027781077 | 0.031536493 |
| MTCH1 | Epi6 | green | 0.189185523 | 0.036804704 | 0.11342933 | 0.204116174 | 0.142667776 | 0.023988597 | 0.218603511 | 0.048787594 | 0.165105062 |
| CTGF | Epi6 | green | -0.006553557 | -0.022701139 | -0.134075812 | 0.063834358 | -0.069660731 | -0.009323812 | 0.270539517 | -0.155294338 | -0.007592641 |
| SGK1 | Epi6 | green | 0.061853969 | 0.009111309 | -0.009289999 | 0.09437384 | 0.03932107 | 0.013350672 | 0.164875081 | -0.020865746 | 0.071376524 |
| CITED2 | Epi6 | green | 0.083423128 | 0.021488314 | -0.058252607 | 0.120840608 | 0.009653138 | -0.004425258 | 0.263841741 | -0.101324692 | 0.054621349 |
| MPC1 | Epi6 | green | 0.060944803 | 0.008447171 | 0.033229711 | 0.077916625 | 0.037251705 | 0.008907562 | 0.125484128 | 0.011680685 | 0.041614674 |
| RNASET2 | Epi6 | green | 0.041556045 | -0.014671202 | -0.007974606 | 0.074847868 | 0.04333178 | 0.002895866 | 0.150013476 | -0.044668064 | 0.057306924 |
| POLD2 | Epi6 | green | 0.064626641 | 0.034881724 | 0.000650577 | 0.060471292 | 0.032770742 | 0.010195157 | 0.091390146 | -0.007896715 | 0.022620862 |
| SNHG15 | Epi6 | green | 0.059280427 | 0.014121693 | 0.035579187 | 0.030599804 | 0.046891299 | 0.011107098 | 0.054342156 | 0.010815265 | 0.041003943 |
| NUPR2 | Epi6 | green | 0.037939031 | 0.002223841 | -0.030205818 | 0.045690032 | 0.034916584 | -0.001985875 | 0.155924279 | -0.060713516 | 0.025497797 |
| CLDN3 | Epi6 | green | 0.110726349 | 0.079616323 | -0.05334695 | 0.204987443 | 0.066216011 | 0.00889548 | 0.358934495 | -0.126551068 | 0.117969064 |
| CLDN4 | Epi6 | green | 0.203405477 | 0.202042265 | 0.045724293 | 0.206892816 | 0.131238289 | 0.040797444 | 0.248219222 | -0.011799166 | 0.126863443 |
| APTR | Epi6 | green | 0.052763671 | 0.000193131 | 0.052209717 | 0.079161427 | 0.033916767 | 0.042822302 | 0.111069855 | -0.00209595 | 0.083867897 |
| PDK4 | Epi6 | green | 0.058360475 | 0.044748599 | 0.017619415 | 0.024145515 | -0.02248297 | 0.014924523 | 0.072685554 | 0.021820287 | -0.012882819 |
| POLR2J3 | Epi6 | green | 0.055709363 | -0.001565641 | -0.005480615 | 0.064944122 | 0.02259482 | 0.001099661 | 0.13091955 | -0.041114292 | 0.045172578 |
| SLC26A4-AS1 | Epi6 | green | 0.041663168 | -0.058505283 | -0.093805741 | 0.056162353 | 0.07805739 | 0.015601858 | 0.289907512 | -0.192421199 | 0.057802503 |
| SLC26A4 | Epi6 | green | 0.013921652 | -0.062017176 | -0.098149649 | 0.055207961 | 0.020288129 | 0.018550453 | 0.264869712 | -0.152476771 | 0.029695907 |
| CAV2 | Epi6 | green | 0.071584965 | -0.101637976 | -0.027574452 | 0.094048947 | 0.129058964 | 0.022550493 | 0.324861958 | -0.192070839 | 0.17120821 |
| CAV1 | Epi6 | green | -0.010823127 | -0.100687287 | -0.18339845 | 0.090139036 | 0.005851513 | -0.009559327 | 0.372897906 | -0.261386902 | 0.052874051 |
| SMS | Epi6 | green | 0.110977887 | 0.009247473 | 0.059473243 | 0.103920866 | 0.044857135 | 0.05013757 | 0.105157942 | 0.037478061 | 0.078734558 |
| SLC38A5 | Epi6 | green | 0.066003631 | -0.058417855 | 0.0218572 | 0.10636771 | 0.139329841 | -0.000225509 | 0.193836405 | -0.109509445 | 0.192521439 |
| IGBP1 | Epi6 | green | 0.10826876 | 0.023553126 | 0.064017677 | 0.088525439 | 0.148240656 | 0.030054128 | 0.106278955 | 0.018056961 | 0.099872442 |
| BEX2 | Epi6 | green | 0.135932728 | -0.007534904 | 0.087843557 | 0.123974382 | 0.074297698 | 0.049172631 | 0.140338069 | 0.059074295 | 0.102335809 |
| WBP5 | Epi6 | green | 0.108024944 | -0.031502197 | -0.020257447 | 0.176012402 | 0.07366785 | 0.01144789 | 0.339738474 | -0.095689305 | 0.145398904 |
| NGFRAP1 | Epi6 | green | 0.190926982 | 0.036225201 | 0.103203003 | 0.194250964 | 0.105360147 | 0.034695797 | 0.269465577 | 0.053714565 | 0.136637197 |
| PRPS1 | Epi6 | green | 0.059885336 | 0.044379209 | 0.024745658 | 0.077805827 | 0.021808357 | 0.012946454 | 0.085040526 | 0.009177598 | 0.028786575 |
| SLC25A5 | Epi6 | green | 0.120837862 | 0.096801225 | 0.081158347 | 0.105638221 | 0.131459091 | 0.018835633 | 0.113546007 | 0.00669506 | 0.084115493 |
| FHL1 | Epi6 | green | 0.031985658 | -0.02996774 | -0.054131877 | 0.092571863 | -0.01295084 | 0.018491338 | 0.223909616 | -0.066075992 | 0.030592026 |
| RBMX | Epi6 | green | 0.141424554 | 0.035243087 | 0.092924268 | 0.126999208 | 0.143310031 | 0.040828758 | 0.113474036 | 0.037970467 | 0.10172679 |
| INTS10 | Epi6 | green | 0.047035938 | -0.013564091 | 0.031987159 | 0.05572533 | 0.055171549 | 0.011909724 | 0.072157627 | -0.011497253 | 0.063803094 |
| NPM2 | Epi6 | green | 0.034949371 | -0.022277095 | 0.006490662 | 0.046259689 | 0.069866344 | 0.009628111 | 0.103475314 | -0.056143391 | 0.07564715 |
| RBPMS | Epi6 | green | 0.08622158 | 0.031948932 | 0.035117735 | 0.076718816 | 0.079585849 | 0.047898013 | 0.127719613 | -0.028999275 | 0.064970872 |
| VDAC3 | Epi6 | green | 0.091573456 | 0.039508495 | 0.021693084 | 0.134179605 | 0.059374478 | 0.005711712 | 0.172809934 | -0.000684555 | 0.096145716 |
| SLC20A2 | Epi6 | green | 0.056814797 | 0.087953496 | -0.002174191 | 0.043163206 | 0.004346839 | 0.013416426 | 0.08681759 | 0.004967353 | -0.003252116 |
| PKIA | Epi6 | green | 0.035321729 | -0.053404763 | 0.011853064 | 0.056179122 | 0.036099255 | 0.010011823 | 0.120533716 | -0.018122166 | 0.06820234 |
| SLC26A7 | Epi6 | green | -0.034933163 | -0.186137156 | -0.186886189 | 0.021728314 | 0.04994197 | -0.028704864 | 0.361609217 | -0.2848421 | 0.060146373 |
| MAL2 | Epi6 | green | 0.137631482 | 0.104590259 | 0.045316407 | 0.068517441 | 0.096562797 | 0.053073359 | 0.13832286 | -0.020933301 | 0.048673014 |
| DEPTOR | Epi6 | green | 0.045117862 | -0.019981712 | -0.023062276 | 0.057832789 | 0.031078694 | -0.005240794 | 0.211519219 | -0.071510733 | 0.042802767 |
| TG | Epi6 | green | 0.028096186 | -0.080383953 | -0.122291034 | 0.143431275 | -0.024781 | -0.013451236 | 0.377947073 | -0.212631179 | 0.08179793 |
| KLF4 | Epi6 | green | 0.051617543 | 0.04300086 | -0.011416359 | 0.013986888 | -0.000629297 | 0.015272605 | 0.099612976 | -0.016818739 | -0.011157644 |
| ASS1 | Epi6 | green | 0.078735274 | 0.026770624 | 0.035536695 | 0.089389402 | 0.081449102 | 0.033588514 | 0.06766637 | 0.027769202 | 0.064919308 |
| LCN12 | Epi6 | green | 0.026675701 | -0.024542989 | -0.032186161 | 0.070017121 | 0.004070698 | 0.002495211 | 0.093668517 | -0.002874168 | 0.034395255 |
| IFITM2 | Epi6 | green | 0.073111931 | 0.022742836 | -0.009023448 | 0.111287633 | 0.024307231 | 0.010705238 | 0.148609366 | -0.035982038 | 0.070387736 |
| IFITM3 | Epi6 | green | 0.115547454 | 0.207568528 | -0.027917818 | 0.184395591 | 0.021587646 | 0.026016376 | 0.20534727 | -0.020638839 | 0.055122023 |
| RASSF7 | Epi6 | green | 0.054893934 | 0.023607775 | 0.013819461 | 0.048353578 | 0.059985313 | 0.001057684 | 0.060602642 | -0.028153467 | 0.041046475 |
| CARS | Epi6 | green | 0.033309402 | -0.017021899 | -0.015170535 | 0.045305432 | 0.022951405 | 0.00681633 | 0.121135739 | -0.02308342 | 0.022648142 |
| MTRNR2L8 | Epi6 | green | 0.009766887 | 0.006000722 | -0.047375235 | 0.057328962 | -0.036570648 | -0.01782843 | 0.142264284 | -0.065553448 | 0.018393814 |
| C11orf74 | Epi6 | green | 0.030687862 | -0.033386456 | -0.055176498 | 0.06800464 | 0.008280477 | 0.001397334 | 0.211983978 | -0.086078587 | 0.034298315 |
| ARFGAP2 | Epi6 | green | 0.004565887 | 0.021946743 | -0.025504322 | 0.032887295 | 0.003678945 | 0.001356278 | 0.066295984 | -0.026868684 | 0.002665903 |
| EEF1G | Epi6 | green | 0.070738381 | 0.02377388 | 0.019017292 | 0.072188303 | 0.085533681 | 0.0097028 | 0.072825824 | -0.019199225 | 0.050243899 |
| GPR137 | Epi6 | green | 0.043826591 | 0.011524941 | 0.024030756 | 0.053500198 | 0.027609089 | -0.010448329 | 0.072163936 | -0.001578906 | 0.038355874 |
| CAPN1 | Epi6 | green | 0.057937552 | 0.005888771 | 0.014228022 | 0.080108738 | 0.054370352 | 0.012417331 | 0.09164048 | -0.031397405 | 0.077840403 |
| TMEM126B | Epi6 | green | 0.117519172 | 0.037823954 | 0.068545688 | 0.1388931 | 0.069424068 | 0.031519302 | 0.118825675 | 0.041638129 | 0.107200239 |
| CRYAB | Epi6 | green | 0.02377942 | 0.006992614 | -0.103229449 | 0.091200488 | -0.075682098 | 0.021866802 | 0.230833477 | -0.093119728 | -0.008846235 |
| RP11-158I9.8 | Epi6 | green | 0.037295905 | -0.017711745 | 0.027575244 | 0.031989019 | 0.032211256 | 0.025934542 | 0.074933901 | -0.002504094 | 0.029134787 |
| ESAM | Epi6 | green | 0.0506486 | -0.049748041 | 0.00918907 | 0.102603717 | 0.074045502 | -0.015173482 | 0.188094961 | -0.055634645 | 0.11637396 |
| NEBL | Epi6 | green | 0.050701541 | 0.003722074 | -0.007294294 | 0.03184617 | 0.037657498 | 0.002504257 | 0.113136242 | -0.025383978 | 0.025600539 |
| SRGN | Epi6 | green | 0.056583095 | 0.017488358 | -0.02625313 | 0.073385687 | 0.001619692 | 0.020758148 | 0.12272316 | -0.055883773 | 0.014702214 |
| GLUD1 | Epi6 | green | 0.069092053 | 0.004340292 | 0.032407745 | 0.082889607 | 0.044317469 | 0.005148101 | 0.126802183 | -0.007985894 | 0.085834905 |
| ACTA2 | Epi6 | green | 0.064431783 | 0.054422708 | 0.020666714 | 0.064206095 | 0.044159015 | 0.0252769 | 0.022542241 | 0.01395252 | 0.028254062 |
| AFAP1L2 | Epi6 | green | 0.017173 | -0.026102075 | -0.006418934 | 0.03192301 | 0.016318813 | 0.019794143 | 0.094795001 | -0.034073253 | 0.03932154 |
| ABLIM1 | Epi6 | green | 0.045257443 | 0.010833702 | -0.017944088 | 0.028715204 | 0.035113612 | -0.005366759 | 0.066684679 | -0.033226994 | 0.008094141 |
| RGS10 | Epi6 | green | 0.025365618 | -0.039664915 | -0.021935811 | 0.045371589 | 0.068107989 | 0.012093 | 0.149325399 | -0.094697018 | 0.047514877 |
| BNIP3 | Epi6 | green | 0.076150033 | 0.047703627 | 0.014415115 | 0.095202161 | 0.035494358 | 0.017245824 | 0.076423592 | 0.034640985 | 0.034131463 |
| DAZAP2 | Epi6 | green | 0.133272728 | 0.133127598 | 0.062709656 | 0.113954441 | 0.055052624 | 0.045379903 | 0.090234947 | 0.065683677 | 0.054212975 |
| KRT7 | Epi6 | green | 0.089906495 | 0.093637302 | -0.09850887 | 0.191622746 | 0.027576815 | 0.026420416 | 0.304920012 | -0.132298822 | 0.059384813 |
| HSD17B6 | Epi6 | green | 0.008407248 | -0.053081253 | -0.054035705 | 0.099855618 | 0.015891118 | -0.018418322 | 0.194193813 | -0.098095064 | 0.064251487 |
| DDIT3 | Epi6 | green | 0.123403766 | 0.065305013 | 0.028296182 | 0.128024398 | 0.020086641 | 0.045769545 | 0.147245229 | 0.019150377 | 0.046456654 |
| C12orf29 | Epi6 | green | 0.055773237 | -0.007574035 | 0.02817361 | 0.050338182 | 0.054993948 | 0.013865189 | 0.058292535 | -0.00499483 | 0.064592339 |
| IKBIP | Epi6 | green | 0.054692151 | 0.044423183 | 0.02747974 | 0.091178731 | 0.032950705 | 0.00856231 | 0.039806224 | 0.007773772 | 0.070821827 |
| HSP90B1 | Epi6 | green | 0.158387359 | 0.060021925 | 0.009700724 | 0.260699155 | 0.050792193 | 0.029389453 | 0.335657054 | -0.061933647 | 0.174986583 |
| RILPL2 | Epi6 | green | 0.050735133 | 0.018910765 | -0.025022966 | 0.052951823 | 0.043627835 | 0.003292491 | 0.154983994 | -0.046641513 | 0.039027481 |
| EBPL | Epi6 | green | 0.080153414 | 0.059769911 | 0.0434296 | 0.077256647 | 0.064461883 | 0.025701361 | 0.077211586 | 0.026996456 | 0.062993433 |
| MBNL2 | Epi6 | green | 0.038350398 | 0.015995367 | -0.013601595 | 1.97E-05 | 0.037591151 | -0.017827879 | 0.057352708 | -0.032918094 | -0.019938212 |
| APEX1 | Epi6 | green | 0.139883803 | 0.031474434 | 0.087728637 | 0.136976279 | 0.142055495 | 0.048003085 | 0.162388582 | 0.011291369 | 0.131626341 |
| NKX2-1 | Epi6 | green | 0.155918568 | 0.097719628 | 0.077145302 | 0.167655819 | 0.112056406 | 0.05167503 | 0.177259293 | -0.000930173 | 0.137903911 |
| TIMM9 | Epi6 | green | 0.06364223 | -0.007175143 | 0.03773706 | 0.04832901 | 0.067038568 | 0.022235267 | 0.081759886 | -0.014145602 | 0.051511417 |
| LTBP2 | Epi6 | green | 0.060317214 | 0.003485152 | 0.022498738 | 0.046524611 | 0.047564414 | 0.022253005 | 0.091028486 | -0.016220628 | 0.057867915 |
| PGF | Epi6 | green | 0.017534015 | -0.017755289 | -0.066798545 | 0.040230254 | 0.000941126 | -0.00673811 | 0.167010676 | -0.087029561 | -0.000377813 |
| DIO2 | Epi6 | green | -0.006457295 | -0.055796966 | -0.104884857 | 0.056040599 | -0.026499954 | -0.019304801 | 0.228658219 | -0.151079095 | 0.012669828 |
| TSHR | Epi6 | green | 0.117725957 | -0.010934678 | -0.00384264 | 0.094512663 | 0.154440909 | 0.028028958 | 0.279145552 | -0.153026946 | 0.137976198 |
| SLC25A29 | Epi6 | green | 0.058139035 | -0.008533662 | 0.012926276 | 0.033432305 | 0.046461831 | 0.009303618 | 0.140223881 | -0.043401661 | 0.050644449 |
| CKB | Epi6 | green | 0.065945269 | 0.079525833 | -0.030000855 | 0.093070879 | 0.048141146 | 0.002339649 | 0.143855038 | -0.019864334 | 0.024488414 |
| CRIP1 | Epi6 | green | 0.043925484 | 0.089943093 | -0.102877044 | 0.090647775 | -0.037060142 | 0.000799119 | 0.198768148 | -0.061559216 | -0.059309616 |
| SORD | Epi6 | green | 0.02508593 | -0.047178922 | -0.130341131 | 0.134804653 | -0.034706624 | -0.007431547 | 0.330630837 | -0.170407955 | 0.046593209 |
| DAPK2 | Epi6 | green | 0.0904582 | -0.073586065 | 0.055278495 | 0.060733715 | 0.163371929 | 0.037697268 | 0.199960022 | -0.096889959 | 0.151121205 |
| ISG20 | Epi6 | green | 0.09374943 | 0.085181906 | 0.011700768 | 0.07215752 | 0.048156657 | 0.023950895 | 0.092970762 | -0.023826997 | 0.048924382 |
| SNHG9 | Epi6 | green | 0.094659962 | -0.013230299 | 0.072471244 | 0.115860262 | 0.068314299 | 0.045998699 | 0.120569017 | 0.003644379 | 0.098851758 |
| LITAF | Epi6 | green | 0.111718336 | 0.045428955 | 0.026082929 | 0.097942637 | 0.075619616 | 0.030416942 | 0.145271082 | -0.001290534 | 0.06243443 |
| MT2A | Epi6 | green | 0.099835285 | 0.090140149 | -0.018587709 | 0.094704433 | 0.002113988 | 0.021618532 | 0.259976782 | -0.062465552 | 0.026192063 |
| MT1E | Epi6 | green | 0.083833225 | 0.025428498 | -0.056834909 | 0.122477455 | 0.019590496 | 0.007986374 | 0.354591122 | -0.148361746 | 0.064098192 |
| MT1F | Epi6 | green | 0.004238821 | -0.052273358 | -0.156713592 | 0.11248939 | -0.062269696 | -0.015286648 | 0.375840983 | -0.195500539 | 0.004599122 |
| MT1G | Epi6 | green | 0.010604072 | -0.018326047 | -0.118500922 | 0.074168418 | -0.07374418 | -0.002611106 | 0.260787405 | -0.124457626 | -0.018756037 |
| MT1X | Epi6 | green | 0.06537776 | 0.002528507 | -0.072361384 | 0.1273857 | -0.026900331 | 0.011747773 | 0.298461613 | -0.087725287 | 0.025583156 |
| ADGRG1 | Epi6 | green | 0.069887298 | 0.050821726 | 0.018336236 | 0.040162224 | 0.043670441 | 0.016377146 | 0.080351752 | 0.001323851 | 0.041214051 |
| TEPP | Epi6 | green | 0.01819379 | -0.032023428 | -0.027771931 | 0.040380656 | 0.054390239 | -0.023615749 | 0.085831056 | -0.051516003 | 0.064957549 |
| WWOX | Epi6 | green | 0.027373171 | -0.033659408 | 0.003038034 | 0.040464496 | 0.038248402 | -0.0207951 | 0.099036114 | -0.026450083 | 0.046751815 |
| GABARAP | Epi6 | green | 0.122772709 | 0.050799104 | 0.053490596 | 0.138156863 | 0.098917514 | 0.051957389 | 0.133289767 | 0.019529927 | 0.094055297 |
| SAT2 | Epi6 | green | 0.095685253 | 0.025734461 | 0.03317085 | 0.106529411 | 0.07325217 | 0.015362321 | 0.137212771 | 0.020415702 | 0.067843083 |
| PMP22 | Epi6 | green | 0.043959572 | 0.028020787 | -0.010655371 | 0.099440517 | 0.003834957 | -0.013500611 | 0.123713795 | 0.007768028 | 0.030181484 |
| CCL4 | Epi6 | green | 0.060455441 | 0.044729665 | -0.041457773 | 0.091811709 | 0.013142738 | 0.034852215 | 0.144720186 | -0.06492147 | 0.017910407 |
| IGFBP4 | Epi6 | green | 0.117647582 | 0.178481081 | -0.026232375 | 0.073339678 | 0.068317276 | 0.044393852 | 0.138269538 | -0.037572045 | -0.022115666 |
| P3H4 | Epi6 | green | 0.046354455 | 0.044433682 | -0.000341642 | 0.06926703 | 0.021078254 | -0.000454926 | 0.044449936 | -0.000552402 | 0.048059106 |
| NT5C3B | Epi6 | green | 0.060000399 | 0.040603744 | -0.006254832 | 0.065150748 | 0.03971966 | 0.004014843 | 0.151666435 | -0.014562689 | 0.038036332 |
| PRR15L | Epi6 | green | 0.059230495 | 0.101029334 | -0.06514495 | 0.103357397 | 0.031216763 | 0.019855703 | 0.180402748 | -0.063249697 | 0.000395702 |
| NME2 | Epi6 | green | 0.081751588 | 0.054079431 | 0.050387994 | 0.063166962 | 0.068051323 | 0.0554351 | 0.001867684 | 0.024246423 | 0.049134204 |
| RPL17 | Epi6 | green | 0.234483096 | 0.045742363 | 0.117073972 | 0.168198472 | 0.32539359 | 0.067484487 | 0.180339097 | -0.012094564 | 0.174550274 |
| TXNL1 | Epi6 | green | 0.080003119 | -0.047294057 | -0.025562181 | 0.182384281 | 0.035205234 | -0.01361635 | 0.286262736 | -0.080165816 | 0.127804326 |
| DSTN | Epi6 | green | 0.280792907 | 0.121241228 | 0.173118306 | 0.311942779 | 0.243317708 | 0.073275261 | 0.305324394 | 0.044348819 | 0.283620756 |
| ID1 | Epi6 | green | 0.093534991 | -0.118175422 | 0.018272589 | 0.119938924 | 0.120668673 | 0.010097729 | 0.393870179 | -0.164697817 | 0.193422928 |
| AHCY | Epi6 | green | 0.091278966 | 0.037825214 | 0.048557343 | 0.084912617 | 0.127616256 | 0.04056774 | 0.091403157 | 0.000347024 | 0.106053844 |
| MAP1LC3A | Epi6 | green | 0.059759082 | -0.004165317 | -0.018368499 | 0.07442632 | 0.043783594 | 0.000758657 | 0.208028188 | -0.069709185 | 0.054841852 |
| MYL9 | Epi6 | green | 0.122837493 | -0.032465351 | 0.049209737 | 0.220642567 | 0.172298687 | 0.027425302 | 0.264167946 | -0.052121112 | 0.239811853 |
| MAFB | Epi6 | green | 0.0471481 | 0.034204353 | -0.05899018 | 0.053466211 | 0.006435054 | 0.001920493 | 0.184048589 | -0.086005822 | -0.003999825 |
| WFDC2 | Epi6 | green | 0.047748493 | 0.075434178 | -0.059184716 | 0.16447998 | 0.086721068 | -0.010257431 | 0.211437805 | -0.101764504 | 0.110143933 |
| CNN2 | Epi6 | green | 0.070628802 | -0.007331778 | 0.024014277 | 0.082458788 | 0.094762242 | 0.008331475 | 0.15116595 | -0.043301208 | 0.098764076 |
| SLC44A2 | Epi6 | green | 0.043720562 | 0.073085056 | -0.013355106 | 0.048950981 | 0.02575808 | 0.015330817 | 0.07265715 | -0.009224042 | 0.008893484 |
| QTRT1 | Epi6 | green | 0.031510202 | 0.013597122 | -0.014524202 | 0.070728913 | -0.009293544 | -0.00426512 | 0.096891318 | -0.024627927 | 0.040135834 |
| FCGBP | Epi6 | green | 0.026562668 | -0.036274235 | -0.043990778 | 0.037318415 | 0.022997532 | -0.019431472 | 0.196006601 | -0.079751221 | 0.030651119 |
| BCAM | Epi6 | green | 0.002160468 | -0.016678756 | -0.133196989 | 0.137808897 | -0.036226982 | -0.011388789 | 0.267300707 | -0.147415152 | 0.045424176 |
| C19orf48 | Epi6 | green | 0.02816666 | -0.01971068 | -0.046500285 | 0.04665451 | 0.025103952 | -0.010434177 | 0.17635534 | -0.082460715 | 0.028047229 |
| CLDND2 | Epi6 | green | 0.00845333 | -0.028562603 | -0.001787472 | 0.025497594 | -0.002402682 | -0.023575918 | 0.055559886 | -0.006893559 | 0.038392271 |
| MYADM | Epi6 | green | 0.091564782 | 0.069360129 | 0.005898413 | 0.04591945 | 0.023635648 | 0.016002212 | 0.125693672 | -0.044899883 | 0.019616347 |
| H1F0 | Epi6 | green | 0.094426947 | 0.061552457 | 0.028447033 | 0.028196392 | 0.108511397 | 0.045876481 | 0.113606607 | -0.036177434 | 0.0533677 |
| MRPS6 | Epi6 | green | 0.190058278 | 0.033889756 | 0.149990106 | 0.237566133 | 0.150661809 | 0.035823299 | 0.21357072 | 0.079279942 | 0.213513673 |
| PCP4 | Epi6 | green | -0.00562042 | -0.050940494 | -0.17071514 | 0.105708862 | -0.049859335 | -0.021285175 | 0.340052189 | -0.205241753 | 0.007754039 |
| TFF3 | Epi6 | green | -0.077414781 | -0.084671392 | -0.215465912 | 0.06549051 | -0.136851257 | -0.057939302 | 0.295711679 | -0.208630581 | -0.051025609 |
| FAM159A | Epi7 | black | 0.073855643 | 0.082336937 | 0.097638482 | 0.069660067 | -0.054770804 | 0.011254017 | -0.077551711 | 0.239981049 | -0.052211045 |
| SGIP1 | Epi7 | black | 0.073253418 | 0.144619793 | 0.078850421 | 0.025241772 | -0.097732552 | 0.033208926 | -0.126766885 | 0.276487631 | -0.108636302 |
| DPYD | Epi7 | black | 0.067624701 | 0.031020568 | 0.094620077 | 0.042367072 | 0.001346104 | -0.003797857 | -0.038894494 | 0.146620747 | 0.015336448 |
| S100A5 | Epi7 | black | 0.089751463 | 0.044814689 | 0.154122653 | 0.085731569 | 0.02885782 | 0.009866743 | -0.04977166 | 0.214938111 | 0.052612668 |
| S100A4 | Epi7 | black | 0.105763336 | 0.094500083 | 0.183522226 | 0.178315208 | 0.032399437 | 0.046626932 | -0.078227096 | 0.30172925 | 0.118607086 |
| S100A13 | Epi7 | black | 0.30894063 | 0.172781285 | 0.358568908 | 0.309568337 | 0.156938968 | 0.097090003 | 0.019016882 | 0.45975334 | 0.194958779 |
| FCER1G | Epi7 | black | 0.105927406 | 0.066953558 | 0.140657046 | 0.073037877 | 0.039196756 | 0.050534451 | -0.019964368 | 0.190821891 | 0.023676293 |
| RXRG | Epi7 | black | 0.095418207 | 0.144121999 | 0.101761567 | 0.033154906 | 0.004053666 | 0.035797566 | -0.105851665 | 0.211890589 | -0.04093756 |
| MPC2 | Epi7 | black | 0.176327632 | 0.153671471 | 0.19054635 | 0.111797822 | 0.002720353 | 0.058459243 | -0.039483162 | 0.331976067 | 0.000692539 |
| LAMB3 | Epi7 | black | 0.085194496 | 0.133926312 | 0.130423829 | 0.083450964 | -0.024115292 | 0.070684923 | -0.099316151 | 0.299762886 | -0.019368091 |
| G0S2 | Epi7 | black | 0.111745468 | 0.179510713 | 0.171770847 | 0.070764132 | -0.098299374 | 0.040497446 | -0.187182805 | 0.444702067 | -0.110334698 |
| ST3GAL5 | Epi7 | black | 0.173901106 | 0.130553085 | 0.206826512 | 0.092407663 | 0.066555083 | 0.05294525 | -0.05865314 | 0.259629948 | 0.06175869 |
| DBI | Epi7 | black | 0.233523703 | 0.303188108 | 0.230339621 | 0.251629526 | 0.079808023 | 0.069443001 | -0.062771169 | 0.347412776 | 0.10727413 |
| SUMO1 | Epi7 | black | 0.232892017 | 0.144162733 | 0.243553906 | 0.188320292 | 0.127317559 | 0.090060844 | 0.014117737 | 0.279859298 | 0.114030978 |
| CAMK1 | Epi7 | black | 0.08303163 | 0.05477532 | 0.12182051 | 0.080404171 | -0.016847256 | -0.000734779 | -0.052472149 | 0.239417204 | 0.015353487 |
| RHOA | Epi7 | black | 0.286141455 | 0.209668199 | 0.260303314 | 0.19880941 | 0.167641607 | 0.081988182 | 0.030645269 | 0.252800863 | 0.161039999 |
| PTPRG | Epi7 | black | 0.11850099 | 0.045289541 | 0.151040643 | 0.051532555 | -0.000524915 | 0.047249113 | -0.052759632 | 0.243884917 | 0.02532984 |
| NUDT16 | Epi7 | black | 0.098668462 | 0.072703339 | 0.172013389 | 0.119074283 | 0.026253262 | 0.025514362 | -0.076829499 | 0.254800012 | 0.083671068 |
| PLOD2 | Epi7 | black | 0.141772317 | 0.101761695 | 0.154749787 | 0.140810387 | -0.004862989 | 0.045121888 | -0.021325588 | 0.22035812 | 0.060570263 |
| GALNT7 | Epi7 | black | 0.104522451 | 0.097057539 | 0.136154023 | 0.072011463 | 0.028700881 | 0.057637088 | -0.044517159 | 0.192124659 | 0.048703869 |
| SLC26A2 | Epi7 | black | 0.108549897 | 0.041922941 | 0.141773244 | 0.057894388 | 0.010129269 | 0.044393578 | -0.038866828 | 0.204755142 | 0.030833221 |
| RNF145 | Epi7 | black | 0.12482086 | 0.128978073 | 0.131064242 | 0.051089067 | 0.01691676 | 0.03611205 | -0.057040498 | 0.221922933 | -0.027284632 |
| RHBDD2 | Epi7 | black | 0.155537152 | 0.128756175 | 0.145449003 | 0.186205707 | 0.080694356 | 0.063661503 | 0.023802522 | 0.19061402 | 0.110314465 |
| HS6ST2 | Epi7 | black | 0.109217513 | 0.164687463 | 0.093547275 | 0.079679949 | -0.038777534 | 0.043951326 | -0.081926662 | 0.244310644 | -0.034329474 |
| PDLIM2 | Epi7 | black | 0.076607823 | 0.115832475 | 0.061488959 | 0.038352211 | 0.006078321 | 0.04702321 | -0.018240478 | 0.108932665 | -0.020666336 |
| HAS2 | Epi7 | black | 0.044380169 | 0.017283626 | 0.100522581 | 0.024581659 | -0.00872218 | 0.033092422 | -0.06221805 | 0.151778627 | 0.018155817 |
| LY6E | Epi7 | black | 0.261339742 | 0.399983811 | 0.221070244 | 0.18816139 | 0.058921494 | 0.093569711 | -0.055076763 | 0.35053933 | 0.03327165 |
| MSMP | Epi7 | black | 0.079284213 | 0.067372371 | 0.111325115 | 0.107335703 | -0.103585832 | 0.047722557 | -0.073254429 | 0.335905729 | -0.059632144 |
| SUSD3 | Epi7 | black | 0.021928592 | 0.058844622 | 0.071417658 | 0.047375406 | -0.041397753 | 0.006723573 | -0.078575777 | 0.159204571 | -0.011001726 |
| CARD19 | Epi7 | black | 0.108189543 | 0.132093566 | 0.120422476 | 0.098276194 | 0.0066481 | 0.071155902 | -0.035289664 | 0.202717106 | 0.007338976 |
| NINJ1 | Epi7 | black | 0.181839186 | 0.227138999 | 0.211513083 | 0.168780247 | 0.076136583 | 0.06313338 | -0.076425073 | 0.321081787 | 0.060750299 |
| TNC | Epi7 | black | 0.123083701 | 0.157272107 | 0.117923508 | 0.082872699 | -0.014559719 | 0.019513753 | -0.053736547 | 0.225563369 | -0.015408284 |
| ENG | Epi7 | black | 0.090147253 | 0.121130292 | 0.137838591 | 0.120234381 | -0.043727945 | 0.018260782 | -0.119437595 | 0.308300728 | 0.001575854 |
| AK1 | Epi7 | black | 0.20274801 | 0.293374506 | 0.221635275 | 0.210162219 | -0.015241258 | 0.052176488 | -0.167460421 | 0.480180844 | -0.003387287 |
| C9orf16 | Epi7 | black | 0.253208048 | 0.20911904 | 0.255421119 | 0.088212704 | 0.149606016 | 0.082617186 | -0.004407945 | 0.264669128 | 0.058330361 |
| NPDC1 | Epi7 | black | 0.133768618 | 0.158233087 | 0.111293534 | 0.104733147 | 0.012233446 | 0.05341415 | -0.044849609 | 0.227231585 | 0.002228006 |
| ADM | Epi7 | black | 0.101174432 | 0.137529493 | 0.126218481 | 0.078122514 | -0.015620666 | 0.004797961 | -0.084957562 | 0.214247485 | 0.008665582 |
| NEAT1 | Epi7 | black | 0.355712352 | 0.299547798 | 0.296842354 | 0.095223338 | 0.100836051 | 0.136397289 | -0.0279901 | 0.349987395 | 0.029310198 |
| HMBS | Epi7 | black | 0.109020903 | 0.079253358 | 0.234461529 | 0.087527314 | -0.013550305 | 0.024375313 | -0.16310066 | 0.357147795 | 0.036901762 |
| NTM | Epi7 | black | 0.044632343 | 0.064737048 | 0.101985355 | 0.038064357 | -0.062534381 | 0.051863059 | -0.14086424 | 0.263229698 | -0.040676419 |
| DDIT4 | Epi7 | black | 0.085540501 | 0.067924375 | 0.096024037 | 0.091508345 | 0.01647176 | 0.039214372 | -0.035749579 | 0.156297491 | 0.042076382 |
| PLAU | Epi7 | black | 0.242324701 | 0.427974524 | 0.213145356 | 0.297069496 | -0.033651663 | 0.062087646 | -0.045968059 | 0.348235169 | 0.083714201 |
| SLIT1 | Epi7 | black | 0.047878171 | 0.040606201 | 0.149462843 | 0.039999532 | -0.097999938 | 0.019001152 | -0.176158273 | 0.361456103 | -0.045751077 |
| SORCS1 | Epi7 | black | 0.071122937 | 0.024989937 | 0.132541715 | 0.042399267 | 0.0243042 | 0.046224597 | -0.042044268 | 0.181405795 | 0.041352962 |
| AC079630.2 | Epi7 | black | 0.13050531 | 0.086962701 | 0.163757082 | 0.052851235 | 0.080564821 | 0.049143685 | -0.067513192 | 0.206138552 | 0.036431709 |
| YAF2 | Epi7 | black | 0.180775473 | 0.138849992 | 0.23937138 | 0.124161323 | 0.029434977 | 0.068208368 | -0.0983938 | 0.366375193 | 0.050159487 |
| RP11-547C5.1 | Epi7 | black | 0.072455817 | 0.015554568 | 0.140905562 | 0.08251344 | -0.04252808 | 0.013286007 | -0.07406055 | 0.265880712 | 0.027550953 |
| NELL2 | Epi7 | black | 0.125025517 | 0.028499945 | 0.220725369 | 0.118514075 | 0.075138151 | 0.059978438 | -0.055098769 | 0.242989119 | 0.137788911 |
| ANO6 | Epi7 | black | 0.083853877 | -0.009051615 | 0.140402395 | 0.053489982 | -0.002576841 | 0.051210543 | -0.061140609 | 0.228241155 | 0.034710546 |
| RP11-983P16.4 | Epi7 | black | 0.061991137 | 0.052166045 | 0.082257553 | 0.009224954 | 0.02059967 | 0.032568381 | -0.041988381 | 0.136686007 | -0.017402426 |
| IGFBP6 | Epi7 | black | 0.237350503 | 0.466018864 | 0.15101943 | 0.234184213 | -0.080318218 | 0.104377417 | -0.13304674 | 0.493684243 | -0.094563366 |
| WIF1 | Epi7 | black | 0.048604338 | 0.005903401 | 0.122886989 | 0.115306355 | -0.025868126 | -0.000903834 | -0.077132021 | 0.179155501 | 0.080200848 |
| HMGA2 | Epi7 | black | 0.160611508 | 0.251075181 | 0.137665633 | 0.041654706 | -0.02113592 | 0.075622107 | -0.130792425 | 0.297403899 | -0.057294825 |
| DUSP6 | Epi7 | black | 0.293800985 | 0.228263017 | 0.30261699 | 0.126372838 | 0.131502813 | 0.146359339 | -0.02900068 | 0.29816133 | 0.121833817 |
| NUDT4 | Epi7 | black | 0.185816157 | 0.124745377 | 0.205318643 | 0.068724088 | 0.073726722 | 0.107896904 | -0.040511424 | 0.220282335 | 0.077183073 |
| ISCU | Epi7 | black | 0.292622846 | 0.210358278 | 0.307435652 | 0.25285691 | 0.142092593 | 0.090228259 | -0.015027078 | 0.381830777 | 0.144109064 |
| TESC | Epi7 | black | 0.158221161 | 0.06834986 | 0.296746804 | 0.207116874 | 0.039455809 | 0.04330414 | -0.120693666 | 0.400964101 | 0.167090495 |
| DYNLL1 | Epi7 | black | 0.331918251 | 0.271302763 | 0.338008806 | 0.249951708 | 0.162359128 | 0.109137783 | 0.019616889 | 0.370434443 | 0.17216654 |
| TSC22D1 | Epi7 | black | 0.268784476 | 0.046623732 | 0.380809159 | 0.233219471 | 0.13043047 | 0.093108166 | -0.091058679 | 0.406519805 | 0.224893614 |
| TRDC | Epi7 | black | 0.220598677 | 0.287086965 | 0.252103785 | 0.155788316 | 0.009952251 | 0.088024715 | -0.130670992 | 0.392783869 | 0.034423819 |
| NOVA1 | Epi7 | black | 0.166769391 | 0.238731119 | 0.253577352 | 0.10001169 | -0.086239229 | 0.067823208 | -0.21651347 | 0.501052831 | -0.08251273 |
| CALM1 | Epi7 | black | 0.30375417 | 0.211885028 | 0.318741683 | 0.258555639 | 0.076620877 | 0.080718109 | -0.00575908 | 0.446896449 | 0.134247217 |
| TMEM87A | Epi7 | black | 0.197617657 | 0.154268082 | 0.211948757 | 0.133508434 | 0.098719105 | 0.057741137 | -0.027732225 | 0.240557617 | 0.117469658 |
| MAP2K1 | Epi7 | black | 0.129800266 | 0.019107797 | 0.235020838 | 0.090376601 | 0.041345283 | 0.053830523 | -0.082680029 | 0.276027792 | 0.092402784 |
| TSPAN3 | Epi7 | black | 0.207031456 | 0.150129717 | 0.208064822 | 0.169904417 | 0.080963766 | 0.079062495 | -0.000328182 | 0.266256333 | 0.119547997 |
| HMOX2 | Epi7 | black | 0.080187481 | 0.118217004 | 0.111731855 | 0.089990272 | -0.07059419 | 0.011573946 | -0.099576841 | 0.309650242 | -0.040269821 |
| KIF22 | Epi7 | black | 0.161406703 | 0.065838783 | 0.236589438 | 0.130008571 | 0.068247545 | 0.072896466 | -0.038610597 | 0.293210109 | 0.09032521 |
| AC026471.6 | Epi7 | black | 0.012159204 | 0.039907607 | 0.06091954 | 0.003389524 | -0.032322301 | 0.045002845 | -0.081297567 | 0.149573416 | -0.02802981 |
| MYH10 | Epi7 | black | 0.116687633 | 0.135841887 | 0.134553023 | 0.077620822 | -0.002078773 | 0.047319287 | -0.080392757 | 0.239277412 | 0.017884369 |
| CPD | Epi7 | black | 0.135023171 | 0.179296743 | 0.159102523 | 0.070543509 | -0.015879503 | 0.027335673 | -0.06052319 | 0.286292528 | -0.026085801 |
| HEXIM1 | Epi7 | black | 0.093418159 | 0.09832643 | 0.072263526 | 0.054745674 | 0.009599454 | 0.072990628 | -0.002871436 | 0.118651517 | -0.008793671 |
| TMEM100 | Epi7 | black | 0.116188639 | 0.121050049 | 0.120531231 | 0.091576337 | -0.017413541 | 0.034215818 | -0.057043087 | 0.243261456 | -0.0203338 |
| PRR29 | Epi7 | black | 0.050369677 | 0.127300557 | 0.071282979 | 0.024677263 | -0.052565291 | 0.011815416 | -0.107077288 | 0.248599303 | -0.061173926 |
| PRKAR1A | Epi7 | black | 0.195667625 | 0.141948197 | 0.186927823 | 0.150136809 | 0.073599804 | 0.076471283 | 0.019260084 | 0.231488267 | 0.079614068 |
| GPRC5C | Epi7 | black | 0.119540568 | 0.132828468 | 0.134237964 | 0.182164121 | -0.018166976 | 0.039303691 | -0.042112718 | 0.279590861 | 0.049468401 |
| TTR | Epi7 | black | 0.017651629 | 0.004612669 | 0.064360965 | 0.076893636 | -0.023664012 | -0.009150539 | -0.031673771 | 0.107074301 | 0.055659374 |
| B4GALT6 | Epi7 | black | 0.056006222 | 0.039811528 | 0.133162927 | 0.027999311 | -0.031906023 | 0.034048773 | -0.092702798 | 0.19582166 | -0.001235486 |
| YWHAB | Epi7 | black | 0.243457366 | 0.154872532 | 0.2346983 | 0.189695352 | 0.13149919 | 0.096132838 | 0.048322164 | 0.232845491 | 0.149695787 |
| EDN3 | Epi7 | black | 0.028069795 | 0.027329969 | 0.02665707 | 0.024822732 | -0.034499052 | 0.017128096 | -0.033891906 | 0.109275406 | -0.045109807 |
| ADGRE5 | Epi7 | black | 0.069322549 | 0.090768527 | 0.080715888 | 0.088173373 | 0.001308531 | 0.004188152 | -0.023347939 | 0.123200645 | 0.028276507 |
| SCN1B | Epi7 | black | 0.113974696 | 0.15558058 | 0.1653727 | 0.084937292 | -0.00609819 | 0.042760646 | -0.07687245 | 0.26260113 | 0.00657329 |
| FXYD5 | Epi7 | black | 0.171821833 | 0.319674698 | 0.185303999 | 0.240334365 | -0.010966259 | 0.057705275 | -0.126247701 | 0.397262843 | 0.033699033 |
| POLR2I | Epi7 | black | 0.196790859 | 0.14208562 | 0.200306414 | 0.194217287 | 0.132852704 | 0.035835584 | 0.047386533 | 0.21784417 | 0.141377836 |
| APOE | Epi7 | black | 0.110675869 | 0.024703939 | 0.192326132 | 0.3241576 | -0.09870308 | 0.039373018 | -0.094432454 | 0.485712321 | 0.118577952 |
| APOC1 | Epi7 | black | 0.075854695 | 0.027057322 | 0.215922127 | 0.111817353 | -0.141436 | 0.034808998 | -0.209308416 | 0.554174475 | -0.060237037 |
| SEPW1 | Epi7 | black | 0.318331838 | 0.119644558 | 0.374504677 | 0.176466807 | 0.214059168 | 0.101606045 | 0.017963619 | 0.388808386 | 0.180712251 |
| EMP3 | Epi7 | black | 0.168525935 | 0.224970776 | 0.264240742 | 0.344590658 | -0.038590072 | 0.070329343 | -0.122813955 | 0.457401679 | 0.179358939 |
| USP18 | Epi7 | black | 0.116771012 | 0.08356801 | 0.17909314 | 0.080250498 | -0.06165097 | 0.043631318 | -0.10998314 | 0.385705837 | -0.019309981 |
| DGCR6 | Epi7 | black | 0.078067355 | 0.044300972 | 0.080193164 | 0.074516336 | -0.025782821 | 0.037713314 | -0.043817382 | 0.252400166 | -0.024795244 |
| TPST2 | Epi7 | black | 0.108451171 | 0.077165472 | 0.082983494 | 0.115899315 | 0.001330365 | 0.041501322 | 0.014192903 | 0.166056591 | 0.026760212 |
| LGALS1 | Epi7 | black | 0.328774709 | 0.390494267 | 0.407805844 | 0.29293939 | 0.162727953 | 0.117723468 | -0.102627159 | 0.467558563 | 0.203865193 |
| CSTB | Epi7 | black | 0.26002371 | 0.217687325 | 0.254068346 | 0.279600825 | 0.167385687 | 0.083575307 | 0.07621464 | 0.27312902 | 0.19665413 |
| AC007325.4 | Epi7 | black | 0.100418135 | 0.073201951 | 0.143818853 | 0.1060461 | -0.076278846 | 0.029283797 | -0.079485915 | 0.383269182 | -0.043489105 |
| OLFML3 | Epi8 | pink | 0.056044408 | -0.050519939 | 0.092941244 | 0.054635689 | 0.096224189 | 0.001506497 | 0.048195785 | -0.002789449 | 0.204466919 |
| TXNIP | Epi8 | pink | 0.171265429 | -0.039328678 | 0.198700755 | 0.192054743 | 0.139451333 | 0.052111531 | 0.098556509 | 0.121877344 | 0.261037247 |
| SLC39A10 | Epi8 | pink | 0.141687057 | -0.02612673 | 0.154977529 | 0.102497048 | 0.123150084 | 0.066264546 | 0.103215826 | 0.056437386 | 0.220914278 |
| SSR3 | Epi8 | pink | 0.125217311 | -0.018764988 | 0.127635976 | 0.159657677 | 0.183242729 | 0.081834872 | 0.138175527 | -0.020335397 | 0.281729101 |
| SOD3 | Epi8 | pink | 0.047594381 | -0.122330759 | 0.032929901 | 0.151698446 | 0.054510813 | 0.002077314 | 0.203996971 | -0.040173831 | 0.225369918 |
| AGA | Epi8 | pink | 0.071920756 | -0.022186795 | 0.081300993 | 0.147108958 | 0.075175804 | 0.010298171 | 0.097056564 | 0.002475519 | 0.205508317 |
| SLC35B3 | Epi8 | pink | 0.098199531 | -0.014213158 | 0.075881534 | 0.081918233 | 0.078527313 | 0.037873436 | 0.063296683 | 0.016181943 | 0.146523985 |
| HIST1H4C | Epi8 | pink | 0.149719616 | -0.030514889 | 0.188379087 | 0.077730506 | 0.154392042 | 0.072199252 | 0.083106954 | 0.070414507 | 0.234048761 |
| TUBB | Epi8 | pink | 0.192193804 | 0.008253009 | 0.206947291 | 0.202127101 | 0.218661813 | 0.060781445 | 0.134917043 | 0.059140721 | 0.336799671 |
| GJA1 | Epi8 | pink | 0.054972401 | -0.030404669 | 0.035419003 | 0.061197656 | 0.101687374 | 0.01990588 | 0.084774968 | -0.087340632 | 0.161157323 |
| AGR2 | Epi8 | pink | 0.098652925 | -0.037989491 | 0.08179568 | 0.169650879 | 0.175493369 | 0.03999552 | 0.13999052 | -0.060805814 | 0.291615123 |
| NPY | Epi8 | pink | 0.041820061 | -0.051907571 | 0.097840733 | 0.03375931 | 0.099407522 | 0.008388095 | 0.020951487 | -0.032981889 | 0.16760447 |
| SEC61G | Epi8 | pink | 0.285363773 | 0.066508115 | 0.30617901 | 0.265735284 | 0.289329432 | 0.091944685 | 0.169319688 | 0.125558678 | 0.403273193 |
| SYPL1 | Epi8 | pink | 0.121035064 | 0.028470329 | 0.104514182 | 0.278052684 | 0.128476649 | 0.014716648 | 0.147115358 | -0.003048053 | 0.342805767 |
| NDUFB2 | Epi8 | pink | 0.2833427 | 0.032568673 | 0.329994967 | 0.27596735 | 0.288832606 | 0.087425639 | 0.185855655 | 0.120981058 | 0.432148419 |
| MAGED1 | Epi8 | pink | 0.073690971 | -0.010055383 | 0.071828899 | 0.089727449 | 0.064595773 | 0.006573517 | 0.077857649 | 0.007130711 | 0.130482193 |
| ITM2A | Epi8 | pink | 0.031067463 | -0.101410229 | 0.05780337 | 0.187045209 | 0.130236205 | -0.001153804 | 0.171037317 | -0.086213894 | 0.289724227 |
| LDOC1 | Epi8 | pink | 0.081065594 | -0.035368339 | 0.099359846 | 0.107279937 | 0.104263007 | 0.00970342 | 0.077407128 | 0.007835588 | 0.182753954 |
| ASAH1 | Epi8 | pink | 0.195537204 | -0.039181616 | 0.131940893 | 0.375493994 | 0.172776811 | 0.036326954 | 0.309171547 | -0.000126367 | 0.424093836 |
| SDC2 | Epi8 | pink | 0.177132219 | -0.022181696 | 0.124150692 | 0.258512889 | 0.1046599 | 0.044728331 | 0.273765385 | -0.013618848 | 0.326958599 |
| CPQ | Epi8 | pink | 0.092535806 | -0.028652752 | 0.056784345 | 0.281623573 | 0.057595597 | 0.014601166 | 0.210365024 | -0.028468696 | 0.310350984 |
| CLTA | Epi8 | pink | 0.195768447 | 0.000846977 | 0.274703436 | 0.199390473 | 0.254368395 | 0.054198537 | 0.048632029 | 0.139799098 | 0.351687277 |
| PCSK5 | Epi8 | pink | 0.06937476 | -0.045738425 | 0.13490826 | 0.04682273 | 0.086331468 | 0.014672088 | -0.002720664 | 0.048823147 | 0.181891749 |
| CTNNAL1 | Epi8 | pink | 0.14443444 | -0.02561049 | 0.155729487 | 0.145715581 | 0.099771206 | 0.072067768 | 0.096182805 | 0.046393098 | 0.263936671 |
| PRDX5 | Epi8 | pink | 0.345137171 | 0.155043579 | 0.409470127 | 0.347516177 | 0.333242406 | 0.115870119 | 0.116504694 | 0.222257221 | 0.456157153 |
| DRAP1 | Epi8 | pink | 0.189733163 | 0.041418329 | 0.21308106 | 0.171204326 | 0.199347086 | 0.028127224 | 0.094078693 | 0.067993541 | 0.285946798 |
| CCND1 | Epi8 | pink | 0.184022289 | 0.01960419 | 0.231929063 | 0.126322806 | 0.182556154 | 0.067670424 | 0.054092872 | 0.109317905 | 0.273160531 |
| PTS | Epi8 | pink | 0.132059209 | 0.042327645 | 0.183736394 | 0.149616662 | 0.094072572 | 0.056793796 | 0.028124429 | 0.121761908 | 0.210130153 |
| RP11-356J5.12 | Epi8 | pink | 0.104697254 | -0.070608427 | 0.191619489 | 0.043282685 | 0.169793261 | 0.045663022 | 0.030030834 | 0.036394194 | 0.230711425 |
| APLP2 | Epi8 | pink | 0.270232801 | 0.051400562 | 0.223144586 | 0.28105281 | 0.238998166 | 0.060217184 | 0.243126582 | 0.001389576 | 0.440350137 |
| ENTPD1 | Epi8 | pink | 0.082537307 | -0.041757533 | 0.123727079 | 0.054529156 | 0.128813143 | 0.065273057 | 0.049693473 | -0.009299088 | 0.18671065 |
| NPM3 | Epi8 | pink | 0.136440529 | 0.017978839 | 0.160781396 | 0.100183593 | 0.178423216 | 0.023535285 | 0.061892522 | 0.030806866 | 0.208574746 |
| SLC25A3 | Epi8 | pink | 0.182576714 | 0.031387999 | 0.161370524 | 0.193418994 | 0.240604111 | 0.040249907 | 0.170442655 | 0.023037407 | 0.277032042 |
| LECT1 | Epi8 | pink | 0.064493863 | -0.050721645 | 0.122612533 | 0.094936902 | 0.104327386 | 0.001803246 | 0.050494586 | -0.019745679 | 0.194570895 |
| NPC2 | Epi8 | pink | 0.351468109 | 0.120197468 | 0.392153008 | 0.40730253 | 0.42118655 | 0.110165469 | 0.172180908 | 0.151317409 | 0.592776024 |
| NDUFB1 | Epi8 | pink | 0.255156266 | 0.028086903 | 0.298167996 | 0.242977632 | 0.225296883 | 0.057167777 | 0.149279559 | 0.146986392 | 0.353539779 |
| DUOXA2 | Epi8 | pink | 0.04351042 | -0.075438514 | 0.066732435 | 0.112781574 | 0.049657985 | 0.009640021 | 0.107989988 | -0.015391907 | 0.185801121 |
| EID1 | Epi8 | pink | 0.246504581 | 0.052727048 | 0.234223928 | 0.288378756 | 0.193051107 | 0.069889302 | 0.153876387 | 0.142830988 | 0.364463154 |
| SNX1 | Epi8 | pink | 0.137364791 | 0.03709126 | 0.118080665 | 0.149683445 | 0.12265345 | 0.030116841 | 0.144888183 | -0.014729893 | 0.23397194 |
| TNFRSF12A | Epi8 | pink | 0.201227834 | 0.059800674 | 0.259084283 | 0.147077435 | 0.185976193 | 0.050298514 | 0.066565575 | 0.078971147 | 0.308668643 |
| CDH2 | Epi8 | pink | 0.061091086 | -0.075231729 | 0.121731132 | 0.049740637 | 0.087722382 | 0.02126071 | 0.010326597 | 0.048483406 | 0.15768522 |
| IER3IP1 | Epi8 | pink | 0.124903092 | 0.006218198 | 0.153552339 | 0.115766717 | 0.132098451 | 0.058123158 | 0.034340507 | 0.068853197 | 0.234311189 |
| SMOX | Epi8 | pink | 0.042935278 | -0.034150298 | 0.121525051 | 0.082349775 | 0.069917522 | 0.004839025 | 0.006081548 | 0.06198925 | 0.176012704 |
| APMAP | Epi8 | pink | 0.083661491 | -0.01520582 | 0.039995707 | 0.177146675 | 0.079345387 | 0.011417815 | 0.171000255 | -0.055061094 | 0.208194336 |
| EIF3G | Epi8 | pink | 0.148376462 | -0.000402885 | 0.171889158 | 0.166404076 | 0.194793671 | 0.053699325 | 0.082154099 | 0.070950101 | 0.260188302 |
| DMKN | Epi8 | pink | 0.139711515 | -0.021899198 | 0.15605544 | 0.127089935 | 0.184053514 | 0.034174082 | 0.113571238 | 0.000304618 | 0.276284318 |
| SPINT2 | Epi8 | pink | 0.356918879 | 0.106431133 | 0.386455679 | 0.402092627 | 0.274515585 | 0.093363244 | 0.162084136 | 0.22711442 | 0.52487784 |
| EIF3K | Epi8 | pink | 0.172123369 | -0.009462539 | 0.198769443 | 0.167479332 | 0.231790944 | 0.04508227 | 0.109934817 | 0.079231053 | 0.283187414 |
| MRPL40 | Epi8 | pink | 0.12129339 | -0.011189802 | 0.164995346 | 0.173917144 | 0.134502057 | 0.022024106 | 0.084875429 | 0.043903394 | 0.278349786 |
| APP | Epi8 | pink | 0.140350079 | -0.062179125 | 0.231743406 | 0.142059046 | 0.178548743 | 0.025624997 | 0.102966348 | -0.008412456 | 0.354582194 |

**Additional table2**

| gene | p_val | avg_log2FC | pct.1 | pct.2 | p_val_adj | group1 | group2 | test_group_number | test_group |
| --- | --- | --- | --- | --- | --- | --- | --- | --- | --- |
| CAMK2N1 | 8.73E-140 | 1.028622755 | 0.801 | 0.356 | 6.82E-136 | T-M | others | 2 | T;T-M |
| ATP5E | 1.96E-131 | 0.640118036 | 0.999 | 0.99 | 1.53E-127 | T-M | others | 2 | T;T-M |
| TMSB4X | 3.81E-111 | 1.025936624 | 0.999 | 0.997 | 2.97E-107 | T-M | others | 2 | T;T-M |
| LGALS1 | 1.36E-96 | 0.769618646 | 0.98 | 0.845 | 1.07E-92 | T-M | others | 2 | T;T-M |
| RPL28 | 2.80E-93 | 0.416808632 | 1 | 1 | 2.18E-89 | T-M | others | 2 | T;T-M |
| C4orf48 | 1.21E-85 | 0.622880621 | 0.957 | 0.744 | 9.45E-82 | T-M | others | 2 | T;T-M |
| KCTD12 | 1.34E-78 | 0.766786023 | 0.587 | 0.205 | 1.05E-74 | T-M | others | 2 | T;T-M |
| PRSS23 | 3.90E-78 | 0.64247761 | 0.944 | 0.691 | 3.05E-74 | T-M | others | 2 | T;T-M |
| NMB | 1.61E-73 | 0.767851149 | 0.88 | 0.632 | 1.26E-69 | T-M | others | 2 | T;T-M |
| POLR2L | 1.67E-71 | 0.518616603 | 0.985 | 0.941 | 1.31E-67 | T-M | others | 2 | T;T-M |
| MACROD2 | 6.24E-69 | 0.62565025 | 0.658 | 0.314 | 4.88E-65 | T-M | others | 2 | T;T-M |
| ECM1 | 1.26E-67 | 0.695930332 | 0.739 | 0.412 | 9.82E-64 | T-M | others | 2 | T;T-M |
| AC090498.1 | 3.22E-67 | 0.535711288 | 0.517 | 0.186 | 2.52E-63 | T-M | others | 2 | T;T-M |
| APOC1 | 9.90E-61 | 0.814741006 | 0.854 | 0.599 | 7.74E-57 | T-M | others | 2 | T;T-M |
| GPX1 | 1.09E-46 | 0.411789636 | 0.95 | 0.854 | 8.48E-43 | T-M | others | 2 | T;T-M |
| ZCCHC12 | 1.24E-45 | 0.556270403 | 0.818 | 0.577 | 9.70E-42 | T-M | others | 2 | T;T-M |
| EMP3 | 1.97E-44 | 0.47931951 | 0.733 | 0.481 | 1.54E-40 | T-M | others | 2 | T;T-M |
| TRDC | 1.07E-43 | 0.532878886 | 0.454 | 0.184 | 8.36E-40 | T-M | others | 2 | T;T-M |
| PRR15 | 1.26E-39 | 0.444269991 | 0.619 | 0.356 | 9.84E-36 | T-M | others | 2 | T;T-M |
| S100A10 | 4.31E-37 | 0.451887559 | 0.786 | 0.593 | 3.36E-33 | T-M | others | 2 | T;T-M |
| PLAU | 9.12E-35 | 0.507690132 | 0.483 | 0.243 | 7.12E-31 | T-M | others | 2 | T;T-M |
| HMBS | 3.02E-34 | 0.42107498 | 0.392 | 0.168 | 2.36E-30 | T-M | others | 2 | T;T-M |
| MSMP | 9.40E-32 | 1.312793364 | 0.382 | 0.166 | 7.34E-28 | T-M | others | 2 | T;T-M |
| PDLIM1 | 2.63E-31 | 0.46119627 | 0.841 | 0.724 | 2.05E-27 | T-M | others | 2 | T;T-M |
| TIMP1 | 6.47E-28 | 0.401095289 | 0.958 | 0.829 | 5.06E-24 | T-M | others | 2 | T;T-M |
| FN1 | 1.19E-27 | 0.649895413 | 0.583 | 0.392 | 9.30E-24 | T-M | others | 2 | T;T-M |
| G0S2 | 1.86E-14 | 0.514823622 | 0.44 | 0.296 | 1.45E-10 | T-M | others | 2 | T;T-M |
| S100A4 | 5.76E-12 | 0.425336485 | 0.751 | 0.674 | 4.50E-08 | T-M | others | 2 | T;T-M |

**Additional table3**

| Gene | logFC | AveExpr | t | P.Value | adj.P.Val | B |
| --- | --- | --- | --- | --- | --- | --- |
| S100A10 | 2.36910464 | 8.3304831 | 29.32243462 | 3.45E-111 | 4.75E-107 | 243.064875 |
| KCNN4 | 3.272032835 | 4.62660006 | 23.98878882 | 1.68E-85 | 1.16E-81 | 184.2196098 |
| RUNX1 | 1.584521947 | 3.46006389 | 22.81374054 | 9.37E-80 | 4.29E-76 | 171.0342558 |
| PLAUR | 2.091740094 | 4.00125492 | 22.39473076 | 1.05E-77 | 3.62E-74 | 166.3650912 |
| CDH6 | 1.831938019 | 3.405657042 | 22.11163934 | 2.56E-76 | 7.03E-73 | 163.1585934 |
| BCL2L1 | 0.883700163 | 6.774379443 | 21.70732448 | 2.44E-74 | 5.58E-71 | 158.6793423 |
| NFE2L3 | 2.248651199 | 3.783299095 | 21.60634021 | 7.60E-74 | 1.49E-70 | 157.5162375 |
| SLPI | 3.429353974 | 7.690333453 | 21.00397112 | 6.69E-71 | 1.15E-67 | 150.7952496 |
| ANXA2 | 1.430428865 | 7.383151314 | 20.86271538 | 3.27E-70 | 5.00E-67 | 149.2155176 |
| SFN | 3.143385624 | 5.017578232 | 20.61594366 | 5.24E-69 | 7.20E-66 | 146.454503 |
| B3GNT3 | 3.562767009 | 4.396014128 | 20.53983244 | 1.23E-68 | 1.54E-65 | 145.5886036 |
| TMPRSS4 | 3.26610049 | 3.411815876 | 20.43324717 | 4.08E-68 | 4.32E-65 | 144.3474308 |
| LDLR | 1.83971884 | 3.317153279 | 20.43300683 | 4.09E-68 | 4.32E-65 | 144.3827276 |
| ITGB4 | 1.652445052 | 5.480906224 | 20.42499021 | 4.47E-68 | 4.39E-65 | 144.3195652 |
| ALOX5 | 2.989669064 | 5.094225228 | 20.19875001 | 5.66E-67 | 5.18E-64 | 141.7960943 |
| CD276 | 0.771074196 | 5.526953855 | 20.18181372 | 6.84E-67 | 5.87E-64 | 141.6029417 |
| DST | 1.292563393 | 2.804616768 | 19.96812225 | 7.49E-66 | 6.06E-63 | 139.1774334 |
| PELI1 | 1.55490095 | 4.77606177 | 19.74970381 | 8.63E-65 | 6.59E-62 | 136.7899674 |
| EVA1A | 2.085645982 | 4.185975666 | 19.6233145 | 3.54E-64 | 2.56E-61 | 135.3877541 |
| STAC | 1.889718793 | 2.026171017 | 19.52726082 | 1.04E-63 | 7.12E-61 | 134.1571508 |
| MCUB | 1.392896039 | 3.685687204 | 19.44067695 | 2.72E-63 | 1.78E-60 | 133.3583323 |
| CD55 | 2.036804638 | 6.988238331 | 19.43288897 | 2.97E-63 | 1.86E-60 | 133.267166 |
| PLAU | 2.372413006 | 6.453056937 | 19.33089308 | 9.27E-63 | 5.54E-60 | 132.1336308 |
| CFH | 2.026143849 | 5.109112453 | 19.25303414 | 2.21E-62 | 1.26E-59 | 131.2706947 |
| DRAM1 | 1.218500244 | 4.542660738 | 19.07036422 | 1.69E-61 | 9.28E-59 | 129.2473877 |
| CYP2S1 | 2.285642382 | 3.797539822 | 18.92334283 | 8.66E-61 | 4.57E-58 | 127.6276498 |
| CREB5 | 1.43317219 | 2.047203257 | 18.8363214 | 2.28E-60 | 1.16E-57 | 126.5561885 |
| NBL1 | 1.728709988 | 5.940154112 | 18.82849216 | 2.48E-60 | 1.22E-57 | 126.5662569 |
| MYO1G | 2.32084255 | 3.283729198 | 18.80646243 | 3.17E-60 | 1.50E-57 | 126.3252531 |
| GPR153 | 1.276550712 | 2.41509817 | 18.78464003 | 4.04E-60 | 1.85E-57 | 126.0413863 |
| S100A2 | 2.126117271 | 5.104820351 | 18.781306 | 4.19E-60 | 1.86E-57 | 126.0491307 |
| TMCO4 | -0.509169132 | 5.368180554 | -18.66817534 | 1.47E-59 | 6.31E-57 | 124.7932772 |
| OSMR | 1.324207546 | 4.082653969 | 18.63974855 | 2.02E-59 | 8.39E-57 | 124.494742 |
| CCNA1 | 1.712258958 | 1.616621437 | 18.60482306 | 2.97E-59 | 1.20E-56 | 123.9218906 |
| KRT15 | 1.711776988 | 1.585297731 | 18.42675794 | 2.13E-58 | 8.36E-56 | 121.9654066 |
| CDC42EP3 | 1.322039808 | 4.249692333 | 18.34456123 | 5.29E-58 | 2.02E-55 | 121.2414472 |
| LGALS1 | 1.438826676 | 8.914859042 | 18.31633905 | 7.22E-58 | 2.68E-55 | 120.9373155 |
| CATSPER1 | 1.713466228 | 1.820513826 | 18.26609514 | 1.26E-57 | 4.55E-55 | 120.2566459 |
| COL8A2 | 2.130871907 | 5.951749722 | 18.18778451 | 2.98E-57 | 1.05E-54 | 119.508285 |
| GRHL3 | 1.627219614 | 2.174066385 | 18.1503218 | 4.51E-57 | 1.55E-54 | 119.0493178 |
| C16orf89 | -1.876117258 | 8.455743789 | -18.14221026 | 4.93E-57 | 1.65E-54 | 119.0229342 |
| DIS3L | -0.490015863 | 4.359371324 | -18.11105676 | 6.96E-57 | 2.28E-54 | 118.6711491 |
| ST6GALNAC5 | 2.846699977 | 2.182409726 | 18.027438 | 1.75E-56 | 5.58E-54 | 117.6715415 |
| HRH1 | 1.434523022 | 2.900732713 | 18.00358687 | 2.27E-56 | 7.09E-54 | 117.5046876 |
| KCNQ3 | 1.762120042 | 2.81687307 | 17.97295269 | 3.18E-56 | 9.72E-54 | 117.1635569 |
| RIC8B | -0.650794767 | 3.312455369 | -17.94875298 | 4.15E-56 | 1.24E-53 | 116.9129163 |
| ELF3 | 1.769547671 | 3.697882391 | 17.89292639 | 7.68E-56 | 2.24E-53 | 116.3016909 |
| LAMB3 | 2.81909078 | 6.15442985 | 17.88475811 | 8.40E-56 | 2.35E-53 | 116.1901526 |
| QSOX1 | 1.109982738 | 5.873658318 | 17.88632059 | 8.25E-56 | 2.35E-53 | 116.2005454 |
| SCTR | -1.035030821 | 0.706179861 | -17.85424034 | 1.17E-55 | 3.23E-53 | 115.5198275 |
| MICAL2 | 1.230712959 | 3.175269094 | 17.83555933 | 1.44E-55 | 3.88E-53 | 115.6760447 |
| ADAMTS14 | 1.850480553 | 1.334765947 | 17.81137713 | 1.88E-55 | 4.97E-53 | 115.202625 |
| BEAN1 | 1.334508495 | 1.765035644 | 17.79967263 | 2.14E-55 | 5.54E-53 | 115.1739082 |
| CSF2 | 1.9899282 | 1.997692793 | 17.74911461 | 3.73E-55 | 9.48E-53 | 114.6447672 |
| SFTPB | 4.419999928 | 7.488987404 | 17.74564608 | 3.87E-55 | 9.67E-53 | 114.6817295 |
| MVP | 0.980530894 | 7.107052957 | 17.68496315 | 7.53E-55 | 1.85E-52 | 114.0071755 |
| NXN | 1.231042462 | 4.984436874 | 17.67996383 | 7.95E-55 | 1.92E-52 | 113.9490863 |
| TAGLN2 | 0.873120391 | 9.1117777 | 17.67002211 | 8.87E-55 | 2.10E-52 | 113.8632477 |
| ITGA2 | 1.755700216 | 4.646982455 | 17.61180116 | 1.68E-54 | 3.91E-52 | 113.2149356 |
| CCL13 | 2.411557642 | 3.214404168 | 17.5906805 | 2.12E-54 | 4.84E-52 | 113.0036474 |
| KCNQ1 | -0.88058148 | 6.440677551 | -17.57405672 | 2.54E-54 | 5.72E-52 | 112.7925499 |
| ACTBL2 | 1.381269409 | 0.610375932 | 17.55004537 | 3.30E-54 | 7.32E-52 | 112.1967116 |
| SLC20A1 | 0.998213616 | 4.724462581 | 17.53392603 | 3.94E-54 | 8.59E-52 | 112.3588987 |
| MUC1 | 2.498983779 | 5.3134513 | 17.5212657 | 4.52E-54 | 9.71E-52 | 112.2246678 |
| MARVELD1 | 0.915313741 | 4.832472452 | 17.49389023 | 6.10E-54 | 1.29E-51 | 111.9212629 |
| STK38 | 0.666654176 | 5.030652505 | 17.4585296 | 8.98E-54 | 1.87E-51 | 111.5335623 |
| GPER1 | -1.316877741 | 3.832930775 | -17.44945574 | 9.92E-54 | 2.03E-51 | 111.4628943 |
| PMAIP1 | 1.656343674 | 3.117930203 | 17.44490895 | 1.04E-53 | 2.11E-51 | 111.4222726 |
| TGM2 | 1.533622837 | 5.676259169 | 17.42174942 | 1.34E-53 | 2.67E-51 | 111.1337517 |
| TNFSF9 | 1.970203461 | 1.583513765 | 17.41515858 | 1.44E-53 | 2.83E-51 | 110.9620693 |
| LOXL1 | 1.28325423 | 3.555212576 | 17.40811265 | 1.56E-53 | 3.02E-51 | 111.019214 |
| TPMT | -0.77596588 | 7.224727468 | -17.37368012 | 2.27E-53 | 4.33E-51 | 110.6186054 |
| TM4SF1 | 1.772100338 | 7.063121422 | 17.3565473 | 2.74E-53 | 5.15E-51 | 110.4323775 |
| MAP7D1 | 0.487158239 | 6.412201552 | 17.34797926 | 3.01E-53 | 5.58E-51 | 110.3321634 |
| FAXC | 1.122098006 | 2.255549039 | 17.30294547 | 4.91E-53 | 9.00E-51 | 109.8521975 |
| ZNF781 | -0.892122365 | 0.997656321 | -17.28614275 | 5.90E-53 | 1.07E-50 | 109.4736768 |
| EPHB3 | 1.659946295 | 3.723451843 | 17.23350964 | 1.05E-52 | 1.87E-50 | 109.1247244 |
| SLC25A42 | -0.890744345 | 5.018807977 | -17.214427 | 1.29E-52 | 2.27E-50 | 108.8832703 |
| MAPK10 | -0.745236744 | 1.251318355 | -17.19152163 | 1.65E-52 | 2.88E-50 | 108.5143591 |
| TMEM163 | 2.324272041 | 2.991154002 | 17.17927505 | 1.89E-52 | 3.25E-50 | 108.5395951 |
| ANKRD18A | -0.866646231 | 0.675641174 | -17.16934517 | 2.11E-52 | 3.57E-50 | 108.133616 |
| ADIPOR2 | -0.650547366 | 5.595208596 | -17.1567906 | 2.41E-52 | 4.05E-50 | 108.2555083 |
| MTARC2 | -1.037145964 | 5.01055078 | -17.01851475 | 1.09E-51 | 1.80E-49 | 106.7633196 |
| B4GALT5 | 0.813999163 | 5.600656781 | 16.99844504 | 1.35E-51 | 2.21E-49 | 106.5428283 |
| KRT19 | 2.592583241 | 8.083549466 | 16.99679758 | 1.37E-51 | 2.22E-49 | 106.5484134 |
| MTMR11 | 1.115851755 | 3.261029355 | 16.9720945 | 1.80E-51 | 2.87E-49 | 106.3050942 |
| DSC2 | 1.345586741 | 2.981006096 | 16.96593914 | 1.92E-51 | 3.03E-49 | 106.2409677 |
| AHNAK2 | 2.073593814 | 3.135270474 | 16.91328733 | 3.40E-51 | 5.31E-49 | 105.6736393 |
| MYT1 | -0.962244754 | 0.525983089 | -16.90477174 | 3.73E-51 | 5.76E-49 | 105.2577631 |
| AHR | 1.067968284 | 5.253767846 | 16.88751682 | 4.50E-51 | 6.87E-49 | 105.3471044 |
| PGM2L1 | 0.90393209 | 3.302276851 | 16.88620522 | 4.56E-51 | 6.89E-49 | 105.3777144 |
| S100A11 | 0.891879618 | 10.69394062 | 16.87815833 | 4.98E-51 | 7.43E-49 | 105.2929236 |
| TUBA1A | 0.90458125 | 6.515474963 | 16.84629964 | 7.03E-51 | 1.04E-48 | 104.9046952 |
| PTPRE | 1.540726895 | 4.187622087 | 16.82302319 | 9.05E-51 | 1.31E-48 | 104.676287 |
| LIPG | -1.524384315 | 5.81211441 | -16.82340391 | 9.01E-51 | 1.31E-48 | 104.6562744 |
| CXCL2 | 2.340773139 | 4.345635878 | 16.76238525 | 1.74E-50 | 2.50E-48 | 104.0294402 |
| MMP15 | -1.081916809 | 7.06142949 | -16.76022627 | 1.79E-50 | 2.53E-48 | 103.9830259 |
| BTBD11 | -1.625166806 | 3.256277579 | -16.74534317 | 2.10E-50 | 2.94E-48 | 103.8669155 |
| SERPINB8 | 0.697423139 | 3.118478074 | 16.74107533 | 2.20E-50 | 3.05E-48 | 103.8199108 |
| CTSC | 1.50799156 | 5.032123394 | 16.69142171 | 3.76E-50 | 5.16E-48 | 103.2402411 |
| LOX | 1.909626193 | 3.257666986 | 16.67305005 | 4.58E-50 | 6.23E-48 | 103.0915058 |
| LCA5L | -0.680447941 | 1.454623062 | -16.67061003 | 4.70E-50 | 6.33E-48 | 102.9680026 |
| YBX3 | 0.70792564 | 5.958398519 | 16.63660451 | 6.79E-50 | 9.05E-48 | 102.6448994 |
| SLC27A6 | 3.11582467 | 4.902203921 | 16.60685985 | 9.36E-50 | 1.23E-47 | 102.3570209 |
| CDH11 | 1.93335343 | 2.529932878 | 16.60636457 | 9.41E-50 | 1.23E-47 | 102.3679076 |
| SOX4 | 1.172600845 | 6.650274568 | 16.59211109 | 1.10E-49 | 1.42E-47 | 102.1725782 |
| CLDN10 | 3.044840452 | 2.788864549 | 16.58803881 | 1.15E-49 | 1.47E-47 | 102.1676704 |
| COL1A1 | 2.466452984 | 7.161522755 | 16.56028303 | 1.55E-49 | 1.97E-47 | 101.839475 |
| FN1 | 3.406859366 | 10.04839678 | 16.5318209 | 2.10E-49 | 2.65E-47 | 101.5700217 |
| ANKRD46 | -0.639224423 | 3.595605195 | -16.5253235 | 2.25E-49 | 2.81E-47 | 101.4913372 |
| EHBP1L1 | 0.863833273 | 4.513153618 | 16.48603813 | 3.44E-49 | 4.26E-47 | 101.0408613 |
| FAXDC2 | -1.053930917 | 4.253970575 | -16.4597969 | 4.56E-49 | 5.60E-47 | 100.7693368 |
| COMP | 2.892973964 | 4.229337799 | 16.45637169 | 4.73E-49 | 5.76E-47 | 100.7600297 |
| TNFRSF21 | 1.396032027 | 5.097538876 | 16.43534273 | 5.94E-49 | 7.15E-47 | 100.4920904 |
| XPR1 | 0.858944674 | 5.675324672 | 16.43194855 | 6.16E-49 | 7.35E-47 | 100.4505855 |
| OCIAD2 | 0.594920813 | 6.952852605 | 16.41834721 | 7.13E-49 | 8.44E-47 | 100.3130542 |
| FSTL3 | 1.368879013 | 5.492391464 | 16.38425475 | 1.03E-48 | 1.21E-46 | 99.94247549 |
| MEDAG | 1.516813021 | 4.332355467 | 16.37502213 | 1.14E-48 | 1.32E-46 | 99.86544477 |
| CEACAM6 | 3.171677355 | 2.355037717 | 16.36832104 | 1.22E-48 | 1.41E-46 | 99.79694036 |
| LEKR1 | -0.718356348 | 0.561658349 | -16.34644405 | 1.54E-48 | 1.77E-46 | 99.34022596 |
| TBC1D20 | -0.275274017 | 5.348764758 | -16.3418916 | 1.62E-48 | 1.84E-46 | 99.48697142 |
| ACOT9 | 0.406258661 | 4.596540703 | 16.3037795 | 2.44E-48 | 2.75E-46 | 99.08797835 |
| RTN3 | -0.514569654 | 7.371388892 | -16.287208 | 2.91E-48 | 3.25E-46 | 98.91708733 |
| MST1R | 1.182716643 | 3.076159048 | 16.27763812 | 3.23E-48 | 3.58E-46 | 98.86493558 |
| ACOT7 | 0.778560783 | 4.099206434 | 16.27429562 | 3.35E-48 | 3.68E-46 | 98.78919521 |
| PRKCQ | -0.782460251 | 4.430157482 | -16.27072828 | 3.48E-48 | 3.79E-46 | 98.74139238 |
| TRMT9B | -1.130715131 | 3.345764405 | -16.24975643 | 4.35E-48 | 4.71E-46 | 98.5611894 |
| DUSP5 | 1.920341216 | 7.334275725 | 16.22861878 | 5.46E-48 | 5.86E-46 | 98.29395268 |
| UPP1 | 1.212452663 | 5.199442655 | 16.22495042 | 5.68E-48 | 6.05E-46 | 98.24285699 |
| MRC1 | 1.659403735 | 3.375951947 | 16.18391382 | 8.81E-48 | 9.31E-46 | 97.86307713 |
| PTPRU | 1.405868937 | 4.597577243 | 16.18320386 | 8.88E-48 | 9.31E-46 | 97.80991006 |
| GATM | -1.477172096 | 3.387511022 | -16.15251045 | 1.23E-47 | 1.28E-45 | 97.52794703 |
| BATF3 | 0.996805127 | 1.531907628 | 16.11085022 | 1.93E-47 | 1.99E-45 | 97.0279115 |
| CMTM3 | 0.935846237 | 5.542740152 | 16.09808477 | 2.21E-47 | 2.26E-45 | 96.88936285 |
| CXCL8 | 2.052147517 | 2.903527706 | 16.0879719 | 2.46E-47 | 2.50E-45 | 96.85281428 |
| LDHD | -1.485670772 | 3.87976192 | -16.08649848 | 2.50E-47 | 2.52E-45 | 96.80855494 |
| P2RY6 | 1.434163519 | 2.544459065 | 16.06769244 | 3.05E-47 | 3.06E-45 | 96.6360589 |
| CDC42EP1 | 0.908997789 | 6.776115142 | 16.06610515 | 3.11E-47 | 3.09E-45 | 96.55605757 |
| TCEANC | -0.467016942 | 1.691839947 | -16.0589374 | 3.35E-47 | 3.31E-45 | 96.50090891 |
| GNAS | -1.054214576 | 9.746207455 | -16.05529071 | 3.49E-47 | 3.42E-45 | 96.48507074 |
| EPHA4 | 1.375231063 | 5.42034973 | 16.05345733 | 3.56E-47 | 3.46E-45 | 96.41747242 |
| SPOCK2 | 2.216415997 | 6.616926331 | 16.04754374 | 3.79E-47 | 3.66E-45 | 96.36138214 |
| TCIM | 1.890046171 | 7.11233421 | 16.02776051 | 4.68E-47 | 4.49E-45 | 96.15479412 |
| TMOD3 | 0.550308015 | 4.429711403 | 16.01560511 | 5.33E-47 | 5.08E-45 | 96.02500905 |
| VGLL1 | 2.183204228 | 1.245993435 | 16.01001471 | 5.65E-47 | 5.36E-45 | 95.90028311 |
| DPY19L1 | 0.767951168 | 4.363128998 | 15.99806918 | 6.42E-47 | 6.04E-45 | 95.84219975 |
| MGAT3 | 2.100115807 | 3.969118342 | 15.98905402 | 7.07E-47 | 6.61E-45 | 95.78023102 |
| PPP1R13B | -0.552365395 | 4.962700068 | -15.98787393 | 7.16E-47 | 6.62E-45 | 95.72105239 |
| PLVAP | -1.246267981 | 9.078912712 | -15.98761732 | 7.18E-47 | 6.62E-45 | 95.75730545 |
| ZNF346 | -0.438243096 | 3.371062638 | -15.98556891 | 7.34E-47 | 6.72E-45 | 95.7487672 |
| TMED4 | -0.459484494 | 8.001468032 | -15.97161561 | 8.51E-47 | 7.75E-45 | 95.5692477 |
| INPP5J | -1.367095059 | 7.544321399 | -15.94749962 | 1.10E-46 | 9.95E-45 | 95.30787242 |
| JAK3 | 1.414125692 | 3.106540174 | 15.94023138 | 1.19E-46 | 1.07E-44 | 95.2835535 |
| PDE7B | -1.412252562 | 2.652341289 | -15.93805848 | 1.22E-46 | 1.09E-44 | 95.26554065 |
| TBC1D2 | 1.171837104 | 6.301686841 | 15.91500383 | 1.56E-46 | 1.38E-44 | 94.9493801 |
| LYRM7 | -0.58880706 | 3.593655989 | -15.91393122 | 1.57E-46 | 1.39E-44 | 94.98085753 |
| ETHE1 | 1.004697932 | 5.890993416 | 15.88506041 | 2.14E-46 | 1.87E-44 | 94.62967591 |
| FAS | 0.894767114 | 4.199138119 | 15.8516163 | 3.06E-46 | 2.66E-44 | 94.29746138 |
| PON2 | 0.628399005 | 5.987113719 | 15.84145303 | 3.40E-46 | 2.94E-44 | 94.16769086 |
| RAP2B | 0.602852674 | 3.935906251 | 15.82456315 | 4.07E-46 | 3.50E-44 | 94.01918696 |
| SMIM3 | 0.746743779 | 5.432629259 | 15.79119679 | 5.81E-46 | 4.95E-44 | 93.63617201 |
| EHF | 1.930237712 | 3.540191261 | 15.78954804 | 5.91E-46 | 5.01E-44 | 93.68333641 |
| KCTD17 | 1.056283316 | 4.089186704 | 15.7721484 | 7.11E-46 | 5.99E-44 | 93.46392517 |
| TMEM37 | -0.827741905 | 6.447836889 | -15.7422387 | 9.76E-46 | 8.18E-44 | 93.12291773 |
| FAM20A | 2.035151489 | 5.229511787 | 15.72737519 | 1.14E-45 | 9.52E-44 | 92.97209328 |
| METTL7A | -0.819468367 | 8.534156616 | -15.72625333 | 1.16E-45 | 9.57E-44 | 92.98389743 |
| CST5 | 2.264172869 | 1.401373934 | 15.69454688 | 1.62E-45 | 1.33E-43 | 92.60938984 |
| TNC | 2.099205104 | 4.739665833 | 15.68035821 | 1.88E-45 | 1.54E-43 | 92.48868976 |
| CFI | 1.229469486 | 3.252826887 | 15.65811082 | 2.38E-45 | 1.93E-43 | 92.30166062 |
| SPX | -1.890326246 | 1.570800227 | -15.65807787 | 2.38E-45 | 1.93E-43 | 92.25592113 |
| TMPRSS6 | 3.229750953 | 4.00483878 | 15.64019939 | 2.88E-45 | 2.31E-43 | 92.11489364 |
| UST | -1.02943196 | 5.506965791 | -15.63547623 | 3.03E-45 | 2.42E-43 | 91.9941132 |
| S100A16 | 0.668277697 | 7.79732389 | 15.61949465 | 3.58E-45 | 2.85E-43 | 91.84679857 |
| LIF | 1.226776612 | 3.537307914 | 15.60105259 | 4.36E-45 | 3.44E-43 | 91.6891623 |
| PEBP1 | -0.650823195 | 11.29767131 | -15.59717076 | 4.54E-45 | 3.56E-43 | 91.6660473 |
| CRY2 | -0.587919528 | 5.31973981 | -15.58975634 | 4.91E-45 | 3.83E-43 | 91.51226364 |
| SUCLG1 | -0.483554174 | 6.316328032 | -15.56327437 | 6.50E-45 | 5.04E-43 | 91.23606821 |
| BTNL9 | -1.509969591 | 2.848992923 | -15.53463347 | 8.79E-45 | 6.78E-43 | 91.01850709 |
| ITGA3 | 1.081958609 | 8.078689748 | 15.53106435 | 9.13E-45 | 7.01E-43 | 90.92223389 |
| CXCL16 | 0.814474538 | 6.126626449 | 15.5225021 | 9.99E-45 | 7.63E-43 | 90.80688863 |
| PRSS23 | 1.558951135 | 6.174245033 | 15.52078823 | 1.02E-44 | 7.72E-43 | 90.7916895 |
| EBAG9 | -0.441771163 | 5.251991776 | -15.51379073 | 1.10E-44 | 8.27E-43 | 90.71388156 |
| DZANK1 | -0.570594777 | 2.104480329 | -15.49517566 | 1.33E-44 | 1.00E-42 | 90.60076539 |
| CTHRC1 | 2.152633876 | 3.866159509 | 15.48072327 | 1.55E-44 | 1.16E-42 | 90.4261993 |
| MACC1 | 1.487373275 | 2.718168068 | 15.47840376 | 1.59E-44 | 1.18E-42 | 90.4310345 |
| CDKN2B | 1.203342466 | 3.097548973 | 15.47326288 | 1.68E-44 | 1.24E-42 | 90.36695569 |
| IQCH | -0.702512261 | 1.130917934 | -15.46639129 | 1.81E-44 | 1.33E-42 | 90.21616697 |
| ZNF658 | -0.586219478 | 1.102645253 | -15.46248891 | 1.88E-44 | 1.38E-42 | 90.17229497 |
| NOX4 | 1.023362016 | 2.060602982 | 15.45336262 | 2.07E-44 | 1.51E-42 | 90.16103852 |
| TMEM92 | 2.143656828 | 1.802834137 | 15.45263269 | 2.09E-44 | 1.51E-42 | 90.12696422 |
| STK17B | 1.236073901 | 3.657180068 | 15.45130612 | 2.12E-44 | 1.52E-42 | 90.11176978 |
| SLC26A7 | -2.324889934 | 5.820842092 | -15.44286462 | 2.32E-44 | 1.66E-42 | 89.97793182 |
| SRGAP2 | 0.590968222 | 2.596072106 | 15.43796957 | 2.44E-44 | 1.74E-42 | 90.00824953 |
| MLEC | -0.692906802 | 7.08409406 | -15.43668057 | 2.47E-44 | 1.75E-42 | 89.91620394 |
| CD58 | 0.668475447 | 4.79132433 | 15.43204227 | 2.59E-44 | 1.83E-42 | 89.86210673 |
| KATNAL2 | -1.251176514 | 2.07652215 | -15.42462408 | 2.81E-44 | 1.97E-42 | 89.86044019 |
| MAP4 | 0.499615524 | 6.000624185 | 15.42050753 | 2.93E-44 | 2.04E-42 | 89.73555684 |
| GOLGA8N | -0.966416284 | 0.815379775 | -15.41217614 | 3.20E-44 | 2.22E-42 | 89.59940225 |
| ZC3H8 | -0.48148983 | 2.595761552 | -15.41100077 | 3.24E-44 | 2.24E-42 | 89.72632918 |
| CLEC2B | 0.973073997 | 3.596372702 | 15.40086019 | 3.60E-44 | 2.48E-42 | 89.58313264 |
| MISP | 2.133786766 | 2.655957669 | 15.39593611 | 3.80E-44 | 2.59E-42 | 89.56979173 |
| HAPLN3 | 1.276718868 | 3.231301694 | 15.39560613 | 3.81E-44 | 2.59E-42 | 89.54951156 |
| CELF2 | 1.011198426 | 3.419415264 | 15.39473286 | 3.84E-44 | 2.60E-42 | 89.52873507 |
| EML1 | -0.851361928 | 2.878118482 | -15.39295628 | 3.92E-44 | 2.64E-42 | 89.53258232 |
| PERP | 1.102125181 | 7.537996596 | 15.37811441 | 4.58E-44 | 3.07E-42 | 89.31003837 |
| ABTB2 | 1.364220153 | 3.979098459 | 15.36896671 | 5.04E-44 | 3.36E-42 | 89.2361815 |
| CTXN1 | 1.648108123 | 5.303611353 | 15.3635393 | 5.34E-44 | 3.54E-42 | 89.14471579 |
| S100A6 | 1.29926046 | 12.66672534 | 15.35076355 | 6.11E-44 | 4.03E-42 | 89.09590876 |
| TNFSF11 | 1.244805292 | 0.42612091 | 15.34448448 | 6.52E-44 | 4.29E-42 | 88.81680264 |
| ZMYND12 | -0.712408109 | 3.240193569 | -15.3427692 | 6.64E-44 | 4.34E-42 | 88.99275682 |
| CXCL14 | 3.799826699 | 6.614620104 | 15.3363354 | 7.11E-44 | 4.63E-42 | 88.87590986 |
| PRICKLE1 | 1.14538195 | 3.528371017 | 15.33555209 | 7.16E-44 | 4.64E-42 | 88.9065248 |
| ITGB6 | 1.327960722 | 4.017237024 | 15.33505978 | 7.20E-44 | 4.64E-42 | 88.87925121 |
| AP3M2 | -0.35916322 | 3.644675927 | -15.32465913 | 8.03E-44 | 5.16E-42 | 88.77920234 |
| ADGRF1 | 1.295312984 | 3.611507298 | 15.3222264 | 8.24E-44 | 5.27E-42 | 88.76547869 |
| UNC5CL | 1.373057897 | 2.759781593 | 15.31913391 | 8.51E-44 | 5.42E-42 | 88.76640984 |
| NT5E | 1.502005857 | 6.102456036 | 15.30174848 | 1.02E-43 | 6.47E-42 | 88.49645164 |
| IVL | 2.802527671 | 1.876354171 | 15.29872514 | 1.06E-43 | 6.65E-42 | 88.5193819 |
| CRLF1 | 3.002346347 | 5.081613354 | 15.29039622 | 1.15E-43 | 7.22E-42 | 88.40463216 |
| NFIA | -0.644576586 | 2.706484985 | -15.29006353 | 1.16E-43 | 7.22E-42 | 88.46269785 |
| SNX4 | -0.593681024 | 5.939463644 | -15.26499904 | 1.50E-43 | 9.35E-42 | 88.10882628 |
| KRT80 | 1.716047994 | 4.009197054 | 15.25427826 | 1.68E-43 | 1.04E-41 | 88.04325689 |
| COPZ1 | -0.312966596 | 7.494121297 | -15.23209474 | 2.12E-43 | 1.31E-41 | 87.78306309 |
| ALDH3B1 | 1.286413485 | 4.2827511 | 15.22882484 | 2.20E-43 | 1.35E-41 | 87.75790387 |
| CD70 | 2.038560897 | 1.387669937 | 15.21434638 | 2.56E-43 | 1.55E-41 | 87.61298431 |
| LMNA | 0.602841652 | 7.920320956 | 15.214754 | 2.55E-43 | 1.55E-41 | 87.60946639 |
| JPT1 | 0.687491226 | 4.832189601 | 15.19832573 | 3.03E-43 | 1.83E-41 | 87.41866448 |
| ESAM | -0.887447955 | 7.334076098 | -15.19775269 | 3.04E-43 | 1.83E-41 | 87.42299492 |
| ALOX15B | 2.329213212 | 4.960127801 | 15.19608667 | 3.10E-43 | 1.86E-41 | 87.41065063 |
| BBS4 | -0.482412923 | 4.285163809 | -15.18967324 | 3.31E-43 | 1.98E-41 | 87.34165143 |
| TCTA | -0.487527644 | 6.542978118 | -15.16897249 | 4.11E-43 | 2.45E-41 | 87.11200729 |
| IL1RAP | 1.344522498 | 3.177589768 | 15.16757581 | 4.18E-43 | 2.47E-41 | 87.1750682 |
| MMP14 | 1.021925646 | 6.889832494 | 15.16511248 | 4.28E-43 | 2.53E-41 | 87.07685954 |
| INAVA | 1.602860044 | 1.175074252 | 15.15376727 | 4.82E-43 | 2.83E-41 | 86.9683649 |
| PIAS2 | -0.408109321 | 2.75209164 | -15.14385747 | 5.35E-43 | 3.13E-41 | 86.94002938 |
| NNMT | 1.732973503 | 4.033475003 | 15.13686872 | 5.76E-43 | 3.35E-41 | 86.81918818 |
| BEND6 | 1.142005438 | 0.801009298 | 15.08943698 | 9.46E-43 | 5.48E-41 | 86.25667688 |
| ASB8 | -0.300568945 | 4.850878768 | -15.08863972 | 9.54E-43 | 5.50E-41 | 86.27526217 |
| C20orf96 | -0.566246128 | 4.894388767 | -15.08036316 | 1.04E-42 | 5.98E-41 | 86.18947647 |
| FNDC4 | 1.572764307 | 5.195651128 | 15.05220977 | 1.40E-42 | 7.99E-41 | 85.89970656 |
| TMSB4X | 1.055400055 | 10.92230247 | 15.04962316 | 1.43E-42 | 8.17E-41 | 85.94715893 |
| CDCP1 | 0.884272933 | 5.105537182 | 15.03437378 | 1.68E-42 | 9.54E-41 | 85.7107379 |
| INSL3 | 1.170540604 | 0.831646557 | 15.02576878 | 1.84E-42 | 1.04E-40 | 85.60619765 |
| SLC22A31 | 2.662012086 | 5.092513823 | 15.02304469 | 1.89E-42 | 1.07E-40 | 85.61476442 |
| WASF3 | -1.033579869 | 2.87144223 | -15.02167684 | 1.92E-42 | 1.08E-40 | 85.67121095 |
| IGFL2 | 2.531376353 | 2.292490459 | 15.01447965 | 2.07E-42 | 1.16E-40 | 85.60051771 |
| FAM117A | -0.604380783 | 4.617769718 | -15.01206174 | 2.12E-42 | 1.18E-40 | 85.48585269 |
| PDLIM4 | 2.335969831 | 6.357114409 | 14.99672869 | 2.49E-42 | 1.38E-40 | 85.32700235 |
| NDUFA5 | -0.500553434 | 4.895804456 | -14.995621 | 2.52E-42 | 1.39E-40 | 85.30945171 |
| DIRAS2 | -1.297620775 | 0.648502176 | -14.98647457 | 2.77E-42 | 1.52E-40 | 85.16851824 |
| FOSL2 | 1.007881893 | 5.574302392 | 14.9855389 | 2.80E-42 | 1.53E-40 | 85.20182322 |
| FAAH | -0.738876899 | 5.661784581 | -14.98070325 | 2.94E-42 | 1.60E-40 | 85.1506057 |
| SEMA3B | 1.313032341 | 3.927344117 | 14.97741125 | 3.04E-42 | 1.65E-40 | 85.16177458 |
| CACNG4 | 2.087173149 | 1.591586542 | 14.9768494 | 3.06E-42 | 1.66E-40 | 85.17900005 |
| HLA-G | 2.139852245 | 3.923238339 | 14.97373009 | 3.16E-42 | 1.70E-40 | 85.1398712 |
| MAFF | 1.111142509 | 4.170113069 | 14.97093155 | 3.26E-42 | 1.75E-40 | 85.07985752 |
| ANKLE2 | 0.381793135 | 4.295736553 | 14.96333843 | 3.53E-42 | 1.88E-40 | 84.98892121 |
| MDM1 | -0.445996738 | 2.838377793 | -14.96156388 | 3.59E-42 | 1.91E-40 | 85.04790822 |
| ELAC1 | -0.52751386 | 3.075222526 | -14.95642968 | 3.79E-42 | 2.01E-40 | 84.98400839 |
| ICAM1 | 1.520327549 | 6.0655763 | 14.94696556 | 4.18E-42 | 2.21E-40 | 84.80529087 |
| COL25A1 | -1.281432265 | 0.590023506 | -14.93612252 | 4.68E-42 | 2.46E-40 | 84.64120677 |
| ITFG2 | -0.377274019 | 4.650633661 | -14.92325471 | 5.35E-42 | 2.81E-40 | 84.56373843 |
| AP4S1 | -0.388802139 | 2.593036743 | -14.91238691 | 5.99E-42 | 3.13E-40 | 84.54774369 |
| SYNM | -0.96903106 | 2.47821147 | -14.90659701 | 6.37E-42 | 3.31E-40 | 84.49083291 |
| SFRP2 | 3.382015702 | 4.75492757 | 14.90613159 | 6.40E-42 | 3.32E-40 | 84.43154351 |
| CARD11 | 1.566163542 | 2.562307108 | 14.89860908 | 6.92E-42 | 3.57E-40 | 84.40823928 |
| TMEM63B | 0.639717474 | 5.468010524 | 14.89763791 | 6.99E-42 | 3.60E-40 | 84.29053138 |
| SCML2 | -0.692299315 | 0.867208965 | -14.89331284 | 7.31E-42 | 3.75E-40 | 84.25498366 |
| CDADC1 | -0.627385452 | 3.381132757 | -14.89202564 | 7.41E-42 | 3.77E-40 | 84.30162819 |
| BDH1 | -0.735961377 | 3.189746921 | -14.89232917 | 7.38E-42 | 3.77E-40 | 84.31681747 |
| USP30 | -0.333293103 | 3.913500403 | -14.88998148 | 7.57E-42 | 3.84E-40 | 84.24673324 |
| DCLK1 | 1.040051696 | 1.095034791 | 14.88052479 | 8.35E-42 | 4.22E-40 | 84.15194096 |
| ALOX5AP | 1.527226704 | 4.677938252 | 14.86327096 | 9.99E-42 | 5.02E-40 | 83.95277708 |
| ADAL | -0.597365346 | 2.87500707 | -14.86312962 | 1.00E-41 | 5.02E-40 | 84.03032601 |
| MLH3 | -0.38227799 | 3.594924548 | -14.85756628 | 1.06E-41 | 5.29E-40 | 83.93042906 |
| CCL17 | 2.599433691 | 2.999124699 | 14.81838141 | 1.59E-41 | 7.93E-40 | 83.57884613 |
| MBOAT2 | 1.001985337 | 4.491772879 | 14.81787763 | 1.60E-41 | 7.94E-40 | 83.48355828 |
| BEX3 | -0.635083012 | 8.748413847 | -14.80150584 | 1.90E-41 | 9.37E-40 | 83.34055481 |
| CCL20 | 1.882469729 | 1.487907 | 14.78582502 | 2.23E-41 | 1.10E-39 | 83.20997775 |
| MIDN | 0.749502596 | 6.298101685 | 14.77712769 | 2.44E-41 | 1.20E-39 | 83.04897144 |
| CCDC149 | -0.51371591 | 2.895742569 | -14.77591241 | 2.47E-41 | 1.21E-39 | 83.1304481 |
| SPTBN4 | -0.837509226 | 0.618251374 | -14.76519111 | 2.76E-41 | 1.35E-39 | 82.90304527 |
| SLC16A3 | 1.070319988 | 3.665229815 | 14.74631949 | 3.36E-41 | 1.63E-39 | 82.78564462 |
| NECTIN4 | 1.91843271 | 4.342067967 | 14.74147605 | 3.53E-41 | 1.71E-39 | 82.71750816 |
| ZNF546 | -0.491697228 | 1.531863641 | -14.74022247 | 3.58E-41 | 1.73E-39 | 82.75735317 |
| MAPK8 | -0.380569609 | 3.786166706 | -14.73586692 | 3.75E-41 | 1.80E-39 | 82.66429906 |
| HES2 | 1.043898992 | 0.565930643 | 14.71432746 | 4.68E-41 | 2.24E-39 | 82.37348272 |
| LUM | 2.637863056 | 5.587552456 | 14.71047639 | 4.87E-41 | 2.32E-39 | 82.37361214 |
| MROH8 | -0.615473499 | 1.773128317 | -14.7026366 | 5.28E-41 | 2.51E-39 | 82.38496381 |
[truncated: 932,733 more chars]
